# lncRNA CDKN2B-AS1 regulates collagen expression

**DOI:** 10.1007/s00439-024-02674-1

**Published:** 2024-06-04

**Authors:** Weiwei Shi, Jiahui Song, January Mikolaj Weiner, Avneesh Chopra, Henrik Dommisch, Dieter Beule, Arne S. Schaefer

**Affiliations:** 1 Dept. of Periodontology, Oral Medicine and Oral Surgery, Institute for Dental and Craniofacial Sciences, Charité - University Medicine Berlin, Berlin, Germany; 2 Core Unit Bioinformatics, Berlin Institute of Health at Charité, Berlin, Germany

## Abstract

The long noncoding RNA CDKN2B-AS1 harbors a major coronary artery disease risk haplotype, which is also associated with progressive forms of the oral inflammatory disease periodontitis as well as myocardial infarction (MI). Despite extensive research, there is currently no broad consensus on the function of CDKN2B-AS1 that would explain a common molecular role of this lncRNA in these diseases. Our aim was to investigate the role of CDKN2B-AS1 in gingival cells to better understand the molecular mechanisms underlying the increased risk of progressive periodontitis. We downregulated CDKN2B-AS1 transcript levels in primary gingival fibroblasts with LNA GapmeRs. Following RNA-sequencing, we performed differential expression, gene set enrichment analyses and Western Blotting. Putative causal alleles were searched by analyzing associated DNA sequence variants for changes of predicted transcription factor binding sites. We functionally characterized putative functional alleles using luciferase-reporter and antibody electrophoretic mobility shift assays in gingival fibroblasts and HeLa cells. Of all gene sets analysed, collagen biosynthesis was most significantly upregulated (P_ad_j=9.7 × 10^− 5^ (AUC > 0.65) with the CAD and MI risk gene *COL4A1* showing strongest upregulation of the enriched gene sets (Fold change = 12.13, P_adj_ = 4.9 × 10^− 25^). The inflammatory “TNFA signaling via NFKB” gene set was downregulated the most (P_adj_=1 × 10^− 5^ (AUC = 0.60). On the single gene level, *CAPNS2*, involved in extracellular matrix organization, was the top upregulated protein coding gene (Fold change = 48.5, *P* < 9 × 10^− 24^). The risk variant rs10757278 altered a binding site of the pathogen responsive transcription factor STAT1 (*P* = 5.8 × 10^− 6^). rs10757278-G allele reduced STAT1 binding 14.4% and rs10757278-A decreased luciferase activity in gingival fibroblasts 41.2% (*P* = 0.0056), corresponding with GTEx data. CDKN2B-AS1 represses collagen gene expression in gingival fibroblasts. Dysregulated collagen biosynthesis through allele-specific CDKN2B-AS1 expression in response to inflammatory factors may affect collagen synthesis, and in consequence tissue barrier and atherosclerotic plaque stability.

**Supplementary Information:**

The online version contains supplementary material available at 10.1007/s00439-024-02674-1.

## Introduction

*CDKN2B-*AS1 (CDKN2B antisense RNA 1; ANRIL) encodes a long non-coding RNA (lncRNAs), a class of molecules that are considered critical players of gene regulation in multiple biological processes. In general, lncRNAs act as transcriptional repressors, downregulating gene activity by directly interacting with the chromatin or mRNA of their target genes (Statello et al. 2021). Accordingly, CDKN2B-AS1 has been shown to regulate the neighboring genes *CDKN2A* and *CDKN2B* (cyclin dependent kinase inhibitors 2 A and 2B) (Yap et al. 2010) as well as the expression of distant genes (Holdt et al. 2013). CDKN2B-AS1 expression is particularly strong in gastrointestinal barrier tissues like small intestines and colon found in the Human Protein Atlas project from analysis of 27 different tissues in order to determine tissue-specificity implying a functional role of CDKN2B-AS1 for gene regulation in gastrointestinal tissues. A recent study that investigated the function of CDKN2B-AS1 in the intestines showed that cells with reduced CDKN2B-AS1 activity exhibited enhanced tissue barrier function and showed that here, CDKN2BAS-1 is expressed mainly in epithelial cells (Rankin et al. 2019).

Dysregulation of lncRNAs is associated with many complex diseases (Mercer et al. 2009; Zhao et al. 2016). Despite the biased expression in the colon, *CDKN2B-AS1* is the major genetic risk locus of coronary artery disease (CAD) (Consortium et al. 2013; WTCCC 2007). The CAD associated haplotype block (tagged by GWAS lead SNP rs1333049) is also associated with progressive early-onset forms of the oral inflammatory disease periodontitis (Schaefer et al. 2009) and myocardial infarction (MI) (Helgadottir et al. 2007b; Myocardial Infarction Genetics et al. 2009; Nikpay et al. 2015) (Munz et al. 2018; Schaefer et al. 2009). Genetic risk variants of this haplotype block have an influence on CDKN2B-AS1 transcript levels (Folkersen et al. 2009), implying a molecular biological link between susceptibility for these diseases and regulation of CDKN2B-AS1 expression.

A link of CDKN2B-AS1 to inflammation has also been established. Reduced CDKN2B-AS1 transcript levels repressed TNFA induced IL6 and IL8 expression (Zhou et al. 2016), whereas upstream in the inflammatory signaling cascade the pro-inflammatory cytokine IFNG stimulated CDKN2B-AS1 expression (Harismendy et al. 2011). Moreover, the CAD and MI GWAS lead SNP rs10757278 (Nikpay et al. 2015; Tcheandjieu et al. 2022), which is in strong linkage disequilibrium (LD, *r2* > 0.9) with rs1333049, disrupts a binding site for the immune signal transducer STAT1 (Harismendy et al. 2011).

However, in general, genes that had differential expression after increasing or decreasing CDKN2B-AS1 activity showed little overlap between the different studies, possibly due to the heterogeneity of the methods and cell models used.

The aim of the current study was to investigate the role of CDKN2B-AS1 in gingival fibroblasts. To this end, we investigated the cell type-specific downstream regulatory effects of CDKN2B-AS1 on gene expression in gingival fibroblasts. Here, we followed the rational of previous studies (Alfeghaly et al. 2021; Bochenek et al. 2013; Hubberten et al. 2019; Rankin et al. 2019), which suggested that overexpression of CDKN2B-AS1 can induce cellular stress and that in cells where CDKN2B-AS1 is already naturally expressed, overexpression does not lead to further downregulation of the potentially suppressed target genes because they would have already been silenced. In addition, we searched for biologically functional genetic variants in the associated haplotype block in order to obtain information about the upstream signaling events that regulate CDKN2B-AS1 activity.

Here, we show that CDKN2B-AS1 suppresses collagen synthesis in gingival fibroblasts and that the periodontitis and infarction associated susceptibility gene polymorphism rs10757278-G reduced STAT1 binding and increased CDKN2B-AS1 expression.

## Materials and methods

### Knockdown of CDKN2B-AS1 transcript levels by locked nucleic acids

LNA GapmeRs (single stranded antisense oligos) either targeting unique regions of CDKN2B-AS1 isoforms or non-targeting any region (scrambled, used as a negative control) were designed by QIAGEN as published before (Alfeghaly et al. 2021). A mix of four CDKN2B-AS1 LNA GapmeRs or a scrambled LNA GapmeR was used. The GapmeRs hybridized to unique regions of the main CDKN2B-AS1 isoforms as follows: gene globe ID LG00217779-DFA (exon 1), LG00217777-DFA (exon 7–13, numbered as in transcript EU741058), LG00217784-DFA (exon 12–13, numbered as in transcript DQ485454), LG00217785-DFA (exon 17–18, numbered as in transcript NR_003529). Primary gingival fibroblasts(pGFs) (from 3 different donators) and immortalized Human Gingival Fibroblast cells (iHGF) were sown with a density of 1.3 × 10^5^ cells / well in 6-well tissue culture plates (Techno Plastic Products, Switzerland) one day prior to LNA GapmeR transfection with Lipofectamine 2000 (Thermo Fisher Scientific, Waltham, USA) at a final concentration of 225 μm 48 h after transfection. Total RNA was extracted using the RNeasy Mini Kit (Qiagen, Germany).

### Cell culture

Primary gingival fibroblasts (pGFs) were isolated from gingival biopsies and cultured as previously described (Freitag-Wolf et al. 2019). In brief, pGFs cells and immortalized Human Gingival Fibroblast cells (iHGF) (ABM, Canada) were grown in Dulbecco’s modified Eagle’s medium (DMEM, PAN Biotech, Germany) supplemented with 10% fetal bovine serum (FBS, Gibco, USA), and 1% non-essential amino acids (MEM-NEAA, PAN Biotech). HeLa cells were cultured as recently described (Chopra et al. 2021). Briefly, cells were cultured in Earle’s MEM (Bio&Sell, Nuremberg, Germany) supplemented with 10% FBS, 2mM L-Glutamine (Bio&Sell), and 1%NEAA.

### Quantitative real-time PCR

Complementary DNA (cDNA) was synthesized from 100 ng total RNA, using the High-Capacity cDNA Reverse Transcription Kit (Applied Biosystems, Thermo Fisher Scientific). Quantitative real-time PCR (qRT-PCR) was performed using SYBR Select Master Mix (Applied Biosystems) to validate downregulation of CDKN2B-AS1 mRNA levels. The results were analyzed by using the 2 − ΔCT or 2 − ΔΔCT method and normalized to GAPDH as an internal control. The primer sequences were described in (Supplementary Materials Table. S1).

### RNA-sequencing

Total RNA was extracted from pGFs and iHGF cells using the RNeasy Mini Kit. 500 ng total RNA of transfected cell cultures were sequenced with 16 million reads (75 bp single end) on a NextSeq 500 using the NextSeq 500/550 High Output Kit v2.5 (75 cycles). RNA-Seq was performed at the Berlin Institute of Health, Core Facility Genomics. Reads were aligned to the human genome sequences (build GRCh38.p7) using the STAR aligner v. 2.7.8a (Dobin et al. 2013). Quality control (QC) of the reads was inspected using the multiqc reporting tool (Ewels et al. 2016) summarizing a number of approaches, including fastqc (available online at http://www.bioinformatics.babraham.ac.uk/projects/fastqc), dupradar (Sayols et al. 2016), qualimap (Garcia-Alcalde et al. 2012), and RNA-SeqC (DeLuca et al. 2012). Raw counts were extracted using the STAR program. For differential gene expression, the R package DESeq2 (Love et al. 2014), version 1.30 was used. Gene set enrichment was performed using the CERNO test from the tmod package (Zyla et al. 2019), version 0.50.07, using the gene expression profiling-based gene set included in the package, as well as the MSigDB (Liberzon et al. 2015), v.7.4.1. For the hypergeometric test and the Gene Ontology gene sets, the goseq package, version 1.38 (Young et al. 2010) was used. The *P* values of the differently expressed genes were corrected for multiple testing using Benjamini-Hochberg correction. The corrected *P* values are given as q values (false discovery rate [FDR]).

### Western blotting

To validate the expression of iHGF after knockdown the CDKN2B-AS1, the total protein of iHGF was extracted using RIPA with I.P. for 30 min on ice. The lysed samples were separated on polyacrylamide gels and transferred to a polyvinylidene fluoride (PVDF) membrane (Millipore, USA). Then the PVDF membranes were incubated with the primary antibody COL4A1(1:1000, Cell Signaling), Col 6A1 (1:500, Santa Cruz), CAPNS2(1:1000, Invitrogen) and β-actin (1:2000, Santa Cruz) overnight at 4 °C. Subsequently, the membranes were incubated with horseradish peroxidase (HRP)-conjugated secondary antibodies at room temperature for 1 h. The signal was acquired by using chemiluminescence detection (Chemostar Touch, INTAS, Indian). Then, ImageJ software was used to calculate the band intensities, and β-actin antibody was used as an internal control for signal normalization.

### Screening for functional periodontitis associated variants

For screening functional periodontitis associated variants, LD between the lead SNP rs1333049 and other common SNPs of this haplotype block was assessed using LDproxy Tool (Machiela and Chanock 2015) with population groups CEU (Utah Residents from North and West Europe) and GBR (British in England and Scotland). We assessed LD using *r*^*2*^ as a measure of correlation of alleles for 2 genetic variants (Supplementary Materials Fig. S2 and Table 1). We analyzed whether these SNPs located to chromatin elements that correlate with regulatory functions of gene expression provided from ENCODE (ENCODE-Project- Consortium 2012) (Supplementary Materials Fig. S3). To annotate eQTL effects of the associated SNPs, we used the software tool QTLizer (Munz et al. 2020). To investigate whether these SNPs changed predicted TFBSs, we used the TF databases Transfac (Thomas-Chollier et al. 2011) and the open-access database Jaspar (Sandelin et al. 2004). If Transfac as well as Jasper TF matrix files predicted a TF binding affinity at a SNP, with a stronger binding affinity at the common allele compared to the alternative allele, we selected the SNP for functional follow-up experiments. This conservative selection criterion was preassigned to avoid choosing false positive TFBS predictions for functional follow-up. TF binding motives were confirmed using the web interface for Position Weight Matrix (PWM) model generation and evaluation, PWMTools (Ambrosini et al. 2018).

**Table 1:** TFs predicted to bind at the SNP sequences with *p* < 0.01

SNP	Name	*P*-value (common allele)	*P*-value (rare allele)	*p*-value (combined)
rs10757278	V$STAT_01	0.0080	0.077	0.0052
rs7859727	V$GATA6_01	< 0.00057	0.824	0.0041

Calculated with the Transcription factor Affinity Prediction (TRAP) Web Tool TRAP (multiple sequences), using the matrix file ‘transfac_2010.1 vertebrates’, the background model ‘human_promoters’ and 10 bp +/- the SNP alleles:

rs10757278: CATTCCGGTA[A/G]GCAGCGATGC,

rs7859727: ATCTGAATGA[T/C]AGGCATTCCT

### Electrophoretic mobility shift assay (EMSA)

To characterize allele-specific DNA-protein interaction and TF STAT1 binding at rs10757278, we performed EMSAs with the Gelshift Chemiluminescent EMSA Kit (Active Motif, Germany) as recently described (Chopra et al. 2021). In brief, allele-specific oligonucleotide probes were synthesized (Metabiom International, Supplementary Materials Table S2). Nuclear protein extract was prepared from iHGF cells using the NE-PER Nuclear and Cytoplasmic Extraction Kit (Thermo Fisher). The double-stranded oligonucleotides corresponding to both alleles of rs10757278 flanked by 21 bp in both cold and 3′-biotinylated form were obtained by annealing with their respective complementary primers. For supershift EMSA, 20 fmol biotin-labeled, double-stranded oligonucleotides were incubated for 20 min with nuclear extract (5 µg) in 1x binding buffer and 2 µL of a specific monoclonal antibody (STAT1, 10 µg/50 µL each (Santa Cruz Biotechnology, California, USA) at room temperature. For competition assay, 4 pmol unlabeled double-stranded oligonucleotides were added to the binding reaction. The DNA-protein complexes were electrophoresed in a 5% native polyacrylamide gel in 0.5x TBE buffer at 100 V for 1 h. After electric transfer of the products on a nylon membrane and cross-linking, the biotinylated probes were visualized by chemiluminescence detection (Chemostar Touch, INTAS, Indian). Band intensities were quantified by the absolute value area of the shifted antibody bands using the software ImageJ (Rueden et al. 2017).

### Luciferase reporter gene assays

The putative regulatory DNA sequences (total length 539 bp) spanning 269 bp up- and downstream of the individual alleles of SNP rs10757278 were cloned into the firefly luciferase vector pGL4.24 (Promega, Madison, USA). The Further details were described in **Supplementary Materials**. iHGF cells were seeded at a density of 330,000 cells per 6-well before transfection with Lipofectamine 2000. HeLa cells were seeded at a density of 80,000 cells per well in 6-well plates and cultured until reaching 50–60% confluence. Transfection of HeLa cells was performed using the jetPEI transfection reagent (Polyplus transfection, France) following the manufacturer’s instructions. Cells were co-transfected in triplicates with 2.7 µg firefly luciferase reporter plasmid containing the putative regulatory sequence together with 0.3 µg renilla luciferase reporter vector (phRL-SV40, Promega) in 6-well plates for 24 h. In parallel, cells were transfected with the empty pGL4.24 plasmid and 0.3 µg phRL-SV40 as control. Firefly and renilla luciferase activities were quantified using the Dual-Luciferase Stop & Glo Reporter Assay System (Promega) with the Orion II Microplate Luminometer (Berthold Technologies). Relative fold changes (FC) in activities were normalized according to the manufacturer’s instructions (Promega) and differences of transcript levels were calculated with a T-Test using the software GraphPad Prism 9.

## Results

### CDKN2B-AS1 modulates the expression of collagen genes in gingival fibroblasts

In a first step, we quantified the expression of CDKN2B-AS1 in the gingiva to compare it with the expression in colon tissue, where this lncRNA is relatively highly expressed. We quantified the expression levels of CDKN2B-AS1 poly-A transcripts that terminate with exon-13 and − 19 (numbered as in transcript EU741058 and numbered as in transcript NR_003529, respectively) between healthy gingiva and colon biopsies using qRT-PCR. We found an equal expression in the gingiva compared to colon and similar expression of 3’-exon 13 and 3’-exon 19 transcripts between gingival and colon biopsies (Fig. S1A&B).

**Fig. 1:**
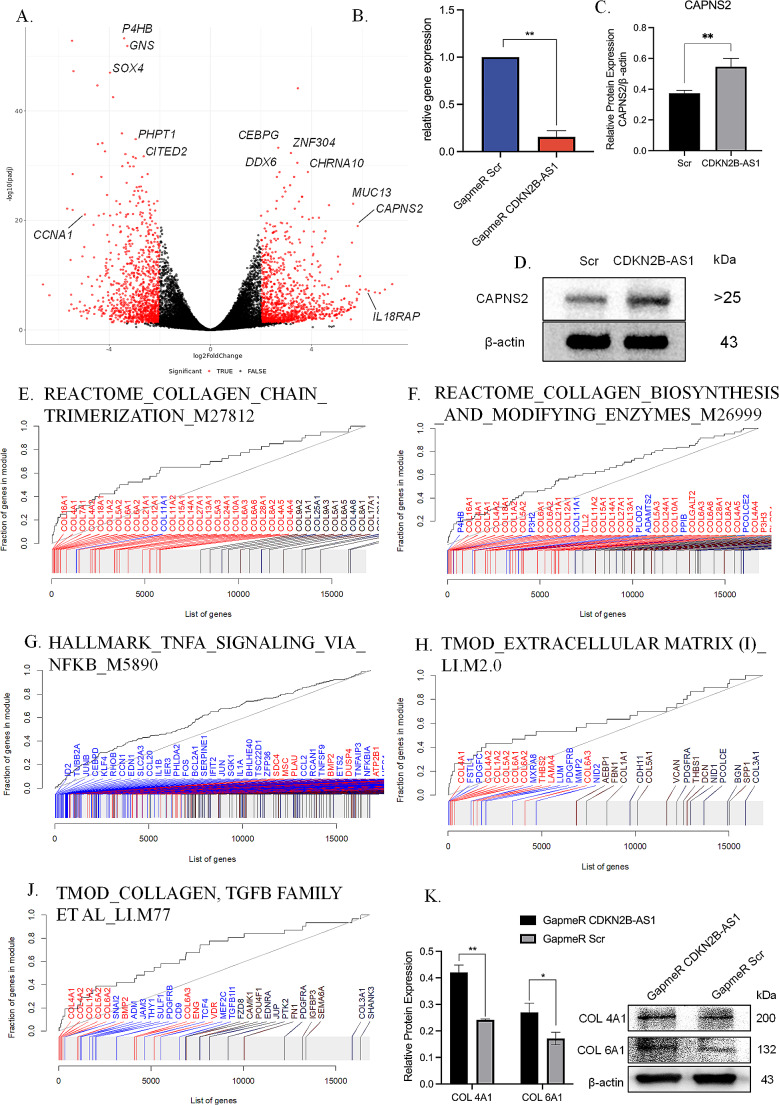
Differentially expressed genes and enriched gene sets in gingival fibroblasts after CDKN2B-AS1 knockdown. **(A)** Volcano plot of LNA GapmeRs transfected gingival fibroblasts showing differential expression of protein coding genes and numerous lncRNAs and pseudogenes. The names of the most significant differentially expressed protein coding genes are shown. The names of the most significant differentially expressed lncRNAs and pseudogenes are not shown to highlight the observed prominent role of CDKN2B-AS1 interaction with non-protein coding genes. **(B)** Transfection of primary gingival fibroblasts with LNA GapmeRs induced significant reduction of CDKN2B-AS1 transcript levels (qRT-PCR). **(C-D)** Western blot analysis validated that reduced CDKN2B-AS1 transcript levels correlated with significantly reduced CAPNS2 protein levels. Western Blot band intensities are normalized to *ACTB* (*p < 0.05; **p < 0.01). **(E-G)** Gene set enrichment analysis of GapmeR transfected gingival fibroblasts. Shown are evidence plots (receiver operator characteristic curves) for the significant gene sets with an area under the curve (AUC) ≥ 0.6. (C) From Reactome database, REACTOME_COLLAGEN_BIOSYNTHESIS_AND_ MODIFYING_ENZYMES _ M26999, enriched 62 genes. (D) From Reactome database, REACTOME_COLLAGEN_ CHAIN_TRIMERIZATION_ M27812, enriched 40 genes; (E) From Hallmark database, HALLMARK_TNFA_SIGNALING_VIA_ NFKB_ M5890, enriched 191 genes; (F&G) From Tmod database, EXTRACELLULAR MATRIX (I)_ LI.M2.0 enriched 30 genes and COLLAGEN, TGFB FAMILY ET AL_ LI.M77 enriched 31 genes, respectively. The gray rug plot underneath each curve corresponds to genes sorted by *P* value, with the genes belonging to the corresponding gene sets highlighted in red (upregulated genes) or blue (downregulated genes). Bright red or bright blue indicates that the genes are significantly regulated. **(H)** Western blotting validation of LNA GapmeRs transfected gingival fibroblasts showing *COL4A1* and *COL6A1* upregulation after CDKN2B-AS1 knockdown (*COL4A1* fold change = 1.74, *P* = 0.0004; *COL6A1* fold change = 1.57, *P* = 0.0155 respectively)

Silencing CDKN2B-AS1 expression in primary gingival fibroblasts using a mix of four LNA GapmeRs resulted in 84% reduction of CDKN2B-AS1 expression (Fig. 1B). Genomewide expression profiling by RNA-Seq following CDKN2B-AS1 knockdown revealed 1,167 upregulated genes with log_2_ Fold change (log_2_FC) > 2) and 2,829 downregulated genes with log_2_FC < -1 (P_adj_ < 0.05; Fig. 1A, Supplementary Materials Table S4). The most upregulated protein-coding gene with the lowest *p*-value was the gene *CAPNS2* (log_2_FC = 5.65 (*P* = 9 × 10^− 24^; Table 2; Fig. 1A). This gene is predicted to be involved in proteolysis [provided by Alliance of Genome Resources, Apr 2022] and based on gene content similarity is part of the gene cluster ‘Extracellular matrix organization’ (PathCard). Western blotting with protein extract of CDKN2B-AS1 knocked-down gingival fibroblasts showed a significant upregulation in the protein expression of the CAPNS2 (fold change = 1.5, *P* = 0.0063) (Fig. 1C**&D**). We also noticed that many lncRNAs were differentially expressed following CDKN2B-AS1 knockdown and separately list the top 10 differentially expressed ncRNAs in Table 3.

**Table 2:** Top 10 differentially expressed protein coding genes in gingival fibroblasts after CDKN2B-AS1 knockdown

	Gene	Log_2_ Fold change	IfcSE^†^	*P*_adj_(< 0.005)
Top Up-regulated	*IL18RAP*	6.261	1.065	7.20E-08
	*ATP13A5*	5.878	1.045	2.80E-07
	*CAPNS2*	5.645	0.532	9.00E-24
	*KERA*	5.497	1.113	0.000008
	*CMTM2*	5.486	1.15	0.000017
	*GSDMC*	5.441	1.329	0.00027
	*SLC5A12*	5.302	1.008	0.0000018
	*ASCL3*	5.298	1.263	0.00018
	*INSL5*	5.227	1.177	0.000069
	*GOLT1A*	5.194	1.475	0.002
Top Down-regulated	*CCNA1*	-4.961	0.489	8.30E-22
	*UBE2L5*	-4.561	1.012	0.000052
	*WDR87*	-4.481	0.671	6.70E-10
	*KLHDC7B*	-4.406	1.057	0.0002
	*TRBV12-4*	-4.378	0.998	0.000086
	*KRTAP2-4*	-4.338	1.052	0.00024
	*CCL20*	-4.141	0.549	2.1E-12
	*IL1B*	-4.122	0.556	5.0E-12
	*OR14K1*	-4.121	0.584	5.7E-11
	*LBX1*	-4.051	0.888	4.2E-5

^†^: Ifc: logarithmic fold change

**Table 3:** Top 10 differentially expressed lncRNA genes in gingival fibroblasts after CDKN2B-AS1 knockdown

	Gene	Log_2_ Fold change	IfcSE^†^	P_adj_(*P* < 0.005)
Top Up-regulated	*LINC00536*	7.192	1.129	4.40E-09
	*RGS5-AS1*	6.507	1.13	1.40E-07
	*LINC02516*	5.802	1.007	1.30E-07
	*LOC112268408*	5.793	1.106	0.000002
	*LINC01091*	5.597	0.634	1.20E-16
	*ENSG00000224478*	5.483	0.878	8.80E-09
	*LINC02435*	5.452	1.302	0.00019
	*ENSG00000272094*	5.385	1.336	0.00034
	*NPSR1-AS1*	5.274	1.347	0.00052
	*ENSG00000257894*	5.229	1.324	0.00046
Top Down-regulated	*NUP33-DT*	-6.618	1.043	5.00E-09
	*ENSG00000255317*	-5.662	0.544	7.10E-23
	*KIF18B-DT*	-5.528	0.7	1.60E-13
	*ENSG00000272226*	-5.498	1.166	0.000022
	*ZBED9-AS1*	-5.471	0.342	1.60E-53
	*ENSG00000274460*	-5.447	0.462	3.60E-29
	*ENSG00000277511*	-5.413	0.358	5.70E-48
	*ENSG00000272366*	-5.397	1.266	0.00014
	*USP12-AS1*	-5.141	1.3	0.00045
	*LINC02112*	-4.985	0.58	7.30E-16

^†^: Ifc: logarithmic fold change

We performed gene set enrichment analyses (GSEA) with the co-expression gene set tmod and the Molecular Signatures Database (MSigDB) gene sets reactome, hallmark, KEGG, and GO, by contrasting CDKN2B-AS1 knockdown cells to negative control transfected cells. The Reactome gene sets COLLAGEN_CHAIN_TRIMERIZATION (M27812) and COLLAGEN_BIOSYNTHESIS_AND_MODIFYING_ENZYMES (M26999) were most significantly upregulated, with P_adj_ = 9.7 × 10^− 5^ (AUC = 0.66 and 0.67) and Hallmark gene set TNFA_SIGNALING_VIA_NFKB (M5890) was most significantly downregulated with P_adj_ = 1.0 × 10^− 5^ (AUC = 0.60) (Fig. 1E-G). Additionally, Tmod gene sets “collagen, TGFB family (LI.M77) and extracellular matrix I (LI.M2.0) showed significant enrichment with P_adj_ = 0.01 (AUC = 0.67) and P_adj_ = 0.002 (AUC = 0.63), respectively (Fig. 1H**&J**).

### CDKN2B-AS1 knockdown increased COL4A1 and COL6A1 protein levels in gingival fibroblasts

We validated the positive effect of CDKN2B-AS1 knockdown on the expression of the collagen genes *COL4A1* and *COL6A1* by Western blotting. *COL4A1* showed the strongest upregulation on the RNA level in our RNA-Seq data (FC = 12.13, P_adj_ = 4.9 × 10^− 25^) and *COL6A1* (FC = 3.73, P_adj_ = 1.1 × 10^− 12^) was found in a previous ChIRP-Seq experiments to be a direct regulatory target of CDKN2B-AS1. Western blotting with protein extract of CDKN2B-AS1 knocked-down gingival fibroblasts showed a significant upregulation in the protein expression of the collagen genes *COL4A1* (fold change = 1.74, *P* = 0.0004) and *COL6A1* (fold change = 1.57, *P* = 0.0155) (Fig. 1K). These results proved *COL4A1* and *COL6A1* repression by CDKN2B-AS1.

### rs10757278-G allele reduced STAT1 binding in gingival fibroblasts

**Fig. 2:**
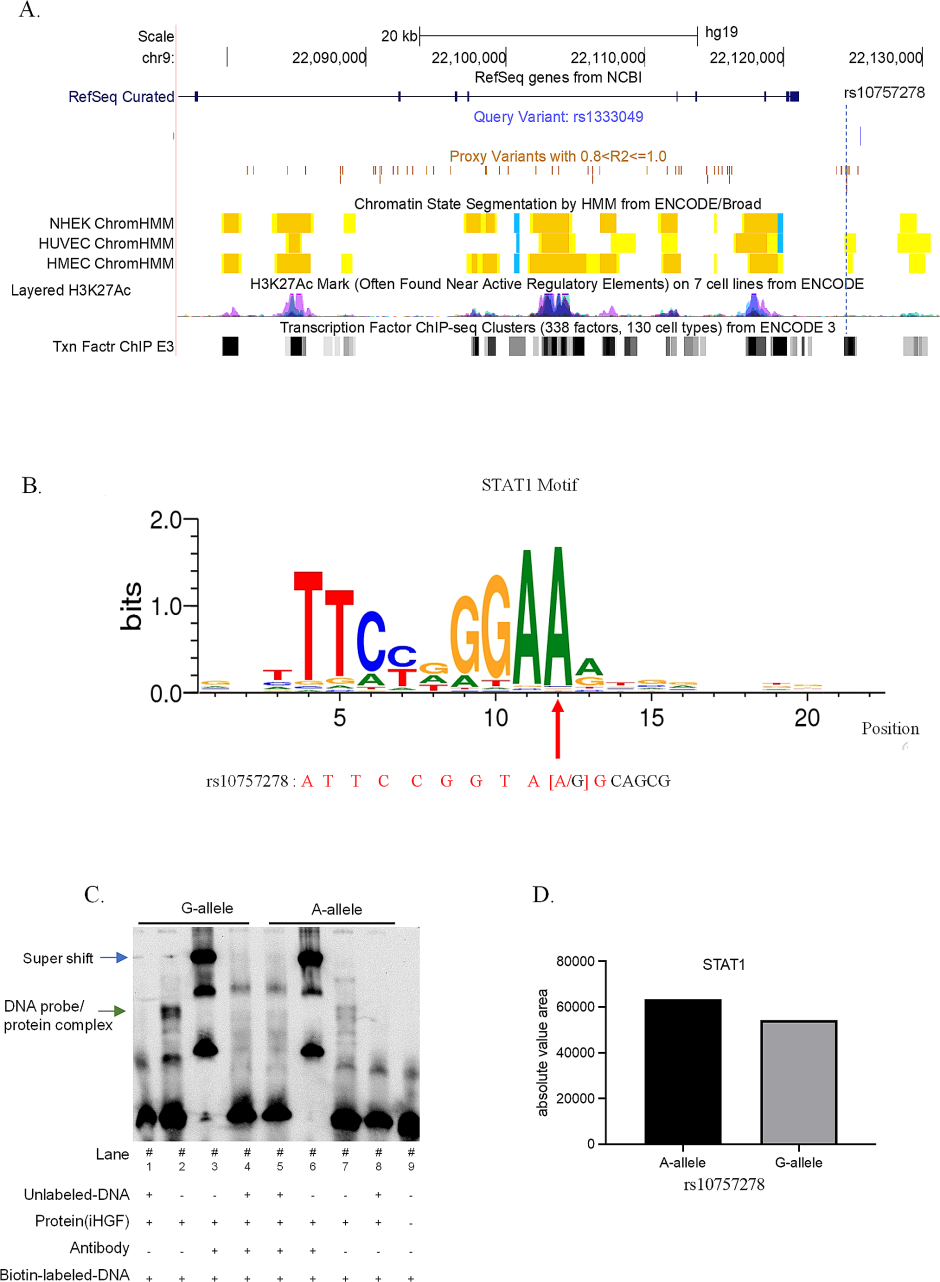
rs10757278 is a functional SNP within the chr9p21.3 risk haploblock and localizes to a STAT1 binding site. **(A)** 55 common SNPs (MAF ≤ 0.05) in CEU and GBR populations in strong LD with the GWAS lead SNP rs1333049 and 24 SNPs located within chromatin elements that correlate with regulatory functions of gene expression, which is indicated by chromatin state segmentation for 3 cell types (data from ENCODE; orange = predicted strong enhancer, yellow = weak enhancer, blue = insulator). Some proxy SNPs locate in H3K4me1 and H3K27ac methylation marks, which are often associated with regulation of gene transcription and within TFBS that were determined from ENCODE ChIP-Seq data. The position of rs10757278 is marked with a dashed line. **(B)** The DNA sequence at rs10757278-A allele shares a matrix similarity of 95% with the STAT1 transcription factor (TF) binding motif. **(C)** EMSA was performed with rs10757278 allele-specific oligonucleotide probes and nuclear protein extract from gingival fibroblast cells. Binding of STAT1 antibody to allele-specific probes is shown in lane 2 and 7. The supershift caused by STAT1 antibody binding to the DNA probe-protein complex is seen in lane 3 and 6. Unlabeled DNA was added in lanes 1 and 8 to verify that the band shift was antibody-specific. **(D)** Absolute value area of the antibody-specific bands. In the background of rs10757278-G allele, STAT1 binding to the allele-specific oligonucleotide probe was reduced 14.4% compared to the A-allele

We next searched for putative causal variants within the CAD/periodontitis/MI risk haplotype block that were located within chromatin stretches marked with biochemical modifications characteristic of regulatory DNA elements. The CAD GWAS lead SNP rs1333049 is in strong LD (*r*^*2*^ > 0.8) with 55 common SNPs (minor allele frequency ≤ 0.05) in North-West European populations (1000 genomes population codes CEU and GBR). Of these, 24 SNPs located within chromatin elements with biochemical marks assigning them as putative regulatory elements (Fig. 2A, Supplementary Materials Table S3). We computationally analyzed whether the alternative alleles of these 24 SNPs changed predicted transcription factor binding sites (TFBSs). We found that Transfac and Jasper TF matrix files predicted TFBSs at two SNPs (rs10757278 and rs7859727) with the alternative alleles decreasing TF binding affinities (Table 1). rs10757278 locates within a STAT1 binding site with the common A allele being part of the STAT1 binding motif (*P* = 5.8 × 10^− 6^) and a STAT1 matrix similarity of 94.8% (Fig. 2B). In transfac_2010.1 vertebrates matrix files, the alternative rs10757278-G allele 3,057-fold reduced binding affinity (*P* = 0.0177). rs10757278-G was described before as a putative causal variant for CAD affecting a STAT1 binding site (Harismendy et al. 2011). STAT1 is strongly expressed in gingival fibroblast (qRT-PCR threshold cycle [C_t_] value for STAT1 = 20.45, C_t (GAPDH)_ = 14.61), indicating biological activity of STAT1 in this cell type (Supplementary Materials Fig. S4).

rs7859727-T locates within a predicted GATA binding site (*P* = 0.0006). In transfac_2010.1 vertebrates matrix files, the alternative rs10757278-C allele 836-fold reduced binding affinity (*P* = 0.549). However, RNA-Seq data of healthy gingival biopsies (Richter et al. 2019) showed GATA expression in gingival tissues below detection limit, indicating that this tissue does not express GATA.

Allele specific STAT1 binding at rs10757278 was described by Chromatin Immunoprecipitation (ChIP) in lymphoblastoid cells (LCL) before (Harismendy et al. 2011). We validated rs10757278 allele specific STAT1 binding with protein extract isolated from gingival fibroblasts and performed a STAT1 antibody EMSA with DNA probes that contained either rs10757278 ref-A allele or alt-G allele. Using protein extract of gingival fibroblasts, STAT1 binding at DNA probes with rs10757278 alt-G allele was 14.4% reduced compared to the ref-A allele (Fig. 2C**&D**). The observation of increased STAT1 binding at the G allele corresponded with the previous observation in LCL cells.

### STAT1 binding at rs10757278 repressed gene activity

**Fig. 3:**
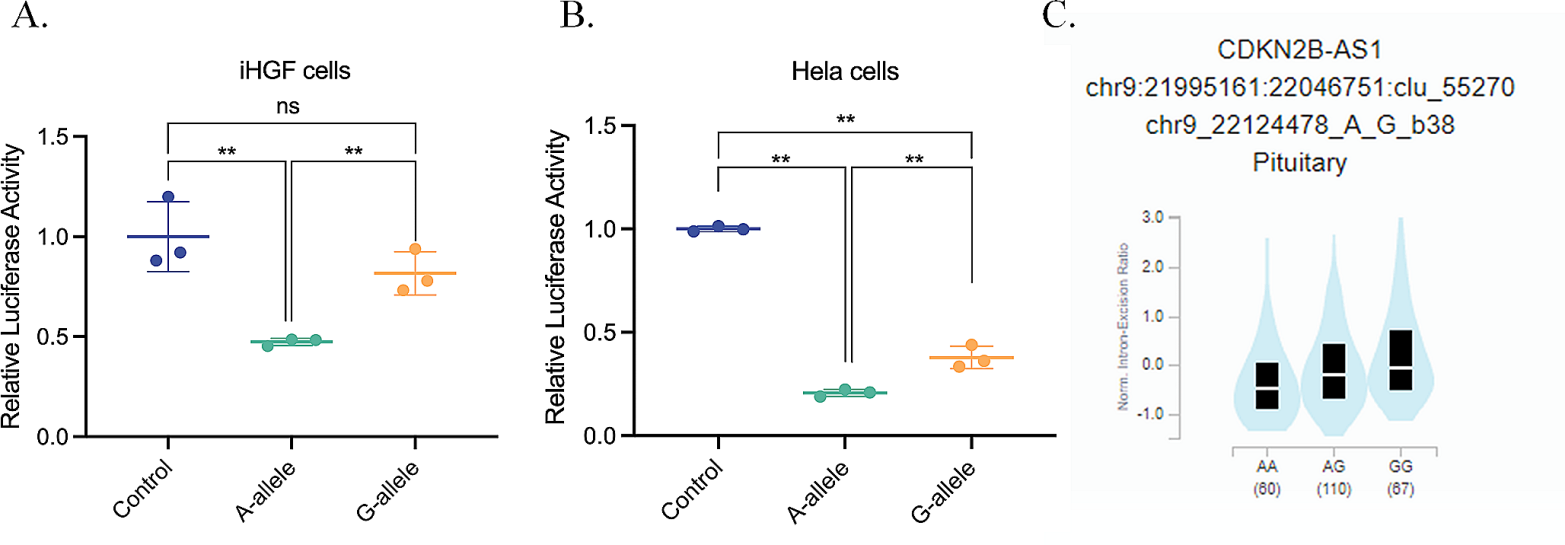
rs10757278 STAT1 binding A-allele reduces reporter gene activity. **(A, B)** rs10757278-A significantly reduced luciferase activity in gingival fibroblasts (fold change = 1.72-fold, *P* = 0.0056) and in HeLa cells (fold change = 1.83-fold, *P* = 0.0063). (Control = empty pGL4.24 plasmid, *p < 0.05; **p < 0.01). **(C)** GTEx data indicate a cis-eQTL effect for rs10757278 on *CDKN2B*-AS1 expression (tissue: pituitary gland) with rs10757278-A significantly reducing *CDKN2B-AS1* expression compared to the G allele (*p* = 3.9 × 10^− 6^)

We tested the regulatory effect direction of the DNA element at rs10757278 in gingival fibroblasts using luciferase reporter gene assays. The DNA sequence containing the STAT1 binding allele rs10757278-A decreased luciferase activity in gingival fibroblasts 41.2%, when compared with the common G-allele (*P* = 0.0056; Fig. 3A). We validated this finding in HeLa cells, because STAT1 is also expressed in this cell type, which can be efficiently transfected in vitro. Luciferase reporter gene transfection into HeLa cells confirmed decreased luciferase activity in the background of the rs10757278-A allele compared with the alternative G-allele (*P* = 0.0063; Fig. 3B). These results corresponded with GTEx data that also showed reduced CDKN2B-AS1 expression in homozygous carriers of the A allele compared to the G- allele (*P* = 3.9 × 10^− 6^; Fig. 3C). Taken together, these results demonstrated that the reference rs10757278-A allele is part of a biological functional STAT1 binding site that acts as a transcriptional repressor.

## Discussion

In the current work, we provide evidence that *CDKN2B-AS1* represses expression of collagen genes in gingival fibroblasts and that *CDKN2B-AS1* is under negative control of the inflammatory transcription factor STAT1.

A previous study, which used the identical set of GapmeRs for CDKN2B-AS1 knockdown in the kidney cell line HEK293 and subsequently combined chromatin immune RNA precipitation followed by sequencing (ChIRP-Seq) with genomewide expression profiling, found that CDKN2B-AS1 directly contacted and regulated collagen gene expression (Alfeghaly et al. 2021). This study showed upregulation of the genes *COL6A1* and *COL12A1* and concluded that these collagen genes were direct targets of CDKN2B-AS1. However, this study did not find any enriched pathways in the list of direct trans-regulated genes. The immortalized kidney cell line HEK293 has a different expression pattern and differentiation state compared with primary gingival fibroblasts. Accordingly, it may not fully represent the biological functions, which CDKN2B-AS1 has in gingival fibroblasts, where this gene is naturally expressed at comparatively high levels. Therefore, to validate the findings of this previous study, we used the identical set of gapmers to reduce CDKN2B-AS2 transcript levels in gingival fibroblasts. Here, we gave evidence that collagen synthesis pathways were the most enriched gene sets in response to suppression of CDKN2B-AS1 transcript levels. We also proved on the protein level that in gingival fibroblasts CDKN2B-AS1 negatively controls the collagen genes *COL6A1*, but also *COL4A1*, which was the strongest upregulated collagen gene in our study. Moreover, as a novel finding, we detected that *CAPNS2*, which encodes the Calpain Small Subunit 2, was the most upregulated gene after CDKN2B-AS1 knockdown. Calpains are calcium-activated cysteine proteases that act as part of numerous intracellular signaling pathways. Of particular interest, calpain activity is required for differentiation/activation of fibroblasts, which lay down extracellular collagen matrix (ECM) proteins (e.g. collagen). In response to tissue injury, calpain is activated and promotes, in addition to the expression and release of proinflammatory cytokines (Ji et al. 2016), the activation and differentiation of fibroblasts, thereby promoting the production of collagens (Scaffidi et al. 2002). In contrast it was shown that inhibition of calpains interrupt the early steps of fibroblast activation and differentiation, thereby attenuating the production of collagen (Kim et al. 2019; Letavernier et al. 2012). Therefore, collagen production and accumulation as seen in repeated injury, chronic inflammation and wound healing requires precise regulation to avoid fibrosis and to maintain barrier tissue function. Our data indicate CDKN2B-AS1 regulates CAPNS2 activity.

Additionally, our gene pathway enrichment analysis also revealed, in response to CDKN2B-AS1 repression, a significant downregulation of the pathway ‘TNFA Signaling via NFKB’ including significant repression of the genes *IL1A* and − *1B*. Consistent with this observation, it was previously shown that knockdown of CDKN2B-AS1 transcripts in endothelial cells inhibited TNFA induced IL6 and IL8 expression (Zhou et al. 2016). It has long been known that endogenous TNFA down-regulates collagen synthesis during normal wound healing (Regan et al. 1993) and that TNFA inhibits collagen-alpha gene expression in cultured fibroblasts (Buck et al. 1996). Considering the association of CDKN2B-AS1 with severe, progressive periodontitis, our data imply that CDKN2B-AS1 is a molecular regulator that aligns TNFA signaling and collagen synthesis in gingival fibroblasts.

Our data also confirmed the previously described role of rs10757278, a risk SNP for CAD (Tcheandjieu et al. 2022) and MI (Helgadottir et al. 2007a), as being a putative causal variant of this risk haplotype block, which impairs binding of the IFNG responsive signal transducer STAT1 (Harismendy et al. 2011). We confirmed STAT1 binding at this SNP sequence and showed that the rs10757278-G allele reduced STAT1 binding and increased reporter gene expression in gingival fibroblasts. GTEx data also reported increased CDKN2B-AS1 expression in homozygous carriers of the G-allele compared to the A-allele, confirming our result.

Validation of the results of previous work through an independent approach in a cell type in which CDKN2B-AS1 is naturally and comparatively highly expressed represents a significant value of our work by emphasizing these results and placing them in a new functional context. This context lies in the regulation of collagen synthesis in a barrier tissue, possibly in response to an inflammatory phase.

Periodontitis is characterized by recurrent and prolonged inflammation and gingival bleeding. Periodontal healing after active inflammation requires reconstruction of the gingival barrier tissues. These wound healing processes include tissue formation and tissue remodeling, which follow but partially overlap with inflammation (Yen et al. 2018). To fully restore the tissue barrier, these two processes of healing from gingival bleeding involves collagen deposition and collagen remodeling, respectively.

The CDKN2B-AS1 rs10757278-G allele is also associated with increased risk for MI (Helgadottir et al. 2007a). Of note, *COL4A1*, which is regulated by CDKN2B-AS1 shown in the current study for gingival fibroblasts and also previously for HEK293 cells (Alfeghaly et al. 2021), is also a risk gene for MI (Nikpay et al. 2015). Collagen is a critical component of atherosclerotic lesions and constitutes up to 60% of total plaque protein (Rekhter et al. 1996; Smith 1965). High collagen contributes to plaque structural integrity and mechanical “strength”. Therefore, a deficit of collagen reinforcement leads to plaque weakness and vulnerability (Burleigh et al. 1992; Lee and Libby 1997), making atherosclerotic plaque prone to rupture (Rekhter 1999), increasing the risk for MI. The in vitro results of the current study provide a functional link between the two MI risk genes *CDKN2B-AS1* and *COL4A1*. Our study implies that the rs10757278-G allele leads to reduced CDKN2B-AS1 repression by reducing STAT1 binding. As a result, higher CDKN2B-AS1 would in turn lead to increased collagen repression. This could destabilize atherosclerotic plaque and weaken the gingival tissue barrier, independently increasing the risk for MI and periodontitis.

A limitation of the current study is that we have not shown that CDKN2B-AS1 directly binds to the collagen genes in gingival fibroblasts, e.g. by RNA immune precipitation. However, we consider our finding that CDKN2B-AS1 knockdown in gingival cells upregulated collagen genes and the results from our protein blots as a validation of the previous ChIRP-Seq results obtained in HEK293 cells (Alfeghaly et al. 2021). Another limitation of our study was that we silenced the expression of all major CDKN2B-AS1 isoforms in parallel by using a mixture of four LNA-GapmeRs that hybridized to individual regions of the major CDKN2B-AS1 isoforms exon1 (all isoforms), exon17-18 (NR isoform), exon12-13 (DQ isoform), and exon7-13 (EU isoform). It is possible that only one particular isoform regulates the expression of collagen genes. Future work is needed to determine whether a specific isoform or all major isoforms contribute to this process.

In conclusion, the results provide an explanation for the molecular mechanisms underlying the shared genetic risk for periodontitis and MI at CDKN2B-AS1. This mechanism implies that the effect on each disease is independent of the presence of the other diseases, an effect known as horizontal pleiotropy. Validation of this explanation of the epidemiological connection between periodontitis and MI, e.g. by correlating the carriage of the risk allele rs10757278-G in MI patients with the collagen content of their atherosclerotic plaques, could give final proof of the causality of the proposed genetic effect. We conclude that this genetic effect is caused by reduced STAT1 binding affinities at the risk G allele, leading to increased CDKN2B-AS1 expression with corresponding increased TNFA signaling, as suggested by our data. This would cause a prolongation of the inflammatory reaction and thereby contribute to disease progression.

### Electronic supplementary material

Below is the link to the electronic supplementary material.

Supplementary Material 1

## Data Availability

RNA-Seq datasets are accessible in the Gene Expression Omnibus GEO under the accession number GSE266478 https://www.ncbi.nlm.nih.gov/geo/query/acc.cgi?acc=GSE266478.
