# Supplementary material for: lncRNA CDKN2B-AS1 regulates collagen expression
Source: Hum Genet. 2024 Jun 4;143(7):907–19. doi: 10.1007/s00439-024-02674-1 (PMC11294485; doi:10.1007/s00439-024-02674-1)
Supplement: Supplementary file 1 — Supplementary Material 1 [file 439_2024_2674_MOESM1_ESM.docx]

**Supplemental material**

**lncRNA CDKN2B-AS1 regulates collagen expression**

**Human Genetics**

Weiwei Shi^1^, Jiahui Song^1^, January Mikolaj Weiner 3rd ^2^, Avneesh Chopra^1^, Henrik Dommisch^1^, Dieter Beule^2^, Arne S Schaefer^1^

1. Dept. of Periodontology, Oral Medicine and Oral Surgery, Institute for Dental and Craniofacial Sciences, Charité - University Medicine Berlin, Germany

2. Core Unit Bioinformatics, Berlin Institute of Health at Charité, Berlin, Germany

**Email address corresponding author:** arne.schaefer@charite.de

**Supplementary Methods and Materials**

**Table S1.** Oligonucleotides sequences of qRT-PCR

| Primer name | Sequence (5’-3’) |
| --- | --- |
| GAPDH_Fw | GCATCTTCTTTTGCGTCG |
| GAPDH_Rev | TGTAAACCATGTAGTTGAGGT |
| CDKN2B-AS1_Fw | ATCTCTGTTTTCTGGCCACCA |
| CDKN2B-AS1_Rev | GGATTCCAGCACACCTAACAGT |
| CDKN2B-AS1_Exon13_Fw | GGACATTGGACAAAAACACAGA |
| CDKN2B-AS1_Exon13_Rev | GCAGGTATCATTCTCCTCAA |
| CDKN2B-AS1_Exon19_Fw | GGATGCATGAGCTATTGAGG |
| CDKN2B-AS1_Exon19_Rev | ATTTGCAAAAACAGCTGACC |

**Luciferase reporter gene assays**

The putative regulatory DNA sequences (total length 539 bp) spanning 269 bp up- and downstream of the individual alleles of SNP rs10757278 were cloned into the firefly luciferase vector pGL4.24 (Promega, Madison, USA).

**Cloning**

Genomic DNA (gDNA) was extracted from human cells using the AllPrep DNA/RNA/miRNA Universal Kit (Qiagen). The purified gDNA was used as a PCR-template. The DNA sequences spanning the putative causal SNPs were amplified by PCR to subsequently test their regulatory potential on reporter gene expression.

The PCR product was amplified using Phusion® High-Fidelity DNA Polymerase (NEB) with forward and reverse primers containing the KpnI restriction sites. The primer sequences were:

| Primer name | Sequence (5’-3’) |
| --- | --- |
| rs10757278_Fw | CGGGGTACCTGAGGTCGCAACTAAAAGCCA |
| rs10757278_Rev | CGGGGTACCTCCACGCTGTTCCCAAGTAG |

The PCR product was purified using QIAquick gel extraction kit (QIAGEN) and ligated to the KpnI digested plasmid pGL4.24 (Promega). The modified plasmid was purified using the QIAprep Spin Miniprep Kit. The allele was exchanged with the Q5 site-directed mutagenesis kit (NEB) according to the manufacturer’s instructions. The primer sequences were:

| Primer name | Sequence (5’-3’) |
| --- | --- |
| Q5_rs10757278_G_Fw | CATTCCGGTAGGCAGCGATGC |
| Q5_rs10757278_G_Rev | ACCACACCCTGACTTGTC |

**Electrophoretic Mobility Shift Assay (EMSA)**

**Supplementary Table S2.** Oligonucleotides sequences of EMSA probes

| Primer name | Sequence (5’-3’) |
| --- | --- |
| rs10757278_A_Fw | CAGGGTGTGGTCATTCCGGTAAGCAGCGATGCAGAATCAAGAC |
| rs10757278_A_Rev | GTCTTGATTCTGCATCGCTGCTTACCGGAATGACCACACCCTG |
| rs10757278_G_Fw | CAGGGTGTGGTCATTCCGGTAGGCAGCGATGCAGAATCAAGAC |
| rs10757278_G_Rev | GTCTTGATTCTGCATCGCTGCCTACCGGAATGACCACACCCTG |

**Statistical analysis**

Statistical analyses were performed by Student’s paired t-test or One-way analysis of variance (ANOVA), as appropriate (GraphPad Prism; GraphPad Software, La Jolla, CA, USA). A p-value < 0.05 was considered statistically significant. All data are expressed as means ± standard deviation.

**Supplementary Fig. S1**


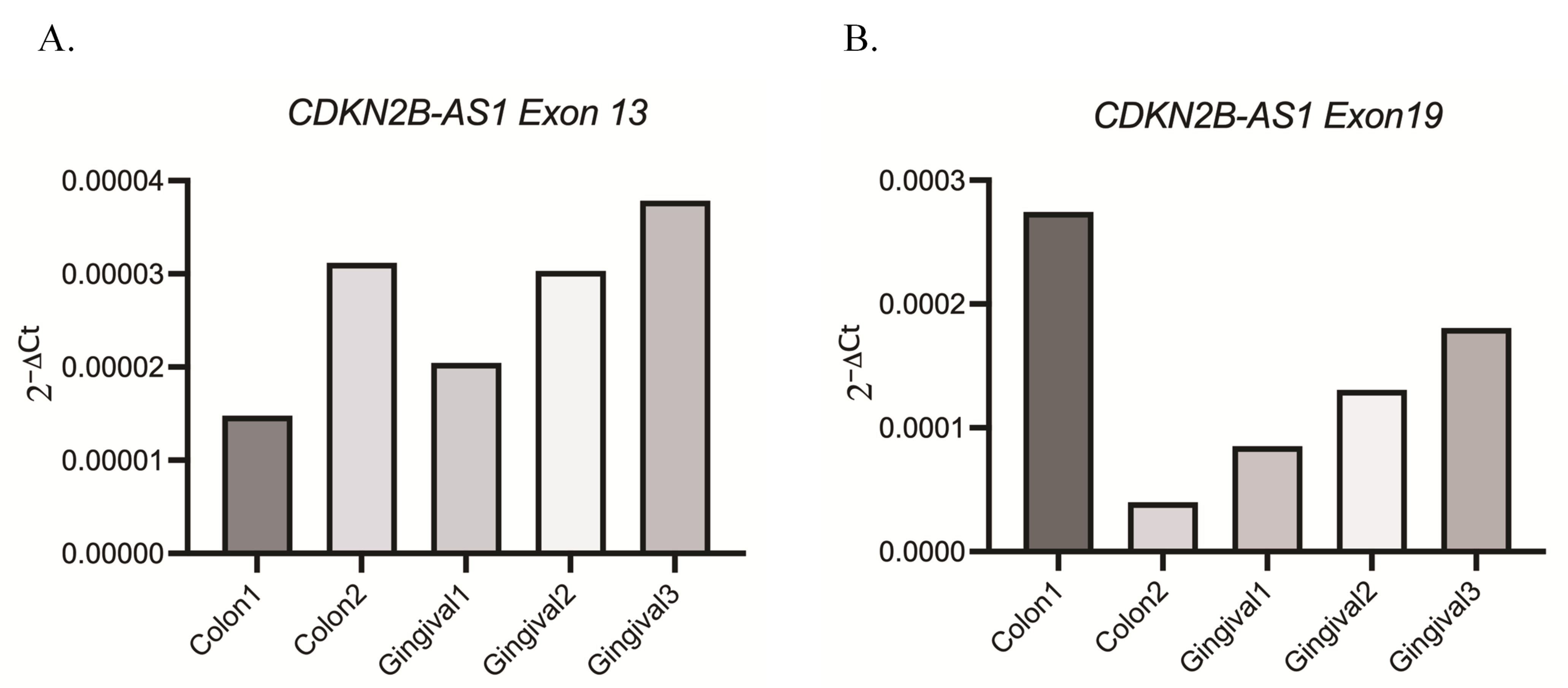


**Supplementary Fig. S1** The poly-adenylated transcripts of CDKN2B-AS1 (terminating with poly-A exon 13 and -19) show equal expression in healthy colon and gingiva (determined by qRT-PCR).

**Supplementary Fig. S2**  Linkage disequilibrium (LD, *r^2^* > 0.8) of the lead SNP rs1333049 with other SNPs


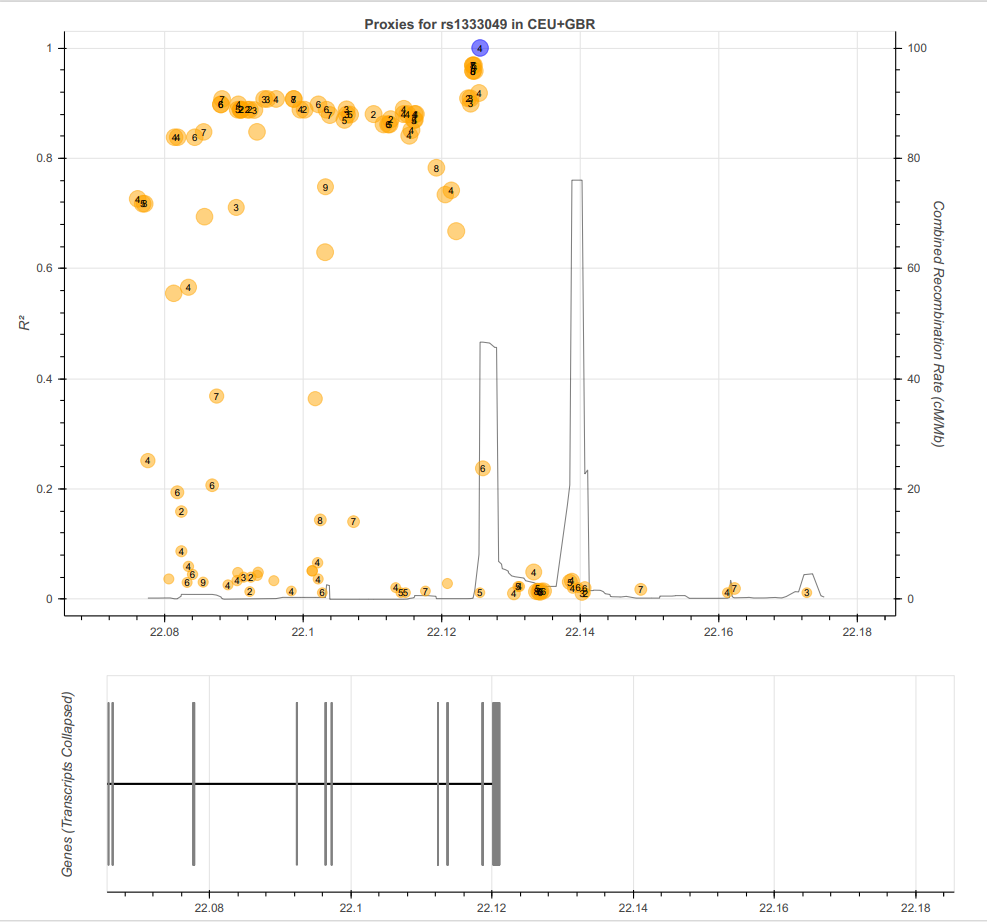


**Supplementary Fig. S3** Chromatin alignments of SNPs in linkage disequilibrium (LD, *r^2^* > 0.8) with the lead SNP rs1333049

**Supplementary Fig. S4**


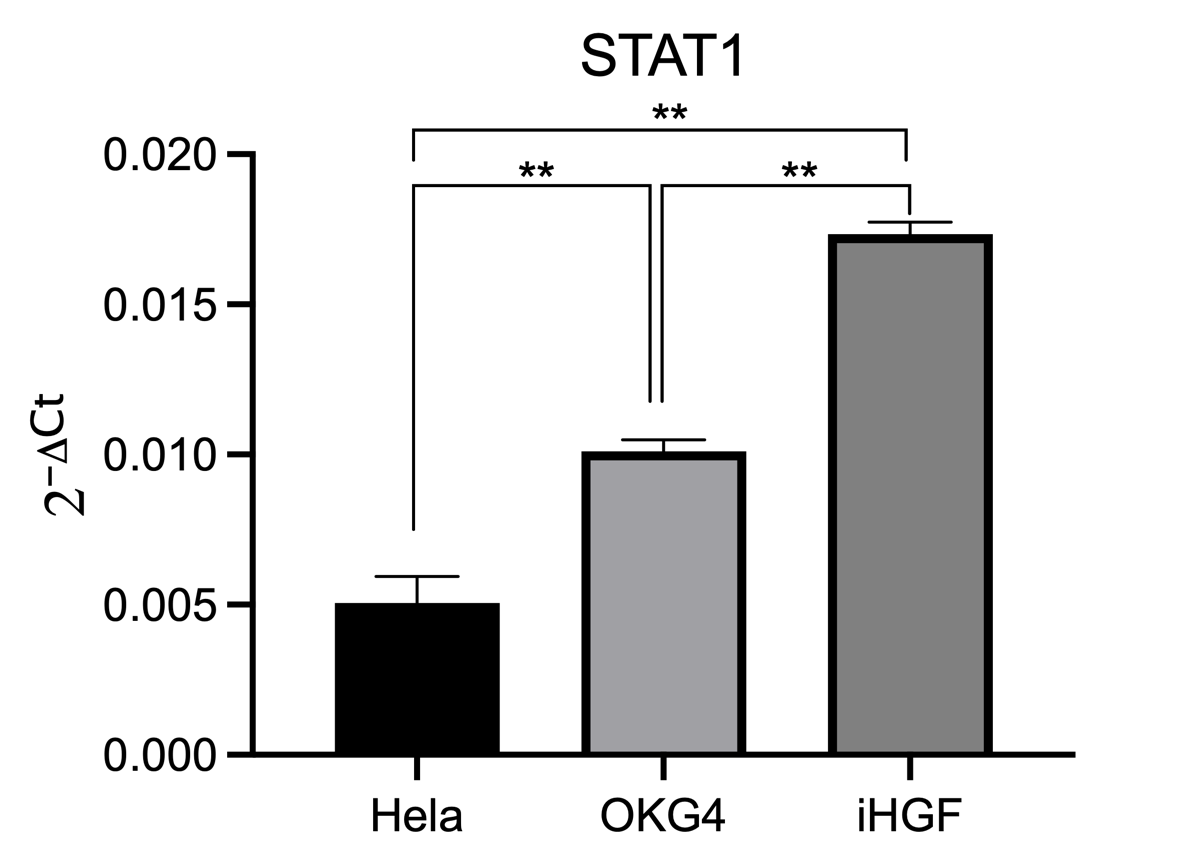


**Supplementary Fig. S4** qRT-PCR validation of *STAT1* gene expression in Hela cells, oral epithelial (OKG4) and gingival fibroblasts (iHGF) cell lines. The data revealed a significant upregulation of *STAT1* expression in iHGF cells compared to both Hela and OKG4 cells (*p < 0.05, **p<0.01).

| **Supplementary Table S3.** SNPs in linkage disequilibrium (LD, r2 > 0.8) with the lead SNP rs1333049 | | | | | | |
| --- | --- | --- | --- | --- | --- | --- |
| **RS_Number** | **chromosome** | **basepair position (hg19)** | **Alleles** | **MAF** | **Distance (bp) from GWAS lead SNP rs1333049** | **R2** |
| rs1333049 | chr9 | 22125503 | (G/C) | 0,4684 | NA | 1,0 |
| rs1333048 | chr9 | 22125347 | (A/C) | 0,4842 | -156 | 0,9183 |
| rs4977575 | chr9 | 22124744 | (C/G) | 0,4737 | -603 | 0,9583 |
| rs10757279 | chr9 | 22124630 | (A/G) | 0,4711 | -114 | 0,9686 |
| rs1333047 | chr9 | 22124504 | (A/T) | 0,4737 | -126 | 0,9583 |
| rs10757278 | chr9 | 22124477 | (A/G) | 0,4711 | -27 | 0,9686 |
| rs10811656 | chr9 | 22124472 | (C/T) | 0,4684 | -5 | 0,9582 |
| rs10757277 | chr9 | 22124450 | (A/G) | 0,4711 | -22 | 0,9686 |
| rs7857118 | chr9 | 22124140 | (A/T) | 0,4895 | -310 | 0,899 |
| rs1333046 | chr9 | 22124123 | (T/A) | 0,4868 | -17 | 0,9086 |
| rs10738610 | chr9 | 22123766 | (A/C) | 0,4868 | -357 | 0,9086 |
| rs1537376 | chr9 | 22116220 | (T/C) | 0,4947 | -7546 | 0,88 |
| rs1537375 | chr9 | 22116071 | (T/C) | 0,4921 | -149 | 0,8697 |
| rs1537374 | chr9 | 22116046 | (A/G) | 0,4947 | -25 | 0,88 |
| rs2383207 | chr9 | 22115959 | (A/G) | 0,4921 | -87 | 0,8697 |
| rs1004638 | chr9 | 22115589 | (A/T) | 0,4974 | -370 | 0,8511 |
| rs944797 | chr9 | 22115286 | (T/C) | 0,4947 | -303 | 0,8409 |
| rs2383206 | chr9 | 22115026 | (A/G) | 0,4947 | -260 | 0,88 |
| rs10738609 | chr9 | 22114495 | (A/G) | 0,4921 | -531 | 0,8895 |
| rs10733376 | chr9 | 22114469 | (G/C) | 0,4947 | -26 | 0,88 |
| rs10511701 | chr9 | 22112599 | (T/C) | 0,4974 | -1870 | 0,8707 |
| rs7341791 | chr9 | 22112427 | (A/G) | 0,5 | -172 | 0,8615 |
| rs7341786 | chr9 | 22112241 | (A/C) | 0,5 | -186 | 0,8615 |
| rs112917455 | chr9 | 22111584 | (-/ATTTG) | 0,5 | -657 | 0,8615 |
| rs1412834 | chr9 | 22110131 | (T/C) | 0,4947 | -1453 | 0,88 |
| rs1333043 | chr9 | 22106731 | (T/A) | 0,4895 | -3400 | 0,8791 |
| rs6475609 | chr9 | 22106271 | (A/G) | 0,4895 | -460 | 0,8791 |
| rs10757275 | chr9 | 22106225 | (G/A) | 0,4868 | -46 | 0,8886 |
| rs7859362 | chr9 | 22105927 | (T/C) | 0,4921 | -298 | 0,8697 |
| rs1333042 | chr9 | 22103813 | (A/G) | 0,4842 | -2114 | 0,8783 |
| rs1537373 | chr9 | 22103341 | (T/G) | 0,4816 | -472 | 0,8879 |
| rs7859727 | chr9 | 22102165 | (C/T) | 0,4789 | -1176 | 0,8976 |
| rs1556516 | chr9 | 22100176 | (G/C) | 0,4816 | -1989 | 0,8879 |
| rs1537371 | chr9 | 22099568 | (C/A) | 0,4816 | -608 | 0,8879 |
| rs2891168 | chr9 | 22098619 | (A/G) | 0,4763 | -949 | 0,9075 |
| rs4977574 | chr9 | 22098574 | (A/G) | 0,4763 | -45 | 0,9075 |
| rs10757274 | chr9 | 22096055 | (A/G) | 0,4763 | -2519 | 0,9075 |
| rs10738608 | chr9 | 22094796 | (A/C) | 0,4763 | -1259 | 0,9075 |
| rs4977757 | chr9 | 22094330 | (A/G) | 0,4763 | -466 | 0,9075 |
| rs144858577 | chr9 | 22093299 | (A/T) | 0,4711 | -1031 | 0,8478 |
| rs141014318 | chr9 | 22092924 | (A/G) | 0,4763 | -375 | 0,8875 |
| rs2210538 | chr9 | 22092257 | (G/A) | 0,4816 | -667 | 0,8879 |
| rs7866503 | chr9 | 22091924 | (G/T) | 0,4816 | -333 | 0,8879 |
| rs10811653 | chr9 | 22091069 | (C/T) | 0,4816 | -855 | 0,8879 |
| rs9644862 | chr9 | 22090936 | (T/G) | 0,4816 | -133 | 0,8879 |
| rs9644861 | chr9 | 22090935 | (C/T) | 0,4816 | -1 | 0,8879 |
| rs9644860 | chr9 | 22090603 | (C/T) | 0,4789 | -332 | 0,8976 |
| rs9644859 | chr9 | 22090521 | (G/A) | 0,4816 | -82 | 0,8879 |
| rs10757272 | chr9 | 22088260 | (C/T) | 0,4763 | -2261 | 0,9075 |
| rs10738607 | chr9 | 22088094 | (A/G) | 0,4789 | -166 | 0,8976 |
| rs10738606 | chr9 | 22088090 | (A/T) | 0,4789 | -4 | 0,8976 |
| rs1970112 | chr9 | 22085598 | (T/C) | 0,4658 | -2492 | 0,8477 |
| rs1537370 | chr9 | 22084310 | (C/T) | 0,4684 | -1288 | 0,8381 |
| rs6475606 | chr9 | 22081850 | (C/T) | 0,4684 | -2460 | 0,8381 |
| rs10116277 | chr9 | 22081397 | (G/T) | 0,4684 | NA | 0,8381 |

| **Appendix Table S4.** Differentially expressed genes after GapmeRs induced CDKN2B-AS1 knockdown(DEGs_up_log2FC>2) | | | | | | |
| --- | --- | --- | --- | --- | --- | --- |
| **ENSEMBL ID** | **gene symbol** | **log2FoldChange (lgc)** | **standard error (lfc)** | **stat** | **pvalue** | **padj** |
| ENSG00000249917 | LINC00536 | 7.192 | 1.129 | 6.367 | 0 | 0 |
| ENSG00000253153 |  | 6.899 | 1.155 | 5.974 | 0 | 0 |
| ENSG00000237977 |  | 6.698 | 1.175 | 5.702 | 0 | 0 |
| ENSG00000232892 | RGS5-AS1 | 6.507 | 1.13 | 5.758 | 0 | 0 |
| ENSG00000115607 | IL18RAP | 6.261 | 1.065 | 5.879 | 0 | 0 |
| ENSG00000259388 | RLIMP3 | 6.144 | 1.026 | 5.987 | 0 | 0 |
| ENSG00000235716 | KRT18P46 | 5.919 | 0.857 | 6.91 | 0 | 0 |
| ENSG00000164556 | FAM183BP | 5.917 | 0.991 | 5.971 | 0 | 0 |
| ENSG00000187527 | ATP13A5 | 5.878 | 1.045 | 5.623 | 0 | 0 |
| ENSG00000214626 | POLR3DP1 | 5.826 | 0.605 | 9.633 | 0 | 0 |
| ENSG00000261083 | LINC02516 | 5.802 | 1.007 | 5.763 | 0 | 0 |
| ENSG00000266988 | LOC112268408 | 5.793 | 1.106 | 5.238 | 0 | 0 |
| ENSG00000233833 | ETF1P3 | 5.788 | 1.138 | 5.085 | 0 | 0 |
| ENSG00000248565 | TECRP2 | 5.718 | 1.281 | 4.464 | 0 | 0.0001 |
| ENSG00000226908 | H2BP6 | 5.671 | 1.274 | 4.452 | 0 | 0.0001 |
| ENSG00000256812 | CAPNS2 | 5.645 | 0.532 | 10.608 | 0 | 0 |
| ENSG00000218189 |  | 5.604 | 1.148 | 4.883 | 0 | 0 |
| ENSG00000249464 | LINC01091 | 5.597 | 0.634 | 8.822 | 0 | 0 |
| ENSG00000213070 | HMGB3P6 | 5.554 | 1.279 | 4.343 | 0 | 0.0001 |
| ENSG00000115616 | SLC9A2 | 5.552 | 1.896 | 2.929 | 0.0034 | 0.0115 |
| ENSG00000237406 | NDUFA9P1 | 5.55 | 1.197 | 4.637 | 0 | 0 |
| ENSG00000259144 |  | 5.545 | 1.279 | 4.337 | 0 | 0.0001 |
| ENSG00000139330 | KERA | 5.497 | 1.113 | 4.937 | 0 | 0 |
| ENSG00000140932 | CMTM2 | 5.486 | 1.15 | 4.769 | 0 | 0 |
| ENSG00000250484 | PPIAP76 | 5.485 | 1.316 | 4.167 | 0 | 0.0002 |
| ENSG00000224478 |  | 5.483 | 0.878 | 6.248 | 0 | 0 |
| ENSG00000251526 | LINC02435 | 5.452 | 1.302 | 4.187 | 0 | 0.0002 |
| ENSG00000147697 | GSDMC | 5.441 | 1.329 | 4.092 | 0 | 0.0003 |
| ENSG00000226126 | PSMC1P12 | 5.436 | 1.312 | 4.142 | 0 | 0.0002 |
| ENSG00000272094 |  | 5.385 | 1.336 | 4.031 | 0.0001 | 0.0003 |
| ENSG00000236148 | RPL23AP37 | 5.373 | 0.863 | 6.227 | 0 | 0 |
| ENSG00000148942 | SLC5A12 | 5.302 | 1.008 | 5.259 | 0 | 0 |
| ENSG00000176009 | ASCL3 | 5.298 | 1.263 | 4.195 | 0 | 0.0002 |
| ENSG00000255331 |  | 5.284 | 1.011 | 5.228 | 0 | 0 |
| ENSG00000197085 | NPSR1-AS1 | 5.274 | 1.347 | 3.916 | 0.0001 | 0.0005 |
| ENSG00000257894 |  | 5.229 | 1.324 | 3.949 | 0.0001 | 0.0005 |
| ENSG00000172410 | INSL5 | 5.227 | 1.177 | 4.441 | 0 | 0.0001 |
| ENSG00000174567 | GOLT1A | 5.194 | 1.475 | 3.521 | 0.0004 | 0.002 |
| ENSG00000256288 | LINC02617 | 5.183 | 1.233 | 4.204 | 0 | 0.0002 |
| ENSG00000124103 | FAM209A | 5.18 | 1.012 | 5.119 | 0 | 0 |
| ENSG00000231079 |  | 5.172 | 1.166 | 4.436 | 0 | 0.0001 |
| ENSG00000278966 |  | 5.172 | 1.249 | 4.142 | 0 | 0.0002 |
| ENSG00000232327 | LOC100419739 | 5.14 | 1.214 | 4.233 | 0 | 0.0002 |
| ENSG00000185002 | RFX6 | 5.133 | 1.31 | 3.917 | 0.0001 | 0.0005 |
| ENSG00000213218 | CSH2 | 5.125 | 0.672 | 7.631 | 0 | 0 |
| ENSG00000225719 |  | 5.09 | 1.381 | 3.685 | 0.0002 | 0.0012 |
| ENSG00000251309 |  | 5.087 | 1.357 | 3.748 | 0.0002 | 0.0009 |
| ENSG00000136488 | CSH1 | 5.044 | 0.623 | 8.091 | 0 | 0 |
| ENSG00000124749 | COL21A1 | 5.041 | 0.7 | 7.2 | 0 | 0 |
| ENSG00000183654 | MARCHF11 | 5.026 | 1.169 | 4.298 | 0 | 0.0001 |
| ENSG00000267097 | SLC14A2-AS1 | 5.018 | 1.186 | 4.229 | 0 | 0.0002 |
| ENSG00000169550 | MUC15 | 5.006 | 1.4 | 3.574 | 0.0004 | 0.0017 |
| ENSG00000253547 | TUBAP7 | 4.999 | 1.594 | 3.137 | 0.0017 | 0.0064 |
| ENSG00000204086 | RPA4 | 4.993 | 1.232 | 4.052 | 0.0001 | 0.0003 |
| ENSG00000173452 | TMEM196 | 4.991 | 1.367 | 3.652 | 0.0003 | 0.0013 |
| ENSG00000267272 | LINC01140 | 4.979 | 1.38 | 3.609 | 0.0003 | 0.0015 |
| ENSG00000228489 | RPL21P50 | 4.97 | 1.411 | 3.521 | 0.0004 | 0.002 |
| ENSG00000231114 |  | 4.951 | 1.382 | 3.582 | 0.0003 | 0.0016 |
| ENSG00000259924 |  | 4.936 | 1.43 | 3.453 | 0.0006 | 0.0025 |
| ENSG00000272610 | MAGI1-IT1 | 4.927 | 1.22 | 4.038 | 0.0001 | 0.0003 |
| ENSG00000165443 | PHYHIPL | 4.917 | 1.394 | 3.526 | 0.0004 | 0.002 |
| ENSG00000249837 |  | 4.896 | 1.421 | 3.445 | 0.0006 | 0.0025 |
| ENSG00000248506 | TUBAP10 | 4.886 | 1.142 | 4.28 | 0 | 0.0001 |
| ENSG00000250727 |  | 4.845 | 0.983 | 4.929 | 0 | 0 |
| ENSG00000270591 |  | 4.822 | 1.198 | 4.024 | 0.0001 | 0.0003 |
| ENSG00000137766 | UNC13C | 4.816 | 1.806 | 2.667 | 0.0077 | 0.0227 |
| ENSG00000267354 |  | 4.808 | 1.275 | 3.772 | 0.0002 | 0.0009 |
| ENSG00000261208 |  | 4.79 | 0.967 | 4.956 | 0 | 0 |
| ENSG00000112246 | SIM1 | 4.788 | 0.71 | 6.74 | 0 | 0 |
| ENSG00000232587 | EEF1A1P3 | 4.779 | 1.026 | 4.658 | 0 | 0 |
| ENSG00000187753 | C9orf153 | 4.73 | 1.418 | 3.335 | 0.0009 | 0.0036 |
| ENSG00000186051 | TAL2 | 4.709 | 1.301 | 3.618 | 0.0003 | 0.0014 |
| ENSG00000101489 | CELF4 | 4.707 | 1.292 | 3.645 | 0.0003 | 0.0013 |
| ENSG00000263464 | PPIAL4C | 4.702 | 1.299 | 3.621 | 0.0003 | 0.0014 |
| ENSG00000244281 |  | 4.701 | 0.99 | 4.75 | 0 | 0 |
| ENSG00000258679 |  | 4.693 | 1.382 | 3.395 | 0.0007 | 0.003 |
| ENSG00000173702 | MUC13 | 4.69 | 0.445 | 10.546 | 0 | 0 |
| ENSG00000198670 | LPA | 4.68 | 0.761 | 6.15 | 0 | 0 |
| ENSG00000277186 |  | 4.672 | 1.078 | 4.334 | 0 | 0.0001 |
| ENSG00000163666 | HESX1 | 4.642 | 0.825 | 5.624 | 0 | 0 |
| ENSG00000277246 |  | 4.641 | 1.086 | 4.274 | 0 | 0.0001 |
| ENSG00000122188 | LAX1 | 4.603 | 1.252 | 3.675 | 0.0002 | 0.0012 |
| ENSG00000254997 | KRTAP5-9 | 4.6 | 0.97 | 4.741 | 0 | 0 |
| ENSG00000253991 |  | 4.6 | 1.035 | 4.446 | 0 | 0.0001 |
| ENSG00000214216 | IQCJ | 4.595 | 1.077 | 4.265 | 0 | 0.0001 |
| ENSG00000187999 |  | 4.589 | 1.276 | 3.596 | 0.0003 | 0.0016 |
| ENSG00000165863 | C10orf82 | 4.562 | 1.254 | 3.637 | 0.0003 | 0.0014 |
| ENSG00000174529 | TMEM81 | 4.556 | 0.486 | 9.383 | 0 | 0 |
| ENSG00000166268 | MYRFL | 4.523 | 0.719 | 6.294 | 0 | 0 |
| ENSG00000227877 | MRLN | 4.52 | 1.326 | 3.409 | 0.0007 | 0.0028 |
| ENSG00000187944 | C2orf66 | 4.52 | 1.335 | 3.387 | 0.0007 | 0.003 |
| ENSG00000204414 | CSHL1 | 4.518 | 0.5 | 9.044 | 0 | 0 |
| ENSG00000270429 | KNOP1P2 | 4.511 | 0.813 | 5.55 | 0 | 0 |
| ENSG00000185041 |  | 4.511 | 1.034 | 4.363 | 0 | 0.0001 |
| ENSG00000235595 |  | 4.501 | 1.091 | 4.125 | 0 | 0.0002 |
| ENSG00000127249 | ATP13A4 | 4.479 | 0.923 | 4.854 | 0 | 0 |
| ENSG00000197651 | CCER1 | 4.479 | 0.888 | 5.045 | 0 | 0 |
| ENSG00000225231 | LINC02470 | 4.479 | 1.464 | 3.059 | 0.0022 | 0.008 |
| ENSG00000224238 |  | 4.468 | 1.222 | 3.655 | 0.0003 | 0.0013 |
| ENSG00000255303 |  | 4.453 | 0.788 | 5.651 | 0 | 0 |
| ENSG00000217791 | ASS1P9 | 4.451 | 1.227 | 3.628 | 0.0003 | 0.0014 |
| ENSG00000258084 |  | 4.45 | 1.037 | 4.293 | 0 | 0.0001 |
| ENSG00000116882 | HAO2 | 4.439 | 1.479 | 3.002 | 0.0027 | 0.0094 |
| ENSG00000214203 | RPS4XP1 | 4.431 | 1.067 | 4.154 | 0 | 0.0002 |
| ENSG00000197273 | GUCA2A | 4.425 | 0.705 | 6.276 | 0 | 0 |
| ENSG00000263235 |  | 4.421 | 0.501 | 8.832 | 0 | 0 |
| ENSG00000116745 | RPE65 | 4.416 | 1.192 | 3.706 | 0.0002 | 0.0011 |
| ENSG00000213714 | FAM209B | 4.412 | 1.016 | 4.342 | 0 | 0.0001 |
| ENSG00000126838 | PZP | 4.41 | 1.122 | 3.931 | 0.0001 | 0.0005 |
| ENSG00000250492 | INTS6P1 | 4.374 | 1.456 | 3.005 | 0.0027 | 0.0093 |
| ENSG00000230876 | LINC00486 | 4.36 | 1.218 | 3.579 | 0.0003 | 0.0016 |
| ENSG00000171658 | NMRAL2P | 4.35 | 0.574 | 7.581 | 0 | 0 |
| ENSG00000232709 |  | 4.332 | 0.605 | 7.155 | 0 | 0 |
| ENSG00000267101 |  | 4.331 | 0.945 | 4.585 | 0 | 0 |
| ENSG00000110244 | APOA4 | 4.329 | 0.792 | 5.467 | 0 | 0 |
| ENSG00000230358 | SPDYE21 | 4.326 | 1.197 | 3.613 | 0.0003 | 0.0015 |
| ENSG00000203859 | HSD3B2 | 4.323 | 1.361 | 3.177 | 0.0015 | 0.0057 |
| ENSG00000244073 | RPS4XP6 | 4.315 | 1.053 | 4.096 | 0 | 0.0003 |
| ENSG00000165023 | DIRAS2 | 4.313 | 0.816 | 5.284 | 0 | 0 |
| ENSG00000225755 |  | 4.31 | 0.967 | 4.459 | 0 | 0.0001 |
| ENSG00000233766 |  | 4.307 | 0.654 | 6.581 | 0 | 0 |
| ENSG00000164764 | SBSPON | 4.299 | 1.044 | 4.119 | 0 | 0.0002 |
| ENSG00000181418 | DDN | 4.29 | 0.429 | 10.003 | 0 | 0 |
| ENSG00000167711 | SERPINF2 | 4.285 | 0.919 | 4.661 | 0 | 0 |
| ENSG00000136487 | GH2 | 4.277 | 0.571 | 7.484 | 0 | 0 |
| ENSG00000163217 | BMP10 | 4.259 | 1.071 | 3.977 | 0.0001 | 0.0004 |
| ENSG00000229808 |  | 4.239 | 0.542 | 7.826 | 0 | 0 |
| ENSG00000171989 | LDHAL6B | 4.224 | 0.842 | 5.018 | 0 | 0 |
| ENSG00000188373 | C10orf99 | 4.211 | 1.008 | 4.176 | 0 | 0.0002 |
| ENSG00000259188 |  | 4.206 | 0.985 | 4.272 | 0 | 0.0001 |
| ENSG00000237415 |  | 4.204 | 1.045 | 4.025 | 0.0001 | 0.0003 |
| ENSG00000233597 | RPL19P6 | 4.203 | 1.061 | 3.96 | 0.0001 | 0.0004 |
| ENSG00000168743 | NPNT | 4.183 | 1.412 | 2.962 | 0.0031 | 0.0105 |
| ENSG00000226655 |  | 4.181 | 1.34 | 3.121 | 0.0018 | 0.0067 |
| ENSG00000269404 | SPIB | 4.176 | 0.862 | 4.845 | 0 | 0 |
| ENSG00000248196 | LOC100419572 | 4.17 | 1.215 | 3.431 | 0.0006 | 0.0026 |
| ENSG00000172548 | NIPAL4 | 4.169 | 1.2 | 3.474 | 0.0005 | 0.0023 |
| ENSG00000227056 | RPL6P2 | 4.158 | 1.166 | 3.566 | 0.0004 | 0.0017 |
| ENSG00000266818 |  | 4.157 | 1.205 | 3.449 | 0.0006 | 0.0025 |
| ENSG00000264379 |  | 4.154 | 1.206 | 3.446 | 0.0006 | 0.0025 |
| ENSG00000116883 |  | 4.141 | 0.625 | 6.628 | 0 | 0 |
| ENSG00000180869 | LINC01555 | 4.129 | 1.117 | 3.696 | 0.0002 | 0.0011 |
| ENSG00000257777 |  | 4.126 | 1.361 | 3.032 | 0.0024 | 0.0086 |
| ENSG00000184608 | FAM167A-AS1 | 4.115 | 1.246 | 3.303 | 0.001 | 0.0039 |
| ENSG00000179131 | RPL31P38 | 4.114 | 1.053 | 3.906 | 0.0001 | 0.0005 |
| ENSG00000177291 | GJD4 | 4.114 | 1.152 | 3.572 | 0.0004 | 0.0017 |
| ENSG00000234199 | LINC01191 | 4.113 | 0.661 | 6.221 | 0 | 0 |
| ENSG00000236154 |  | 4.109 | 1.329 | 3.092 | 0.002 | 0.0073 |
| ENSG00000143839 | REN | 4.102 | 0.814 | 5.04 | 0 | 0 |
| ENSG00000144290 | SLC4A10 | 4.102 | 0.915 | 4.485 | 0 | 0.0001 |
| ENSG00000228196 |  | 4.09 | 0.97 | 4.218 | 0 | 0.0002 |
| ENSG00000233780 | LINC00367 | 4.087 | 0.436 | 9.366 | 0 | 0 |
| ENSG00000122194 | PLG | 4.08 | 0.768 | 5.311 | 0 | 0 |
| ENSG00000232172 | RPL19P15 | 4.072 | 0.494 | 8.238 | 0 | 0 |
| ENSG00000188662 |  | 4.056 | 0.497 | 8.153 | 0 | 0 |
| ENSG00000147655 | RSPO2 | 4.048 | 0.405 | 9.995 | 0 | 0 |
| ENSG00000226968 |  | 4.047 | 1.289 | 3.14 | 0.0017 | 0.0063 |
| ENSG00000257611 |  | 4.046 | 1.235 | 3.277 | 0.001 | 0.0043 |
| ENSG00000253672 |  | 4.045 | 1.336 | 3.029 | 0.0025 | 0.0087 |
| ENSG00000253894 | LOC102724612 | 4.043 | 1.12 | 3.61 | 0.0003 | 0.0015 |
| ENSG00000113492 | AGXT2 | 4.042 | 0.962 | 4.202 | 0 | 0.0002 |
| ENSG00000074803 | SLC12A1 | 4.039 | 0.789 | 5.121 | 0 | 0 |
| ENSG00000164326 | CARTPT | 4.036 | 1.073 | 3.762 | 0.0002 | 0.0009 |
| ENSG00000197410 | DCHS2 | 4.032 | 1.379 | 2.924 | 0.0035 | 0.0116 |
| ENSG00000130413 | STK33 | 4.028 | 0.773 | 5.211 | 0 | 0 |
| ENSG00000231194 |  | 4.021 | 0.713 | 5.643 | 0 | 0 |
| ENSG00000115263 | GCG | 4.018 | 0.525 | 7.654 | 0 | 0 |
| ENSG00000166159 | LRTM2 | 4.018 | 0.585 | 6.871 | 0 | 0 |
| ENSG00000259384 | GH1 | 4.017 | 0.466 | 8.626 | 0 | 0 |
| ENSG00000251215 |  | 4.014 | 1.377 | 2.915 | 0.0036 | 0.0119 |
| ENSG00000150048 | CLEC1A | 3.999 | 1.15 | 3.478 | 0.0005 | 0.0023 |
| ENSG00000235413 |  | 3.994 | 1.023 | 3.903 | 0.0001 | 0.0005 |
| ENSG00000233008 | LOC101927560 | 3.99 | 1.07 | 3.729 | 0.0002 | 0.001 |
| ENSG00000129538 | RNASE1 | 3.986 | 0.72 | 5.535 | 0 | 0 |
| ENSG00000203435 |  | 3.984 | 0.945 | 4.216 | 0 | 0.0002 |
| ENSG00000270469 |  | 3.984 | 1.199 | 3.324 | 0.0009 | 0.0037 |
| ENSG00000249307 | LINC01088 | 3.981 | 1.019 | 3.908 | 0.0001 | 0.0005 |
| ENSG00000241280 |  | 3.98 | 0.879 | 4.526 | 0 | 0 |
| ENSG00000229817 | LOC645266 | 3.96 | 0.571 | 6.939 | 0 | 0 |
| ENSG00000230461 | PROX1-AS1 | 3.959 | 1.268 | 3.121 | 0.0018 | 0.0067 |
| ENSG00000162676 | GFI1 | 3.949 | 0.829 | 4.764 | 0 | 0 |
| ENSG00000255693 | LINC02389 | 3.944 | 1.047 | 3.769 | 0.0002 | 0.0009 |
| ENSG00000260166 |  | 3.941 | 1.107 | 3.562 | 0.0004 | 0.0017 |
| ENSG00000173212 | MAB21L3 | 3.93 | 0.558 | 7.045 | 0 | 0 |
| ENSG00000168509 | HJV | 3.921 | 0.802 | 4.891 | 0 | 0 |
| ENSG00000281404 | LINC01176 | 3.918 | 1.239 | 3.161 | 0.0016 | 0.006 |
| ENSG00000174680 |  | 3.917 | 1.21 | 3.238 | 0.0012 | 0.0048 |
| ENSG00000229314 | ORM1 | 3.914 | 1.056 | 3.707 | 0.0002 | 0.0011 |
| ENSG00000248245 |  | 3.903 | 0.931 | 4.19 | 0 | 0.0002 |
| ENSG00000239873 |  | 3.903 | 1.154 | 3.381 | 0.0007 | 0.0031 |
| ENSG00000219163 |  | 3.901 | 1.256 | 3.106 | 0.0019 | 0.007 |
| ENSG00000188991 | SLC15A5 | 3.899 | 1.19 | 3.277 | 0.001 | 0.0043 |
| ENSG00000229140 | CCDC26 | 3.896 | 1.201 | 3.243 | 0.0012 | 0.0047 |
| ENSG00000198028 | ZNF560 | 3.895 | 0.392 | 9.932 | 0 | 0 |
| ENSG00000111783 | RFX4 | 3.894 | 0.729 | 5.338 | 0 | 0 |
| ENSG00000273056 |  | 3.874 | 1.095 | 3.536 | 0.0004 | 0.0019 |
| ENSG00000259928 |  | 3.869 | 0.729 | 5.304 | 0 | 0 |
| ENSG00000226542 |  | 3.86 | 0.577 | 6.689 | 0 | 0 |
| ENSG00000129749 | CHRNA10 | 3.855 | 0.325 | 11.866 | 0 | 0 |
| ENSG00000257752 | LOC100420011 | 3.849 | 0.958 | 4.017 | 0.0001 | 0.0004 |
| ENSG00000248180 |  | 3.844 | 0.917 | 4.193 | 0 | 0.0002 |
| ENSG00000227694 | RPL23AP74 | 3.839 | 1.195 | 3.214 | 0.0013 | 0.0051 |
| ENSG00000146477 | SLC22A3 | 3.837 | 0.631 | 6.076 | 0 | 0 |
| ENSG00000230948 | AURKBP1 | 3.83 | 0.63 | 6.079 | 0 | 0 |
| ENSG00000103710 | RASL12 | 3.826 | 0.611 | 6.262 | 0 | 0 |
| ENSG00000223414 |  | 3.823 | 0.863 | 4.429 | 0 | 0.0001 |
| ENSG00000186452 | TMPRSS12 | 3.81 | 1.042 | 3.655 | 0.0003 | 0.0013 |
| ENSG00000170044 | ZPLD1 | 3.805 | 0.557 | 6.828 | 0 | 0 |
| ENSG00000256464 |  | 3.804 | 0.989 | 3.844 | 0.0001 | 0.0007 |
| ENSG00000138315 | OIT3 | 3.794 | 0.716 | 5.3 | 0 | 0 |
| ENSG00000176268 |  | 3.788 | 1.264 | 2.996 | 0.0027 | 0.0095 |
| ENSG00000259556 |  | 3.785 | 0.63 | 6.005 | 0 | 0 |
| ENSG00000189431 | RASSF10 | 3.779 | 0.85 | 4.446 | 0 | 0.0001 |
| ENSG00000280543 | ASAP1-IT2 | 3.771 | 1.006 | 3.75 | 0.0002 | 0.0009 |
| ENSG00000099282 | TSPAN15 | 3.765 | 0.671 | 5.612 | 0 | 0 |
| ENSG00000223575 |  | 3.759 | 1.318 | 2.852 | 0.0043 | 0.0141 |
| ENSG00000255311 |  | 3.752 | 1.204 | 3.117 | 0.0018 | 0.0068 |
| ENSG00000142700 | DMRTA2 | 3.75 | 0.955 | 3.925 | 0.0001 | 0.0005 |
| ENSG00000171115 | GIMAP8 | 3.746 | 0.802 | 4.67 | 0 | 0 |
| ENSG00000235683 |  | 3.743 | 0.986 | 3.795 | 0.0001 | 0.0008 |
| ENSG00000256155 |  | 3.742 | 1.247 | 3.001 | 0.0027 | 0.0094 |
| ENSG00000123843 | C4BPB | 3.737 | 1.089 | 3.432 | 0.0006 | 0.0026 |
| ENSG00000144847 | IGSF11 | 3.737 | 1.179 | 3.171 | 0.0015 | 0.0058 |
| ENSG00000227695 | DNMBP-AS1 | 3.722 | 0.51 | 7.305 | 0 | 0 |
| ENSG00000223963 | THAP12P8 | 3.721 | 1.102 | 3.377 | 0.0007 | 0.0031 |
| ENSG00000227440 |  | 3.717 | 0.66 | 5.633 | 0 | 0 |
| ENSG00000168314 | MOBP | 3.716 | 0.642 | 5.791 | 0 | 0 |
| ENSG00000100336 | APOL4 | 3.713 | 0.47 | 7.902 | 0 | 0 |
| ENSG00000204740 | MALRD1 | 3.706 | 0.624 | 5.935 | 0 | 0 |
| ENSG00000270852 |  | 3.705 | 0.802 | 4.622 | 0 | 0 |
| ENSG00000124678 | TCP11 | 3.704 | 1.358 | 2.727 | 0.0064 | 0.0196 |
| ENSG00000273416 |  | 3.703 | 0.876 | 4.227 | 0 | 0.0002 |
| ENSG00000105143 | SLC1A6 | 3.7 | 0.782 | 4.73 | 0 | 0 |
| ENSG00000141505 | ASGR1 | 3.696 | 0.394 | 9.38 | 0 | 0 |
| ENSG00000234352 | LOC349160 | 3.694 | 0.834 | 4.429 | 0 | 0.0001 |
| ENSG00000140835 | CHST4 | 3.691 | 1.295 | 2.849 | 0.0044 | 0.0142 |
| ENSG00000180113 | TDRD6 | 3.671 | 1.045 | 3.513 | 0.0004 | 0.002 |
| ENSG00000182050 | MGAT4C | 3.66 | 1.182 | 3.097 | 0.002 | 0.0072 |
| ENSG00000134207 | SYT6 | 3.659 | 1.113 | 3.288 | 0.001 | 0.0041 |
| ENSG00000101638 | ST8SIA5 | 3.655 | 0.723 | 5.054 | 0 | 0 |
| ENSG00000223718 | RPL15P11 | 3.654 | 0.854 | 4.278 | 0 | 0.0001 |
| ENSG00000237133 |  | 3.654 | 1.145 | 3.19 | 0.0014 | 0.0055 |
| ENSG00000155307 | SAMSN1 | 3.653 | 0.792 | 4.614 | 0 | 0 |
| ENSG00000283554 | LINC02341 | 3.653 | 1.282 | 2.848 | 0.0044 | 0.0143 |
| ENSG00000066813 | ACSM2B | 3.652 | 1.116 | 3.273 | 0.0011 | 0.0043 |
| ENSG00000216516 |  | 3.651 | 1.002 | 3.646 | 0.0003 | 0.0013 |
| ENSG00000274654 |  | 3.646 | 0.58 | 6.283 | 0 | 0 |
| ENSG00000100170 | SLC5A1 | 3.644 | 1.065 | 3.421 | 0.0006 | 0.0027 |
| ENSG00000152592 | DMP1 | 3.643 | 0.824 | 4.42 | 0 | 0.0001 |
| ENSG00000242220 | TCP10L | 3.638 | 0.996 | 3.651 | 0.0003 | 0.0013 |
| ENSG00000249593 |  | 3.638 | 1.359 | 2.678 | 0.0074 | 0.0221 |
| ENSG00000119457 | SLC46A2 | 3.637 | 0.945 | 3.848 | 0.0001 | 0.0007 |
| ENSG00000116254 | CHD5 | 3.634 | 0.362 | 10.042 | 0 | 0 |
| ENSG00000118997 | DNAH7 | 3.626 | 0.332 | 10.907 | 0 | 0 |
| ENSG00000187498 | COL4A1 | 3.625 | 0.333 | 10.896 | 0 | 0 |
| ENSG00000277831 |  | 3.619 | 0.732 | 4.945 | 0 | 0 |
| ENSG00000270981 | LOC100420048 | 3.616 | 1.352 | 2.674 | 0.0075 | 0.0223 |
| ENSG00000188001 | TPRG1 | 3.609 | 1.013 | 3.563 | 0.0004 | 0.0017 |
| ENSG00000265933 | LINC00668 | 3.605 | 0.851 | 4.235 | 0 | 0.0002 |
| ENSG00000165805 | C12orf50 | 3.596 | 0.612 | 5.873 | 0 | 0 |
| ENSG00000254272 |  | 3.594 | 1.304 | 2.756 | 0.0059 | 0.0182 |
| ENSG00000075461 | CACNG4 | 3.593 | 0.802 | 4.48 | 0 | 0.0001 |
| ENSG00000257225 |  | 3.591 | 0.613 | 5.855 | 0 | 0 |
| ENSG00000111834 | RSPH4A | 3.59 | 0.83 | 4.322 | 0 | 0.0001 |
| ENSG00000175646 | PRM1 | 3.589 | 1.193 | 3.008 | 0.0026 | 0.0092 |
| ENSG00000095596 | CYP26A1 | 3.585 | 1.022 | 3.507 | 0.0005 | 0.0021 |
| ENSG00000180532 | ZSCAN4 | 3.583 | 0.695 | 5.158 | 0 | 0 |
| ENSG00000214629 | RPSAP6 | 3.577 | 0.778 | 4.598 | 0 | 0 |
| ENSG00000101349 | PAK5 | 3.569 | 1.122 | 3.181 | 0.0015 | 0.0057 |
| ENSG00000144407 | PTH2R | 3.566 | 1.276 | 2.795 | 0.0052 | 0.0164 |
| ENSG00000148156 | ACTL7B | 3.558 | 0.471 | 7.561 | 0 | 0 |
| ENSG00000166689 | PLEKHA7 | 3.549 | 0.402 | 8.828 | 0 | 0 |
| ENSG00000129167 | TPH1 | 3.539 | 0.87 | 4.069 | 0 | 0.0003 |
| ENSG00000272636 | DOC2B | 3.536 | 0.563 | 6.286 | 0 | 0 |
| ENSG00000249360 |  | 3.523 | 0.625 | 5.639 | 0 | 0 |
| ENSG00000231784 | DBIL5P | 3.521 | 0.451 | 7.802 | 0 | 0 |
| ENSG00000234785 | EEF1GP5 | 3.52 | 0.735 | 4.791 | 0 | 0 |
| ENSG00000131482 | G6PC | 3.512 | 1.11 | 3.163 | 0.0016 | 0.0059 |
| ENSG00000186369 |  | 3.511 | 0.391 | 8.988 | 0 | 0 |
| ENSG00000250519 |  | 3.507 | 0.878 | 3.993 | 0.0001 | 0.0004 |
| ENSG00000272434 |  | 3.507 | 0.888 | 3.949 | 0.0001 | 0.0005 |
| ENSG00000255075 |  | 3.503 | 1.038 | 3.376 | 0.0007 | 0.0031 |
| ENSG00000255150 | EID3 | 3.501 | 0.327 | 10.703 | 0 | 0 |
| ENSG00000171133 | OR2K2 | 3.501 | 1.276 | 2.743 | 0.0061 | 0.0188 |
| ENSG00000182747 | SLC35D3 | 3.5 | 0.766 | 4.572 | 0 | 0 |
| ENSG00000253697 |  | 3.495 | 1.381 | 2.531 | 0.0114 | 0.0314 |
| ENSG00000239917 |  | 3.493 | 0.784 | 4.456 | 0 | 0.0001 |
| ENSG00000141437 | SLC25A52 | 3.481 | 1.051 | 3.313 | 0.0009 | 0.0038 |
| ENSG00000238172 | RPS2P35 | 3.47 | 0.74 | 4.688 | 0 | 0 |
| ENSG00000243440 |  | 3.463 | 1.106 | 3.132 | 0.0017 | 0.0065 |
| ENSG00000262488 |  | 3.463 | 1.219 | 2.841 | 0.0045 | 0.0145 |
| ENSG00000163606 | CD200R1 | 3.462 | 1.295 | 2.673 | 0.0075 | 0.0223 |
| ENSG00000232716 |  | 3.459 | 0.237 | 14.604 | 0 | 0 |
| ENSG00000237417 |  | 3.459 | 0.57 | 6.071 | 0 | 0 |
| ENSG00000153294 | ADGRF4 | 3.454 | 0.638 | 5.412 | 0 | 0 |
| ENSG00000149516 | MS4A3 | 3.452 | 1.117 | 3.09 | 0.002 | 0.0073 |
| ENSG00000260302 |  | 3.451 | 0.907 | 3.804 | 0.0001 | 0.0008 |
| ENSG00000125869 | LAMP5 | 3.45 | 0.774 | 4.459 | 0 | 0.0001 |
| ENSG00000143921 | ABCG8 | 3.449 | 0.475 | 7.264 | 0 | 0 |
| ENSG00000259158 | ADAM20P1 | 3.448 | 0.457 | 7.54 | 0 | 0 |
| ENSG00000259459 |  | 3.447 | 1.118 | 3.084 | 0.002 | 0.0074 |
| ENSG00000283294 |  | 3.446 | 1.009 | 3.415 | 0.0006 | 0.0028 |
| ENSG00000261239 |  | 3.439 | 0.282 | 12.203 | 0 | 0 |
| ENSG00000196115 | ADAM5 | 3.436 | 0.774 | 4.441 | 0 | 0.0001 |
| ENSG00000113600 | C9 | 3.434 | 0.531 | 6.465 | 0 | 0 |
| ENSG00000164756 | SLC30A8 | 3.434 | 0.869 | 3.952 | 0.0001 | 0.0005 |
| ENSG00000198300 | PEG3 | 3.427 | 0.776 | 4.414 | 0 | 0.0001 |
| ENSG00000278737 |  | 3.426 | 0.878 | 3.903 | 0.0001 | 0.0005 |
| ENSG00000177710 | SLC35G5 | 3.424 | 0.693 | 4.938 | 0 | 0 |
| ENSG00000176857 |  | 3.423 | 1.319 | 2.596 | 0.0094 | 0.0269 |
| ENSG00000111490 | TBC1D30 | 3.422 | 0.646 | 5.3 | 0 | 0 |
| ENSG00000240128 | KRT18P43 | 3.421 | 1.126 | 3.037 | 0.0024 | 0.0085 |
| ENSG00000258220 | LINC02424 | 3.417 | 1.339 | 2.552 | 0.0107 | 0.0298 |
| ENSG00000232884 |  | 3.416 | 1.043 | 3.276 | 0.0011 | 0.0043 |
| ENSG00000234699 |  | 3.41 | 0.772 | 4.419 | 0 | 0.0001 |
| ENSG00000186150 | UBL4B | 3.407 | 0.942 | 3.616 | 0.0003 | 0.0015 |
| ENSG00000115602 | IL1RL1 | 3.406 | 0.437 | 7.803 | 0 | 0 |
| ENSG00000253892 |  | 3.403 | 0.765 | 4.45 | 0 | 0.0001 |
| ENSG00000258907 | PSMB3P1 | 3.403 | 0.835 | 4.073 | 0 | 0.0003 |
| ENSG00000261456 | TUBB8 | 3.402 | 0.932 | 3.651 | 0.0003 | 0.0013 |
| ENSG00000275645 |  | 3.391 | 0.867 | 3.909 | 0.0001 | 0.0005 |
| ENSG00000282989 |  | 3.39 | 0.677 | 5.01 | 0 | 0 |
| ENSG00000267153 |  | 3.39 | 1.022 | 3.318 | 0.0009 | 0.0038 |
| ENSG00000282390 |  | 3.39 | 1.024 | 3.31 | 0.0009 | 0.0039 |
| ENSG00000236712 |  | 3.386 | 1.148 | 2.949 | 0.0032 | 0.0109 |
| ENSG00000215086 |  | 3.385 | 1.06 | 3.192 | 0.0014 | 0.0055 |
| ENSG00000118514 | ALDH8A1 | 3.371 | 0.535 | 6.305 | 0 | 0 |
| ENSG00000272109 |  | 3.371 | 0.881 | 3.825 | 0.0001 | 0.0007 |
| ENSG00000276662 | DDX20P1 | 3.368 | 0.961 | 3.505 | 0.0005 | 0.0021 |
| ENSG00000266127 | ZNF415P1 | 3.367 | 1.334 | 2.525 | 0.0116 | 0.0318 |
| ENSG00000242198 |  | 3.358 | 0.834 | 4.027 | 0.0001 | 0.0003 |
| ENSG00000255220 |  | 3.357 | 0.865 | 3.88 | 0.0001 | 0.0006 |
| ENSG00000007312 | CD79B | 3.354 | 0.454 | 7.381 | 0 | 0 |
| ENSG00000270011 | ZNF559-ZNF177 | 3.344 | 1 | 3.343 | 0.0008 | 0.0035 |
| ENSG00000127364 | TAS2R4 | 3.339 | 0.79 | 4.229 | 0 | 0.0002 |
| ENSG00000180245 | RRH | 3.339 | 0.981 | 3.405 | 0.0007 | 0.0029 |
| ENSG00000140015 | KCNH5 | 3.338 | 0.821 | 4.067 | 0 | 0.0003 |
| ENSG00000119915 | ELOVL3 | 3.337 | 0.321 | 10.406 | 0 | 0 |
| ENSG00000230523 | LINC01756 | 3.337 | 1.18 | 2.827 | 0.0047 | 0.0151 |
| ENSG00000149564 | ESAM | 3.336 | 0.449 | 7.422 | 0 | 0 |
| ENSG00000253564 |  | 3.333 | 0.745 | 4.475 | 0 | 0.0001 |
| ENSG00000177025 | C19orf18 | 3.329 | 0.907 | 3.672 | 0.0002 | 0.0012 |
| ENSG00000236814 |  | 3.327 | 0.4 | 8.328 | 0 | 0 |
| ENSG00000213071 | LPAL2 | 3.318 | 0.532 | 6.233 | 0 | 0 |
| ENSG00000273038 |  | 3.309 | 0.283 | 11.677 | 0 | 0 |
| ENSG00000274227 |  | 3.307 | 1.046 | 3.161 | 0.0016 | 0.006 |
| ENSG00000129151 | BBOX1 | 3.301 | 1.054 | 3.132 | 0.0017 | 0.0065 |
| ENSG00000198624 | CCDC69 | 3.3 | 0.507 | 6.507 | 0 | 0 |
| ENSG00000254275 |  | 3.3 | 0.659 | 5.004 | 0 | 0 |
| ENSG00000277744 |  | 3.298 | 1.145 | 2.879 | 0.004 | 0.0131 |
| ENSG00000267462 |  | 3.297 | 0.838 | 3.934 | 0.0001 | 0.0005 |
| ENSG00000260277 |  | 3.297 | 0.967 | 3.409 | 0.0007 | 0.0028 |
| ENSG00000273365 |  | 3.295 | 1.128 | 2.921 | 0.0035 | 0.0117 |
| ENSG00000109943 | CRTAM | 3.294 | 0.501 | 6.58 | 0 | 0 |
| ENSG00000196946 | ZNF705A | 3.294 | 0.663 | 4.967 | 0 | 0 |
| ENSG00000049247 | UTS2 | 3.293 | 0.903 | 3.648 | 0.0003 | 0.0013 |
| ENSG00000230601 | TEX48 | 3.293 | 1.132 | 2.91 | 0.0036 | 0.0121 |
| ENSG00000125895 | TMEM74B | 3.292 | 1.085 | 3.034 | 0.0024 | 0.0086 |
| ENSG00000187054 | TMPRSS11A | 3.29 | 1.077 | 3.053 | 0.0023 | 0.0081 |
| ENSG00000236212 |  | 3.286 | 1.386 | 2.371 | 0.0178 | 0.0453 |
| ENSG00000175749 | EIF3KP1 | 3.282 | 0.68 | 4.825 | 0 | 0 |
| ENSG00000213862 | RPL7AP62 | 3.282 | 1.1 | 2.982 | 0.0029 | 0.0099 |
| ENSG00000256358 | RPL31P57 | 3.271 | 0.556 | 5.883 | 0 | 0 |
| ENSG00000124019 | FAM124B | 3.268 | 0.549 | 5.948 | 0 | 0 |
| ENSG00000255004 |  | 3.266 | 0.805 | 4.058 | 0 | 0.0003 |
| ENSG00000166569 | CPLX4 | 3.263 | 0.706 | 4.619 | 0 | 0 |
| ENSG00000218632 | RPL7P28 | 3.259 | 0.805 | 4.046 | 0.0001 | 0.0003 |
| ENSG00000198515 | CNGA1 | 3.258 | 0.621 | 5.245 | 0 | 0 |
| ENSG00000225450 |  | 3.256 | 1.136 | 2.867 | 0.0042 | 0.0136 |
| ENSG00000048540 | LMO3 | 3.252 | 1.051 | 3.093 | 0.002 | 0.0073 |
| ENSG00000227331 | RPL7AP22 | 3.251 | 0.932 | 3.49 | 0.0005 | 0.0022 |
| ENSG00000157856 | DRC1 | 3.247 | 0.669 | 4.85 | 0 | 0 |
| ENSG00000232896 |  | 3.24 | 0.858 | 3.777 | 0.0002 | 0.0008 |
| ENSG00000240567 | LINC02067 | 3.24 | 1.359 | 2.384 | 0.0171 | 0.044 |
| ENSG00000177842 | ZNF620 | 3.236 | 0.369 | 8.776 | 0 | 0 |
| ENSG00000260423 | LINC02367 | 3.234 | 0.995 | 3.252 | 0.0011 | 0.0046 |
| ENSG00000133134 | BEX2 | 3.231 | 0.536 | 6.024 | 0 | 0 |
| ENSG00000153820 | SPHKAP | 3.224 | 0.876 | 3.68 | 0.0002 | 0.0012 |
| ENSG00000251203 |  | 3.224 | 0.887 | 3.634 | 0.0003 | 0.0014 |
| ENSG00000235275 | KRT18P16 | 3.22 | 0.717 | 4.493 | 0 | 0.0001 |
| ENSG00000265787 | CYP4F35P | 3.219 | 0.94 | 3.425 | 0.0006 | 0.0027 |
| ENSG00000216990 | HSPD1P10 | 3.219 | 0.953 | 3.377 | 0.0007 | 0.0031 |
| ENSG00000228600 |  | 3.218 | 1.163 | 2.767 | 0.0057 | 0.0177 |
| ENSG00000164393 | ADGRF2 | 3.217 | 0.663 | 4.85 | 0 | 0 |
| ENSG00000127366 | TAS2R5 | 3.216 | 0.789 | 4.077 | 0 | 0.0003 |
| ENSG00000078549 | ADCYAP1R1 | 3.216 | 0.972 | 3.307 | 0.0009 | 0.0039 |
| ENSG00000250247 |  | 3.21 | 1.042 | 3.081 | 0.0021 | 0.0075 |
| ENSG00000253556 | MTCO1P4 | 3.209 | 0.897 | 3.579 | 0.0003 | 0.0016 |
| ENSG00000182389 | CACNB4 | 3.207 | 0.55 | 5.833 | 0 | 0 |
| ENSG00000274979 |  | 3.207 | 0.808 | 3.967 | 0.0001 | 0.0004 |
| ENSG00000250026 | TMPRSS11BNL | 3.207 | 0.81 | 3.96 | 0.0001 | 0.0004 |
| ENSG00000168158 | OR2C1 | 3.207 | 1.059 | 3.028 | 0.0025 | 0.0087 |
| ENSG00000274776 |  | 3.207 | 1.1 | 2.914 | 0.0036 | 0.0119 |
| ENSG00000156475 | PPP2R2B | 3.201 | 0.675 | 4.742 | 0 | 0 |
| ENSG00000260977 |  | 3.201 | 0.664 | 4.823 | 0 | 0 |
| ENSG00000149534 | MS4A2 | 3.201 | 1.117 | 2.865 | 0.0042 | 0.0137 |
| ENSG00000239706 |  | 3.2 | 1.04 | 3.078 | 0.0021 | 0.0076 |
| ENSG00000170382 | LRRN2 | 3.194 | 1.062 | 3.007 | 0.0026 | 0.0092 |
| ENSG00000232486 |  | 3.193 | 0.579 | 5.515 | 0 | 0 |
| ENSG00000124143 | ARHGAP40 | 3.192 | 1.157 | 2.758 | 0.0058 | 0.0181 |
| ENSG00000280239 |  | 3.191 | 0.507 | 6.298 | 0 | 0 |
| ENSG00000270804 |  | 3.19 | 0.941 | 3.39 | 0.0007 | 0.003 |
| ENSG00000231691 |  | 3.189 | 1.167 | 2.732 | 0.0063 | 0.0193 |
| ENSG00000169439 | SDC2 | 3.185 | 0.39 | 8.176 | 0 | 0 |
| ENSG00000224416 |  | 3.185 | 0.945 | 3.37 | 0.0008 | 0.0032 |
| ENSG00000131845 | ZNF304 | 3.183 | 0.253 | 12.557 | 0 | 0 |
| ENSG00000227359 |  | 3.183 | 1.03 | 3.091 | 0.002 | 0.0073 |
| ENSG00000276747 | PADI6 | 3.183 | 1.104 | 2.883 | 0.0039 | 0.013 |
| ENSG00000122733 | PHF24 | 3.182 | 0.63 | 5.049 | 0 | 0 |
| ENSG00000276445 |  | 3.178 | 1.031 | 3.081 | 0.0021 | 0.0075 |
| ENSG00000152433 | ZNF547 | 3.176 | 0.331 | 9.592 | 0 | 0 |
| ENSG00000237265 | LOC100129291 | 3.174 | 0.88 | 3.606 | 0.0003 | 0.0015 |
| ENSG00000226284 |  | 3.172 | 0.687 | 4.616 | 0 | 0 |
| ENSG00000198711 | SSBP3-AS1 | 3.171 | 0.83 | 3.82 | 0.0001 | 0.0007 |
| ENSG00000249584 | LINC02225 | 3.171 | 0.96 | 3.302 | 0.001 | 0.0039 |
| ENSG00000134240 | HMGCS2 | 3.168 | 0.544 | 5.824 | 0 | 0 |
| ENSG00000185860 | CCDC190 | 3.165 | 0.467 | 6.777 | 0 | 0 |
| ENSG00000134548 | SPX | 3.156 | 0.557 | 5.665 | 0 | 0 |
| ENSG00000164404 | GDF9 | 3.155 | 0.278 | 11.365 | 0 | 0 |
| ENSG00000138109 | CYP2C9 | 3.155 | 0.923 | 3.42 | 0.0006 | 0.0027 |
| ENSG00000198092 | TMPRSS11F | 3.154 | 1.112 | 2.835 | 0.0046 | 0.0148 |
| ENSG00000143850 | PLEKHA6 | 3.152 | 0.379 | 8.318 | 0 | 0 |
| ENSG00000164692 | COL1A2 | 3.148 | 0.356 | 8.841 | 0 | 0 |
| ENSG00000271913 | LOC105378083 | 3.14 | 0.883 | 3.557 | 0.0004 | 0.0018 |
| ENSG00000185221 |  | 3.138 | 1.096 | 2.864 | 0.0042 | 0.0137 |
| ENSG00000135643 | KCNMB4 | 3.128 | 0.724 | 4.323 | 0 | 0.0001 |
| ENSG00000229105 | ASTN2-AS1 | 3.128 | 1.17 | 2.675 | 0.0075 | 0.0223 |
| ENSG00000011638 | TMEM159 | 3.123 | 0.343 | 9.102 | 0 | 0 |
| ENSG00000213621 | RPSAP54 | 3.123 | 1.059 | 2.947 | 0.0032 | 0.0109 |
| ENSG00000159708 | LRRC36 | 3.121 | 1.006 | 3.101 | 0.0019 | 0.0071 |
| ENSG00000215190 | GUSBP1 | 3.115 | 0.458 | 6.793 | 0 | 0 |
| ENSG00000236704 | CNOT4P1 | 3.107 | 0.706 | 4.398 | 0 | 0.0001 |
| ENSG00000148541 | FAM13C | 3.102 | 1.08 | 2.872 | 0.0041 | 0.0134 |
| ENSG00000253865 |  | 3.099 | 0.882 | 3.513 | 0.0004 | 0.002 |
| ENSG00000119547 | ONECUT2 | 3.098 | 0.441 | 7.019 | 0 | 0 |
| ENSG00000188761 | BCL2L15 | 3.096 | 0.926 | 3.345 | 0.0008 | 0.0035 |
| ENSG00000264546 |  | 3.096 | 1.229 | 2.52 | 0.0117 | 0.0322 |
| ENSG00000260112 |  | 3.094 | 0.828 | 3.734 | 0.0002 | 0.001 |
| ENSG00000196114 |  | 3.094 | 0.958 | 3.23 | 0.0012 | 0.0049 |
| ENSG00000215817 | ZC3H11B | 3.093 | 1.141 | 2.71 | 0.0067 | 0.0204 |
| ENSG00000214465 |  | 3.085 | 0.547 | 5.637 | 0 | 0 |
| ENSG00000171217 | CLDN20 | 3.085 | 1.004 | 3.074 | 0.0021 | 0.0076 |
| ENSG00000257199 |  | 3.084 | 0.644 | 4.786 | 0 | 0 |
| ENSG00000001084 | GCLC | 3.084 | 0.341 | 9.035 | 0 | 0 |
| ENSG00000241135 |  | 3.083 | 0.37 | 8.329 | 0 | 0 |
| ENSG00000281021 |  | 3.081 | 0.942 | 3.271 | 0.0011 | 0.0043 |
| ENSG00000269549 |  | 3.081 | 1.072 | 2.873 | 0.0041 | 0.0134 |
| ENSG00000170835 | CEL | 3.08 | 0.443 | 6.948 | 0 | 0 |
| ENSG00000106278 | PTPRZ1 | 3.078 | 1.116 | 2.757 | 0.0058 | 0.0181 |
| ENSG00000154227 | CERS3 | 3.074 | 0.795 | 3.866 | 0.0001 | 0.0006 |
| ENSG00000154316 | TDH | 3.069 | 0.867 | 3.541 | 0.0004 | 0.0019 |
| ENSG00000246363 | LINC02458 | 3.066 | 0.813 | 3.773 | 0.0002 | 0.0009 |
| ENSG00000215515 | IFIT1P1 | 3.066 | 1.088 | 2.819 | 0.0048 | 0.0154 |
| ENSG00000147647 | DPYS | 3.063 | 0.989 | 3.097 | 0.002 | 0.0072 |
| ENSG00000240280 | TCAM1P | 3.061 | 0.514 | 5.961 | 0 | 0 |
| ENSG00000279355 |  | 3.048 | 1.144 | 2.664 | 0.0077 | 0.0229 |
| ENSG00000183873 | SCN5A | 3.045 | 0.503 | 6.058 | 0 | 0 |
| ENSG00000121853 | GHSR | 3.043 | 1.222 | 2.49 | 0.0128 | 0.0345 |
| ENSG00000261610 |  | 3.041 | 1.039 | 2.928 | 0.0034 | 0.0115 |
| ENSG00000262712 |  | 3.04 | 0.503 | 6.039 | 0 | 0 |
| ENSG00000185345 | PRKN | 3.04 | 0.914 | 3.326 | 0.0009 | 0.0037 |
| ENSG00000182168 | UNC5C | 3.04 | 0.993 | 3.062 | 0.0022 | 0.0079 |
| ENSG00000258525 | LOC100506071 | 3.04 | 1.185 | 2.565 | 0.0103 | 0.029 |
| ENSG00000134871 | COL4A2 | 3.037 | 0.324 | 9.374 | 0 | 0 |
| ENSG00000156427 | FGF18 | 3.032 | 0.311 | 9.756 | 0 | 0 |
| ENSG00000183206 | POTEC | 3.025 | 0.773 | 3.915 | 0.0001 | 0.0005 |
| ENSG00000255815 | KRT8P11 | 3.021 | 0.539 | 5.606 | 0 | 0 |
| ENSG00000254037 |  | 3.018 | 1.084 | 2.783 | 0.0054 | 0.0169 |
| ENSG00000153563 | CD8A | 3.015 | 0.826 | 3.652 | 0.0003 | 0.0013 |
| ENSG00000182898 | TCHHL1 | 3.007 | 1.016 | 2.961 | 0.0031 | 0.0105 |
| ENSG00000248711 |  | 3.004 | 1.084 | 2.771 | 0.0056 | 0.0175 |
| ENSG00000178201 | VN1R1 | 3.002 | 0.923 | 3.252 | 0.0011 | 0.0046 |
| ENSG00000182871 | COL18A1 | 2.998 | 0.333 | 8.993 | 0 | 0 |
| ENSG00000242252 | BGLAP | 2.996 | 0.635 | 4.721 | 0 | 0 |
| ENSG00000262228 |  | 2.996 | 1.136 | 2.637 | 0.0084 | 0.0244 |
| ENSG00000164794 | KCNV1 | 2.988 | 0.888 | 3.363 | 0.0008 | 0.0033 |
| ENSG00000241941 | RPL32P26 | 2.981 | 1.127 | 2.646 | 0.0081 | 0.0238 |
| ENSG00000248455 | LINC02217 | 2.972 | 0.947 | 3.138 | 0.0017 | 0.0064 |
| ENSG00000092607 | TBX15 | 2.969 | 0.566 | 5.249 | 0 | 0 |
| ENSG00000101255 | TRIB3 | 2.966 | 0.318 | 9.333 | 0 | 0 |
| ENSG00000084636 | COL16A1 | 2.965 | 0.267 | 11.109 | 0 | 0 |
| ENSG00000168280 | KIF5C | 2.965 | 0.817 | 3.631 | 0.0003 | 0.0014 |
| ENSG00000145721 | LIX1 | 2.961 | 1.01 | 2.931 | 0.0034 | 0.0114 |
| ENSG00000119714 | GPR68 | 2.96 | 0.359 | 8.233 | 0 | 0 |
| ENSG00000153898 | MCOLN2 | 2.959 | 0.562 | 5.263 | 0 | 0 |
| ENSG00000280157 |  | 2.957 | 0.848 | 3.486 | 0.0005 | 0.0022 |
| ENSG00000162891 | IL20 | 2.957 | 0.873 | 3.388 | 0.0007 | 0.003 |
| ENSG00000188848 | BEND4 | 2.955 | 0.46 | 6.419 | 0 | 0 |
| ENSG00000168675 | LDLRAD4 | 2.95 | 0.322 | 9.157 | 0 | 0 |
| ENSG00000259450 |  | 2.947 | 0.641 | 4.599 | 0 | 0 |
| ENSG00000232518 |  | 2.947 | 1.07 | 2.755 | 0.0059 | 0.0182 |
| ENSG00000227379 |  | 2.946 | 0.81 | 3.636 | 0.0003 | 0.0014 |
| ENSG00000158560 | DYNC1I1 | 2.933 | 0.84 | 3.489 | 0.0005 | 0.0022 |
| ENSG00000198156 | NPIPB6 | 2.929 | 1.03 | 2.845 | 0.0044 | 0.0144 |
| ENSG00000198216 | CACNA1E | 2.928 | 0.632 | 4.635 | 0 | 0 |
| ENSG00000237638 | LINC02245 | 2.927 | 0.641 | 4.568 | 0 | 0 |
| ENSG00000061455 | PRDM6 | 2.925 | 0.61 | 4.798 | 0 | 0 |
| ENSG00000266613 | COP1P1 | 2.922 | 0.915 | 3.193 | 0.0014 | 0.0055 |
| ENSG00000105507 | CABP5 | 2.922 | 1.025 | 2.85 | 0.0044 | 0.0142 |
| ENSG00000224153 | LINC02054 | 2.921 | 1.143 | 2.557 | 0.0106 | 0.0295 |
| ENSG00000271425 | NBPF10 | 2.92 | 0.391 | 7.474 | 0 | 0 |
| ENSG00000182308 | DCAF4L1 | 2.918 | 0.259 | 11.262 | 0 | 0 |
| ENSG00000132874 | SLC14A2 | 2.916 | 0.454 | 6.425 | 0 | 0 |
| ENSG00000258472 |  | 2.916 | 0.913 | 3.193 | 0.0014 | 0.0055 |
| ENSG00000100473 | COCH | 2.912 | 0.79 | 3.685 | 0.0002 | 0.0012 |
| ENSG00000263232 | ATP5F1AP3 | 2.911 | 0.638 | 4.56 | 0 | 0 |
| ENSG00000231574 | LINC02015 | 2.911 | 1.082 | 2.692 | 0.0071 | 0.0214 |
| ENSG00000104043 | ATP8B4 | 2.908 | 0.397 | 7.324 | 0 | 0 |
| ENSG00000250130 |  | 2.907 | 1.017 | 2.859 | 0.0042 | 0.0139 |
| ENSG00000274443 | C8orf89 | 2.907 | 1.187 | 2.45 | 0.0143 | 0.0378 |
| ENSG00000257893 | LINC02404 | 2.905 | 1.109 | 2.619 | 0.0088 | 0.0254 |
| ENSG00000203867 | RBM20 | 2.904 | 0.976 | 2.975 | 0.0029 | 0.0101 |
| ENSG00000205702 | CYP2D7 | 2.904 | 1.095 | 2.651 | 0.008 | 0.0236 |
| ENSG00000196074 | SYCP2 | 2.9 | 0.631 | 4.595 | 0 | 0 |
| ENSG00000148600 | CDHR1 | 2.9 | 0.771 | 3.764 | 0.0002 | 0.0009 |
| ENSG00000134375 | TIMM17A | 2.898 | 0.367 | 7.89 | 0 | 0 |
| ENSG00000253611 | VN1R46P | 2.897 | 0.835 | 3.472 | 0.0005 | 0.0023 |
| ENSG00000081237 | PTPRC | 2.895 | 0.604 | 4.79 | 0 | 0 |
| ENSG00000116701 | NCF2 | 2.893 | 0.466 | 6.207 | 0 | 0 |
| ENSG00000173728 | C1orf100 | 2.89 | 1.008 | 2.867 | 0.0041 | 0.0136 |
| ENSG00000104371 | DKK4 | 2.889 | 0.787 | 3.673 | 0.0002 | 0.0012 |
| ENSG00000223930 |  | 2.889 | 1.091 | 2.648 | 0.0081 | 0.0238 |
| ENSG00000181195 | PENK | 2.887 | 0.379 | 7.624 | 0 | 0 |
| ENSG00000259378 |  | 2.883 | 0.874 | 3.3 | 0.001 | 0.004 |
| ENSG00000280287 |  | 2.882 | 0.43 | 6.71 | 0 | 0 |
| ENSG00000164318 | EGFLAM | 2.882 | 0.904 | 3.186 | 0.0014 | 0.0056 |
| ENSG00000226329 |  | 2.88 | 0.393 | 7.323 | 0 | 0 |
| ENSG00000198939 | ZFP2 | 2.88 | 0.711 | 4.054 | 0.0001 | 0.0003 |
| ENSG00000225082 |  | 2.879 | 1.158 | 2.487 | 0.0129 | 0.0348 |
| ENSG00000266242 | GRAMD4P7 | 2.877 | 0.568 | 5.062 | 0 | 0 |
| ENSG00000254999 | BRK1 | 2.875 | 0.373 | 7.714 | 0 | 0 |
| ENSG00000219747 |  | 2.873 | 0.973 | 2.952 | 0.0032 | 0.0108 |
| ENSG00000180815 | MAP3K15 | 2.871 | 0.718 | 4 | 0.0001 | 0.0004 |
| ENSG00000226161 |  | 2.868 | 0.509 | 5.637 | 0 | 0 |
| ENSG00000263724 | DLGAP1-AS3 | 2.865 | 1.033 | 2.775 | 0.0055 | 0.0173 |
| ENSG00000277504 |  | 2.864 | 0.528 | 5.423 | 0 | 0 |
| ENSG00000277151 |  | 2.861 | 0.896 | 3.195 | 0.0014 | 0.0054 |
| ENSG00000212123 | PRR22 | 2.857 | 0.359 | 7.968 | 0 | 0 |
| ENSG00000263874 | LINC00672 | 2.854 | 0.457 | 6.252 | 0 | 0 |
| ENSG00000242613 |  | 2.854 | 1.024 | 2.786 | 0.0053 | 0.0168 |
| ENSG00000233223 | LOC100996842 | 2.852 | 0.262 | 10.905 | 0 | 0 |
| ENSG00000122304 | PRM2 | 2.85 | 0.815 | 3.497 | 0.0005 | 0.0021 |
| ENSG00000181171 | FER1L6-AS1 | 2.843 | 1.029 | 2.763 | 0.0057 | 0.0179 |
| ENSG00000092850 | TEKT2 | 2.842 | 0.512 | 5.555 | 0 | 0 |
| ENSG00000116521 | SCAMP3 | 2.84 | 0.291 | 9.763 | 0 | 0 |
| ENSG00000236334 | PPIAL4G | 2.839 | 0.639 | 4.444 | 0 | 0.0001 |
| ENSG00000251467 |  | 2.836 | 0.811 | 3.496 | 0.0005 | 0.0022 |
| ENSG00000259677 |  | 2.835 | 1.14 | 2.488 | 0.0129 | 0.0347 |
| ENSG00000083307 | GRHL2 | 2.833 | 0.583 | 4.859 | 0 | 0 |
| ENSG00000089692 | LAG3 | 2.832 | 0.59 | 4.798 | 0 | 0 |
| ENSG00000163501 | IHH | 2.831 | 0.79 | 3.583 | 0.0003 | 0.0016 |
| ENSG00000205856 | C22orf42 | 2.831 | 1.15 | 2.461 | 0.0138 | 0.0368 |
| ENSG00000230825 | LOC101927354 | 2.822 | 0.557 | 5.069 | 0 | 0 |
| ENSG00000219553 |  | 2.821 | 0.517 | 5.457 | 0 | 0 |
| ENSG00000263606 |  | 2.819 | 1.024 | 2.754 | 0.0059 | 0.0183 |
| ENSG00000181322 | NME9 | 2.818 | 1.031 | 2.732 | 0.0063 | 0.0193 |
| ENSG00000250426 | FTLP10 | 2.816 | 1.095 | 2.572 | 0.0101 | 0.0285 |
| ENSG00000224858 |  | 2.809 | 1.085 | 2.588 | 0.0096 | 0.0274 |
| ENSG00000198838 | RYR3 | 2.808 | 0.458 | 6.127 | 0 | 0 |
| ENSG00000260036 |  | 2.808 | 0.811 | 3.464 | 0.0005 | 0.0024 |
| ENSG00000278703 |  | 2.807 | 1.026 | 2.737 | 0.0062 | 0.0191 |
| ENSG00000280184 |  | 2.806 | 0.488 | 5.748 | 0 | 0 |
| ENSG00000160993 | ALKBH4 | 2.803 | 0.395 | 7.095 | 0 | 0 |
| ENSG00000189127 | ANKRD34B | 2.796 | 1.049 | 2.665 | 0.0077 | 0.0228 |
| ENSG00000271715 |  | 2.795 | 0.64 | 4.365 | 0 | 0.0001 |
| ENSG00000255833 | TIFAB | 2.792 | 1.18 | 2.365 | 0.018 | 0.0458 |
| ENSG00000005022 | SLC25A5 | 2.791 | 0.37 | 7.552 | 0 | 0 |
| ENSG00000233018 |  | 2.789 | 0.587 | 4.749 | 0 | 0 |
| ENSG00000134193 | REG4 | 2.789 | 0.435 | 6.408 | 0 | 0 |
| ENSG00000278000 |  | 2.784 | 0.284 | 9.79 | 0 | 0 |
| ENSG00000064547 | LPAR2 | 2.782 | 0.582 | 4.784 | 0 | 0 |
| ENSG00000156194 | PPEF2 | 2.78 | 0.346 | 8.043 | 0 | 0 |
| ENSG00000115604 | IL18R1 | 2.78 | 0.682 | 4.077 | 0 | 0.0003 |
| ENSG00000122574 | WIPF3 | 2.779 | 1.036 | 2.682 | 0.0073 | 0.0219 |
| ENSG00000183114 | FAM43B | 2.777 | 0.859 | 3.235 | 0.0012 | 0.0048 |
| ENSG00000254363 | LOC101929719 | 2.776 | 0.889 | 3.124 | 0.0018 | 0.0066 |
| ENSG00000125492 | BARHL1 | 2.776 | 0.917 | 3.027 | 0.0025 | 0.0087 |
| ENSG00000224660 | SH3BP5-AS1 | 2.775 | 0.377 | 7.353 | 0 | 0 |
| ENSG00000219736 | LOC100127900 | 2.774 | 0.981 | 2.827 | 0.0047 | 0.0151 |
| ENSG00000227456 | LINC00310 | 2.771 | 0.471 | 5.884 | 0 | 0 |
| ENSG00000213060 |  | 2.771 | 0.739 | 3.753 | 0.0002 | 0.0009 |
| ENSG00000280078 |  | 2.77 | 1.174 | 2.36 | 0.0183 | 0.0464 |
| ENSG00000261056 |  | 2.768 | 1.023 | 2.707 | 0.0068 | 0.0206 |
| ENSG00000214788 |  | 2.765 | 0.862 | 3.208 | 0.0013 | 0.0052 |
| ENSG00000171815 | PCDHB1 | 2.756 | 0.949 | 2.903 | 0.0037 | 0.0123 |
| ENSG00000109927 | TECTA | 2.752 | 0.466 | 5.906 | 0 | 0 |
| ENSG00000153064 | BANK1 | 2.752 | 0.662 | 4.156 | 0 | 0.0002 |
| ENSG00000214093 | RPS18P14 | 2.747 | 0.913 | 3.01 | 0.0026 | 0.0092 |
| ENSG00000162843 | WDR64 | 2.746 | 0.942 | 2.916 | 0.0035 | 0.0119 |
| ENSG00000197083 |  | 2.745 | 0.453 | 6.062 | 0 | 0 |
| ENSG00000231234 |  | 2.744 | 0.733 | 3.744 | 0.0002 | 0.0009 |
| ENSG00000150594 | ADRA2A | 2.741 | 0.433 | 6.324 | 0 | 0 |
| ENSG00000258806 |  | 2.736 | 0.556 | 4.918 | 0 | 0 |
| ENSG00000185482 | STAC3 | 2.734 | 0.465 | 5.878 | 0 | 0 |
| ENSG00000110367 | DDX6 | 2.734 | 0.231 | 11.854 | 0 | 0 |
| ENSG00000168703 | WFDC12 | 2.731 | 0.969 | 2.819 | 0.0048 | 0.0154 |
| ENSG00000240038 | AMY2B | 2.727 | 0.468 | 5.826 | 0 | 0 |
| ENSG00000277959 |  | 2.726 | 0.571 | 4.776 | 0 | 0 |
| ENSG00000267346 |  | 2.723 | 1.07 | 2.544 | 0.011 | 0.0304 |
| ENSG00000279184 |  | 2.722 | 0.525 | 5.189 | 0 | 0 |
| ENSG00000266954 |  | 2.722 | 1.031 | 2.641 | 0.0083 | 0.0241 |
| ENSG00000238002 | NPAP1P6 | 2.721 | 0.93 | 2.926 | 0.0034 | 0.0116 |
| ENSG00000254233 | LINC02365 | 2.72 | 0.913 | 2.979 | 0.0029 | 0.01 |
| ENSG00000176971 | FIBIN | 2.719 | 0.442 | 6.147 | 0 | 0 |
| ENSG00000262117 | BCAR4 | 2.712 | 0.374 | 7.248 | 0 | 0 |
| ENSG00000112499 | SLC22A2 | 2.711 | 0.364 | 7.449 | 0 | 0 |
| ENSG00000280266 |  | 2.709 | 0.541 | 5.003 | 0 | 0 |
| ENSG00000130203 | APOE | 2.706 | 0.418 | 6.478 | 0 | 0 |
| ENSG00000131730 | CKMT2 | 2.706 | 0.571 | 4.736 | 0 | 0 |
| ENSG00000133116 | KL | 2.705 | 0.575 | 4.708 | 0 | 0 |
| ENSG00000181016 | LSMEM1 | 2.703 | 0.338 | 8.006 | 0 | 0 |
| ENSG00000234383 |  | 2.699 | 0.532 | 5.077 | 0 | 0 |
| ENSG00000241157 | RPL32P32 | 2.699 | 0.826 | 3.269 | 0.0011 | 0.0044 |
| ENSG00000277879 |  | 2.696 | 0.756 | 3.567 | 0.0004 | 0.0017 |
| ENSG00000272669 |  | 2.687 | 0.615 | 4.365 | 0 | 0.0001 |
| ENSG00000205853 | RFPL3S | 2.687 | 0.846 | 3.175 | 0.0015 | 0.0057 |
| ENSG00000251453 |  | 2.687 | 0.891 | 3.015 | 0.0026 | 0.009 |
| ENSG00000130173 | ANGPTL8 | 2.686 | 0.701 | 3.829 | 0.0001 | 0.0007 |
| ENSG00000223731 |  | 2.684 | 1.094 | 2.454 | 0.0141 | 0.0375 |
| ENSG00000153879 | CEBPG | 2.682 | 0.211 | 12.741 | 0 | 0 |
| ENSG00000180720 | CHRM4 | 2.682 | 0.562 | 4.771 | 0 | 0 |
| ENSG00000249055 |  | 2.68 | 1.03 | 2.602 | 0.0093 | 0.0265 |
| ENSG00000170091 | NSG2 | 2.678 | 0.444 | 6.027 | 0 | 0 |
| ENSG00000115705 | TPO | 2.676 | 0.642 | 4.168 | 0 | 0.0002 |
| ENSG00000227197 |  | 2.676 | 1.141 | 2.344 | 0.0191 | 0.0479 |
| ENSG00000203288 | TDRKH-AS1 | 2.671 | 1.071 | 2.494 | 0.0126 | 0.0342 |
| ENSG00000230739 |  | 2.67 | 0.957 | 2.788 | 0.0053 | 0.0167 |
| ENSG00000021461 | CYP3A43 | 2.67 | 1.146 | 2.329 | 0.0198 | 0.0496 |
| ENSG00000174652 | ZNF266 | 2.668 | 0.296 | 9.019 | 0 | 0 |
| ENSG00000111644 | ACRBP | 2.665 | 0.5 | 5.334 | 0 | 0 |
| ENSG00000261512 |  | 2.665 | 0.913 | 2.919 | 0.0035 | 0.0118 |
| ENSG00000226825 |  | 2.664 | 1.126 | 2.367 | 0.0179 | 0.0457 |
| ENSG00000224261 | RPSAP18 | 2.663 | 0.753 | 3.537 | 0.0004 | 0.0019 |
| ENSG00000223694 | ADH5P3 | 2.661 | 0.744 | 3.576 | 0.0003 | 0.0017 |
| ENSG00000134539 | KLRD1 | 2.66 | 0.4 | 6.655 | 0 | 0 |
| ENSG00000139116 | KIF21A | 2.659 | 0.542 | 4.903 | 0 | 0 |
| ENSG00000144214 | LYG1 | 2.657 | 0.639 | 4.157 | 0 | 0.0002 |
| ENSG00000247131 | LOC101928002 | 2.654 | 0.902 | 2.943 | 0.0033 | 0.0111 |
| ENSG00000130653 | PNPLA7 | 2.653 | 0.499 | 5.321 | 0 | 0 |
| ENSG00000283599 | LOC101059915 | 2.652 | 0.614 | 4.32 | 0 | 0.0001 |
| ENSG00000161992 | PRR35 | 2.65 | 0.54 | 4.913 | 0 | 0 |
| ENSG00000090382 | LYZ | 2.648 | 1.047 | 2.529 | 0.0114 | 0.0315 |
| ENSG00000255746 | LOC102723544 | 2.648 | 1.063 | 2.492 | 0.0127 | 0.0344 |
| ENSG00000276073 |  | 2.647 | 0.898 | 2.95 | 0.0032 | 0.0108 |
| ENSG00000213228 |  | 2.646 | 0.342 | 7.749 | 0 | 0 |
| ENSG00000233061 |  | 2.646 | 1.046 | 2.529 | 0.0114 | 0.0315 |
| ENSG00000271419 | LOC100421116 | 2.646 | 1.064 | 2.488 | 0.0129 | 0.0347 |
| ENSG00000283683 | MYOCOS | 2.64 | 0.524 | 5.036 | 0 | 0 |
| ENSG00000225017 | LOC100420057 | 2.637 | 0.973 | 2.709 | 0.0068 | 0.0204 |
| ENSG00000260190 |  | 2.634 | 0.741 | 3.557 | 0.0004 | 0.0018 |
| ENSG00000205879 |  | 2.634 | 0.997 | 2.641 | 0.0083 | 0.0241 |
| ENSG00000263635 |  | 2.633 | 1.108 | 2.376 | 0.0175 | 0.0448 |
| ENSG00000206149 | HERC2P9 | 2.63 | 0.553 | 4.753 | 0 | 0 |
| ENSG00000249082 | C5orf66-AS1 | 2.63 | 0.998 | 2.636 | 0.0084 | 0.0244 |
| ENSG00000231305 |  | 2.629 | 0.365 | 7.208 | 0 | 0 |
| ENSG00000223403 | MEG9 | 2.629 | 0.368 | 7.136 | 0 | 0 |
| ENSG00000155506 | LARP1 | 2.628 | 0.225 | 11.675 | 0 | 0 |
| ENSG00000196660 | SLC30A10 | 2.627 | 0.469 | 5.595 | 0 | 0 |
| ENSG00000137491 | SLCO2B1 | 2.626 | 0.812 | 3.233 | 0.0012 | 0.0049 |
| ENSG00000240207 |  | 2.625 | 0.558 | 4.704 | 0 | 0 |
| ENSG00000248132 | LINC02101 | 2.624 | 1.067 | 2.459 | 0.0139 | 0.0371 |
| ENSG00000214282 | KRT8P14 | 2.623 | 0.358 | 7.318 | 0 | 0 |
| ENSG00000114270 | COL7A1 | 2.622 | 0.244 | 10.736 | 0 | 0 |
| ENSG00000135973 | GPR45 | 2.622 | 0.538 | 4.869 | 0 | 0 |
| ENSG00000139351 | SYCP3 | 2.622 | 0.993 | 2.641 | 0.0083 | 0.0241 |
| ENSG00000143512 | HHIPL2 | 2.618 | 0.948 | 2.763 | 0.0057 | 0.0179 |
| ENSG00000198633 | ZNF534 | 2.616 | 0.649 | 4.028 | 0.0001 | 0.0003 |
| ENSG00000070031 | SCT | 2.616 | 0.798 | 3.28 | 0.001 | 0.0042 |
| ENSG00000251381 | LINC00958 | 2.615 | 0.48 | 5.45 | 0 | 0 |
| ENSG00000185615 | PDIA2 | 2.611 | 0.576 | 4.531 | 0 | 0 |
| ENSG00000251595 | ABCA11P | 2.611 | 1.015 | 2.571 | 0.0101 | 0.0285 |
| ENSG00000008226 | DLEC1 | 2.61 | 0.757 | 3.449 | 0.0006 | 0.0025 |
| ENSG00000187950 | OVCH1 | 2.609 | 0.983 | 2.655 | 0.0079 | 0.0234 |
| ENSG00000236539 |  | 2.607 | 0.543 | 4.8 | 0 | 0 |
| ENSG00000087253 | LPCAT2 | 2.602 | 0.277 | 9.4 | 0 | 0 |
| ENSG00000250033 | SLC7A11-AS1 | 2.601 | 0.596 | 4.364 | 0 | 0.0001 |
| ENSG00000118137 | APOA1 | 2.6 | 0.562 | 4.625 | 0 | 0 |
| ENSG00000249437 | NAIP | 2.598 | 0.788 | 3.297 | 0.001 | 0.004 |
| ENSG00000235049 | LINC00940 | 2.598 | 0.988 | 2.631 | 0.0085 | 0.0247 |
| ENSG00000198231 | DDX42 | 2.596 | 0.25 | 10.403 | 0 | 0 |
| ENSG00000152380 | FAM151B | 2.596 | 0.671 | 3.867 | 0.0001 | 0.0006 |
| ENSG00000159753 | CARMIL2 | 2.593 | 0.29 | 8.941 | 0 | 0 |
| ENSG00000213561 |  | 2.593 | 0.63 | 4.115 | 0 | 0.0002 |
| ENSG00000230067 |  | 2.593 | 0.751 | 3.452 | 0.0006 | 0.0025 |
| ENSG00000215861 |  | 2.592 | 0.722 | 3.592 | 0.0003 | 0.0016 |
| ENSG00000226261 |  | 2.589 | 0.64 | 4.045 | 0.0001 | 0.0003 |
| ENSG00000204010 | IFIT1B | 2.588 | 0.448 | 5.774 | 0 | 0 |
| ENSG00000196503 | ARL9 | 2.587 | 0.545 | 4.75 | 0 | 0 |
| ENSG00000213432 | RPL17P34 | 2.583 | 0.897 | 2.88 | 0.004 | 0.0131 |
| ENSG00000086730 | LAT2 | 2.577 | 0.309 | 8.351 | 0 | 0 |
| ENSG00000267769 |  | 2.577 | 0.899 | 2.868 | 0.0041 | 0.0136 |
| ENSG00000257838 | OTOAP1 | 2.575 | 0.529 | 4.865 | 0 | 0 |
| ENSG00000188820 | CALHM6 | 2.573 | 0.822 | 3.131 | 0.0017 | 0.0065 |
| ENSG00000184060 | ADAP2 | 2.567 | 1.02 | 2.516 | 0.0119 | 0.0324 |
| ENSG00000186417 | GLDN | 2.565 | 0.475 | 5.401 | 0 | 0 |
| ENSG00000168661 | ZNF30 | 2.564 | 0.526 | 4.873 | 0 | 0 |
| ENSG00000070718 | AP3M2 | 2.56 | 0.266 | 9.622 | 0 | 0 |
| ENSG00000113494 | PRLR | 2.558 | 0.974 | 2.628 | 0.0086 | 0.0249 |
| ENSG00000139988 | RDH12 | 2.555 | 0.876 | 2.916 | 0.0035 | 0.0119 |
| ENSG00000267131 | LOC101927855 | 2.555 | 1.09 | 2.343 | 0.0191 | 0.0481 |
| ENSG00000160161 | CILP2 | 2.551 | 0.353 | 7.228 | 0 | 0 |
| ENSG00000260213 |  | 2.549 | 0.73 | 3.491 | 0.0005 | 0.0022 |
| ENSG00000138308 | PLA2G12B | 2.549 | 0.948 | 2.689 | 0.0072 | 0.0215 |
| ENSG00000104689 | TNFRSF10A | 2.548 | 0.363 | 7.013 | 0 | 0 |
| ENSG00000236467 | KCNMA1-AS1 | 2.548 | 0.772 | 3.299 | 0.001 | 0.004 |
| ENSG00000204193 | TXNDC8 | 2.547 | 0.683 | 3.728 | 0.0002 | 0.001 |
| ENSG00000095932 | SMIM24 | 2.546 | 0.654 | 3.892 | 0.0001 | 0.0006 |
| ENSG00000184995 | IFNE | 2.545 | 0.783 | 3.252 | 0.0011 | 0.0046 |
| ENSG00000182220 | ATP6AP2 | 2.542 | 0.326 | 7.789 | 0 | 0 |
| ENSG00000164935 | DCSTAMP | 2.541 | 0.93 | 2.733 | 0.0063 | 0.0193 |
| ENSG00000175189 | INHBC | 2.54 | 0.793 | 3.204 | 0.0014 | 0.0053 |
| ENSG00000254285 | KRT8P3 | 2.539 | 0.937 | 2.71 | 0.0067 | 0.0204 |
| ENSG00000248480 |  | 2.538 | 0.572 | 4.441 | 0 | 0.0001 |
| ENSG00000253738 | OTUD6B-AS1 | 2.533 | 0.271 | 9.354 | 0 | 0 |
| ENSG00000175197 | DDIT3 | 2.533 | 0.281 | 9.025 | 0 | 0 |
| ENSG00000233579 | KRT8P15 | 2.533 | 0.538 | 4.706 | 0 | 0 |
| ENSG00000224383 | PRR29 | 2.533 | 0.398 | 6.361 | 0 | 0 |
| ENSG00000169194 | IL13 | 2.532 | 0.396 | 6.393 | 0 | 0 |
| ENSG00000166558 | SLC38A8 | 2.529 | 0.797 | 3.172 | 0.0015 | 0.0058 |
| ENSG00000106605 | BLVRA | 2.528 | 0.86 | 2.938 | 0.0033 | 0.0112 |
| ENSG00000104331 | BPNT2 | 2.526 | 0.358 | 7.063 | 0 | 0 |
| ENSG00000253205 |  | 2.525 | 0.874 | 2.89 | 0.0038 | 0.0127 |
| ENSG00000129437 | KLK14 | 2.523 | 0.933 | 2.705 | 0.0068 | 0.0207 |
| ENSG00000145113 | MUC4 | 2.52 | 0.582 | 4.329 | 0 | 0.0001 |
| ENSG00000224080 |  | 2.516 | 0.394 | 6.39 | 0 | 0 |
| ENSG00000280181 |  | 2.515 | 0.877 | 2.867 | 0.0041 | 0.0136 |
| ENSG00000053108 | FSTL4 | 2.513 | 0.357 | 7.033 | 0 | 0 |
| ENSG00000253666 |  | 2.512 | 1.059 | 2.372 | 0.0177 | 0.0452 |
| ENSG00000220494 | YAP1P1 | 2.51 | 0.641 | 3.919 | 0.0001 | 0.0005 |
| ENSG00000166006 | KCNC2 | 2.51 | 0.849 | 2.957 | 0.0031 | 0.0106 |
| ENSG00000280924 | LINC00628 | 2.508 | 0.579 | 4.329 | 0 | 0.0001 |
| ENSG00000118407 | FILIP1 | 2.508 | 0.909 | 2.759 | 0.0058 | 0.0181 |
| ENSG00000170917 | NUDT6 | 2.506 | 0.314 | 7.982 | 0 | 0 |
| ENSG00000233845 |  | 2.502 | 1.031 | 2.428 | 0.0152 | 0.0398 |
| ENSG00000250081 |  | 2.5 | 0.848 | 2.949 | 0.0032 | 0.0109 |
| ENSG00000160117 | ANKLE1 | 2.498 | 0.644 | 3.876 | 0.0001 | 0.0006 |
| ENSG00000130338 | TULP4 | 2.497 | 0.267 | 9.343 | 0 | 0 |
| ENSG00000108176 | DNAJC12 | 2.497 | 0.708 | 3.525 | 0.0004 | 0.002 |
| ENSG00000153561 | RMND5A | 2.494 | 0.259 | 9.612 | 0 | 0 |
| ENSG00000237186 | RPS8P5 | 2.491 | 0.564 | 4.414 | 0 | 0.0001 |
| ENSG00000101049 | SGK2 | 2.491 | 0.944 | 2.639 | 0.0083 | 0.0242 |
| ENSG00000250673 | REELD1 | 2.49 | 0.822 | 3.028 | 0.0025 | 0.0087 |
| ENSG00000232667 |  | 2.484 | 0.878 | 2.83 | 0.0047 | 0.015 |
| ENSG00000217482 | HMGB1P17 | 2.484 | 0.989 | 2.51 | 0.0121 | 0.0329 |
| ENSG00000213892 | CEACAM16 | 2.483 | 0.782 | 3.177 | 0.0015 | 0.0057 |
| ENSG00000229858 |  | 2.482 | 0.906 | 2.741 | 0.0061 | 0.0189 |
| ENSG00000254934 | LINC00678 | 2.479 | 0.858 | 2.891 | 0.0038 | 0.0127 |
| ENSG00000232750 |  | 2.478 | 0.971 | 2.552 | 0.0107 | 0.0298 |
| ENSG00000160051 | IQCC | 2.477 | 0.266 | 9.307 | 0 | 0 |
| ENSG00000243697 | RPL7AP63 | 2.476 | 0.516 | 4.796 | 0 | 0 |
| ENSG00000134532 | SOX5 | 2.474 | 1.005 | 2.46 | 0.0139 | 0.0369 |
| ENSG00000087589 | CASS4 | 2.473 | 0.338 | 7.326 | 0 | 0 |
| ENSG00000226406 | RBMX2P1 | 2.468 | 0.717 | 3.443 | 0.0006 | 0.0025 |
| ENSG00000260466 |  | 2.465 | 0.783 | 3.149 | 0.0016 | 0.0062 |
| ENSG00000145040 | UCN2 | 2.462 | 0.419 | 5.878 | 0 | 0 |
| ENSG00000261087 | ZNNT1 | 2.462 | 0.732 | 3.362 | 0.0008 | 0.0033 |
| ENSG00000127914 | AKAP9 | 2.461 | 0.264 | 9.306 | 0 | 0 |
| ENSG00000264570 |  | 2.459 | 0.911 | 2.7 | 0.0069 | 0.0209 |
| ENSG00000274204 |  | 2.459 | 0.916 | 2.684 | 0.0073 | 0.0218 |
| ENSG00000203734 | ECT2L | 2.457 | 0.94 | 2.614 | 0.0089 | 0.0257 |
| ENSG00000131480 | AOC2 | 2.453 | 0.295 | 8.318 | 0 | 0 |
| ENSG00000249647 | C5orf66-AS2 | 2.453 | 0.964 | 2.545 | 0.0109 | 0.0304 |
| ENSG00000182866 | LCK | 2.452 | 0.479 | 5.123 | 0 | 0 |
| ENSG00000205534 |  | 2.45 | 0.617 | 3.97 | 0.0001 | 0.0004 |
| ENSG00000261764 | KRT18P18 | 2.449 | 0.813 | 3.013 | 0.0026 | 0.0091 |
| ENSG00000188996 | HUS1B | 2.449 | 0.936 | 2.617 | 0.0089 | 0.0256 |
| ENSG00000229468 |  | 2.448 | 0.963 | 2.543 | 0.011 | 0.0305 |
| ENSG00000213104 |  | 2.445 | 0.414 | 5.912 | 0 | 0 |
| ENSG00000117601 | SERPINC1 | 2.444 | 0.603 | 4.055 | 0.0001 | 0.0003 |
| ENSG00000196684 | HSH2D | 2.443 | 0.527 | 4.634 | 0 | 0 |
| ENSG00000203710 | CR1 | 2.44 | 0.826 | 2.955 | 0.0031 | 0.0107 |
| ENSG00000164674 | SYTL3 | 2.439 | 0.393 | 6.2 | 0 | 0 |
| ENSG00000186714 | CCDC73 | 2.438 | 0.855 | 2.85 | 0.0044 | 0.0142 |
| ENSG00000152154 | TMEM178A | 2.437 | 0.496 | 4.917 | 0 | 0 |
| ENSG00000174776 | WDR49 | 2.433 | 0.947 | 2.569 | 0.0102 | 0.0286 |
| ENSG00000283526 | PRRT1B | 2.432 | 0.919 | 2.648 | 0.0081 | 0.0238 |
| ENSG00000270890 |  | 2.431 | 0.748 | 3.249 | 0.0012 | 0.0046 |
| ENSG00000070770 | CSNK2A2 | 2.428 | 0.311 | 7.803 | 0 | 0 |
| ENSG00000229436 |  | 2.426 | 1.037 | 2.339 | 0.0193 | 0.0485 |
| ENSG00000120669 | SOHLH2 | 2.424 | 0.716 | 3.386 | 0.0007 | 0.0031 |
| ENSG00000253585 |  | 2.424 | 0.907 | 2.673 | 0.0075 | 0.0223 |
| ENSG00000165025 | SYK | 2.423 | 0.507 | 4.778 | 0 | 0 |
| ENSG00000189001 | SBSN | 2.423 | 0.498 | 4.869 | 0 | 0 |
| ENSG00000170264 | FAM161A | 2.42 | 0.345 | 7.012 | 0 | 0 |
| ENSG00000182795 | C1orf116 | 2.42 | 0.393 | 6.151 | 0 | 0 |
| ENSG00000236512 | RPL29P1 | 2.419 | 0.904 | 2.675 | 0.0075 | 0.0222 |
| ENSG00000244159 |  | 2.418 | 0.649 | 3.726 | 0.0002 | 0.001 |
| ENSG00000177791 | MYOZ1 | 2.417 | 0.564 | 4.284 | 0 | 0.0001 |
| ENSG00000249898 |  | 2.417 | 0.76 | 3.179 | 0.0015 | 0.0057 |
| ENSG00000232229 | LINC00865 | 2.416 | 0.782 | 3.089 | 0.002 | 0.0073 |
| ENSG00000254793 | FDPSP4 | 2.415 | 0.689 | 3.503 | 0.0005 | 0.0021 |
| ENSG00000185477 | GPRIN3 | 2.414 | 0.987 | 2.445 | 0.0145 | 0.0382 |
| ENSG00000143248 | RGS5 | 2.413 | 0.734 | 3.289 | 0.001 | 0.0041 |
| ENSG00000243396 | RPL32P7 | 2.413 | 0.987 | 2.444 | 0.0145 | 0.0384 |
| ENSG00000243824 | RPL12P6 | 2.412 | 0.967 | 2.495 | 0.0126 | 0.0341 |
| ENSG00000236296 | GUSBP5 | 2.41 | 0.431 | 5.597 | 0 | 0 |
| ENSG00000266968 |  | 2.41 | 1.001 | 2.407 | 0.0161 | 0.0417 |
| ENSG00000257818 |  | 2.408 | 0.79 | 3.048 | 0.0023 | 0.0082 |
| ENSG00000204920 | ZNF155 | 2.407 | 0.449 | 5.365 | 0 | 0 |
| ENSG00000164690 | SHH | 2.407 | 0.337 | 7.149 | 0 | 0 |
| ENSG00000262061 |  | 2.407 | 0.883 | 2.726 | 0.0064 | 0.0196 |
| ENSG00000254615 |  | 2.406 | 0.302 | 7.967 | 0 | 0 |
| ENSG00000243155 |  | 2.406 | 0.598 | 4.025 | 0.0001 | 0.0003 |
| ENSG00000003147 | ICA1 | 2.406 | 0.949 | 2.534 | 0.0113 | 0.0311 |
| ENSG00000223573 | TINCR | 2.405 | 0.59 | 4.08 | 0 | 0.0003 |
| ENSG00000213809 | KLRK1 | 2.405 | 0.889 | 2.706 | 0.0068 | 0.0206 |
| ENSG00000258599 |  | 2.401 | 0.984 | 2.44 | 0.0147 | 0.0387 |
| ENSG00000217702 |  | 2.4 | 0.647 | 3.712 | 0.0002 | 0.0011 |
| ENSG00000178338 | ZNF354B | 2.399 | 0.367 | 6.539 | 0 | 0 |
| ENSG00000162601 | MYSM1 | 2.399 | 0.271 | 8.842 | 0 | 0 |
| ENSG00000074966 | TXK | 2.399 | 0.504 | 4.756 | 0 | 0 |
| ENSG00000249125 |  | 2.399 | 0.767 | 3.128 | 0.0018 | 0.0066 |
| ENSG00000197498 | RPF2 | 2.397 | 0.297 | 8.068 | 0 | 0 |
| ENSG00000128284 | APOL3 | 2.395 | 0.453 | 5.282 | 0 | 0 |
| ENSG00000197584 | KCNMB2 | 2.395 | 0.812 | 2.95 | 0.0032 | 0.0108 |
| ENSG00000101222 | SPEF1 | 2.393 | 0.427 | 5.608 | 0 | 0 |
| ENSG00000165682 | CLEC1B | 2.393 | 1.026 | 2.332 | 0.0197 | 0.0493 |
| ENSG00000166946 | CCNDBP1 | 2.392 | 0.328 | 7.292 | 0 | 0 |
| ENSG00000240602 | AADACP1 | 2.392 | 0.757 | 3.162 | 0.0016 | 0.006 |
| ENSG00000254479 |  | 2.39 | 0.427 | 5.598 | 0 | 0 |
| ENSG00000132554 | RGS22 | 2.389 | 0.783 | 3.052 | 0.0023 | 0.0081 |
| ENSG00000260711 |  | 2.389 | 0.983 | 2.43 | 0.0151 | 0.0397 |
| ENSG00000248115 |  | 2.388 | 1.017 | 2.349 | 0.0188 | 0.0475 |
| ENSG00000213903 | LTB4R | 2.387 | 0.325 | 7.347 | 0 | 0 |
| ENSG00000164309 | CMYA5 | 2.385 | 0.365 | 6.529 | 0 | 0 |
| ENSG00000231882 | F10-AS1 | 2.385 | 0.823 | 2.898 | 0.0038 | 0.0125 |
| ENSG00000147533 | GOLGA7 | 2.384 | 0.267 | 8.932 | 0 | 0 |
| ENSG00000107831 | FGF8 | 2.381 | 0.407 | 5.848 | 0 | 0 |
| ENSG00000169957 | ZNF768 | 2.376 | 0.292 | 8.129 | 0 | 0 |
| ENSG00000165606 | DRGX | 2.376 | 0.737 | 3.222 | 0.0013 | 0.005 |
| ENSG00000023892 | DEF6 | 2.375 | 0.827 | 2.872 | 0.0041 | 0.0134 |
| ENSG00000163219 | ARHGAP25 | 2.375 | 0.864 | 2.75 | 0.006 | 0.0184 |
| ENSG00000161551 | ZNF577 | 2.374 | 0.638 | 3.723 | 0.0002 | 0.001 |
| ENSG00000234210 | LOC101927914 | 2.371 | 0.405 | 5.861 | 0 | 0 |
| ENSG00000075035 | WSCD2 | 2.369 | 0.435 | 5.442 | 0 | 0 |
| ENSG00000164325 | TMEM174 | 2.368 | 0.891 | 2.659 | 0.0078 | 0.0231 |
| ENSG00000242107 |  | 2.368 | 0.988 | 2.396 | 0.0166 | 0.0428 |
| ENSG00000174600 | CMKLR1 | 2.367 | 0.303 | 7.805 | 0 | 0 |
| ENSG00000156535 | CD109 | 2.365 | 0.314 | 7.531 | 0 | 0 |
| ENSG00000283232 | CYP2C23P | 2.365 | 1.007 | 2.349 | 0.0188 | 0.0475 |
| ENSG00000113638 | TTC33 | 2.364 | 0.327 | 7.227 | 0 | 0 |
| ENSG00000233499 |  | 2.364 | 0.659 | 3.59 | 0.0003 | 0.0016 |
| ENSG00000122728 | TAF1L | 2.362 | 0.737 | 3.205 | 0.0014 | 0.0053 |
| ENSG00000088448 | ANKRD10 | 2.36 | 0.243 | 9.696 | 0 | 0 |
| ENSG00000259000 | DOCK11P1 | 2.356 | 0.654 | 3.601 | 0.0003 | 0.0015 |
| ENSG00000185298 | CCDC137 | 2.355 | 0.277 | 8.511 | 0 | 0 |
| ENSG00000189134 | NKAPL | 2.355 | 0.439 | 5.367 | 0 | 0 |
| ENSG00000254423 | LOC100421094 | 2.355 | 0.79 | 2.98 | 0.0029 | 0.01 |
| ENSG00000122386 | ZNF205 | 2.352 | 0.318 | 7.394 | 0 | 0 |
| ENSG00000271254 | LOC102724250 | 2.35 | 0.34 | 6.92 | 0 | 0 |
| ENSG00000141469 | SLC14A1 | 2.346 | 0.343 | 6.835 | 0 | 0 |
| ENSG00000162825 | NBPF20 | 2.345 | 0.316 | 7.425 | 0 | 0 |
| ENSG00000239473 | RPL7P38 | 2.345 | 0.936 | 2.505 | 0.0122 | 0.0332 |
| ENSG00000175267 | VWA3A | 2.339 | 0.577 | 4.05 | 0.0001 | 0.0003 |
| ENSG00000196793 | ZNF239 | 2.334 | 0.314 | 7.422 | 0 | 0 |
| ENSG00000185792 | NLRP9 | 2.334 | 0.672 | 3.473 | 0.0005 | 0.0023 |
| ENSG00000272966 |  | 2.33 | 0.677 | 3.443 | 0.0006 | 0.0026 |
| ENSG00000276533 |  | 2.33 | 0.922 | 2.527 | 0.0115 | 0.0316 |
| ENSG00000095587 | TLL2 | 2.327 | 0.445 | 5.224 | 0 | 0 |
| ENSG00000181634 | TNFSF15 | 2.327 | 0.633 | 3.676 | 0.0002 | 0.0012 |
| ENSG00000184507 | NUTM1 | 2.322 | 0.861 | 2.697 | 0.007 | 0.021 |
| ENSG00000139572 | GPR84 | 2.32 | 0.449 | 5.164 | 0 | 0 |
| ENSG00000177511 | ST8SIA3 | 2.32 | 0.953 | 2.434 | 0.0149 | 0.0393 |
| ENSG00000107742 | SPOCK2 | 2.318 | 0.475 | 4.883 | 0 | 0 |
| ENSG00000188687 | SLC4A5 | 2.317 | 0.745 | 3.111 | 0.0019 | 0.0069 |
| ENSG00000254064 | LOC100507071 | 2.311 | 0.789 | 2.928 | 0.0034 | 0.0115 |
| ENSG00000279551 |  | 2.311 | 0.819 | 2.821 | 0.0048 | 0.0153 |
| ENSG00000173175 | ADCY5 | 2.31 | 0.635 | 3.638 | 0.0003 | 0.0014 |
| ENSG00000170498 | KISS1 | 2.306 | 0.526 | 4.384 | 0 | 0.0001 |
| ENSG00000131966 | ACTR10 | 2.305 | 0.304 | 7.59 | 0 | 0 |
| ENSG00000003137 | CYP26B1 | 2.305 | 0.649 | 3.554 | 0.0004 | 0.0018 |
| ENSG00000125879 | OTOR | 2.301 | 0.725 | 3.172 | 0.0015 | 0.0058 |
| ENSG00000269069 |  | 2.299 | 0.563 | 4.086 | 0 | 0.0003 |
| ENSG00000236404 | VLDLR-AS1 | 2.299 | 0.905 | 2.541 | 0.0111 | 0.0307 |
| ENSG00000134545 | KLRC1 | 2.298 | 0.44 | 5.219 | 0 | 0 |
| ENSG00000204227 | RING1 | 2.297 | 0.329 | 6.984 | 0 | 0 |
| ENSG00000158987 | RAPGEF6 | 2.297 | 0.348 | 6.606 | 0 | 0 |
| ENSG00000251441 | RTEL1P1 | 2.297 | 0.657 | 3.499 | 0.0005 | 0.0021 |
| ENSG00000182965 |  | 2.295 | 0.639 | 3.59 | 0.0003 | 0.0016 |
| ENSG00000272486 |  | 2.295 | 0.749 | 3.066 | 0.0022 | 0.0078 |
| ENSG00000187416 | LHFPL3 | 2.294 | 0.834 | 2.752 | 0.0059 | 0.0184 |
| ENSG00000175105 | ZNF654 | 2.292 | 0.278 | 8.253 | 0 | 0 |
| ENSG00000226121 |  | 2.288 | 0.78 | 2.933 | 0.0034 | 0.0114 |
| ENSG00000090539 | CHRD | 2.287 | 0.435 | 5.253 | 0 | 0 |
| ENSG00000204262 | COL5A2 | 2.287 | 0.279 | 8.195 | 0 | 0 |
| ENSG00000163794 | UCN | 2.287 | 0.57 | 4.009 | 0.0001 | 0.0004 |
| ENSG00000110169 | HPX | 2.286 | 0.46 | 4.964 | 0 | 0 |
| ENSG00000250329 |  | 2.286 | 0.717 | 3.188 | 0.0014 | 0.0055 |
| ENSG00000068976 | PYGM | 2.283 | 0.29 | 7.883 | 0 | 0 |
| ENSG00000171931 | FBXW10 | 2.283 | 0.914 | 2.497 | 0.0125 | 0.0339 |
| ENSG00000163346 | PBXIP1 | 2.279 | 0.278 | 8.193 | 0 | 0 |
| ENSG00000108448 | TRIM16L | 2.279 | 0.394 | 5.777 | 0 | 0 |
| ENSG00000272054 |  | 2.279 | 0.44 | 5.179 | 0 | 0 |
| ENSG00000234420 | ZNF37BP | 2.276 | 0.329 | 6.909 | 0 | 0 |
| ENSG00000274214 |  | 2.275 | 0.904 | 2.517 | 0.0118 | 0.0324 |
| ENSG00000234944 | LINC02623 | 2.274 | 0.82 | 2.774 | 0.0055 | 0.0173 |
| ENSG00000225138 |  | 2.273 | 0.298 | 7.622 | 0 | 0 |
| ENSG00000259186 | MRPS15P1 | 2.272 | 0.865 | 2.628 | 0.0086 | 0.0249 |
| ENSG00000251463 |  | 2.27 | 0.701 | 3.237 | 0.0012 | 0.0048 |
| ENSG00000143520 | FLG2 | 2.27 | 0.729 | 3.114 | 0.0018 | 0.0068 |
| ENSG00000255031 |  | 2.27 | 0.735 | 3.089 | 0.002 | 0.0073 |
| ENSG00000176076 | KCNE5 | 2.269 | 0.576 | 3.937 | 0.0001 | 0.0005 |
| ENSG00000197016 | ZNF470 | 2.267 | 0.278 | 8.157 | 0 | 0 |
| ENSG00000264954 | PRR29-AS1 | 2.262 | 0.529 | 4.277 | 0 | 0.0001 |
| ENSG00000159314 | ARHGAP27 | 2.26 | 0.337 | 6.698 | 0 | 0 |
| ENSG00000228526 | MIR34AHG | 2.259 | 0.22 | 10.288 | 0 | 0 |
| ENSG00000172243 | CLEC7A | 2.259 | 0.753 | 3.001 | 0.0027 | 0.0094 |
| ENSG00000260865 |  | 2.258 | 0.927 | 2.436 | 0.0149 | 0.0391 |
| ENSG00000181404 | WASHC1 | 2.257 | 0.534 | 4.228 | 0 | 0.0002 |
| ENSG00000180353 | HCLS1 | 2.256 | 0.331 | 6.809 | 0 | 0 |
| ENSG00000081148 | IMPG2 | 2.256 | 0.564 | 4.001 | 0.0001 | 0.0004 |
| ENSG00000168928 | CTRB2 | 2.254 | 0.611 | 3.69 | 0.0002 | 0.0011 |
| ENSG00000277462 | ZNF670 | 2.253 | 0.353 | 6.387 | 0 | 0 |
| ENSG00000227474 | RPL6P24 | 2.252 | 0.584 | 3.855 | 0.0001 | 0.0006 |
| ENSG00000137460 | FHDC1 | 2.25 | 0.423 | 5.317 | 0 | 0 |
| ENSG00000165195 | PIGA | 2.244 | 0.42 | 5.35 | 0 | 0 |
| ENSG00000069493 | CLEC2D | 2.244 | 0.586 | 3.832 | 0.0001 | 0.0007 |
| ENSG00000109063 | MYH3 | 2.24 | 0.256 | 8.734 | 0 | 0 |
| ENSG00000253506 | NACA2 | 2.238 | 0.487 | 4.596 | 0 | 0 |
| ENSG00000230978 | LINC00160 | 2.238 | 0.575 | 3.891 | 0.0001 | 0.0006 |
| ENSG00000023734 | STRAP | 2.235 | 0.343 | 6.518 | 0 | 0 |
| ENSG00000175820 | CCDC168 | 2.234 | 0.312 | 7.17 | 0 | 0 |
| ENSG00000121075 | TBX4 | 2.233 | 0.531 | 4.203 | 0 | 0.0002 |
| ENSG00000267634 |  | 2.23 | 0.911 | 2.448 | 0.0144 | 0.038 |
| ENSG00000167733 | HSD11B1L | 2.229 | 0.347 | 6.429 | 0 | 0 |
| ENSG00000138031 | ADCY3 | 2.228 | 0.394 | 5.653 | 0 | 0 |
| ENSG00000241563 | CORT | 2.228 | 0.497 | 4.486 | 0 | 0.0001 |
| ENSG00000153495 | TEX29 | 2.226 | 0.454 | 4.899 | 0 | 0 |
| ENSG00000183423 | LRIT3 | 2.226 | 0.36 | 6.181 | 0 | 0 |
| ENSG00000213940 | RPL13AP13 | 2.225 | 0.714 | 3.115 | 0.0018 | 0.0068 |
| ENSG00000154485 | MMP21 | 2.225 | 0.862 | 2.581 | 0.0099 | 0.0279 |
| ENSG00000264553 | MIR4257 | 2.223 | 0.808 | 2.752 | 0.0059 | 0.0184 |
| ENSG00000198954 | KIFBP | 2.222 | 0.227 | 9.766 | 0 | 0 |
| ENSG00000115593 | SMYD1 | 2.222 | 0.945 | 2.353 | 0.0186 | 0.0471 |
| ENSG00000162086 | ZNF75A | 2.22 | 0.257 | 8.627 | 0 | 0 |
| ENSG00000171747 | LGALS4 | 2.22 | 0.362 | 6.138 | 0 | 0 |
| ENSG00000165471 | MBL2 | 2.217 | 0.892 | 2.485 | 0.013 | 0.0349 |
| ENSG00000261488 | TBILA | 2.216 | 0.703 | 3.152 | 0.0016 | 0.0061 |
| ENSG00000196263 | ZNF471 | 2.215 | 0.422 | 5.252 | 0 | 0 |
| ENSG00000204403 | CASP12 | 2.215 | 0.525 | 4.223 | 0 | 0.0002 |
| ENSG00000168621 | GDNF | 2.213 | 0.266 | 8.309 | 0 | 0 |
| ENSG00000132664 | POLR3F | 2.212 | 0.325 | 6.798 | 0 | 0 |
| ENSG00000149256 | TENM4 | 2.212 | 0.9 | 2.458 | 0.014 | 0.0371 |
| ENSG00000236255 |  | 2.21 | 0.322 | 6.862 | 0 | 0 |
| ENSG00000171517 | LPAR3 | 2.209 | 0.556 | 3.976 | 0.0001 | 0.0004 |
| ENSG00000183090 | FREM3 | 2.207 | 0.727 | 3.037 | 0.0024 | 0.0085 |
| ENSG00000279570 |  | 2.207 | 0.882 | 2.503 | 0.0123 | 0.0334 |
| ENSG00000249661 |  | 2.204 | 0.63 | 3.5 | 0.0005 | 0.0021 |
| ENSG00000227799 |  | 2.204 | 0.7 | 3.151 | 0.0016 | 0.0062 |
| ENSG00000134812 | CBLIF | 2.204 | 0.741 | 2.976 | 0.0029 | 0.0101 |
| ENSG00000260032 | NORAD | 2.202 | 0.235 | 9.387 | 0 | 0 |
| ENSG00000235081 |  | 2.202 | 0.886 | 2.486 | 0.0129 | 0.0348 |
| ENSG00000163006 | CCDC138 | 2.201 | 0.351 | 6.264 | 0 | 0 |
| ENSG00000163510 | CWC22 | 2.2 | 0.255 | 8.628 | 0 | 0 |
| ENSG00000124145 | SDC4 | 2.2 | 0.35 | 6.278 | 0 | 0 |
| ENSG00000204149 | AGAP6 | 2.2 | 0.284 | 7.739 | 0 | 0 |
| ENSG00000167941 | SOST | 2.2 | 0.523 | 4.202 | 0 | 0.0002 |
| ENSG00000249215 |  | 2.199 | 0.605 | 3.634 | 0.0003 | 0.0014 |
| ENSG00000166257 | SCN3B | 2.198 | 0.76 | 2.892 | 0.0038 | 0.0127 |
| ENSG00000279311 |  | 2.198 | 0.771 | 2.849 | 0.0044 | 0.0142 |
| ENSG00000175352 | NRIP3 | 2.197 | 0.306 | 7.172 | 0 | 0 |
| ENSG00000276517 |  | 2.197 | 0.563 | 3.902 | 0.0001 | 0.0005 |
| ENSG00000174827 | PDZK1 | 2.196 | 0.632 | 3.477 | 0.0005 | 0.0023 |
| ENSG00000136895 | GARNL3 | 2.195 | 0.566 | 3.881 | 0.0001 | 0.0006 |
| ENSG00000120215 | MLANA | 2.194 | 0.759 | 2.892 | 0.0038 | 0.0127 |
| ENSG00000254213 |  | 2.193 | 0.326 | 6.736 | 0 | 0 |
| ENSG00000092203 | TOX4 | 2.193 | 0.278 | 7.876 | 0 | 0 |
| ENSG00000181929 | PRKAG1 | 2.193 | 0.323 | 6.792 | 0 | 0 |
| ENSG00000226445 |  | 2.193 | 0.565 | 3.879 | 0.0001 | 0.0006 |
| ENSG00000266469 |  | 2.19 | 0.355 | 6.177 | 0 | 0 |
| ENSG00000249485 |  | 2.189 | 0.756 | 2.897 | 0.0038 | 0.0125 |
| ENSG00000243777 | RPS6P17 | 2.189 | 0.777 | 2.817 | 0.0048 | 0.0155 |
| ENSG00000187553 | CYP26C1 | 2.188 | 0.855 | 2.558 | 0.0105 | 0.0294 |
| ENSG00000105146 | AURKC | 2.187 | 0.486 | 4.502 | 0 | 0.0001 |
| ENSG00000079335 | CDC14A | 2.187 | 0.672 | 3.252 | 0.0011 | 0.0046 |
| ENSG00000244009 |  | 2.186 | 0.593 | 3.685 | 0.0002 | 0.0012 |
| ENSG00000188234 | AGAP4 | 2.186 | 0.814 | 2.686 | 0.0072 | 0.0216 |
| ENSG00000166261 | ZNF202 | 2.181 | 0.257 | 8.496 | 0 | 0 |
| ENSG00000175003 | SLC22A1 | 2.181 | 0.341 | 6.399 | 0 | 0 |
| ENSG00000265296 | FEM1AP2 | 2.181 | 0.716 | 3.047 | 0.0023 | 0.0082 |
| ENSG00000173261 | PLAC8L1 | 2.178 | 0.402 | 5.424 | 0 | 0 |
| ENSG00000157703 | SVOPL | 2.178 | 0.594 | 3.668 | 0.0002 | 0.0012 |
| ENSG00000267620 |  | 2.178 | 0.662 | 3.29 | 0.001 | 0.0041 |
| ENSG00000283511 |  | 2.176 | 0.668 | 3.257 | 0.0011 | 0.0045 |
| ENSG00000161905 | ALOX15 | 2.175 | 0.82 | 2.653 | 0.008 | 0.0235 |
| ENSG00000261786 |  | 2.174 | 0.633 | 3.434 | 0.0006 | 0.0026 |
| ENSG00000136383 | ALPK3 | 2.174 | 0.887 | 2.451 | 0.0143 | 0.0378 |
| ENSG00000241764 | LOC101927974 | 2.173 | 0.538 | 4.037 | 0.0001 | 0.0003 |
| ENSG00000114805 | PLCH1 | 2.168 | 0.589 | 3.683 | 0.0002 | 0.0012 |
| ENSG00000235609 |  | 2.167 | 0.69 | 3.139 | 0.0017 | 0.0064 |
| ENSG00000104756 | KCTD9 | 2.166 | 0.239 | 9.066 | 0 | 0 |
| ENSG00000165181 | SHOC1 | 2.166 | 0.641 | 3.381 | 0.0007 | 0.0031 |
| ENSG00000251095 |  | 2.166 | 0.759 | 2.854 | 0.0043 | 0.0141 |
| ENSG00000155761 | SPAG17 | 2.164 | 0.686 | 3.153 | 0.0016 | 0.0061 |
| ENSG00000278974 |  | 2.164 | 0.817 | 2.647 | 0.0081 | 0.0238 |
| ENSG00000240021 | TEX35 | 2.163 | 0.438 | 4.942 | 0 | 0 |
| ENSG00000282419 | TEX13D | 2.163 | 0.91 | 2.376 | 0.0175 | 0.0448 |
| ENSG00000214814 | FER1L6 | 2.157 | 0.411 | 5.249 | 0 | 0 |
| ENSG00000120471 | TP53AIP1 | 2.156 | 0.786 | 2.742 | 0.0061 | 0.0188 |
| ENSG00000213590 |  | 2.156 | 0.842 | 2.56 | 0.0105 | 0.0293 |
| ENSG00000145908 | ZNF300 | 2.155 | 0.263 | 8.201 | 0 | 0 |
| ENSG00000204519 | ZNF551 | 2.152 | 0.263 | 8.185 | 0 | 0 |
| ENSG00000084652 | TXLNA | 2.15 | 0.226 | 9.513 | 0 | 0 |
| ENSG00000186184 | POLR1D | 2.147 | 0.271 | 7.915 | 0 | 0 |
| ENSG00000144820 | ADGRG7 | 2.146 | 0.663 | 3.234 | 0.0012 | 0.0048 |
| ENSG00000257543 |  | 2.144 | 0.509 | 4.212 | 0 | 0.0002 |
| ENSG00000184492 | FOXD4L1 | 2.143 | 0.331 | 6.467 | 0 | 0 |
| ENSG00000128059 | PPAT | 2.14 | 0.311 | 6.877 | 0 | 0 |
| ENSG00000260910 |  | 2.14 | 0.429 | 4.983 | 0 | 0 |
| ENSG00000229570 | GAPDHP58 | 2.14 | 0.884 | 2.42 | 0.0155 | 0.0405 |
| ENSG00000270231 | NBPF8 | 2.139 | 0.317 | 6.744 | 0 | 0 |
| ENSG00000115155 | OTOF | 2.138 | 0.652 | 3.28 | 0.001 | 0.0042 |
| ENSG00000162849 | KIF26B | 2.138 | 0.854 | 2.504 | 0.0123 | 0.0334 |
| ENSG00000126001 | CEP250 | 2.137 | 0.276 | 7.745 | 0 | 0 |
| ENSG00000276523 |  | 2.137 | 0.494 | 4.326 | 0 | 0.0001 |
| ENSG00000250292 |  | 2.137 | 0.727 | 2.94 | 0.0033 | 0.0111 |
| ENSG00000257281 | LOC196469 | 2.135 | 0.55 | 3.881 | 0.0001 | 0.0006 |
| ENSG00000260918 |  | 2.135 | 0.78 | 2.737 | 0.0062 | 0.019 |
| ENSG00000144029 | MRPS5 | 2.133 | 0.336 | 6.347 | 0 | 0 |
| ENSG00000188725 | SMIM15 | 2.133 | 0.317 | 6.728 | 0 | 0 |
| ENSG00000116852 | KIF21B | 2.132 | 0.576 | 3.7 | 0.0002 | 0.0011 |
| ENSG00000167840 | ZNF232 | 2.13 | 0.297 | 7.173 | 0 | 0 |
| ENSG00000137942 | FNBP1L | 2.13 | 0.515 | 4.136 | 0 | 0.0002 |
| ENSG00000278879 |  | 2.13 | 0.589 | 3.616 | 0.0003 | 0.0015 |
| ENSG00000164707 | SLC13A4 | 2.128 | 0.26 | 8.17 | 0 | 0 |
| ENSG00000278570 | NR2E3 | 2.128 | 0.679 | 3.131 | 0.0017 | 0.0065 |
| ENSG00000130948 | HSD17B3 | 2.128 | 0.831 | 2.56 | 0.0105 | 0.0293 |
| ENSG00000136881 | BAAT | 2.126 | 0.4 | 5.315 | 0 | 0 |
| ENSG00000159915 | ZNF233 | 2.125 | 0.434 | 4.901 | 0 | 0 |
| ENSG00000091542 | ALKBH5 | 2.124 | 0.286 | 7.416 | 0 | 0 |
| ENSG00000059573 | ALDH18A1 | 2.122 | 0.38 | 5.579 | 0 | 0 |
| ENSG00000144488 | ESPNL | 2.121 | 0.444 | 4.774 | 0 | 0 |
| ENSG00000076662 | ICAM3 | 2.117 | 0.722 | 2.93 | 0.0034 | 0.0114 |
| ENSG00000123329 | ARHGAP9 | 2.116 | 0.376 | 5.622 | 0 | 0 |
| ENSG00000254973 |  | 2.116 | 0.856 | 2.473 | 0.0134 | 0.0358 |
| ENSG00000007314 | SCN4A | 2.115 | 0.319 | 6.634 | 0 | 0 |
| ENSG00000213139 | CRYGS | 2.114 | 0.758 | 2.789 | 0.0053 | 0.0167 |
| ENSG00000067048 | DDX3Y | 2.113 | 0.272 | 7.759 | 0 | 0 |
| ENSG00000254635 |  | 2.113 | 0.327 | 6.457 | 0 | 0 |
| ENSG00000233625 |  | 2.113 | 0.904 | 2.338 | 0.0194 | 0.0487 |
| ENSG00000117862 | TXNDC12 | 2.111 | 0.27 | 7.812 | 0 | 0 |
| ENSG00000243742 | RPLP0P2 | 2.111 | 0.42 | 5.027 | 0 | 0 |
| ENSG00000227401 | RPL37P1 | 2.111 | 0.695 | 3.038 | 0.0024 | 0.0085 |
| ENSG00000109667 | SLC2A9 | 2.108 | 0.452 | 4.666 | 0 | 0 |
| ENSG00000157005 | SST | 2.108 | 0.84 | 2.508 | 0.0121 | 0.033 |
| ENSG00000112599 | GUCA1B | 2.107 | 0.683 | 3.087 | 0.002 | 0.0074 |
| ENSG00000163082 | SGPP2 | 2.106 | 0.671 | 3.137 | 0.0017 | 0.0064 |
| ENSG00000224831 |  | 2.104 | 0.899 | 2.341 | 0.0192 | 0.0483 |
| ENSG00000172232 | AZU1 | 2.103 | 0.394 | 5.333 | 0 | 0 |
| ENSG00000197128 | ZNF772 | 2.103 | 0.368 | 5.723 | 0 | 0 |
| ENSG00000179532 | DNHD1 | 2.103 | 0.343 | 6.129 | 0 | 0 |
| ENSG00000163684 | RPP14 | 2.102 | 0.298 | 7.045 | 0 | 0 |
| ENSG00000184811 | TRARG1 | 2.101 | 0.439 | 4.79 | 0 | 0 |
| ENSG00000112706 | IMPG1 | 2.1 | 0.655 | 3.207 | 0.0013 | 0.0053 |
| ENSG00000274286 | ADRA2B | 2.1 | 0.723 | 2.903 | 0.0037 | 0.0123 |
| ENSG00000273489 |  | 2.1 | 0.877 | 2.393 | 0.0167 | 0.0431 |
| ENSG00000148483 | TMEM236 | 2.097 | 0.432 | 4.858 | 0 | 0 |
| ENSG00000183128 | CALHM3 | 2.096 | 0.813 | 2.578 | 0.0099 | 0.0281 |
| ENSG00000223599 |  | 2.094 | 0.569 | 3.684 | 0.0002 | 0.0012 |
| ENSG00000260276 |  | 2.094 | 0.739 | 2.832 | 0.0046 | 0.0149 |
| ENSG00000253223 | LOC100288001 | 2.094 | 0.891 | 2.35 | 0.0188 | 0.0474 |
| ENSG00000134910 | STT3A | 2.092 | 0.258 | 8.119 | 0 | 0 |
| ENSG00000174564 | IL20RB | 2.092 | 0.453 | 4.616 | 0 | 0 |
| ENSG00000205089 | CCNI2 | 2.091 | 0.614 | 3.407 | 0.0007 | 0.0029 |
| ENSG00000132840 | BHMT2 | 2.091 | 0.636 | 3.287 | 0.001 | 0.0041 |
| ENSG00000147873 | IFNA5 | 2.091 | 0.664 | 3.151 | 0.0016 | 0.0061 |
| ENSG00000140107 | SLC25A47 | 2.09 | 0.458 | 4.565 | 0 | 0 |
| ENSG00000150687 | PRSS23 | 2.088 | 0.291 | 7.169 | 0 | 0 |
| ENSG00000183666 | GUSBP1 | 2.085 | 0.394 | 5.294 | 0 | 0 |
| ENSG00000085662 | AKR1B1 | 2.084 | 0.257 | 8.123 | 0 | 0 |
| ENSG00000234155 | LINC02535 | 2.083 | 0.429 | 4.849 | 0 | 0 |
| ENSG00000255423 | EBLN2 | 2.083 | 0.572 | 3.645 | 0.0003 | 0.0013 |
| ENSG00000271086 | NAMA | 2.083 | 0.865 | 2.407 | 0.0161 | 0.0417 |
| ENSG00000234773 |  | 2.082 | 0.446 | 4.673 | 0 | 0 |
| ENSG00000139287 | TPH2 | 2.08 | 0.33 | 6.298 | 0 | 0 |
| ENSG00000205038 | PKHD1L1 | 2.08 | 0.883 | 2.355 | 0.0185 | 0.0469 |
| ENSG00000184999 | SLC22A10 | 2.079 | 0.722 | 2.878 | 0.004 | 0.0132 |
| ENSG00000249306 | LINC01411 | 2.079 | 0.816 | 2.547 | 0.0109 | 0.0302 |
| ENSG00000182872 | RBM10 | 2.076 | 0.267 | 7.777 | 0 | 0 |
| ENSG00000238205 | MPC1L | 2.074 | 0.442 | 4.695 | 0 | 0 |
| ENSG00000280254 |  | 2.074 | 0.539 | 3.849 | 0.0001 | 0.0007 |
| ENSG00000235020 |  | 2.074 | 0.756 | 2.745 | 0.0061 | 0.0187 |
| ENSG00000124157 | SEMG2 | 2.073 | 0.619 | 3.351 | 0.0008 | 0.0034 |
| ENSG00000283236 |  | 2.073 | 0.734 | 2.823 | 0.0048 | 0.0153 |
| ENSG00000237438 |  | 2.073 | 0.736 | 2.815 | 0.0049 | 0.0156 |
| ENSG00000132361 | CLUH | 2.072 | 0.239 | 8.657 | 0 | 0 |
| ENSG00000182324 | KCNJ14 | 2.072 | 0.387 | 5.347 | 0 | 0 |
| ENSG00000253943 | KRT18P37 | 2.072 | 0.46 | 4.507 | 0 | 0.0001 |
| ENSG00000270885 | RASL10B | 2.071 | 0.456 | 4.54 | 0 | 0 |
| ENSG00000141098 | GFOD2 | 2.07 | 0.308 | 6.716 | 0 | 0 |
| ENSG00000122861 | PLAU | 2.07 | 0.333 | 6.219 | 0 | 0 |
| ENSG00000226650 | KIF4B | 2.07 | 0.49 | 4.227 | 0 | 0.0002 |
| ENSG00000230071 | RPL4P6 | 2.07 | 0.69 | 2.997 | 0.0027 | 0.0095 |
| ENSG00000218418 |  | 2.07 | 0.814 | 2.544 | 0.0109 | 0.0304 |
| ENSG00000230262 | LINC02603 | 2.068 | 0.609 | 3.393 | 0.0007 | 0.003 |
| ENSG00000140284 | SLC27A2 | 2.067 | 0.745 | 2.774 | 0.0055 | 0.0174 |
| ENSG00000095485 | CWF19L1 | 2.066 | 0.244 | 8.477 | 0 | 0 |
| ENSG00000142224 | IL19 | 2.065 | 0.759 | 2.722 | 0.0065 | 0.0198 |
| ENSG00000204611 | ZNF616 | 2.061 | 0.28 | 7.357 | 0 | 0 |
| ENSG00000083067 | TRPM3 | 2.06 | 0.437 | 4.712 | 0 | 0 |
| ENSG00000146409 | SLC18B1 | 2.06 | 0.512 | 4.025 | 0.0001 | 0.0003 |
| ENSG00000163207 | IVL | 2.058 | 0.693 | 2.971 | 0.003 | 0.0102 |
| ENSG00000115590 | IL1R2 | 2.057 | 0.647 | 3.18 | 0.0015 | 0.0057 |
| ENSG00000151414 | NEK7 | 2.056 | 0.327 | 6.294 | 0 | 0 |
| ENSG00000069696 | DRD4 | 2.054 | 0.548 | 3.751 | 0.0002 | 0.0009 |
| ENSG00000167100 | SAMD14 | 2.053 | 0.545 | 3.766 | 0.0002 | 0.0009 |
| ENSG00000248586 | LOC100288510 | 2.052 | 0.656 | 3.127 | 0.0018 | 0.0066 |
| ENSG00000139973 | SYT16 | 2.05 | 0.288 | 7.11 | 0 | 0 |
| ENSG00000164823 | OSGIN2 | 2.05 | 0.284 | 7.224 | 0 | 0 |
| ENSG00000198812 | LRRC10 | 2.05 | 0.362 | 5.662 | 0 | 0 |
| ENSG00000105219 | CCNP | 2.05 | 0.543 | 3.775 | 0.0002 | 0.0008 |
| ENSG00000270427 |  | 2.05 | 0.806 | 2.545 | 0.0109 | 0.0304 |
| ENSG00000151320 | AKAP6 | 2.049 | 0.531 | 3.858 | 0.0001 | 0.0006 |
| ENSG00000145365 | TIFA | 2.048 | 0.475 | 4.312 | 0 | 0.0001 |
| ENSG00000084110 | HAL | 2.048 | 0.865 | 2.366 | 0.018 | 0.0457 |
| ENSG00000250796 |  | 2.046 | 0.567 | 3.605 | 0.0003 | 0.0015 |
| ENSG00000102174 | PHEX | 2.044 | 0.709 | 2.883 | 0.0039 | 0.013 |
| ENSG00000255883 |  | 2.043 | 0.488 | 4.186 | 0 | 0.0002 |
| ENSG00000078114 | NEBL | 2.043 | 0.808 | 2.53 | 0.0114 | 0.0314 |
| ENSG00000169071 | ROR2 | 2.042 | 0.348 | 5.863 | 0 | 0 |
| ENSG00000178429 | RPS3AP5 | 2.041 | 0.712 | 2.869 | 0.0041 | 0.0135 |
| ENSG00000111364 | DDX55 | 2.039 | 0.258 | 7.899 | 0 | 0 |
| ENSG00000123243 | ITIH5 | 2.039 | 0.421 | 4.838 | 0 | 0 |
| ENSG00000241458 | RPL7P19 | 2.038 | 0.418 | 4.875 | 0 | 0 |
| ENSG00000270978 | GLULP1 | 2.037 | 0.523 | 3.896 | 0.0001 | 0.0006 |
| ENSG00000178038 | ALS2CL | 2.036 | 0.495 | 4.115 | 0 | 0.0002 |
| ENSG00000134249 | ADAM30 | 2.035 | 0.377 | 5.403 | 0 | 0 |
| ENSG00000226396 |  | 2.033 | 0.444 | 4.579 | 0 | 0 |
| ENSG00000071967 | CYBRD1 | 2.033 | 0.258 | 7.885 | 0 | 0 |
| ENSG00000197362 | ZNF786 | 2.033 | 0.464 | 4.383 | 0 | 0.0001 |
| ENSG00000275367 |  | 2.033 | 0.501 | 4.057 | 0 | 0.0003 |
| ENSG00000225509 |  | 2.031 | 0.501 | 4.057 | 0 | 0.0003 |
| ENSG00000174899 | SLC66A1L | 2.029 | 0.468 | 4.333 | 0 | 0.0001 |
| ENSG00000256995 |  | 2.029 | 0.542 | 3.747 | 0.0002 | 0.0009 |
| ENSG00000137275 | RIPK1 | 2.028 | 0.226 | 8.964 | 0 | 0 |
| ENSG00000114268 | PFKFB4 | 2.027 | 0.269 | 7.526 | 0 | 0 |
| ENSG00000080815 | PSEN1 | 2.026 | 0.226 | 8.945 | 0 | 0 |
| ENSG00000119946 | CNNM1 | 2.026 | 0.445 | 4.554 | 0 | 0 |
| ENSG00000183091 | NEB | 2.026 | 0.245 | 8.27 | 0 | 0 |
| ENSG00000154678 | PDE1C | 2.026 | 0.655 | 3.095 | 0.002 | 0.0072 |
| ENSG00000120658 | ENOX1 | 2.024 | 0.641 | 3.156 | 0.0016 | 0.0061 |
| ENSG00000205221 | VIT | 2.023 | 0.373 | 5.427 | 0 | 0 |
| ENSG00000178691 | SUZ12 | 2.023 | 0.251 | 8.047 | 0 | 0 |
| ENSG00000254017 | IGHEP2 | 2.022 | 0.394 | 5.138 | 0 | 0 |
| ENSG00000057468 | MSH4 | 2.022 | 0.531 | 3.807 | 0.0001 | 0.0008 |
| ENSG00000151012 | SLC7A11 | 2.019 | 0.302 | 6.689 | 0 | 0 |
| ENSG00000250526 | CCT6P2 | 2.019 | 0.855 | 2.361 | 0.0182 | 0.0463 |
| ENSG00000165923 | AGBL2 | 2.017 | 0.338 | 5.974 | 0 | 0 |
| ENSG00000168412 | MTNR1A | 2.016 | 0.626 | 3.221 | 0.0013 | 0.005 |
| ENSG00000168263 | KCNV2 | 2.016 | 0.685 | 2.944 | 0.0032 | 0.011 |
| ENSG00000091106 | NLRC4 | 2.015 | 0.506 | 3.98 | 0.0001 | 0.0004 |
| ENSG00000232756 |  | 2.015 | 0.752 | 2.679 | 0.0074 | 0.022 |
| ENSG00000162947 | LINC01931 | 2.015 | 0.769 | 2.621 | 0.0088 | 0.0253 |
| ENSG00000172939 | OXSR1 | 2.014 | 0.225 | 8.965 | 0 | 0 |
| ENSG00000081052 | COL4A4 | 2.013 | 0.764 | 2.634 | 0.0084 | 0.0245 |
| ENSG00000082074 | FYB1 | 2.012 | 0.487 | 4.131 | 0 | 0.0002 |
| ENSG00000261556 | SMG1P7 | 2.011 | 0.336 | 5.991 | 0 | 0 |
| ENSG00000137474 | MYO7A | 2.011 | 0.783 | 2.569 | 0.0102 | 0.0286 |
| ENSG00000239388 | ASB14 | 2.01 | 0.608 | 3.306 | 0.0009 | 0.0039 |
| ENSG00000166986 | MARS1 | 2.006 | 0.199 | 10.093 | 0 | 0 |
| ENSG00000236901 | MIR600HG | 2.006 | 0.412 | 4.869 | 0 | 0 |
| ENSG00000178386 | ZNF223 | 2.006 | 0.667 | 3.008 | 0.0026 | 0.0092 |
| ENSG00000147113 | DIPK2B | 2.005 | 0.4 | 5.016 | 0 | 0 |
| ENSG00000205838 | TTC23L | 2.005 | 0.563 | 3.558 | 0.0004 | 0.0018 |
| ENSG00000281026 | N4BP2L2-IT2 | 2.005 | 0.582 | 3.446 | 0.0006 | 0.0025 |
| ENSG00000247081 | LOC105369147 | 2.003 | 0.439 | 4.568 | 0 | 0 |
| ENSG00000048649 | RSF1 | 2.002 | 0.262 | 7.628 | 0 | 0 |

| **Appendix Table 5.** Differentially expressed genes after GapmeRs induced CDKN2B-AS1 knockdown(DEGs_down_log2FC<-1) | | | | | | |
| --- | --- | --- | --- | --- | --- | --- |
| **ENSEMBL ID** | **gene symbol** | **log2FoldChange (lgc)** | **standard error (lfc)** | **stat** | **pvalue** | **padj** |
| ENSG00000223635 | NUP133-DT | -6.618 | 1.043 | -6.346 | 0 | 0 |
| ENSG00000226665 | SSR1P2 | -6.366 | 1.152 | -5.526 | 0 | 0 |
| ENSG00000255317 |  | -5.662 | 0.544 | -10.405 | 0 | 0 |
| ENSG00000202415 |  | -5.587 | 1.373 | -4.069 | 0 | 0.0003 |
| ENSG00000267334 | KIF18B-DT | -5.528 | 0.7 | -7.902 | 0 | 0 |
| ENSG00000251888 | RN7SKP190 | -5.505 | 0.988 | -5.57 | 0 | 0 |
| ENSG00000272226 |  | -5.498 | 1.166 | -4.715 | 0 | 0 |
| ENSG00000246350 | ZBED9-AS1 | -5.471 | 0.342 | -15.987 | 0 | 0 |
| ENSG00000274460 |  | -5.447 | 0.462 | -11.78 | 0 | 0 |
| ENSG00000262890 |  | -5.445 | 0.526 | -10.348 | 0 | 0 |
| ENSG00000277511 |  | -5.413 | 0.358 | -15.123 | 0 | 0 |
| ENSG00000272366 |  | -5.397 | 1.266 | -4.263 | 0 | 0.0001 |
| ENSG00000220248 | ZNF402P | -5.289 | 1.224 | -4.319 | 0 | 0.0001 |
| ENSG00000254579 |  | -5.161 | 1.04 | -4.961 | 0 | 0 |
| ENSG00000232162 | USP12-AS1 | -5.141 | 1.3 | -3.955 | 0.0001 | 0.0005 |
| ENSG00000215464 |  | -5.1 | 1.495 | -3.412 | 0.0006 | 0.0028 |
| ENSG00000258153 |  | -5.084 | 0.793 | -6.411 | 0 | 0 |
| ENSG00000229835 | KHSRPP1 | -5.058 | 0.624 | -8.102 | 0 | 0 |
| ENSG00000283563 | ZCWPW2 | -5.019 | 1.266 | -3.966 | 0.0001 | 0.0004 |
| ENSG00000249781 | LINC02112 | -4.985 | 0.58 | -8.601 | 0 | 0 |
| ENSG00000258292 | LINC02410 | -4.965 | 1.077 | -4.608 | 0 | 0 |
| ENSG00000133101 | CCNA1 | -4.961 | 0.489 | -10.149 | 0 | 0 |
| ENSG00000235947 | EGOT | -4.915 | 1.195 | -4.112 | 0 | 0.0003 |
| ENSG00000274949 |  | -4.882 | 1.602 | -3.048 | 0.0023 | 0.0082 |
| ENSG00000229794 | MTCYBP32 | -4.878 | 1.185 | -4.115 | 0 | 0.0002 |
| ENSG00000230943 | LINC02541 | -4.853 | 1.284 | -3.78 | 0.0002 | 0.0008 |
| ENSG00000275763 | ZNF516-DT | -4.827 | 0.703 | -6.867 | 0 | 0 |
| ENSG00000270996 |  | -4.806 | 1.202 | -3.998 | 0.0001 | 0.0004 |
| ENSG00000224609 | HSD52 | -4.769 | 1.101 | -4.333 | 0 | 0.0001 |
| ENSG00000275989 |  | -4.685 | 0.753 | -6.223 | 0 | 0 |
| ENSG00000228109 | MELTF-AS1 | -4.67 | 0.84 | -5.562 | 0 | 0 |
| ENSG00000220875 | H3C9P | -4.62 | 0.477 | -9.685 | 0 | 0 |
| ENSG00000278916 | CEP83-DT | -4.599 | 0.572 | -8.043 | 0 | 0 |
| ENSG00000240790 |  | -4.591 | 1.249 | -3.674 | 0.0002 | 0.0012 |
| ENSG00000164621 | SMAD5-AS1 | -4.582 | 1.04 | -4.407 | 0 | 0.0001 |
| ENSG00000236444 | UBE2L5 | -4.561 | 1.012 | -4.509 | 0 | 0.0001 |
| ENSG00000171804 | WDR87 | -4.481 | 0.671 | -6.676 | 0 | 0 |
| ENSG00000279198 |  | -4.467 | 0.304 | -14.697 | 0 | 0 |
| ENSG00000279865 |  | -4.447 | 0.346 | -12.868 | 0 | 0 |
| ENSG00000243885 |  | -4.435 | 0.877 | -5.057 | 0 | 0 |
| ENSG00000179428 | IL6-AS1 | -4.413 | 0.437 | -10.099 | 0 | 0 |
| ENSG00000130487 | KLHDC7B | -4.406 | 1.057 | -4.168 | 0 | 0.0002 |
| ENSG00000276953 | TRBV12-4 | -4.378 | 0.998 | -4.388 | 0 | 0.0001 |
| ENSG00000235488 | JARID2-AS1 | -4.374 | 0.706 | -6.194 | 0 | 0 |
| ENSG00000225383 | SFTA1P | -4.359 | 0.629 | -6.933 | 0 | 0 |
| ENSG00000213417 | KRTAP2-4 | -4.338 | 1.052 | -4.123 | 0 | 0.0002 |
| ENSG00000275091 |  | -4.337 | 0.963 | -4.505 | 0 | 0.0001 |
| ENSG00000260563 |  | -4.311 | 0.369 | -11.673 | 0 | 0 |
| ENSG00000224077 |  | -4.303 | 1.073 | -4.009 | 0.0001 | 0.0004 |
| ENSG00000231858 | STAT4-AS1 | -4.298 | 0.946 | -4.542 | 0 | 0 |
| ENSG00000257999 |  | -4.295 | 1.262 | -3.403 | 0.0007 | 0.0029 |
| ENSG00000235070 |  | -4.286 | 0.652 | -6.578 | 0 | 0 |
| ENSG00000225541 |  | -4.281 | 1.012 | -4.23 | 0 | 0.0002 |
| ENSG00000280278 | FLJ30679 | -4.272 | 0.331 | -12.906 | 0 | 0 |
| ENSG00000277301 | LOC101929698 | -4.263 | 1.163 | -3.666 | 0.0002 | 0.0012 |
| ENSG00000261513 |  | -4.241 | 0.796 | -5.324 | 0 | 0 |
| ENSG00000234052 |  | -4.238 | 0.852 | -4.976 | 0 | 0 |
| ENSG00000263812 |  | -4.231 | 1.109 | -3.817 | 0.0001 | 0.0007 |
| ENSG00000232969 |  | -4.225 | 1.129 | -3.742 | 0.0002 | 0.001 |
| ENSG00000196166 | C8orf86 | -4.215 | 0.414 | -10.18 | 0 | 0 |
| ENSG00000234676 | IFT74-AS1 | -4.184 | 1.125 | -3.718 | 0.0002 | 0.001 |
| ENSG00000237803 | LINC00211 | -4.163 | 0.33 | -12.616 | 0 | 0 |
| ENSG00000235749 |  | -4.154 | 0.598 | -6.945 | 0 | 0 |
| ENSG00000115009 | CCL20 | -4.141 | 0.549 | -7.544 | 0 | 0 |
| ENSG00000227941 | UQCRBP2 | -4.127 | 1.011 | -4.081 | 0 | 0.0003 |
| ENSG00000125538 | IL1B | -4.122 | 0.556 | -7.42 | 0 | 0 |
| ENSG00000153230 | OR14K1 | -4.121 | 0.584 | -7.056 | 0 | 0 |
| ENSG00000272788 |  | -4.112 | 0.532 | -7.732 | 0 | 0 |
| ENSG00000234292 |  | -4.11 | 0.517 | -7.955 | 0 | 0 |
| ENSG00000253140 |  | -4.105 | 0.567 | -7.245 | 0 | 0 |
| ENSG00000273210 |  | -4.083 | 0.372 | -10.978 | 0 | 0 |
| ENSG00000256678 |  | -4.073 | 0.442 | -9.209 | 0 | 0 |
| ENSG00000260619 |  | -4.07 | 0.572 | -7.118 | 0 | 0 |
| ENSG00000267589 |  | -4.051 | 0.701 | -5.782 | 0 | 0 |
| ENSG00000138136 | LBX1 | -4.051 | 0.888 | -4.563 | 0 | 0 |
| ENSG00000265692 | LINC01970 | -4.041 | 0.551 | -7.331 | 0 | 0 |
| ENSG00000271387 |  | -4.04 | 0.733 | -5.511 | 0 | 0 |
| ENSG00000262412 |  | -4.015 | 0.786 | -5.107 | 0 | 0 |
| ENSG00000272463 |  | -4.009 | 0.588 | -6.819 | 0 | 0 |
| ENSG00000278743 |  | -4.007 | 0.451 | -8.877 | 0 | 0 |
| ENSG00000224547 |  | -3.974 | 1.339 | -2.967 | 0.003 | 0.0103 |
| ENSG00000124766 | SOX4 | -3.969 | 0.263 | -15.068 | 0 | 0 |
| ENSG00000163576 | EFHB | -3.964 | 0.814 | -4.868 | 0 | 0 |
| ENSG00000283122 | HYMAI | -3.964 | 0.393 | -10.1 | 0 | 0 |
| ENSG00000167741 | GGT6 | -3.96 | 1.166 | -3.397 | 0.0007 | 0.0029 |
| ENSG00000229931 | ATXN1-AS1 | -3.958 | 0.5 | -7.918 | 0 | 0 |
| ENSG00000239888 | RN7SL792P | -3.948 | 1.326 | -2.978 | 0.0029 | 0.01 |
| ENSG00000212710 | CTAGE1 | -3.94 | 0.475 | -8.296 | 0 | 0 |
| ENSG00000187686 | KRT18P59 | -3.931 | 0.616 | -6.38 | 0 | 0 |
| ENSG00000213726 | RPS2P52 | -3.923 | 1.332 | -2.944 | 0.0032 | 0.011 |
| ENSG00000207808 | MIR27A | -3.913 | 0.622 | -6.292 | 0 | 0 |
| ENSG00000224843 | LINC00240 | -3.906 | 0.975 | -4.008 | 0.0001 | 0.0004 |
| ENSG00000279433 |  | -3.905 | 1.117 | -3.497 | 0.0005 | 0.0021 |
| ENSG00000255100 | TSKU-AS1 | -3.9 | 0.752 | -5.189 | 0 | 0 |
| ENSG00000272774 |  | -3.899 | 0.74 | -5.267 | 0 | 0 |
| ENSG00000261420 | MPC1-DT | -3.898 | 0.832 | -4.686 | 0 | 0 |
| ENSG00000235481 |  | -3.896 | 0.73 | -5.334 | 0 | 0 |
| ENSG00000270846 |  | -3.896 | 1.156 | -3.371 | 0.0007 | 0.0032 |
| ENSG00000272795 |  | -3.895 | 1.594 | -2.443 | 0.0146 | 0.0384 |
| ENSG00000254991 |  | -3.877 | 0.514 | -7.546 | 0 | 0 |
| ENSG00000203593 |  | -3.877 | 0.925 | -4.191 | 0 | 0.0002 |
| ENSG00000244558 |  | -3.86 | 0.895 | -4.315 | 0 | 0.0001 |
| ENSG00000213857 |  | -3.857 | 1.053 | -3.665 | 0.0002 | 0.0012 |
| ENSG00000170296 | GABARAP | -3.85 | 0.398 | -9.673 | 0 | 0 |
| ENSG00000279088 |  | -3.848 | 0.268 | -14.338 | 0 | 0 |
| ENSG00000265458 |  | -3.845 | 0.607 | -6.331 | 0 | 0 |
| ENSG00000230621 |  | -3.843 | 1.588 | -2.42 | 0.0155 | 0.0405 |
| ENSG00000272691 |  | -3.84 | 0.757 | -5.075 | 0 | 0 |
| ENSG00000231050 |  | -3.835 | 0.886 | -4.329 | 0 | 0.0001 |
| ENSG00000224977 | LINC02776 | -3.827 | 1 | -3.828 | 0.0001 | 0.0007 |
| ENSG00000272043 |  | -3.824 | 1.43 | -2.675 | 0.0075 | 0.0223 |
| ENSG00000132464 | ENAM | -3.819 | 0.679 | -5.625 | 0 | 0 |
| ENSG00000272192 |  | -3.813 | 0.834 | -4.574 | 0 | 0 |
| ENSG00000115008 | IL1A | -3.805 | 0.579 | -6.572 | 0 | 0 |
| ENSG00000276984 |  | -3.796 | 1.014 | -3.744 | 0.0002 | 0.0009 |
| ENSG00000069188 | SDK2 | -3.784 | 0.943 | -4.013 | 0.0001 | 0.0004 |
| ENSG00000204880 | KRTAP4-8 | -3.772 | 0.966 | -3.907 | 0.0001 | 0.0005 |
| ENSG00000261449 |  | -3.758 | 0.868 | -4.332 | 0 | 0.0001 |
| ENSG00000271983 |  | -3.746 | 0.915 | -4.094 | 0 | 0.0003 |
| ENSG00000268628 |  | -3.741 | 1.175 | -3.185 | 0.0014 | 0.0056 |
| ENSG00000277999 |  | -3.738 | 1.062 | -3.518 | 0.0004 | 0.002 |
| ENSG00000244041 | LINC01011 | -3.727 | 0.429 | -8.697 | 0 | 0 |
| ENSG00000231856 |  | -3.717 | 0.347 | -10.727 | 0 | 0 |
| ENSG00000278017 |  | -3.711 | 0.576 | -6.443 | 0 | 0 |
| ENSG00000241933 | DENND6A-DT | -3.708 | 0.65 | -5.701 | 0 | 0 |
| ENSG00000232953 |  | -3.708 | 0.849 | -4.369 | 0 | 0.0001 |
| ENSG00000257698 | GIHCG | -3.704 | 0.365 | -10.153 | 0 | 0 |
| ENSG00000237136 | C4orf51 | -3.694 | 0.915 | -4.038 | 0.0001 | 0.0003 |
| ENSG00000260008 |  | -3.688 | 0.621 | -5.942 | 0 | 0 |
| ENSG00000229896 |  | -3.683 | 0.786 | -4.688 | 0 | 0 |
| ENSG00000240541 | TM4SF1-AS1 | -3.675 | 0.863 | -4.257 | 0 | 0.0001 |
| ENSG00000271167 |  | -3.671 | 0.714 | -5.14 | 0 | 0 |
| ENSG00000272906 |  | -3.656 | 0.57 | -6.419 | 0 | 0 |
| ENSG00000225766 | DHRS4L1 | -3.656 | 1.254 | -2.915 | 0.0036 | 0.0119 |
| ENSG00000236651 | DLX2-DT | -3.649 | 1.055 | -3.459 | 0.0005 | 0.0024 |
| ENSG00000278156 | TSC22D1-AS1 | -3.633 | 0.456 | -7.967 | 0 | 0 |
| ENSG00000225407 |  | -3.632 | 0.892 | -4.073 | 0 | 0.0003 |
| ENSG00000261758 |  | -3.626 | 0.442 | -8.212 | 0 | 0 |
| ENSG00000261839 |  | -3.616 | 0.908 | -3.981 | 0.0001 | 0.0004 |
| ENSG00000272482 |  | -3.612 | 0.614 | -5.887 | 0 | 0 |
| ENSG00000226359 | ACTG1P24 | -3.609 | 0.6 | -6.019 | 0 | 0 |
| ENSG00000235159 |  | -3.606 | 0.563 | -6.407 | 0 | 0 |
| ENSG00000231704 |  | -3.599 | 0.885 | -4.066 | 0 | 0.0003 |
| ENSG00000170374 | SP7 | -3.598 | 0.925 | -3.889 | 0.0001 | 0.0006 |
| ENSG00000200972 | RNU5A-8P | -3.593 | 1.04 | -3.454 | 0.0006 | 0.0025 |
| ENSG00000235371 |  | -3.586 | 0.641 | -5.597 | 0 | 0 |
| ENSG00000265273 | PGDP1 | -3.579 | 1.11 | -3.225 | 0.0013 | 0.005 |
| ENSG00000275198 |  | -3.578 | 0.372 | -9.607 | 0 | 0 |
| ENSG00000259642 |  | -3.577 | 0.338 | -10.593 | 0 | 0 |
| ENSG00000230266 | XXYLT1-AS2 | -3.56 | 0.348 | -10.233 | 0 | 0 |
| ENSG00000234707 |  | -3.555 | 0.906 | -3.924 | 0.0001 | 0.0005 |
| ENSG00000197768 | STPG3 | -3.554 | 0.709 | -5.011 | 0 | 0 |
| ENSG00000169876 | MUC17 | -3.554 | 0.381 | -9.333 | 0 | 0 |
| ENSG00000255959 |  | -3.553 | 1.053 | -3.375 | 0.0007 | 0.0032 |
| ENSG00000212517 |  | -3.546 | 1.012 | -3.506 | 0.0005 | 0.0021 |
| ENSG00000255458 |  | -3.545 | 0.484 | -7.324 | 0 | 0 |
| ENSG00000231530 |  | -3.543 | 0.726 | -4.879 | 0 | 0 |
| ENSG00000227527 |  | -3.54 | 0.743 | -4.765 | 0 | 0 |
| ENSG00000229582 |  | -3.54 | 0.662 | -5.349 | 0 | 0 |
| ENSG00000184349 | EFNA5 | -3.539 | 0.377 | -9.378 | 0 | 0 |
| ENSG00000272172 |  | -3.537 | 0.813 | -4.352 | 0 | 0.0001 |
| ENSG00000206652 | RNU1-1 | -3.533 | 1.368 | -2.583 | 0.0098 | 0.0277 |
| ENSG00000253702 |  | -3.527 | 0.916 | -3.851 | 0.0001 | 0.0006 |
| ENSG00000261693 |  | -3.524 | 0.564 | -6.252 | 0 | 0 |
| ENSG00000203685 | STUM | -3.52 | 0.859 | -4.1 | 0 | 0.0003 |
| ENSG00000279212 |  | -3.518 | 0.691 | -5.095 | 0 | 0 |
| ENSG00000261407 |  | -3.502 | 0.751 | -4.661 | 0 | 0 |
| ENSG00000270722 |  | -3.502 | 1.317 | -2.658 | 0.0079 | 0.0232 |
| ENSG00000273344 | PAXIP1-DT | -3.494 | 0.264 | -13.23 | 0 | 0 |
| ENSG00000252755 | RNU6-703P | -3.489 | 0.995 | -3.506 | 0.0005 | 0.0021 |
| ENSG00000178789 | CD300LB | -3.483 | 0.713 | -4.884 | 0 | 0 |
| ENSG00000230359 | TPI1P2 | -3.481 | 0.288 | -12.077 | 0 | 0 |
| ENSG00000228675 |  | -3.476 | 0.554 | -6.269 | 0 | 0 |
| ENSG00000253408 |  | -3.467 | 0.611 | -5.67 | 0 | 0 |
| ENSG00000203364 |  | -3.46 | 0.958 | -3.612 | 0.0003 | 0.0015 |
| ENSG00000259736 | CRTC3-AS1 | -3.458 | 0.842 | -4.109 | 0 | 0.0003 |
| ENSG00000255355 |  | -3.457 | 0.473 | -7.316 | 0 | 0 |
| ENSG00000255400 |  | -3.445 | 0.486 | -7.088 | 0 | 0 |
| ENSG00000240871 | KRTAP4-7 | -3.441 | 0.764 | -4.504 | 0 | 0.0001 |
| ENSG00000274598 |  | -3.434 | 0.659 | -5.209 | 0 | 0 |
| ENSG00000232536 |  | -3.433 | 0.485 | -7.078 | 0 | 0 |
| ENSG00000267666 |  | -3.426 | 0.728 | -4.706 | 0 | 0 |
| ENSG00000272426 |  | -3.426 | 0.538 | -6.369 | 0 | 0 |
| ENSG00000270361 |  | -3.424 | 0.456 | -7.503 | 0 | 0 |
| ENSG00000267226 | LOC101927322 | -3.424 | 0.399 | -8.573 | 0 | 0 |
| ENSG00000189238 | LINC00943 | -3.422 | 0.743 | -4.605 | 0 | 0 |
| ENSG00000269600 |  | -3.42 | 0.871 | -3.924 | 0.0001 | 0.0005 |
| ENSG00000214518 | KRTAP2-2 | -3.42 | 0.909 | -3.763 | 0.0002 | 0.0009 |
| ENSG00000234698 |  | -3.416 | 1.147 | -2.978 | 0.0029 | 0.01 |
| ENSG00000276603 |  | -3.415 | 0.682 | -5.011 | 0 | 0 |
| ENSG00000269927 |  | -3.41 | 0.562 | -6.064 | 0 | 0 |
| ENSG00000185624 | P4HB | -3.409 | 0.212 | -16.093 | 0 | 0 |
| ENSG00000254678 |  | -3.403 | 0.703 | -4.84 | 0 | 0 |
| ENSG00000250061 |  | -3.386 | 1.409 | -2.404 | 0.0162 | 0.0421 |
| ENSG00000224892 | RPS4XP16 | -3.385 | 0.877 | -3.861 | 0.0001 | 0.0006 |
| ENSG00000265683 | SYPL1P2 | -3.38 | 0.518 | -6.524 | 0 | 0 |
| ENSG00000271888 |  | -3.372 | 0.558 | -6.048 | 0 | 0 |
| ENSG00000275405 |  | -3.366 | 1.036 | -3.248 | 0.0012 | 0.0046 |
| ENSG00000258179 |  | -3.362 | 0.61 | -5.512 | 0 | 0 |
| ENSG00000173451 | THAP2 | -3.361 | 0.269 | -12.513 | 0 | 0 |
| ENSG00000265451 |  | -3.357 | 0.402 | -8.34 | 0 | 0 |
| ENSG00000267412 |  | -3.356 | 0.687 | -4.883 | 0 | 0 |
| ENSG00000105549 | THEG | -3.356 | 1.017 | -3.301 | 0.001 | 0.004 |
| ENSG00000223401 |  | -3.355 | 0.502 | -6.685 | 0 | 0 |
| ENSG00000259347 |  | -3.353 | 0.713 | -4.701 | 0 | 0 |
| ENSG00000166292 | TMEM100 | -3.353 | 1.085 | -3.091 | 0.002 | 0.0073 |
| ENSG00000264456 |  | -3.351 | 0.524 | -6.399 | 0 | 0 |
| ENSG00000270482 |  | -3.347 | 0.389 | -8.606 | 0 | 0 |
| ENSG00000260021 |  | -3.346 | 0.917 | -3.65 | 0.0003 | 0.0013 |
| ENSG00000225676 | LOC105372988 | -3.344 | 0.572 | -5.843 | 0 | 0 |
| ENSG00000180660 | MAB21L1 | -3.344 | 0.333 | -10.048 | 0 | 0 |
| ENSG00000270419 | CAHM | -3.34 | 0.393 | -8.507 | 0 | 0 |
| ENSG00000205364 | MT1M | -3.34 | 0.463 | -7.22 | 0 | 0 |
| ENSG00000275484 |  | -3.34 | 0.383 | -8.728 | 0 | 0 |
| ENSG00000073067 | CYP2W1 | -3.339 | 0.763 | -4.377 | 0 | 0.0001 |
| ENSG00000271926 |  | -3.339 | 1.031 | -3.239 | 0.0012 | 0.0048 |
| ENSG00000259224 | SLC35G6 | -3.33 | 0.405 | -8.224 | 0 | 0 |
| ENSG00000260410 |  | -3.327 | 0.537 | -6.196 | 0 | 0 |
| ENSG00000255537 | LOC403312 | -3.325 | 0.823 | -4.039 | 0.0001 | 0.0003 |
| ENSG00000277287 |  | -3.32 | 0.328 | -10.136 | 0 | 0 |
| ENSG00000177989 | ODF3B | -3.316 | 0.481 | -6.89 | 0 | 0 |
| ENSG00000226457 | RPL22P3 | -3.312 | 0.78 | -4.249 | 0 | 0.0001 |
| ENSG00000138231 | DBR1 | -3.311 | 0.289 | -11.454 | 0 | 0 |
| ENSG00000270195 |  | -3.296 | 0.504 | -6.542 | 0 | 0 |
| ENSG00000233521 | LINC01638 | -3.296 | 1.276 | -2.583 | 0.0098 | 0.0277 |
| ENSG00000273783 |  | -3.291 | 0.393 | -8.378 | 0 | 0 |
| ENSG00000212722 | KRTAP4-9 | -3.29 | 0.699 | -4.705 | 0 | 0 |
| ENSG00000273565 |  | -3.289 | 0.429 | -7.672 | 0 | 0 |
| ENSG00000226754 | LOC100507564 | -3.288 | 0.542 | -6.066 | 0 | 0 |
| ENSG00000277981 |  | -3.287 | 1.241 | -2.648 | 0.0081 | 0.0237 |
| ENSG00000135677 | GNS | -3.286 | 0.208 | -15.824 | 0 | 0 |
| ENSG00000220412 |  | -3.282 | 0.427 | -7.688 | 0 | 0 |
| ENSG00000222043 |  | -3.279 | 0.341 | -9.618 | 0 | 0 |
| ENSG00000236867 |  | -3.276 | 1.058 | -3.096 | 0.002 | 0.0072 |
| ENSG00000237862 | LOC100506271 | -3.273 | 0.823 | -3.977 | 0.0001 | 0.0004 |
| ENSG00000206585 | RNVU1-7 | -3.272 | 0.632 | -5.175 | 0 | 0 |
| ENSG00000178397 | FAM220A | -3.259 | 0.267 | -12.201 | 0 | 0 |
| ENSG00000205562 |  | -3.256 | 0.32 | -10.17 | 0 | 0 |
| ENSG00000227036 | LINC00511 | -3.254 | 0.357 | -9.125 | 0 | 0 |
| ENSG00000276026 |  | -3.252 | 0.742 | -4.385 | 0 | 0.0001 |
| ENSG00000160973 | FOXH1 | -3.249 | 1.323 | -2.455 | 0.0141 | 0.0374 |
| ENSG00000236095 |  | -3.243 | 0.703 | -4.615 | 0 | 0 |
| ENSG00000277459 |  | -3.242 | 0.462 | -7.024 | 0 | 0 |
| ENSG00000270705 |  | -3.242 | 1.013 | -3.201 | 0.0014 | 0.0053 |
| ENSG00000273821 |  | -3.242 | 1.24 | -2.614 | 0.0089 | 0.0257 |
| ENSG00000260475 |  | -3.232 | 0.363 | -8.893 | 0 | 0 |
| ENSG00000163612 | FAM86KP | -3.232 | 1.086 | -2.976 | 0.0029 | 0.0101 |
| ENSG00000213433 |  | -3.23 | 0.872 | -3.703 | 0.0002 | 0.0011 |
| ENSG00000272008 |  | -3.227 | 0.339 | -9.533 | 0 | 0 |
| ENSG00000278071 |  | -3.221 | 0.384 | -8.385 | 0 | 0 |
| ENSG00000240583 | AQP1 | -3.219 | 0.966 | -3.333 | 0.0009 | 0.0036 |
| ENSG00000271709 |  | -3.219 | 1.013 | -3.179 | 0.0015 | 0.0057 |
| ENSG00000236643 |  | -3.218 | 1.049 | -3.067 | 0.0022 | 0.0078 |
| ENSG00000231154 |  | -3.217 | 0.798 | -4.032 | 0.0001 | 0.0003 |
| ENSG00000251040 | LINC02480 | -3.21 | 0.99 | -3.241 | 0.0012 | 0.0047 |
| ENSG00000277602 |  | -3.208 | 0.662 | -4.847 | 0 | 0 |
| ENSG00000138892 | TTLL8 | -3.206 | 0.794 | -4.036 | 0.0001 | 0.0003 |
| ENSG00000268635 |  | -3.201 | 0.92 | -3.48 | 0.0005 | 0.0023 |
| ENSG00000279873 | LINC01126 | -3.199 | 0.416 | -7.681 | 0 | 0 |
| ENSG00000234028 | EIF2AK3-DT | -3.198 | 0.365 | -8.752 | 0 | 0 |
| ENSG00000185442 | FAM174B | -3.196 | 0.358 | -8.935 | 0 | 0 |
| ENSG00000251676 | SNHG27 | -3.195 | 0.862 | -3.707 | 0.0002 | 0.0011 |
| ENSG00000253848 |  | -3.191 | 0.779 | -4.096 | 0 | 0.0003 |
| ENSG00000240050 |  | -3.188 | 0.784 | -4.067 | 0 | 0.0003 |
| ENSG00000255200 |  | -3.185 | 0.501 | -6.355 | 0 | 0 |
| ENSG00000272081 |  | -3.185 | 0.458 | -6.958 | 0 | 0 |
| ENSG00000102554 | KLF5 | -3.185 | 0.264 | -12.066 | 0 | 0 |
| ENSG00000244268 |  | -3.185 | 0.887 | -3.591 | 0.0003 | 0.0016 |
| ENSG00000272719 |  | -3.179 | 0.825 | -3.853 | 0.0001 | 0.0006 |
| ENSG00000278600 |  | -3.178 | 0.297 | -10.707 | 0 | 0 |
| ENSG00000271857 |  | -3.178 | 0.967 | -3.287 | 0.001 | 0.0041 |
| ENSG00000272767 | JMJD1C-AS1 | -3.175 | 0.395 | -8.035 | 0 | 0 |
| ENSG00000277406 |  | -3.173 | 0.922 | -3.442 | 0.0006 | 0.0026 |
| ENSG00000260206 |  | -3.172 | 1.034 | -3.068 | 0.0022 | 0.0078 |
| ENSG00000272703 |  | -3.17 | 0.729 | -4.346 | 0 | 0.0001 |
| ENSG00000155511 | GRIA1 | -3.169 | 0.808 | -3.923 | 0.0001 | 0.0005 |
| ENSG00000248288 |  | -3.168 | 0.578 | -5.48 | 0 | 0 |
| ENSG00000258099 | ATXN2-AS | -3.166 | 0.354 | -8.934 | 0 | 0 |
| ENSG00000253102 |  | -3.166 | 1.188 | -2.665 | 0.0077 | 0.0228 |
| ENSG00000197249 | SERPINA1 | -3.162 | 0.41 | -7.707 | 0 | 0 |
| ENSG00000279879 |  | -3.159 | 0.365 | -8.653 | 0 | 0 |
| ENSG00000105711 | SCN1B | -3.159 | 0.501 | -6.303 | 0 | 0 |
| ENSG00000242190 |  | -3.156 | 0.464 | -6.799 | 0 | 0 |
| ENSG00000278847 |  | -3.147 | 0.939 | -3.353 | 0.0008 | 0.0034 |
| ENSG00000259943 |  | -3.146 | 0.341 | -9.23 | 0 | 0 |
| ENSG00000242474 |  | -3.145 | 0.486 | -6.469 | 0 | 0 |
| ENSG00000273063 |  | -3.139 | 0.642 | -4.886 | 0 | 0 |
| ENSG00000271784 |  | -3.136 | 0.764 | -4.106 | 0 | 0.0003 |
| ENSG00000233060 |  | -3.125 | 1.181 | -2.646 | 0.0082 | 0.0239 |
| ENSG00000258509 |  | -3.124 | 1.206 | -2.59 | 0.0096 | 0.0272 |
| ENSG00000266677 |  | -3.123 | 1.24 | -2.518 | 0.0118 | 0.0323 |
| ENSG00000233069 |  | -3.122 | 0.407 | -7.672 | 0 | 0 |
| ENSG00000249252 | C1QTNF7-AS1 | -3.121 | 1.052 | -2.967 | 0.003 | 0.0103 |
| ENSG00000272524 |  | -3.12 | 0.636 | -4.905 | 0 | 0 |
| ENSG00000242111 |  | -3.119 | 0.396 | -7.884 | 0 | 0 |
| ENSG00000254614 | LOC728975 | -3.115 | 0.403 | -7.722 | 0 | 0 |
| ENSG00000246022 | ALDH1L1-AS2 | -3.114 | 0.44 | -7.076 | 0 | 0 |
| ENSG00000140479 | PCSK6 | -3.113 | 0.584 | -5.329 | 0 | 0 |
| ENSG00000266473 |  | -3.113 | 0.606 | -5.138 | 0 | 0 |
| ENSG00000234361 |  | -3.103 | 1.006 | -3.084 | 0.002 | 0.0074 |
| ENSG00000185565 | LSAMP | -3.093 | 0.434 | -7.135 | 0 | 0 |
| ENSG00000199568 | RNU5A-1 | -3.09 | 0.383 | -8.057 | 0 | 0 |
| ENSG00000165511 | ZNF22-AS1 | -3.087 | 0.352 | -8.768 | 0 | 0 |
| ENSG00000153404 | PLEKHG4B | -3.084 | 0.394 | -7.825 | 0 | 0 |
| ENSG00000271382 |  | -3.084 | 0.941 | -3.279 | 0.001 | 0.0042 |
| ENSG00000260542 |  | -3.078 | 0.958 | -3.214 | 0.0013 | 0.0051 |
| ENSG00000260852 | FBXL19-AS1 | -3.077 | 0.324 | -9.491 | 0 | 0 |
| ENSG00000283098 |  | -3.069 | 0.567 | -5.415 | 0 | 0 |
| ENSG00000234956 | LINC02539 | -3.069 | 0.715 | -4.291 | 0 | 0.0001 |
| ENSG00000198885 | ITPRIPL1 | -3.063 | 0.544 | -5.633 | 0 | 0 |
| ENSG00000256894 |  | -3.063 | 0.671 | -4.562 | 0 | 0 |
| ENSG00000273145 |  | -3.063 | 0.686 | -4.468 | 0 | 0.0001 |
| ENSG00000261366 | MANEA-DT | -3.062 | 0.987 | -3.103 | 0.0019 | 0.007 |
| ENSG00000221852 | KRTAP1-5 | -3.059 | 0.301 | -10.173 | 0 | 0 |
| ENSG00000214049 | UCA1 | -3.058 | 1.085 | -2.817 | 0.0048 | 0.0155 |
| ENSG00000180745 | CLRN3 | -3.055 | 1.217 | -2.51 | 0.0121 | 0.0329 |
| ENSG00000267417 |  | -3.054 | 0.779 | -3.922 | 0.0001 | 0.0005 |
| ENSG00000049860 | HEXB | -3.052 | 0.246 | -12.414 | 0 | 0 |
| ENSG00000274884 |  | -3.052 | 0.505 | -6.048 | 0 | 0 |
| ENSG00000269842 |  | -3.052 | 0.992 | -3.076 | 0.0021 | 0.0076 |
| ENSG00000277324 |  | -3.051 | 0.569 | -5.36 | 0 | 0 |
| ENSG00000245059 |  | -3.049 | 0.652 | -4.676 | 0 | 0 |
| ENSG00000231747 |  | -3.046 | 0.794 | -3.839 | 0.0001 | 0.0007 |
| ENSG00000261222 |  | -3.045 | 0.901 | -3.378 | 0.0007 | 0.0031 |
| ENSG00000174007 | CEP19 | -3.043 | 0.322 | -9.455 | 0 | 0 |
| ENSG00000115844 | DLX2 | -3.039 | 0.252 | -12.064 | 0 | 0 |
| ENSG00000272663 |  | -3.038 | 0.277 | -10.976 | 0 | 0 |
| ENSG00000271427 |  | -3.037 | 0.495 | -6.134 | 0 | 0 |
| ENSG00000232759 | LOC100506178 | -3.033 | 0.426 | -7.124 | 0 | 0 |
| ENSG00000273026 |  | -3.032 | 0.712 | -4.26 | 0 | 0.0001 |
| ENSG00000259448 |  | -3.032 | 0.951 | -3.187 | 0.0014 | 0.0056 |
| ENSG00000226984 | RPL26P10 | -3.031 | 0.57 | -5.313 | 0 | 0 |
| ENSG00000251442 |  | -3.028 | 0.436 | -6.939 | 0 | 0 |
| ENSG00000181798 | LINC00471 | -3.027 | 0.543 | -5.573 | 0 | 0 |
| ENSG00000257696 |  | -3.027 | 0.857 | -3.534 | 0.0004 | 0.0019 |
| ENSG00000162975 | KCNF1 | -3.026 | 0.919 | -3.294 | 0.001 | 0.004 |
| ENSG00000243819 | RN7SL832P | -3.025 | 0.399 | -7.586 | 0 | 0 |
| ENSG00000270019 |  | -3.02 | 0.433 | -6.977 | 0 | 0 |
| ENSG00000261840 | LOC730183 | -3.018 | 0.424 | -7.113 | 0 | 0 |
| ENSG00000228340 | MIR646HG | -3.018 | 0.957 | -3.153 | 0.0016 | 0.0061 |
| ENSG00000277692 |  | -3.011 | 0.589 | -5.112 | 0 | 0 |
| ENSG00000272764 |  | -3.011 | 0.439 | -6.865 | 0 | 0 |
| ENSG00000205231 | TTLL10-AS1 | -3.005 | 0.592 | -5.073 | 0 | 0 |
| ENSG00000256442 | LOC105369728 | -3.001 | 0.644 | -4.661 | 0 | 0 |
| ENSG00000272734 | ADIRF-AS1 | -3 | 0.269 | -11.131 | 0 | 0 |
| ENSG00000266405 | CBX3P2 | -2.997 | 0.667 | -4.492 | 0 | 0.0001 |
| ENSG00000225342 |  | -2.996 | 0.705 | -4.251 | 0 | 0.0001 |
| ENSG00000279306 |  | -2.995 | 0.716 | -4.185 | 0 | 0.0002 |
| ENSG00000261269 |  | -2.979 | 0.732 | -4.072 | 0 | 0.0003 |
| ENSG00000249035 | CLMAT3 | -2.968 | 0.403 | -7.357 | 0 | 0 |
| ENSG00000225269 | LINC00705 | -2.968 | 0.92 | -3.226 | 0.0013 | 0.005 |
| ENSG00000230445 | LRRC37A6P | -2.967 | 0.593 | -5.006 | 0 | 0 |
| ENSG00000170899 | GSTA4 | -2.958 | 0.399 | -7.41 | 0 | 0 |
| ENSG00000054148 | PHPT1 | -2.953 | 0.226 | -13.039 | 0 | 0 |
| ENSG00000227220 |  | -2.953 | 0.869 | -3.397 | 0.0007 | 0.003 |
| ENSG00000100258 | LMF2 | -2.952 | 0.239 | -12.362 | 0 | 0 |
| ENSG00000254028 |  | -2.951 | 0.596 | -4.949 | 0 | 0 |
| ENSG00000207005 | RNU1-2 | -2.948 | 0.698 | -4.223 | 0 | 0.0002 |
| ENSG00000223820 | CFL1P1 | -2.946 | 0.392 | -7.507 | 0 | 0 |
| ENSG00000261504 | LINC01686 | -2.94 | 0.738 | -3.981 | 0.0001 | 0.0004 |
| ENSG00000273314 |  | -2.937 | 0.388 | -7.568 | 0 | 0 |
| ENSG00000279118 |  | -2.935 | 0.937 | -3.131 | 0.0017 | 0.0065 |
| ENSG00000261447 |  | -2.934 | 0.513 | -5.722 | 0 | 0 |
| ENSG00000151303 |  | -2.933 | 1.156 | -2.538 | 0.0112 | 0.0309 |
| ENSG00000240005 |  | -2.931 | 0.505 | -5.804 | 0 | 0 |
| ENSG00000246100 |  | -2.931 | 0.676 | -4.337 | 0 | 0.0001 |
| ENSG00000102935 | ZNF423 | -2.928 | 1.238 | -2.365 | 0.018 | 0.0459 |
| ENSG00000223734 |  | -2.927 | 0.527 | -5.551 | 0 | 0 |
| ENSG00000229124 | VIM-AS1 | -2.926 | 0.278 | -10.52 | 0 | 0 |
| ENSG00000254662 |  | -2.925 | 1.162 | -2.517 | 0.0118 | 0.0324 |
| ENSG00000265185 |  | -2.916 | 0.498 | -5.858 | 0 | 0 |
| ENSG00000230449 | RPL7P4 | -2.916 | 0.441 | -6.612 | 0 | 0 |
| ENSG00000108551 | RASD1 | -2.915 | 0.306 | -9.533 | 0 | 0 |
| ENSG00000217646 | H2BC16P | -2.914 | 1.13 | -2.579 | 0.0099 | 0.028 |
| ENSG00000115738 | ID2 | -2.911 | 0.258 | -11.28 | 0 | 0 |
| ENSG00000260526 |  | -2.911 | 0.432 | -6.744 | 0 | 0 |
| ENSG00000254588 | ETS1-AS1 | -2.91 | 0.874 | -3.331 | 0.0009 | 0.0036 |
| ENSG00000264663 |  | -2.908 | 1.136 | -2.559 | 0.0105 | 0.0293 |
| ENSG00000253559 |  | -2.907 | 0.793 | -3.668 | 0.0002 | 0.0012 |
| ENSG00000244998 |  | -2.907 | 1.002 | -2.9 | 0.0037 | 0.0124 |
| ENSG00000224421 | ATP5MFP1 | -2.902 | 0.785 | -3.695 | 0.0002 | 0.0011 |
| ENSG00000272662 |  | -2.899 | 0.407 | -7.129 | 0 | 0 |
| ENSG00000264475 |  | -2.899 | 1.084 | -2.675 | 0.0075 | 0.0223 |
| ENSG00000278934 |  | -2.894 | 0.821 | -3.525 | 0.0004 | 0.002 |
| ENSG00000196557 | CACNA1H | -2.892 | 0.581 | -4.977 | 0 | 0 |
| ENSG00000146700 | SSC4D | -2.892 | 0.747 | -3.874 | 0.0001 | 0.0006 |
| ENSG00000270022 |  | -2.887 | 0.423 | -6.827 | 0 | 0 |
| ENSG00000188483 | IER5L | -2.886 | 0.302 | -9.544 | 0 | 0 |
| ENSG00000272506 |  | -2.881 | 0.629 | -4.579 | 0 | 0 |
| ENSG00000234062 |  | -2.879 | 0.804 | -3.579 | 0.0003 | 0.0016 |
| ENSG00000140379 | BCL2A1 | -2.878 | 0.421 | -6.843 | 0 | 0 |
| ENSG00000230457 |  | -2.878 | 0.305 | -9.424 | 0 | 0 |
| ENSG00000250548 | LINC01303 | -2.877 | 0.361 | -7.971 | 0 | 0 |
| ENSG00000275216 |  | -2.876 | 0.48 | -5.99 | 0 | 0 |
| ENSG00000264230 | ANXA8L1 | -2.873 | 0.78 | -3.684 | 0.0002 | 0.0012 |
| ENSG00000280129 |  | -2.869 | 0.612 | -4.686 | 0 | 0 |
| ENSG00000167604 | NFKBID | -2.868 | 0.394 | -7.284 | 0 | 0 |
| ENSG00000264235 | LOC104968399 | -2.859 | 0.405 | -7.053 | 0 | 0 |
| ENSG00000110200 | ANAPC15 | -2.856 | 0.256 | -11.141 | 0 | 0 |
| ENSG00000257803 |  | -2.855 | 1.072 | -2.662 | 0.0078 | 0.023 |
| ENSG00000272784 |  | -2.853 | 0.871 | -3.277 | 0.0011 | 0.0043 |
| ENSG00000228363 |  | -2.851 | 0.392 | -7.267 | 0 | 0 |
| ENSG00000132823 | OSER1 | -2.85 | 0.268 | -10.635 | 0 | 0 |
| ENSG00000223522 | LOC100505716 | -2.849 | 0.882 | -3.229 | 0.0012 | 0.0049 |
| ENSG00000232160 | RAP2C-AS1 | -2.847 | 0.912 | -3.122 | 0.0018 | 0.0067 |
| ENSG00000269397 |  | -2.844 | 0.507 | -5.611 | 0 | 0 |
| ENSG00000281376 | ABALON | -2.844 | 0.39 | -7.299 | 0 | 0 |
| ENSG00000243279 | PRAF2 | -2.84 | 0.357 | -7.955 | 0 | 0 |
| ENSG00000228397 | LINC01635 | -2.839 | 1.2 | -2.366 | 0.018 | 0.0458 |
| ENSG00000223660 |  | -2.836 | 0.787 | -3.604 | 0.0003 | 0.0015 |
| ENSG00000075043 | KCNQ2 | -2.835 | 0.576 | -4.919 | 0 | 0 |
| ENSG00000234807 |  | -2.835 | 0.954 | -2.973 | 0.0029 | 0.0102 |
| ENSG00000283160 | MIR4521 | -2.833 | 0.672 | -4.216 | 0 | 0.0002 |
| ENSG00000224939 | LINC00184 | -2.832 | 0.518 | -5.469 | 0 | 0 |
| ENSG00000271788 |  | -2.83 | 0.379 | -7.459 | 0 | 0 |
| ENSG00000228189 | LINC02843 | -2.83 | 0.683 | -4.146 | 0 | 0.0002 |
| ENSG00000126353 | CCR7 | -2.828 | 0.341 | -8.284 | 0 | 0 |
| ENSG00000261386 |  | -2.827 | 0.629 | -4.492 | 0 | 0.0001 |
| ENSG00000264229 | RNU4ATAC | -2.826 | 0.515 | -5.486 | 0 | 0 |
| ENSG00000267064 | UXT-AS1 | -2.826 | 0.511 | -5.535 | 0 | 0 |
| ENSG00000179862 | CITED4 | -2.826 | 0.512 | -5.521 | 0 | 0 |
| ENSG00000278862 |  | -2.823 | 1.119 | -2.523 | 0.0116 | 0.0319 |
| ENSG00000230679 | ENO1-AS1 | -2.819 | 0.587 | -4.801 | 0 | 0 |
| ENSG00000260669 |  | -2.818 | 0.57 | -4.943 | 0 | 0 |
| ENSG00000090530 | P3H2 | -2.818 | 0.363 | -7.775 | 0 | 0 |
| ENSG00000213471 | TTLL13P | -2.816 | 0.681 | -4.134 | 0 | 0.0002 |
| ENSG00000249186 |  | -2.816 | 0.877 | -3.212 | 0.0013 | 0.0052 |
| ENSG00000196141 | SPATS2L | -2.815 | 0.249 | -11.299 | 0 | 0 |
| ENSG00000269961 |  | -2.813 | 0.398 | -7.058 | 0 | 0 |
| ENSG00000091181 | IL5RA | -2.809 | 1.13 | -2.486 | 0.0129 | 0.0348 |
| ENSG00000232860 | SMG7-AS1 | -2.806 | 0.347 | -8.074 | 0 | 0 |
| ENSG00000188706 | ZDHHC9 | -2.805 | 0.257 | -10.904 | 0 | 0 |
| ENSG00000170412 | GPRC5C | -2.804 | 0.685 | -4.091 | 0 | 0.0003 |
| ENSG00000235852 |  | -2.796 | 0.477 | -5.855 | 0 | 0 |
| ENSG00000163995 | ABLIM2 | -2.794 | 0.768 | -3.636 | 0.0003 | 0.0014 |
| ENSG00000206199 | ANKUB1 | -2.791 | 0.479 | -5.826 | 0 | 0 |
| ENSG00000234134 |  | -2.791 | 0.657 | -4.251 | 0 | 0.0001 |
| ENSG00000249357 |  | -2.791 | 0.812 | -3.438 | 0.0006 | 0.0026 |
| ENSG00000258808 | LINC02310 | -2.788 | 0.444 | -6.285 | 0 | 0 |
| ENSG00000049883 | PTCD2 | -2.784 | 0.257 | -10.832 | 0 | 0 |
| ENSG00000261335 | LOC105274304 | -2.782 | 0.579 | -4.805 | 0 | 0 |
| ENSG00000007376 | RPUSD1 | -2.778 | 0.274 | -10.147 | 0 | 0 |
| ENSG00000111344 | RASAL1 | -2.777 | 0.923 | -3.008 | 0.0026 | 0.0092 |
| ENSG00000280620 | SCAANT1 | -2.773 | 0.618 | -4.489 | 0 | 0.0001 |
| ENSG00000274367 |  | -2.773 | 0.93 | -2.982 | 0.0029 | 0.0099 |
| ENSG00000228549 | LOC112267871 | -2.771 | 0.549 | -5.052 | 0 | 0 |
| ENSG00000088827 | SIGLEC1 | -2.768 | 0.562 | -4.928 | 0 | 0 |
| ENSG00000265342 |  | -2.767 | 0.861 | -3.215 | 0.0013 | 0.0051 |
| ENSG00000274698 |  | -2.767 | 0.958 | -2.889 | 0.0039 | 0.0128 |
| ENSG00000146090 | RASGEF1C | -2.765 | 0.61 | -4.531 | 0 | 0 |
| ENSG00000130881 | LRP3 | -2.76 | 0.301 | -9.164 | 0 | 0 |
| ENSG00000269971 |  | -2.76 | 0.655 | -4.216 | 0 | 0.0002 |
| ENSG00000226312 | CFLAR-AS1 | -2.759 | 0.269 | -10.25 | 0 | 0 |
| ENSG00000183496 | MEX3B | -2.755 | 0.243 | -11.314 | 0 | 0 |
| ENSG00000260081 | LOC105373383 | -2.754 | 0.493 | -5.581 | 0 | 0 |
| ENSG00000258789 |  | -2.754 | 0.395 | -6.977 | 0 | 0 |
| ENSG00000214708 | LOC105371730 | -2.753 | 0.523 | -5.263 | 0 | 0 |
| ENSG00000250657 |  | -2.753 | 0.692 | -3.98 | 0.0001 | 0.0004 |
| ENSG00000224323 |  | -2.751 | 0.702 | -3.92 | 0.0001 | 0.0005 |
| ENSG00000239677 |  | -2.75 | 0.6 | -4.584 | 0 | 0 |
| ENSG00000049283 | EPN3 | -2.75 | 0.61 | -4.509 | 0 | 0.0001 |
| ENSG00000237972 | TUBG1P | -2.75 | 0.74 | -3.718 | 0.0002 | 0.001 |
| ENSG00000279259 |  | -2.747 | 0.325 | -8.461 | 0 | 0 |
| ENSG00000267532 | MIR497HG | -2.747 | 0.655 | -4.191 | 0 | 0.0002 |
| ENSG00000241014 | GPR199P | -2.744 | 0.508 | -5.398 | 0 | 0 |
| ENSG00000118946 | PCDH17 | -2.744 | 0.773 | -3.548 | 0.0004 | 0.0018 |
| ENSG00000278969 |  | -2.742 | 0.71 | -3.859 | 0.0001 | 0.0006 |
| ENSG00000170442 | KRT86 | -2.739 | 0.815 | -3.361 | 0.0008 | 0.0033 |
| ENSG00000187957 | DNER | -2.738 | 0.778 | -3.518 | 0.0004 | 0.002 |
| ENSG00000248371 | LINC02056 | -2.734 | 0.598 | -4.574 | 0 | 0 |
| ENSG00000259804 | LOC107984813 | -2.732 | 0.687 | -3.976 | 0.0001 | 0.0004 |
| ENSG00000223695 |  | -2.732 | 1.009 | -2.709 | 0.0067 | 0.0204 |
| ENSG00000260360 |  | -2.728 | 0.489 | -5.58 | 0 | 0 |
| ENSG00000267248 |  | -2.727 | 0.43 | -6.347 | 0 | 0 |
| ENSG00000172404 | DNAJB7 | -2.727 | 0.834 | -3.269 | 0.0011 | 0.0043 |
| ENSG00000249572 |  | -2.726 | 0.426 | -6.404 | 0 | 0 |
| ENSG00000261186 | LINC01238 | -2.725 | 0.903 | -3.016 | 0.0026 | 0.009 |
| ENSG00000228158 |  | -2.721 | 0.965 | -2.82 | 0.0048 | 0.0154 |
| ENSG00000252213 | SNORA74D | -2.717 | 0.879 | -3.091 | 0.002 | 0.0073 |
| ENSG00000236908 |  | -2.716 | 0.781 | -3.476 | 0.0005 | 0.0023 |
| ENSG00000267530 | LINC01836 | -2.714 | 0.624 | -4.346 | 0 | 0.0001 |
| ENSG00000251445 |  | -2.713 | 0.794 | -3.415 | 0.0006 | 0.0028 |
| ENSG00000245248 |  | -2.712 | 0.771 | -3.519 | 0.0004 | 0.002 |
| ENSG00000267736 |  | -2.71 | 0.858 | -3.156 | 0.0016 | 0.0061 |
| ENSG00000163466 | ARPC2 | -2.707 | 0.269 | -10.061 | 0 | 0 |
| ENSG00000262003 | LOC101927727 | -2.704 | 0.278 | -9.722 | 0 | 0 |
| ENSG00000271869 |  | -2.702 | 0.346 | -7.817 | 0 | 0 |
| ENSG00000078967 | UBE2D4 | -2.702 | 0.374 | -7.219 | 0 | 0 |
| ENSG00000230844 | ZNF674-AS1 | -2.701 | 0.231 | -11.668 | 0 | 0 |
| ENSG00000236364 |  | -2.701 | 0.573 | -4.711 | 0 | 0 |
| ENSG00000223764 | LINC02593 | -2.701 | 0.48 | -5.627 | 0 | 0 |
| ENSG00000155893 | PXYLP1 | -2.701 | 0.242 | -11.148 | 0 | 0 |
| ENSG00000266920 |  | -2.7 | 1.07 | -2.522 | 0.0117 | 0.032 |
| ENSG00000146839 | ZAN | -2.699 | 1.09 | -2.476 | 0.0133 | 0.0356 |
| ENSG00000231160 |  | -2.698 | 0.514 | -5.252 | 0 | 0 |
| ENSG00000232359 |  | -2.695 | 0.538 | -5.011 | 0 | 0 |
| ENSG00000141431 | ASXL3 | -2.695 | 0.788 | -3.419 | 0.0006 | 0.0027 |
| ENSG00000277152 |  | -2.692 | 0.587 | -4.588 | 0 | 0 |
| ENSG00000165462 | PHOX2A | -2.692 | 0.968 | -2.781 | 0.0054 | 0.017 |
| ENSG00000267493 | CIRBP-AS1 | -2.691 | 0.33 | -8.16 | 0 | 0 |
| ENSG00000126368 | NR1D1 | -2.688 | 0.274 | -9.804 | 0 | 0 |
| ENSG00000245468 |  | -2.688 | 0.853 | -3.153 | 0.0016 | 0.0061 |
| ENSG00000104738 | MCM4 | -2.684 | 0.251 | -10.682 | 0 | 0 |
| ENSG00000231327 | LINC01816 | -2.682 | 0.491 | -5.46 | 0 | 0 |
| ENSG00000258940 | LOC100288846 | -2.679 | 0.559 | -4.794 | 0 | 0 |
| ENSG00000145431 | PDGFC | -2.678 | 0.262 | -10.222 | 0 | 0 |
| ENSG00000251359 | WWC2-AS2 | -2.675 | 0.362 | -7.385 | 0 | 0 |
| ENSG00000227071 | FOCAD-AS1 | -2.675 | 0.939 | -2.849 | 0.0044 | 0.0142 |
| ENSG00000239739 |  | -2.674 | 0.673 | -3.971 | 0.0001 | 0.0004 |
| ENSG00000268093 |  | -2.673 | 0.83 | -3.222 | 0.0013 | 0.005 |
| ENSG00000272843 |  | -2.672 | 0.451 | -5.922 | 0 | 0 |
| ENSG00000262202 |  | -2.672 | 0.431 | -6.2 | 0 | 0 |
| ENSG00000274092 |  | -2.672 | 0.714 | -3.744 | 0.0002 | 0.0009 |
| ENSG00000255867 | DENND5B-AS1 | -2.671 | 0.796 | -3.354 | 0.0008 | 0.0034 |
| ENSG00000155324 | GRAMD2B | -2.67 | 0.316 | -8.46 | 0 | 0 |
| ENSG00000237828 |  | -2.665 | 1.073 | -2.485 | 0.013 | 0.0349 |
| ENSG00000251669 | FAM86EP | -2.664 | 0.44 | -6.06 | 0 | 0 |
| ENSG00000173267 | SNCG | -2.664 | 0.375 | -7.097 | 0 | 0 |
| ENSG00000238057 | ZEB2-AS1 | -2.664 | 0.702 | -3.797 | 0.0001 | 0.0008 |
| ENSG00000270058 |  | -2.663 | 0.397 | -6.703 | 0 | 0 |
| ENSG00000257496 |  | -2.663 | 0.46 | -5.785 | 0 | 0 |
| ENSG00000239490 | RPS4XP18 | -2.663 | 0.558 | -4.772 | 0 | 0 |
| ENSG00000206043 | C18orf63 | -2.663 | 1.005 | -2.649 | 0.0081 | 0.0237 |
| ENSG00000237310 | GS1-124K5.4 | -2.657 | 0.45 | -5.906 | 0 | 0 |
| ENSG00000239705 |  | -2.657 | 0.605 | -4.395 | 0 | 0.0001 |
| ENSG00000204228 | HSD17B8 | -2.656 | 0.497 | -5.35 | 0 | 0 |
| ENSG00000283376 |  | -2.656 | 0.81 | -3.279 | 0.001 | 0.0042 |
| ENSG00000237172 | B3GNT9 | -2.654 | 0.248 | -10.711 | 0 | 0 |
| ENSG00000279964 |  | -2.654 | 0.73 | -3.633 | 0.0003 | 0.0014 |
| ENSG00000259802 |  | -2.653 | 0.315 | -8.415 | 0 | 0 |
| ENSG00000272853 |  | -2.652 | 0.353 | -7.508 | 0 | 0 |
| ENSG00000270141 |  | -2.652 | 0.671 | -3.953 | 0.0001 | 0.0005 |
| ENSG00000231789 | PIK3CD-AS2 | -2.649 | 0.907 | -2.919 | 0.0035 | 0.0118 |
| ENSG00000225335 |  | -2.645 | 0.41 | -6.455 | 0 | 0 |
| ENSG00000219992 |  | -2.644 | 0.406 | -6.507 | 0 | 0 |
| ENSG00000236449 |  | -2.641 | 0.64 | -4.13 | 0 | 0.0002 |
| ENSG00000145703 | IQGAP2 | -2.64 | 0.436 | -6.049 | 0 | 0 |
| ENSG00000156869 | FRRS1 | -2.64 | 0.431 | -6.129 | 0 | 0 |
| ENSG00000161405 | IKZF3 | -2.64 | 0.404 | -6.529 | 0 | 0 |
| ENSG00000089220 | PEBP1 | -2.639 | 0.212 | -12.435 | 0 | 0 |
| ENSG00000260274 |  | -2.638 | 0.318 | -8.294 | 0 | 0 |
| ENSG00000235092 |  | -2.637 | 0.646 | -4.08 | 0 | 0.0003 |
| ENSG00000261272 | MUC22 | -2.629 | 0.625 | -4.206 | 0 | 0.0002 |
| ENSG00000224046 | LOC101927420 | -2.626 | 0.371 | -7.079 | 0 | 0 |
| ENSG00000170276 | HSPB2 | -2.621 | 0.82 | -3.195 | 0.0014 | 0.0054 |
| ENSG00000280367 |  | -2.615 | 0.917 | -2.851 | 0.0044 | 0.0142 |
| ENSG00000233058 |  | -2.614 | 0.511 | -5.114 | 0 | 0 |
| ENSG00000280273 |  | -2.611 | 0.5 | -5.226 | 0 | 0 |
| ENSG00000259775 |  | -2.611 | 0.565 | -4.625 | 0 | 0 |
| ENSG00000101470 | TNNC2 | -2.611 | 0.815 | -3.204 | 0.0014 | 0.0053 |
| ENSG00000279329 |  | -2.604 | 0.479 | -5.437 | 0 | 0 |
| ENSG00000230699 |  | -2.602 | 0.601 | -4.332 | 0 | 0.0001 |
| ENSG00000135211 | TMEM60 | -2.599 | 0.262 | -9.913 | 0 | 0 |
| ENSG00000231999 | LRRC8C-DT | -2.597 | 0.747 | -3.477 | 0.0005 | 0.0023 |
| ENSG00000272341 |  | -2.596 | 0.654 | -3.966 | 0.0001 | 0.0004 |
| ENSG00000237788 |  | -2.594 | 0.431 | -6.024 | 0 | 0 |
| ENSG00000224165 | DNAJC27-AS1 | -2.593 | 0.591 | -4.388 | 0 | 0.0001 |
| ENSG00000167914 | GSDMA | -2.593 | 0.644 | -4.028 | 0.0001 | 0.0003 |
| ENSG00000272948 |  | -2.591 | 0.508 | -5.098 | 0 | 0 |
| ENSG00000162571 | TTLL10 | -2.591 | 0.529 | -4.899 | 0 | 0 |
| ENSG00000259523 |  | -2.59 | 0.327 | -7.932 | 0 | 0 |
| ENSG00000140465 | CYP1A1 | -2.588 | 0.702 | -3.686 | 0.0002 | 0.0012 |
| ENSG00000255992 |  | -2.587 | 0.749 | -3.454 | 0.0006 | 0.0025 |
| ENSG00000229539 |  | -2.585 | 0.618 | -4.184 | 0 | 0.0002 |
| ENSG00000272501 |  | -2.584 | 0.367 | -7.034 | 0 | 0 |
| ENSG00000217236 | SP9 | -2.582 | 0.49 | -5.264 | 0 | 0 |
| ENSG00000236514 |  | -2.582 | 0.588 | -4.389 | 0 | 0.0001 |
| ENSG00000200169 | RNU5D-1 | -2.58 | 0.625 | -4.128 | 0 | 0.0002 |
| ENSG00000272892 |  | -2.572 | 0.469 | -5.484 | 0 | 0 |
| ENSG00000261202 | LOC114841040 | -2.572 | 0.615 | -4.182 | 0 | 0.0002 |
| ENSG00000207980 | MIR23A | -2.571 | 0.584 | -4.4 | 0 | 0.0001 |
| ENSG00000278709 | NKILA | -2.569 | 0.583 | -4.404 | 0 | 0.0001 |
| ENSG00000100427 | MLC1 | -2.567 | 0.741 | -3.466 | 0.0005 | 0.0024 |
| ENSG00000172794 | RAB37 | -2.566 | 0.733 | -3.499 | 0.0005 | 0.0021 |
| ENSG00000125968 | ID1 | -2.56 | 0.368 | -6.954 | 0 | 0 |
| ENSG00000272205 |  | -2.56 | 0.728 | -3.515 | 0.0004 | 0.002 |
| ENSG00000262074 |  | -2.559 | 0.467 | -5.48 | 0 | 0 |
| ENSG00000281005 | LINC00921 | -2.559 | 0.428 | -5.98 | 0 | 0 |
| ENSG00000243953 | LOC105374171 | -2.558 | 1.032 | -2.478 | 0.0132 | 0.0354 |
| ENSG00000197670 |  | -2.557 | 0.454 | -5.639 | 0 | 0 |
| ENSG00000271551 | LOC115308161 | -2.557 | 0.826 | -3.095 | 0.002 | 0.0072 |
| ENSG00000242242 |  | -2.556 | 0.638 | -4.009 | 0.0001 | 0.0004 |
| ENSG00000247199 | LOC102546294 | -2.555 | 0.536 | -4.766 | 0 | 0 |
| ENSG00000060718 | COL11A1 | -2.551 | 0.477 | -5.346 | 0 | 0 |
| ENSG00000281450 | PANDAR | -2.551 | 0.873 | -2.922 | 0.0035 | 0.0117 |
| ENSG00000274251 |  | -2.55 | 0.535 | -4.769 | 0 | 0 |
| ENSG00000257910 |  | -2.55 | 0.585 | -4.359 | 0 | 0.0001 |
| ENSG00000273162 |  | -2.55 | 0.769 | -3.314 | 0.0009 | 0.0038 |
| ENSG00000279494 |  | -2.548 | 0.662 | -3.847 | 0.0001 | 0.0007 |
| ENSG00000236036 | LINC00445 | -2.548 | 0.888 | -2.871 | 0.0041 | 0.0135 |
| ENSG00000232926 | RPL8P5 | -2.547 | 0.524 | -4.857 | 0 | 0 |
| ENSG00000253716 | MINCR | -2.546 | 0.278 | -9.147 | 0 | 0 |
| ENSG00000260369 |  | -2.545 | 0.295 | -8.612 | 0 | 0 |
| ENSG00000111348 | ARHGDIB | -2.544 | 0.523 | -4.859 | 0 | 0 |
| ENSG00000160752 | FDPS | -2.544 | 0.325 | -7.828 | 0 | 0 |
| ENSG00000280316 |  | -2.541 | 0.825 | -3.08 | 0.0021 | 0.0075 |
| ENSG00000137267 | TUBB2A | -2.54 | 0.229 | -11.091 | 0 | 0 |
| ENSG00000270640 |  | -2.54 | 0.771 | -3.295 | 0.001 | 0.004 |
| ENSG00000214248 |  | -2.538 | 0.341 | -7.438 | 0 | 0 |
| ENSG00000233421 | LINC01783 | -2.538 | 0.623 | -4.073 | 0 | 0.0003 |
| ENSG00000232310 | LOC101928304 | -2.537 | 0.519 | -4.883 | 0 | 0 |
| ENSG00000188493 | C19orf54 | -2.536 | 0.326 | -7.781 | 0 | 0 |
| ENSG00000224063 |  | -2.533 | 0.724 | -3.498 | 0.0005 | 0.0021 |
| ENSG00000275560 |  | -2.532 | 0.611 | -4.141 | 0 | 0.0002 |
| ENSG00000224969 |  | -2.529 | 0.5 | -5.055 | 0 | 0 |
| ENSG00000182648 | LINC01006 | -2.529 | 0.259 | -9.755 | 0 | 0 |
| ENSG00000275017 |  | -2.529 | 0.932 | -2.712 | 0.0067 | 0.0203 |
| ENSG00000227848 |  | -2.527 | 0.444 | -5.695 | 0 | 0 |
| ENSG00000179222 | MAGED1 | -2.523 | 0.283 | -8.92 | 0 | 0 |
| ENSG00000276633 |  | -2.52 | 0.76 | -3.318 | 0.0009 | 0.0038 |
| ENSG00000171532 | NEUROD2 | -2.519 | 0.499 | -5.051 | 0 | 0 |
| ENSG00000283654 | LMLN2 | -2.517 | 0.44 | -5.725 | 0 | 0 |
| ENSG00000254703 | SENCR | -2.517 | 0.734 | -3.432 | 0.0006 | 0.0026 |
| ENSG00000132855 | ANGPTL3 | -2.516 | 0.729 | -3.452 | 0.0006 | 0.0025 |
| ENSG00000249690 | LOC729558 | -2.515 | 0.847 | -2.97 | 0.003 | 0.0102 |
| ENSG00000275538 | RNVU1-19 | -2.512 | 0.556 | -4.517 | 0 | 0.0001 |
| ENSG00000261117 |  | -2.512 | 0.922 | -2.724 | 0.0064 | 0.0197 |
| ENSG00000272273 | IER3-AS1 | -2.51 | 0.411 | -6.111 | 0 | 0 |
| ENSG00000089127 | OAS1 | -2.505 | 0.467 | -5.366 | 0 | 0 |
| ENSG00000270362 | HMGN3-AS1 | -2.505 | 0.347 | -7.22 | 0 | 0 |
| ENSG00000086696 | HSD17B2 | -2.504 | 0.577 | -4.343 | 0 | 0.0001 |
| ENSG00000274210 |  | -2.494 | 0.535 | -4.662 | 0 | 0 |
| ENSG00000169903 | TM4SF4 | -2.494 | 0.841 | -2.965 | 0.003 | 0.0104 |
| ENSG00000270638 |  | -2.492 | 0.421 | -5.921 | 0 | 0 |
| ENSG00000279086 |  | -2.49 | 0.308 | -8.072 | 0 | 0 |
| ENSG00000276651 |  | -2.49 | 0.512 | -4.867 | 0 | 0 |
| ENSG00000241269 |  | -2.49 | 0.587 | -4.244 | 0 | 0.0002 |
| ENSG00000169297 | NR0B1 | -2.485 | 0.834 | -2.981 | 0.0029 | 0.0099 |
| ENSG00000251301 | LINC02384 | -2.485 | 0.876 | -2.835 | 0.0046 | 0.0148 |
| ENSG00000243710 | CFAP57 | -2.483 | 0.549 | -4.519 | 0 | 0 |
| ENSG00000267882 | LOC100131496 | -2.483 | 0.677 | -3.67 | 0.0002 | 0.0012 |
| ENSG00000164442 | CITED2 | -2.482 | 0.211 | -11.779 | 0 | 0 |
| ENSG00000273702 |  | -2.482 | 0.259 | -9.581 | 0 | 0 |
| ENSG00000140873 | ADAMTS18 | -2.482 | 0.555 | -4.472 | 0 | 0.0001 |
| ENSG00000266975 | FARSA-AS1 | -2.48 | 0.608 | -4.083 | 0 | 0.0003 |
| ENSG00000272969 |  | -2.48 | 0.724 | -3.426 | 0.0006 | 0.0027 |
| ENSG00000272572 |  | -2.479 | 0.614 | -4.039 | 0.0001 | 0.0003 |
| ENSG00000234230 | ZFX-AS1 | -2.478 | 0.841 | -2.947 | 0.0032 | 0.0109 |
| ENSG00000224790 |  | -2.475 | 0.7 | -3.536 | 0.0004 | 0.0019 |
| ENSG00000228288 | PCAT6 | -2.473 | 0.619 | -3.997 | 0.0001 | 0.0004 |
| ENSG00000260572 |  | -2.472 | 0.435 | -5.686 | 0 | 0 |
| ENSG00000271614 | ATP2B1-AS1 | -2.472 | 0.277 | -8.929 | 0 | 0 |
| ENSG00000256083 |  | -2.469 | 0.478 | -5.169 | 0 | 0 |
| ENSG00000250803 | LOC100505841 | -2.469 | 0.48 | -5.139 | 0 | 0 |
| ENSG00000188263 | IL17REL | -2.469 | 0.555 | -4.451 | 0 | 0.0001 |
| ENSG00000204929 | LOC101927533 | -2.466 | 0.807 | -3.055 | 0.0023 | 0.0081 |
| ENSG00000260288 |  | -2.465 | 0.733 | -3.363 | 0.0008 | 0.0033 |
| ENSG00000250770 |  | -2.464 | 0.397 | -6.207 | 0 | 0 |
| ENSG00000164379 | FOXQ1 | -2.464 | 0.322 | -7.647 | 0 | 0 |
| ENSG00000270964 |  | -2.463 | 0.452 | -5.451 | 0 | 0 |
| ENSG00000270673 | YTHDF3-AS1 | -2.463 | 0.341 | -7.226 | 0 | 0 |
| ENSG00000198626 | RYR2 | -2.462 | 0.924 | -2.664 | 0.0077 | 0.0228 |
| ENSG00000214402 | LCNL1 | -2.462 | 1.023 | -2.407 | 0.0161 | 0.0417 |
| ENSG00000144642 | RBMS3 | -2.461 | 0.279 | -8.814 | 0 | 0 |
| ENSG00000267632 |  | -2.46 | 0.813 | -3.026 | 0.0025 | 0.0087 |
| ENSG00000119669 | IRF2BPL | -2.459 | 0.262 | -9.393 | 0 | 0 |
| ENSG00000267939 |  | -2.458 | 0.854 | -2.879 | 0.004 | 0.0132 |
| ENSG00000141738 | GRB7 | -2.457 | 0.42 | -5.848 | 0 | 0 |
| ENSG00000231711 |  | -2.457 | 0.322 | -7.626 | 0 | 0 |
| ENSG00000244953 |  | -2.457 | 0.855 | -2.874 | 0.0041 | 0.0134 |
| ENSG00000117318 | ID3 | -2.452 | 0.29 | -8.451 | 0 | 0 |
| ENSG00000268592 | RAET1E-AS1 | -2.452 | 0.903 | -2.715 | 0.0066 | 0.0202 |
| ENSG00000226334 |  | -2.451 | 0.844 | -2.902 | 0.0037 | 0.0123 |
| ENSG00000123700 | KCNJ2 | -2.45 | 0.355 | -6.898 | 0 | 0 |
| ENSG00000125531 | FNDC11 | -2.45 | 0.559 | -4.381 | 0 | 0.0001 |
| ENSG00000273253 |  | -2.449 | 0.674 | -3.634 | 0.0003 | 0.0014 |
| ENSG00000178235 | SLITRK1 | -2.449 | 0.714 | -3.431 | 0.0006 | 0.0026 |
| ENSG00000113946 | CLDN16 | -2.449 | 0.854 | -2.867 | 0.0041 | 0.0136 |
| ENSG00000131944 | FAAP24 | -2.448 | 0.403 | -6.071 | 0 | 0 |
| ENSG00000126803 | HSPA2 | -2.447 | 0.265 | -9.238 | 0 | 0 |
| ENSG00000172969 | FRG2C | -2.446 | 0.977 | -2.504 | 0.0123 | 0.0334 |
| ENSG00000223722 |  | -2.446 | 1.035 | -2.363 | 0.0181 | 0.0461 |
| ENSG00000221821 | C6orf226 | -2.445 | 0.262 | -9.326 | 0 | 0 |
| ENSG00000182195 | LDOC1 | -2.442 | 0.342 | -7.14 | 0 | 0 |
| ENSG00000112773 | TENT5A | -2.438 | 0.228 | -10.701 | 0 | 0 |
| ENSG00000271966 |  | -2.438 | 0.53 | -4.6 | 0 | 0 |
| ENSG00000182366 | FAM87A | -2.437 | 0.924 | -2.638 | 0.0083 | 0.0243 |
| ENSG00000271040 |  | -2.436 | 0.963 | -2.53 | 0.0114 | 0.0314 |
| ENSG00000222020 | HDAC4-AS1 | -2.434 | 0.382 | -6.378 | 0 | 0 |
| ENSG00000272106 |  | -2.433 | 0.339 | -7.167 | 0 | 0 |
| ENSG00000103707 | MTFMT | -2.433 | 0.29 | -8.387 | 0 | 0 |
| ENSG00000280040 |  | -2.43 | 0.939 | -2.587 | 0.0097 | 0.0274 |
| ENSG00000219200 | RNASEK | -2.428 | 0.431 | -5.636 | 0 | 0 |
| ENSG00000111276 | CDKN1B | -2.428 | 0.23 | -10.546 | 0 | 0 |
| ENSG00000227199 | ST7-AS1 | -2.428 | 0.615 | -3.945 | 0.0001 | 0.0005 |
| ENSG00000227039 | ITGB2-AS1 | -2.426 | 0.769 | -3.156 | 0.0016 | 0.0061 |
| ENSG00000183291 | SELENOF | -2.424 | 0.262 | -9.266 | 0 | 0 |
| ENSG00000258457 | LOC107984660 | -2.423 | 0.503 | -4.819 | 0 | 0 |
| ENSG00000227262 |  | -2.422 | 0.796 | -3.041 | 0.0024 | 0.0084 |
| ENSG00000228451 | SDAD1P1 | -2.421 | 0.295 | -8.216 | 0 | 0 |
| ENSG00000279817 |  | -2.421 | 0.915 | -2.647 | 0.0081 | 0.0238 |
| ENSG00000142609 | CFAP74 | -2.42 | 0.695 | -3.481 | 0.0005 | 0.0023 |
| ENSG00000274054 | MIR4727 | -2.42 | 0.921 | -2.627 | 0.0086 | 0.0249 |
| ENSG00000258597 | SERPINA2 | -2.419 | 0.737 | -3.28 | 0.001 | 0.0042 |
| ENSG00000172292 | CERS6 | -2.418 | 0.322 | -7.518 | 0 | 0 |
| ENSG00000272430 |  | -2.417 | 0.859 | -2.814 | 0.0049 | 0.0156 |
| ENSG00000106211 | HSPB1 | -2.416 | 0.255 | -9.457 | 0 | 0 |
| ENSG00000266904 |  | -2.416 | 0.533 | -4.537 | 0 | 0 |
| ENSG00000136689 | IL1RN | -2.416 | 0.554 | -4.363 | 0 | 0.0001 |
| ENSG00000175348 | TMEM9B | -2.414 | 0.299 | -8.071 | 0 | 0 |
| ENSG00000096092 | TMEM14A | -2.414 | 0.333 | -7.26 | 0 | 0 |
| ENSG00000270959 | LPP-AS2 | -2.413 | 0.281 | -8.579 | 0 | 0 |
| ENSG00000103381 | CPPED1 | -2.413 | 0.299 | -8.058 | 0 | 0 |
| ENSG00000268584 |  | -2.409 | 0.38 | -6.335 | 0 | 0 |
| ENSG00000235831 | BHLHE40-AS1 | -2.409 | 0.849 | -2.839 | 0.0045 | 0.0146 |
| ENSG00000266306 |  | -2.407 | 0.473 | -5.083 | 0 | 0 |
| ENSG00000259727 |  | -2.403 | 0.712 | -3.374 | 0.0007 | 0.0032 |
| ENSG00000164187 | LMBRD2 | -2.402 | 0.325 | -7.387 | 0 | 0 |
| ENSG00000280417 |  | -2.401 | 0.649 | -3.698 | 0.0002 | 0.0011 |
| ENSG00000162241 | SLC25A45 | -2.4 | 0.288 | -8.342 | 0 | 0 |
| ENSG00000279901 |  | -2.4 | 0.503 | -4.768 | 0 | 0 |
| ENSG00000277182 |  | -2.4 | 0.467 | -5.139 | 0 | 0 |
| ENSG00000111331 | OAS3 | -2.397 | 0.279 | -8.588 | 0 | 0 |
| ENSG00000277581 |  | -2.397 | 0.644 | -3.72 | 0.0002 | 0.001 |
| ENSG00000273084 |  | -2.396 | 0.361 | -6.63 | 0 | 0 |
| ENSG00000105011 | ASF1B | -2.395 | 0.235 | -10.203 | 0 | 0 |
| ENSG00000198673 | TAFA2 | -2.395 | 0.409 | -5.852 | 0 | 0 |
| ENSG00000111339 | ART4 | -2.395 | 0.768 | -3.119 | 0.0018 | 0.0067 |
| ENSG00000069482 | GAL | -2.393 | 0.635 | -3.768 | 0.0002 | 0.0009 |
| ENSG00000156097 | GPR61 | -2.385 | 0.304 | -7.845 | 0 | 0 |
| ENSG00000265799 |  | -2.384 | 0.615 | -3.877 | 0.0001 | 0.0006 |
| ENSG00000250303 | LINC02762 | -2.383 | 0.246 | -9.688 | 0 | 0 |
| ENSG00000239455 |  | -2.381 | 0.699 | -3.405 | 0.0007 | 0.0029 |
| ENSG00000275454 |  | -2.381 | 0.718 | -3.317 | 0.0009 | 0.0038 |
| ENSG00000271918 |  | -2.38 | 0.348 | -6.836 | 0 | 0 |
| ENSG00000117600 | PLPPR4 | -2.38 | 0.63 | -3.78 | 0.0002 | 0.0008 |
| ENSG00000139433 | GLTP | -2.379 | 0.23 | -10.359 | 0 | 0 |
| ENSG00000248668 | OXCT1-AS1 | -2.377 | 1.001 | -2.376 | 0.0175 | 0.0448 |
| ENSG00000105404 | RABAC1 | -2.376 | 0.237 | -10.02 | 0 | 0 |
| ENSG00000278952 |  | -2.376 | 0.337 | -7.061 | 0 | 0 |
| ENSG00000258893 |  | -2.376 | 0.806 | -2.946 | 0.0032 | 0.0109 |
| ENSG00000271855 |  | -2.375 | 0.607 | -3.911 | 0.0001 | 0.0005 |
| ENSG00000262587 | LOC554206 | -2.375 | 0.898 | -2.646 | 0.0081 | 0.0238 |
| ENSG00000270504 |  | -2.374 | 0.238 | -9.983 | 0 | 0 |
| ENSG00000272986 |  | -2.373 | 0.671 | -3.535 | 0.0004 | 0.0019 |
| ENSG00000213062 |  | -2.372 | 0.407 | -5.824 | 0 | 0 |
| ENSG00000279283 |  | -2.372 | 0.574 | -4.13 | 0 | 0.0002 |
| ENSG00000272183 |  | -2.367 | 0.398 | -5.942 | 0 | 0 |
| ENSG00000189143 | CLDN4 | -2.364 | 0.566 | -4.179 | 0 | 0.0002 |
| ENSG00000240895 |  | -2.364 | 0.765 | -3.091 | 0.002 | 0.0073 |
| ENSG00000135373 | EHF | -2.361 | 0.43 | -5.496 | 0 | 0 |
| ENSG00000265399 |  | -2.36 | 0.584 | -4.04 | 0.0001 | 0.0003 |
| ENSG00000258987 |  | -2.36 | 1.012 | -2.333 | 0.0197 | 0.0492 |
| ENSG00000227542 |  | -2.355 | 0.586 | -4.017 | 0.0001 | 0.0004 |
| ENSG00000126264 | HCST | -2.354 | 0.991 | -2.376 | 0.0175 | 0.0448 |
| ENSG00000123095 | BHLHE41 | -2.352 | 0.354 | -6.644 | 0 | 0 |
| ENSG00000237021 |  | -2.351 | 0.503 | -4.672 | 0 | 0 |
| ENSG00000248590 |  | -2.351 | 0.632 | -3.722 | 0.0002 | 0.001 |
| ENSG00000167011 | NAT16 | -2.348 | 0.703 | -3.343 | 0.0008 | 0.0035 |
| ENSG00000272195 |  | -2.347 | 0.515 | -4.556 | 0 | 0 |
| ENSG00000105088 | OLFM2 | -2.347 | 0.58 | -4.049 | 0.0001 | 0.0003 |
| ENSG00000159231 | CBR3 | -2.346 | 0.414 | -5.663 | 0 | 0 |
| ENSG00000007384 | RHBDF1 | -2.345 | 0.264 | -8.865 | 0 | 0 |
| ENSG00000277597 |  | -2.345 | 0.681 | -3.443 | 0.0006 | 0.0025 |
| ENSG00000177551 | NHLH2 | -2.344 | 0.571 | -4.103 | 0 | 0.0003 |
| ENSG00000087274 | ADD1 | -2.341 | 0.248 | -9.438 | 0 | 0 |
| ENSG00000257167 | TMPO-AS1 | -2.339 | 0.359 | -6.508 | 0 | 0 |
| ENSG00000108733 | PEX12 | -2.338 | 0.231 | -10.121 | 0 | 0 |
| ENSG00000254333 | NDST1-AS1 | -2.335 | 0.907 | -2.574 | 0.0101 | 0.0283 |
| ENSG00000215915 | ATAD3C | -2.334 | 0.832 | -2.805 | 0.005 | 0.016 |
| ENSG00000181211 | HECW1-IT1 | -2.333 | 0.865 | -2.698 | 0.007 | 0.021 |
| ENSG00000171864 | PRND | -2.332 | 0.561 | -4.159 | 0 | 0.0002 |
| ENSG00000235204 | LOC613206 | -2.33 | 0.503 | -4.635 | 0 | 0 |
| ENSG00000276931 |  | -2.329 | 0.643 | -3.624 | 0.0003 | 0.0014 |
| ENSG00000249713 | LOC101929109 | -2.329 | 0.759 | -3.068 | 0.0022 | 0.0078 |
| ENSG00000125871 | MGME1 | -2.328 | 0.264 | -8.806 | 0 | 0 |
| ENSG00000174165 | ZDHHC24 | -2.328 | 0.293 | -7.943 | 0 | 0 |
| ENSG00000178700 | DHFR2 | -2.328 | 0.312 | -7.469 | 0 | 0 |
| ENSG00000249709 | ZNF564 | -2.328 | 0.523 | -4.453 | 0 | 0.0001 |
| ENSG00000256721 |  | -2.327 | 0.741 | -3.14 | 0.0017 | 0.0063 |
| ENSG00000275055 |  | -2.326 | 0.654 | -3.555 | 0.0004 | 0.0018 |
| ENSG00000234832 |  | -2.326 | 0.787 | -2.954 | 0.0031 | 0.0107 |
| ENSG00000165953 | SERPINA12 | -2.323 | 0.745 | -3.118 | 0.0018 | 0.0068 |
| ENSG00000253190 |  | -2.322 | 0.275 | -8.443 | 0 | 0 |
| ENSG00000157326 | DHRS4 | -2.32 | 0.42 | -5.521 | 0 | 0 |
| ENSG00000277453 |  | -2.319 | 0.316 | -7.333 | 0 | 0 |
| ENSG00000273382 |  | -2.316 | 0.407 | -5.697 | 0 | 0 |
| ENSG00000204511 | MCCD1 | -2.315 | 0.539 | -4.292 | 0 | 0.0001 |
| ENSG00000227896 |  | -2.315 | 0.687 | -3.369 | 0.0008 | 0.0032 |
| ENSG00000234183 |  | -2.313 | 0.612 | -3.777 | 0.0002 | 0.0008 |
| ENSG00000267658 |  | -2.311 | 0.546 | -4.231 | 0 | 0.0002 |
| ENSG00000261040 | WFDC21P | -2.309 | 0.401 | -5.765 | 0 | 0 |
| ENSG00000254718 | LOC101927702 | -2.307 | 0.76 | -3.035 | 0.0024 | 0.0086 |
| ENSG00000261270 |  | -2.307 | 0.815 | -2.831 | 0.0046 | 0.015 |
| ENSG00000124107 | SLPI | -2.306 | 0.648 | -3.558 | 0.0004 | 0.0018 |
| ENSG00000158270 | COLEC12 | -2.305 | 0.234 | -9.852 | 0 | 0 |
| ENSG00000212724 | KRTAP2-3 | -2.304 | 0.35 | -6.573 | 0 | 0 |
| ENSG00000250934 |  | -2.303 | 0.601 | -3.835 | 0.0001 | 0.0007 |
| ENSG00000260339 | HEXA-AS1 | -2.302 | 0.346 | -6.657 | 0 | 0 |
| ENSG00000147231 | RADX | -2.302 | 0.595 | -3.868 | 0.0001 | 0.0006 |
| ENSG00000258537 | FRMD6-AS2 | -2.302 | 0.662 | -3.475 | 0.0005 | 0.0023 |
| ENSG00000173598 | NUDT4 | -2.298 | 0.275 | -8.373 | 0 | 0 |
| ENSG00000268324 | LRRC2-AS1 | -2.296 | 0.457 | -5.02 | 0 | 0 |
| ENSG00000272017 |  | -2.296 | 0.882 | -2.602 | 0.0093 | 0.0265 |
| ENSG00000213760 | ATP6V1G2 | -2.294 | 0.703 | -3.262 | 0.0011 | 0.0045 |
| ENSG00000236098 |  | -2.294 | 0.917 | -2.503 | 0.0123 | 0.0335 |
| ENSG00000272269 | NUP153-AS1 | -2.293 | 0.449 | -5.105 | 0 | 0 |
| ENSG00000273768 |  | -2.293 | 0.621 | -3.693 | 0.0002 | 0.0011 |
| ENSG00000261192 | RNF126P1 | -2.293 | 0.622 | -3.686 | 0.0002 | 0.0012 |
| ENSG00000257258 |  | -2.293 | 0.791 | -2.897 | 0.0038 | 0.0125 |
| ENSG00000199036 | MIR219A1 | -2.291 | 0.606 | -3.778 | 0.0002 | 0.0008 |
| ENSG00000169271 | HSPB3 | -2.29 | 0.398 | -5.757 | 0 | 0 |
| ENSG00000260727 | SLC7A5P1 | -2.289 | 0.491 | -4.659 | 0 | 0 |
| ENSG00000271334 | LINC02104 | -2.289 | 0.452 | -5.063 | 0 | 0 |
| ENSG00000237737 | DCTN1-AS1 | -2.289 | 0.536 | -4.271 | 0 | 0.0001 |
| ENSG00000272344 | SNORD114-21 | -2.289 | 0.786 | -2.913 | 0.0036 | 0.012 |
| ENSG00000272829 |  | -2.288 | 0.513 | -4.456 | 0 | 0.0001 |
| ENSG00000245498 | LOC100507283 | -2.283 | 0.402 | -5.682 | 0 | 0 |
| ENSG00000205913 | SRRM2-AS1 | -2.283 | 0.315 | -7.244 | 0 | 0 |
| ENSG00000073605 | GSDMB | -2.282 | 0.395 | -5.774 | 0 | 0 |
| ENSG00000230337 | EXOSC10-AS1 | -2.281 | 0.578 | -3.945 | 0.0001 | 0.0005 |
| ENSG00000169851 | PCDH7 | -2.28 | 0.387 | -5.896 | 0 | 0 |
| ENSG00000271938 |  | -2.28 | 0.766 | -2.977 | 0.0029 | 0.0101 |
| ENSG00000205309 | NT5M | -2.278 | 0.561 | -4.061 | 0 | 0.0003 |
| ENSG00000108691 | CCL2 | -2.277 | 0.387 | -5.881 | 0 | 0 |
| ENSG00000223855 | HRAT92 | -2.277 | 0.658 | -3.459 | 0.0005 | 0.0024 |
| ENSG00000264548 |  | -2.274 | 0.607 | -3.749 | 0.0002 | 0.0009 |
| ENSG00000271122 | LOC101930085 | -2.271 | 0.246 | -9.224 | 0 | 0 |
| ENSG00000280025 |  | -2.271 | 0.704 | -3.226 | 0.0013 | 0.005 |
| ENSG00000228201 |  | -2.27 | 0.455 | -4.985 | 0 | 0 |
| ENSG00000254094 |  | -2.27 | 0.517 | -4.391 | 0 | 0.0001 |
| ENSG00000165410 | CFL2 | -2.269 | 0.269 | -8.432 | 0 | 0 |
| ENSG00000161940 | BCL6B | -2.268 | 0.495 | -4.585 | 0 | 0 |
| ENSG00000280187 |  | -2.267 | 0.317 | -7.141 | 0 | 0 |
| ENSG00000234006 | DDX39B-AS1 | -2.267 | 0.592 | -3.829 | 0.0001 | 0.0007 |
| ENSG00000256073 | URB1-AS1 | -2.265 | 0.246 | -9.196 | 0 | 0 |
| ENSG00000116574 | RHOU | -2.265 | 0.77 | -2.942 | 0.0033 | 0.0111 |
| ENSG00000181350 | LRRC75A | -2.263 | 0.332 | -6.817 | 0 | 0 |
| ENSG00000248100 |  | -2.262 | 0.444 | -5.096 | 0 | 0 |
| ENSG00000180096 | SEPTIN1 | -2.262 | 0.526 | -4.301 | 0 | 0.0001 |
| ENSG00000271991 |  | -2.261 | 0.363 | -6.222 | 0 | 0 |
| ENSG00000175699 | CCDC197 | -2.261 | 0.458 | -4.939 | 0 | 0 |
| ENSG00000168887 | C2orf68 | -2.261 | 0.312 | -7.253 | 0 | 0 |
| ENSG00000224358 |  | -2.261 | 0.403 | -5.612 | 0 | 0 |
| ENSG00000141665 | FBXO15 | -2.261 | 0.799 | -2.829 | 0.0047 | 0.015 |
| ENSG00000005206 | SPPL2B | -2.259 | 0.299 | -7.556 | 0 | 0 |
| ENSG00000238121 | LINC00426 | -2.258 | 0.734 | -3.076 | 0.0021 | 0.0076 |
| ENSG00000280309 |  | -2.255 | 0.858 | -2.627 | 0.0086 | 0.0249 |
| ENSG00000225791 | TRAM2-AS1 | -2.253 | 0.299 | -7.526 | 0 | 0 |
| ENSG00000272735 |  | -2.252 | 0.414 | -5.443 | 0 | 0 |
| ENSG00000131899 | LLGL1 | -2.251 | 0.261 | -8.636 | 0 | 0 |
| ENSG00000160318 | CLDND2 | -2.251 | 0.596 | -3.776 | 0.0002 | 0.0008 |
| ENSG00000259488 |  | -2.248 | 0.353 | -6.376 | 0 | 0 |
| ENSG00000238092 |  | -2.248 | 0.501 | -4.485 | 0 | 0.0001 |
| ENSG00000260677 |  | -2.248 | 0.957 | -2.349 | 0.0188 | 0.0474 |
| ENSG00000230555 |  | -2.247 | 0.42 | -5.35 | 0 | 0 |
| ENSG00000228919 | LOC389199 | -2.243 | 0.652 | -3.442 | 0.0006 | 0.0026 |
| ENSG00000112183 | RBM24 | -2.24 | 0.289 | -7.764 | 0 | 0 |
| ENSG00000205808 | PLPP6 | -2.238 | 0.311 | -7.192 | 0 | 0 |
| ENSG00000154914 | USP43 | -2.238 | 0.851 | -2.628 | 0.0086 | 0.0249 |
| ENSG00000182896 | TMEM95 | -2.237 | 0.614 | -3.644 | 0.0003 | 0.0013 |
| ENSG00000179348 | GATA2 | -2.236 | 0.286 | -7.818 | 0 | 0 |
| ENSG00000245975 | LOC101928725 | -2.236 | 0.644 | -3.471 | 0.0005 | 0.0023 |
| ENSG00000257531 |  | -2.236 | 0.718 | -3.115 | 0.0018 | 0.0068 |
| ENSG00000272092 |  | -2.234 | 0.375 | -5.953 | 0 | 0 |
| ENSG00000280132 |  | -2.234 | 0.399 | -5.596 | 0 | 0 |
| ENSG00000279602 |  | -2.232 | 0.332 | -6.727 | 0 | 0 |
| ENSG00000228150 |  | -2.229 | 0.845 | -2.638 | 0.0083 | 0.0243 |
| ENSG00000168264 | IRF2BP2 | -2.228 | 0.21 | -10.613 | 0 | 0 |
| ENSG00000251136 | LOC101929709 | -2.228 | 0.382 | -5.836 | 0 | 0 |
| ENSG00000281189 | GHET1 | -2.227 | 0.751 | -2.964 | 0.003 | 0.0104 |
| ENSG00000275549 | STPG3-AS1 | -2.226 | 0.522 | -4.267 | 0 | 0.0001 |
| ENSG00000279048 |  | -2.222 | 0.3 | -7.411 | 0 | 0 |
| ENSG00000145217 | SLC26A1 | -2.222 | 0.315 | -7.063 | 0 | 0 |
| ENSG00000283073 |  | -2.222 | 0.654 | -3.395 | 0.0007 | 0.003 |
| ENSG00000273321 |  | -2.22 | 0.656 | -3.387 | 0.0007 | 0.003 |
| ENSG00000254236 |  | -2.22 | 0.712 | -3.119 | 0.0018 | 0.0067 |
| ENSG00000263105 |  | -2.219 | 0.671 | -3.305 | 0.0009 | 0.0039 |
| ENSG00000272277 |  | -2.218 | 0.368 | -6.02 | 0 | 0 |
| ENSG00000260625 |  | -2.217 | 0.839 | -2.644 | 0.0082 | 0.0239 |
| ENSG00000237523 | LINC00857 | -2.214 | 0.472 | -4.694 | 0 | 0 |
| ENSG00000187630 | DHRS4L2 | -2.211 | 0.495 | -4.462 | 0 | 0.0001 |
| ENSG00000221869 | CEBPD | -2.21 | 0.249 | -8.871 | 0 | 0 |
| ENSG00000232810 | TNF | -2.207 | 0.631 | -3.496 | 0.0005 | 0.0021 |
| ENSG00000274922 |  | -2.206 | 0.508 | -4.34 | 0 | 0.0001 |
| ENSG00000258858 |  | -2.206 | 0.598 | -3.689 | 0.0002 | 0.0011 |
| ENSG00000258738 | LOC112268124 | -2.205 | 0.437 | -5.041 | 0 | 0 |
| ENSG00000162959 | MEMO1 | -2.204 | 0.55 | -4.008 | 0.0001 | 0.0004 |
| ENSG00000145506 | NKD2 | -2.204 | 0.81 | -2.72 | 0.0065 | 0.0199 |
| ENSG00000272702 |  | -2.203 | 0.353 | -6.232 | 0 | 0 |
| ENSG00000265100 |  | -2.203 | 0.51 | -4.317 | 0 | 0.0001 |
| ENSG00000259442 |  | -2.203 | 0.522 | -4.22 | 0 | 0.0002 |
| ENSG00000224020 | MIR181A2HG | -2.203 | 0.68 | -3.24 | 0.0012 | 0.0048 |
| ENSG00000233426 | EIF3FP3 | -2.203 | 0.921 | -2.393 | 0.0167 | 0.043 |
| ENSG00000183154 | LOC102723701 | -2.202 | 0.628 | -3.508 | 0.0005 | 0.0021 |
| ENSG00000198435 | NRARP | -2.202 | 0.794 | -2.774 | 0.0055 | 0.0173 |
| ENSG00000263011 |  | -2.202 | 0.929 | -2.37 | 0.0178 | 0.0453 |
| ENSG00000140807 | NKD1 | -2.201 | 0.669 | -3.289 | 0.001 | 0.0041 |
| ENSG00000226992 |  | -2.201 | 0.715 | -3.078 | 0.0021 | 0.0075 |
| ENSG00000224132 |  | -2.199 | 0.554 | -3.969 | 0.0001 | 0.0004 |
| ENSG00000225880 | LINC00115 | -2.198 | 0.325 | -6.759 | 0 | 0 |
| ENSG00000175202 | HIGD2B | -2.196 | 0.542 | -4.049 | 0.0001 | 0.0003 |
| ENSG00000231738 | TSPAN19 | -2.196 | 0.794 | -2.765 | 0.0057 | 0.0178 |
| ENSG00000142327 | RNPEPL1 | -2.194 | 0.243 | -9.016 | 0 | 0 |
| ENSG00000169760 | NLGN1 | -2.193 | 0.395 | -5.551 | 0 | 0 |
| ENSG00000259895 | LOC729652 | -2.193 | 0.647 | -3.388 | 0.0007 | 0.003 |
| ENSG00000262115 |  | -2.193 | 0.767 | -2.858 | 0.0043 | 0.0139 |
| ENSG00000164512 | ANKRD55 | -2.191 | 0.739 | -2.963 | 0.003 | 0.0104 |
| ENSG00000245904 |  | -2.19 | 0.47 | -4.658 | 0 | 0 |
| ENSG00000233010 |  | -2.19 | 0.939 | -2.331 | 0.0197 | 0.0494 |
| ENSG00000140043 | PTGR2 | -2.189 | 0.548 | -3.998 | 0.0001 | 0.0004 |
| ENSG00000197588 | KLKP1 | -2.188 | 0.484 | -4.521 | 0 | 0 |
| ENSG00000184897 | H1-10 | -2.186 | 0.235 | -9.302 | 0 | 0 |
| ENSG00000215182 | MUC5AC | -2.186 | 0.446 | -4.899 | 0 | 0 |
| ENSG00000245067 | IGFBP7-AS1 | -2.185 | 0.599 | -3.645 | 0.0003 | 0.0013 |
| ENSG00000230695 |  | -2.185 | 0.604 | -3.618 | 0.0003 | 0.0014 |
| ENSG00000171223 | JUNB | -2.183 | 0.212 | -10.319 | 0 | 0 |
| ENSG00000224460 |  | -2.182 | 0.804 | -2.712 | 0.0067 | 0.0203 |
| ENSG00000266456 |  | -2.179 | 0.514 | -4.241 | 0 | 0.0002 |
| ENSG00000162068 | NTN3 | -2.178 | 0.572 | -3.806 | 0.0001 | 0.0008 |
| ENSG00000279199 |  | -2.177 | 0.456 | -4.776 | 0 | 0 |
| ENSG00000228043 |  | -2.177 | 0.638 | -3.41 | 0.0006 | 0.0028 |
| ENSG00000146006 | LRRTM2 | -2.176 | 0.388 | -5.616 | 0 | 0 |
| ENSG00000250848 |  | -2.176 | 0.774 | -2.813 | 0.0049 | 0.0157 |
| ENSG00000242052 | RPL10P7 | -2.175 | 0.704 | -3.087 | 0.002 | 0.0074 |
| ENSG00000228701 | TNKS2-AS1 | -2.174 | 0.483 | -4.496 | 0 | 0.0001 |
| ENSG00000257256 |  | -2.174 | 0.65 | -3.347 | 0.0008 | 0.0034 |
| ENSG00000227214 |  | -2.171 | 0.472 | -4.597 | 0 | 0 |
| ENSG00000224959 |  | -2.171 | 0.474 | -4.578 | 0 | 0 |
| ENSG00000261526 |  | -2.171 | 0.312 | -6.956 | 0 | 0 |
| ENSG00000137273 | FOXF2 | -2.17 | 0.223 | -9.744 | 0 | 0 |
| ENSG00000237753 |  | -2.17 | 0.359 | -6.042 | 0 | 0 |
| ENSG00000175336 | APOF | -2.165 | 0.45 | -4.811 | 0 | 0 |
| ENSG00000261423 | TMEM202-AS1 | -2.165 | 0.307 | -7.048 | 0 | 0 |
| ENSG00000249129 |  | -2.164 | 0.895 | -2.419 | 0.0156 | 0.0406 |
| ENSG00000273965 |  | -2.163 | 0.79 | -2.739 | 0.0062 | 0.0189 |
| ENSG00000273270 |  | -2.162 | 0.279 | -7.735 | 0 | 0 |
| ENSG00000198680 | TUSC1 | -2.162 | 0.259 | -8.341 | 0 | 0 |
| ENSG00000228889 | UBAC2-AS1 | -2.161 | 0.373 | -5.793 | 0 | 0 |
| ENSG00000122787 | AKR1D1 | -2.161 | 0.856 | -2.525 | 0.0116 | 0.0318 |
| ENSG00000268686 | LOC101928295 | -2.158 | 0.496 | -4.347 | 0 | 0.0001 |
| ENSG00000258249 |  | -2.158 | 0.832 | -2.595 | 0.0095 | 0.0269 |
| ENSG00000253210 |  | -2.157 | 0.713 | -3.023 | 0.0025 | 0.0088 |
| ENSG00000233220 |  | -2.156 | 0.449 | -4.801 | 0 | 0 |
| ENSG00000258384 |  | -2.156 | 0.621 | -3.473 | 0.0005 | 0.0023 |
| ENSG00000152034 | MCHR2 | -2.153 | 0.68 | -3.165 | 0.0015 | 0.0059 |
| ENSG00000242531 |  | -2.148 | 0.871 | -2.465 | 0.0137 | 0.0365 |
| ENSG00000253385 |  | -2.147 | 0.839 | -2.558 | 0.0105 | 0.0294 |
| ENSG00000260219 | CD2BP2-DT | -2.145 | 0.434 | -4.945 | 0 | 0 |
| ENSG00000229512 |  | -2.145 | 0.525 | -4.084 | 0 | 0.0003 |
| ENSG00000227741 | LOC729867 | -2.144 | 0.303 | -7.071 | 0 | 0 |
| ENSG00000113719 | ERGIC1 | -2.141 | 0.281 | -7.62 | 0 | 0 |
| ENSG00000225783 | MIAT | -2.14 | 0.371 | -5.765 | 0 | 0 |
| ENSG00000247095 | MIR210HG | -2.14 | 0.545 | -3.929 | 0.0001 | 0.0005 |
| ENSG00000170099 | SERPINA6 | -2.14 | 0.627 | -3.411 | 0.0006 | 0.0028 |
| ENSG00000279641 |  | -2.137 | 0.363 | -5.883 | 0 | 0 |
| ENSG00000213782 | DDX47 | -2.137 | 0.482 | -4.435 | 0 | 0.0001 |
| ENSG00000254669 |  | -2.137 | 0.682 | -3.131 | 0.0017 | 0.0065 |
| ENSG00000143942 | CHAC2 | -2.134 | 0.336 | -6.351 | 0 | 0 |
| ENSG00000198858 | R3HDM4 | -2.133 | 0.34 | -6.269 | 0 | 0 |
| ENSG00000135722 | FBXL8 | -2.133 | 0.429 | -4.969 | 0 | 0 |
| ENSG00000083099 | LYRM2 | -2.133 | 0.256 | -8.342 | 0 | 0 |
| ENSG00000273289 |  | -2.133 | 0.757 | -2.817 | 0.0048 | 0.0155 |
| ENSG00000142405 | NLRP12 | -2.132 | 0.844 | -2.527 | 0.0115 | 0.0316 |
| ENSG00000026025 | VIM | -2.13 | 0.29 | -7.334 | 0 | 0 |
| ENSG00000112305 | SMAP1 | -2.13 | 0.354 | -6.009 | 0 | 0 |
| ENSG00000198795 | ZNF521 | -2.13 | 0.356 | -5.991 | 0 | 0 |
| ENSG00000181789 | COPG1 | -2.128 | 0.272 | -7.811 | 0 | 0 |
| ENSG00000139899 | CBLN3 | -2.127 | 0.415 | -5.123 | 0 | 0 |
| ENSG00000261226 |  | -2.126 | 0.791 | -2.688 | 0.0072 | 0.0216 |
| ENSG00000267375 |  | -2.125 | 0.885 | -2.402 | 0.0163 | 0.0422 |
| ENSG00000227268 | KLLN | -2.124 | 0.273 | -7.778 | 0 | 0 |
| ENSG00000167889 | MGAT5B | -2.124 | 0.639 | -3.324 | 0.0009 | 0.0037 |
| ENSG00000227630 | LINC01132 | -2.123 | 0.62 | -3.425 | 0.0006 | 0.0027 |
| ENSG00000213930 | GALT | -2.122 | 0.354 | -6 | 0 | 0 |
| ENSG00000228146 | CASP16P | -2.122 | 0.609 | -3.487 | 0.0005 | 0.0022 |
| ENSG00000260979 |  | -2.12 | 0.73 | -2.904 | 0.0037 | 0.0123 |
| ENSG00000267549 |  | -2.12 | 0.895 | -2.368 | 0.0179 | 0.0455 |
| ENSG00000235688 |  | -2.119 | 0.627 | -3.38 | 0.0007 | 0.0031 |
| ENSG00000259467 |  | -2.119 | 0.692 | -3.063 | 0.0022 | 0.0079 |
| ENSG00000175764 | TTLL11 | -2.116 | 0.376 | -5.63 | 0 | 0 |
| ENSG00000130518 | IQCN | -2.116 | 0.261 | -8.099 | 0 | 0 |
| ENSG00000188624 | IGFL3 | -2.115 | 0.632 | -3.347 | 0.0008 | 0.0034 |
| ENSG00000173110 | HSPA6 | -2.114 | 0.461 | -4.581 | 0 | 0 |
| ENSG00000223810 | KRT8P28 | -2.114 | 0.72 | -2.937 | 0.0033 | 0.0112 |
| ENSG00000085552 | IGSF9 | -2.113 | 0.454 | -4.654 | 0 | 0 |
| ENSG00000228606 | LOC100287049 | -2.113 | 0.756 | -2.794 | 0.0052 | 0.0165 |
| ENSG00000188163 | FAM166A | -2.111 | 0.347 | -6.091 | 0 | 0 |
| ENSG00000163633 | C4orf36 | -2.108 | 0.351 | -6.009 | 0 | 0 |
| ENSG00000162618 | ADGRL4 | -2.108 | 0.345 | -6.106 | 0 | 0 |
| ENSG00000217275 | RPS10P1 | -2.106 | 0.759 | -2.773 | 0.0056 | 0.0174 |
| ENSG00000175664 | TEX26 | -2.106 | 0.79 | -2.666 | 0.0077 | 0.0227 |
| ENSG00000265345 | MIR5188 | -2.105 | 0.59 | -3.57 | 0.0004 | 0.0017 |
| ENSG00000211584 | SLC48A1 | -2.103 | 0.32 | -6.58 | 0 | 0 |
| ENSG00000162694 | EXTL2 | -2.101 | 0.376 | -5.583 | 0 | 0 |
| ENSG00000163430 | FSTL1 | -2.098 | 0.196 | -10.725 | 0 | 0 |
| ENSG00000162231 | NXF1 | -2.097 | 0.251 | -8.362 | 0 | 0 |
| ENSG00000246174 | KCTD21-AS1 | -2.097 | 0.452 | -4.64 | 0 | 0 |
| ENSG00000185958 | FAM186A | -2.097 | 0.697 | -3.009 | 0.0026 | 0.0092 |
| ENSG00000062716 | VMP1 | -2.093 | 0.228 | -9.163 | 0 | 0 |
| ENSG00000261441 |  | -2.092 | 0.219 | -9.545 | 0 | 0 |
| ENSG00000232973 | CYP1B1-AS1 | -2.092 | 0.275 | -7.601 | 0 | 0 |
| ENSG00000005486 | RHBDD2 | -2.09 | 0.236 | -8.854 | 0 | 0 |
| ENSG00000007038 | PRSS21 | -2.089 | 0.706 | -2.958 | 0.0031 | 0.0106 |
| ENSG00000197506 | SLC28A3 | -2.088 | 0.391 | -5.345 | 0 | 0 |
| ENSG00000205269 | TMEM170B | -2.088 | 0.458 | -4.562 | 0 | 0 |
| ENSG00000272953 |  | -2.088 | 0.564 | -3.703 | 0.0002 | 0.0011 |
| ENSG00000176183 |  | -2.088 | 0.802 | -2.604 | 0.0092 | 0.0264 |
| ENSG00000213853 | EMP2 | -2.086 | 0.275 | -7.594 | 0 | 0 |
| ENSG00000260083 | MIR762HG | -2.085 | 0.619 | -3.368 | 0.0008 | 0.0032 |
| ENSG00000165238 | WNK2 | -2.085 | 0.648 | -3.216 | 0.0013 | 0.0051 |
| ENSG00000268996 | MAN1B1-DT | -2.084 | 0.25 | -8.324 | 0 | 0 |
| ENSG00000259374 | NDUFB4P11 | -2.084 | 0.854 | -2.442 | 0.0146 | 0.0386 |
| ENSG00000282961 | PRNCR1 | -2.083 | 0.815 | -2.556 | 0.0106 | 0.0296 |
| ENSG00000101605 | MYOM1 | -2.081 | 0.893 | -2.329 | 0.0199 | 0.0497 |
| ENSG00000168062 | BATF2 | -2.08 | 0.383 | -5.434 | 0 | 0 |
| ENSG00000100038 | TOP3B | -2.08 | 0.764 | -2.721 | 0.0065 | 0.0199 |
| ENSG00000240476 |  | -2.078 | 0.38 | -5.474 | 0 | 0 |
| ENSG00000177854 | TMEM187 | -2.078 | 0.362 | -5.746 | 0 | 0 |
| ENSG00000237330 | RNF223 | -2.078 | 0.508 | -4.089 | 0 | 0.0003 |
| ENSG00000254477 |  | -2.077 | 0.779 | -2.664 | 0.0077 | 0.0228 |
| ENSG00000258001 |  | -2.077 | 0.825 | -2.516 | 0.0119 | 0.0325 |
| ENSG00000204595 | DPRX | -2.076 | 0.848 | -2.446 | 0.0144 | 0.0382 |
| ENSG00000243224 | LOC101929054 | -2.075 | 0.692 | -2.997 | 0.0027 | 0.0095 |
| ENSG00000133114 | GPALPP1 | -2.073 | 0.321 | -6.464 | 0 | 0 |
| ENSG00000188021 | UBQLN2 | -2.072 | 0.237 | -8.729 | 0 | 0 |
| ENSG00000260156 |  | -2.072 | 0.518 | -3.999 | 0.0001 | 0.0004 |
| ENSG00000264175 | MIR3189 | -2.072 | 0.748 | -2.769 | 0.0056 | 0.0176 |
| ENSG00000273888 | FRMD6-AS1 | -2.071 | 0.319 | -6.493 | 0 | 0 |
| ENSG00000269506 |  | -2.071 | 0.502 | -4.127 | 0 | 0.0002 |
| ENSG00000124467 | PSG8 | -2.07 | 0.317 | -6.526 | 0 | 0 |
| ENSG00000258279 | LINC00592 | -2.069 | 0.76 | -2.724 | 0.0065 | 0.0197 |
| ENSG00000234378 |  | -2.068 | 0.565 | -3.657 | 0.0003 | 0.0013 |
| ENSG00000277142 |  | -2.067 | 0.339 | -6.09 | 0 | 0 |
| ENSG00000256463 | SALL3 | -2.067 | 0.773 | -2.674 | 0.0075 | 0.0223 |
| ENSG00000281937 |  | -2.066 | 0.4 | -5.162 | 0 | 0 |
| ENSG00000225945 |  | -2.066 | 0.535 | -3.86 | 0.0001 | 0.0006 |
| ENSG00000112186 | CAP2 | -2.065 | 0.26 | -7.947 | 0 | 0 |
| ENSG00000265798 |  | -2.065 | 0.559 | -3.692 | 0.0002 | 0.0011 |
| ENSG00000253276 | CCDC71L | -2.064 | 0.227 | -9.082 | 0 | 0 |
| ENSG00000178175 | ZNF366 | -2.063 | 0.628 | -3.285 | 0.001 | 0.0042 |
| ENSG00000265688 | MAFG-DT | -2.062 | 0.384 | -5.372 | 0 | 0 |
| ENSG00000234494 | SP2-AS1 | -2.062 | 0.48 | -4.292 | 0 | 0.0001 |
| ENSG00000100445 | SDR39U1 | -2.061 | 0.388 | -5.314 | 0 | 0 |
| ENSG00000245112 | SMARCA5-AS1 | -2.061 | 0.828 | -2.489 | 0.0128 | 0.0346 |
| ENSG00000236318 |  | -2.059 | 0.522 | -3.944 | 0.0001 | 0.0005 |
| ENSG00000282041 |  | -2.058 | 0.77 | -2.673 | 0.0075 | 0.0223 |
| ENSG00000005513 | SOX8 | -2.057 | 0.71 | -2.896 | 0.0038 | 0.0126 |
| ENSG00000260898 | ADPGK-AS1 | -2.056 | 0.395 | -5.205 | 0 | 0 |
| ENSG00000102312 | PORCN | -2.055 | 0.265 | -7.76 | 0 | 0 |
| ENSG00000231652 | LOC101928489 | -2.055 | 0.465 | -4.417 | 0 | 0.0001 |
| ENSG00000177602 | HASPIN | -2.052 | 0.304 | -6.756 | 0 | 0 |
| ENSG00000140718 | FTO | -2.051 | 0.274 | -7.495 | 0 | 0 |
| ENSG00000128016 | ZFP36 | -2.049 | 0.325 | -6.302 | 0 | 0 |
| ENSG00000162073 | PAQR4 | -2.049 | 0.371 | -5.519 | 0 | 0 |
| ENSG00000149485 | FADS1 | -2.049 | 0.3 | -6.836 | 0 | 0 |
| ENSG00000164683 | HEY1 | -2.048 | 0.483 | -4.24 | 0 | 0.0002 |
| ENSG00000170054 | SERPINA9 | -2.048 | 0.858 | -2.388 | 0.017 | 0.0436 |
| ENSG00000100304 | TTLL12 | -2.047 | 0.326 | -6.269 | 0 | 0 |
| ENSG00000077458 | FAM76B | -2.046 | 0.351 | -5.825 | 0 | 0 |
| ENSG00000235143 |  | -2.046 | 0.82 | -2.495 | 0.0126 | 0.0341 |
| ENSG00000158019 | BABAM2 | -2.043 | 0.284 | -7.202 | 0 | 0 |
| ENSG00000181541 | MAB21L2 | -2.043 | 0.352 | -5.802 | 0 | 0 |
| ENSG00000280195 |  | -2.042 | 0.439 | -4.655 | 0 | 0 |
| ENSG00000273749 | CYFIP1 | -2.042 | 0.251 | -8.146 | 0 | 0 |
| ENSG00000196371 | FUT4 | -2.042 | 0.385 | -5.312 | 0 | 0 |
| ENSG00000188032 | C19orf67 | -2.042 | 0.601 | -3.4 | 0.0007 | 0.0029 |
| ENSG00000168517 | HEXIM2 | -2.041 | 0.355 | -5.742 | 0 | 0 |
| ENSG00000213967 | ZNF726 | -2.04 | 0.286 | -7.138 | 0 | 0 |
| ENSG00000172269 | DPAGT1 | -2.039 | 0.286 | -7.124 | 0 | 0 |
| ENSG00000136826 | KLF4 | -2.039 | 0.238 | -8.557 | 0 | 0 |
| ENSG00000232519 |  | -2.038 | 0.436 | -4.673 | 0 | 0 |
| ENSG00000270068 |  | -2.037 | 0.594 | -3.431 | 0.0006 | 0.0026 |
| ENSG00000269855 | RNF225 | -2.036 | 0.41 | -4.969 | 0 | 0 |
| ENSG00000160224 | AIRE | -2.036 | 0.789 | -2.579 | 0.0099 | 0.028 |
| ENSG00000236603 |  | -2.035 | 0.65 | -3.132 | 0.0017 | 0.0065 |
| ENSG00000228172 |  | -2.035 | 0.709 | -2.871 | 0.0041 | 0.0134 |
| ENSG00000255647 | LOC731157 | -2.035 | 0.755 | -2.695 | 0.007 | 0.0212 |
| ENSG00000184990 | SIVA1 | -2.033 | 0.235 | -8.646 | 0 | 0 |
| ENSG00000235823 | OLMALINC | -2.033 | 0.589 | -3.451 | 0.0006 | 0.0025 |
| ENSG00000114902 | SPCS1 | -2.032 | 0.232 | -8.762 | 0 | 0 |
| ENSG00000268030 |  | -2.03 | 0.536 | -3.786 | 0.0002 | 0.0008 |
| ENSG00000154556 | SORBS2 | -2.03 | 0.809 | -2.51 | 0.0121 | 0.0329 |
| ENSG00000106049 | HIBADH | -2.029 | 0.308 | -6.595 | 0 | 0 |
| ENSG00000206527 | HACD2 | -2.029 | 0.251 | -8.09 | 0 | 0 |
| ENSG00000027847 | B4GALT7 | -2.028 | 0.287 | -7.059 | 0 | 0 |
| ENSG00000206344 | HCG27 | -2.028 | 0.531 | -3.819 | 0.0001 | 0.0007 |
| ENSG00000099864 | PALM | -2.026 | 0.636 | -3.184 | 0.0015 | 0.0056 |
| ENSG00000280303 |  | -2.025 | 0.418 | -4.838 | 0 | 0 |
| ENSG00000273008 |  | -2.024 | 0.392 | -5.16 | 0 | 0 |
| ENSG00000270177 |  | -2.024 | 0.334 | -6.059 | 0 | 0 |
| ENSG00000250919 |  | -2.024 | 0.811 | -2.496 | 0.0126 | 0.034 |
| ENSG00000264448 |  | -2.023 | 0.676 | -2.991 | 0.0028 | 0.0097 |
| ENSG00000207547 | MIR25 | -2.023 | 0.75 | -2.699 | 0.0069 | 0.0209 |
| ENSG00000237499 | WAKMAR2 | -2.022 | 0.278 | -7.264 | 0 | 0 |
| ENSG00000126351 | THRA | -2.021 | 0.248 | -8.164 | 0 | 0 |
| ENSG00000279080 |  | -2.021 | 0.421 | -4.797 | 0 | 0 |
| ENSG00000108515 | ENO3 | -2.02 | 0.349 | -5.789 | 0 | 0 |
| ENSG00000275491 | LINC01730 | -2.019 | 0.502 | -4.019 | 0.0001 | 0.0004 |
| ENSG00000261553 |  | -2.019 | 0.557 | -3.627 | 0.0003 | 0.0014 |
| ENSG00000186310 | NAP1L3 | -2.018 | 0.393 | -5.135 | 0 | 0 |
| ENSG00000272281 |  | -2.018 | 0.555 | -3.633 | 0.0003 | 0.0014 |
| ENSG00000273472 |  | -2.017 | 0.517 | -3.902 | 0.0001 | 0.0005 |
| ENSG00000279660 |  | -2.016 | 0.451 | -4.467 | 0 | 0.0001 |
| ENSG00000273619 |  | -2.015 | 0.455 | -4.432 | 0 | 0.0001 |
| ENSG00000164086 | DUSP7 | -2.014 | 0.232 | -8.695 | 0 | 0 |
| ENSG00000274565 |  | -2.012 | 0.312 | -6.448 | 0 | 0 |
| ENSG00000198846 | TOX | -2.011 | 0.389 | -5.173 | 0 | 0 |
| ENSG00000256128 |  | -2.009 | 0.576 | -3.49 | 0.0005 | 0.0022 |
| ENSG00000174348 | PODN | -2.008 | 0.397 | -5.059 | 0 | 0 |
| ENSG00000156467 | UQCRB | -2.006 | 0.278 | -7.212 | 0 | 0 |
| ENSG00000174473 | GALNTL6 | -2.005 | 0.53 | -3.784 | 0.0002 | 0.0008 |
| ENSG00000100036 | SLC35E4 | -2.004 | 0.241 | -8.32 | 0 | 0 |
| ENSG00000136573 | BLK | -2.003 | 0.665 | -3.01 | 0.0026 | 0.0092 |
| ENSG00000264589 |  | -2.003 | 0.81 | -2.473 | 0.0134 | 0.0359 |
| ENSG00000109771 | LRP2BP | -2.002 | 0.429 | -4.668 | 0 | 0 |
| ENSG00000227496 |  | -2.002 | 0.624 | -3.209 | 0.0013 | 0.0052 |
| ENSG00000231521 | LOC105376171 | -2.002 | 0.852 | -2.351 | 0.0187 | 0.0473 |
| ENSG00000066455 | GOLGA5 | -2.001 | 0.3 | -6.673 | 0 | 0 |
| ENSG00000182405 | PGBD4 | -2 | 0.251 | -7.962 | 0 | 0 |
| ENSG00000273456 |  | -1.999 | 0.371 | -5.387 | 0 | 0 |
| ENSG00000179546 | HTR1D | -1.999 | 0.474 | -4.215 | 0 | 0.0002 |
| ENSG00000236559 |  | -1.998 | 0.746 | -2.677 | 0.0074 | 0.0221 |
| ENSG00000169964 | TMEM42 | -1.996 | 0.283 | -7.058 | 0 | 0 |
| ENSG00000148677 | ANKRD1 | -1.996 | 0.265 | -7.547 | 0 | 0 |
| ENSG00000004059 | ARF5 | -1.995 | 0.237 | -8.426 | 0 | 0 |
| ENSG00000247934 |  | -1.994 | 0.403 | -4.953 | 0 | 0 |
| ENSG00000156853 | ZNF689 | -1.994 | 0.305 | -6.536 | 0 | 0 |
| ENSG00000185885 | IFITM1 | -1.993 | 0.272 | -7.318 | 0 | 0 |
| ENSG00000205885 | C1RL-AS1 | -1.993 | 0.472 | -4.223 | 0 | 0.0002 |
| ENSG00000213963 | LOC100130691 | -1.993 | 0.597 | -3.341 | 0.0008 | 0.0035 |
| ENSG00000262118 | MTCO1P28 | -1.991 | 0.766 | -2.599 | 0.0093 | 0.0267 |
| ENSG00000119899 | SLC17A5 | -1.988 | 0.291 | -6.822 | 0 | 0 |
| ENSG00000272870 | SAP30-DT | -1.988 | 0.667 | -2.981 | 0.0029 | 0.0099 |
| ENSG00000272086 |  | -1.987 | 0.391 | -5.076 | 0 | 0 |
| ENSG00000242797 |  | -1.986 | 0.7 | -2.839 | 0.0045 | 0.0146 |
| ENSG00000252827 | RN7SKP11 | -1.986 | 0.809 | -2.456 | 0.0141 | 0.0373 |
| ENSG00000226853 |  | -1.985 | 0.467 | -4.253 | 0 | 0.0001 |
| ENSG00000272812 |  | -1.985 | 0.473 | -4.196 | 0 | 0.0002 |
| ENSG00000269706 |  | -1.983 | 0.533 | -3.722 | 0.0002 | 0.001 |
| ENSG00000240350 |  | -1.983 | 0.764 | -2.594 | 0.0095 | 0.027 |
| ENSG00000134780 | DAGLA | -1.982 | 0.436 | -4.542 | 0 | 0 |
| ENSG00000269001 |  | -1.981 | 0.405 | -4.893 | 0 | 0 |
| ENSG00000163739 | CXCL1 | -1.98 | 0.44 | -4.505 | 0 | 0.0001 |
| ENSG00000272821 |  | -1.98 | 0.481 | -4.112 | 0 | 0.0003 |
| ENSG00000283078 |  | -1.979 | 0.386 | -5.128 | 0 | 0 |
| ENSG00000139644 | TMBIM6 | -1.979 | 0.217 | -9.132 | 0 | 0 |
| ENSG00000260018 |  | -1.979 | 0.338 | -5.862 | 0 | 0 |
| ENSG00000225206 | MIR137HG | -1.978 | 0.302 | -6.555 | 0 | 0 |
| ENSG00000213928 | IRF9 | -1.978 | 0.424 | -4.67 | 0 | 0 |
| ENSG00000164746 | C7orf57 | -1.977 | 0.747 | -2.647 | 0.0081 | 0.0238 |
| ENSG00000256746 | MADD-AS1 | -1.976 | 0.624 | -3.168 | 0.0015 | 0.0059 |
| ENSG00000227355 |  | -1.975 | 0.574 | -3.442 | 0.0006 | 0.0026 |
| ENSG00000228293 |  | -1.973 | 0.641 | -3.08 | 0.0021 | 0.0075 |
| ENSG00000259462 | CPEB1-AS1 | -1.971 | 0.753 | -2.616 | 0.0089 | 0.0256 |
| ENSG00000072818 | ACAP1 | -1.969 | 0.336 | -5.866 | 0 | 0 |
| ENSG00000131771 | PPP1R1B | -1.969 | 0.445 | -4.426 | 0 | 0.0001 |
| ENSG00000273226 |  | -1.969 | 0.517 | -3.811 | 0.0001 | 0.0007 |
| ENSG00000159263 | SIM2 | -1.969 | 0.816 | -2.412 | 0.0159 | 0.0413 |
| ENSG00000184207 | PGP | -1.968 | 0.253 | -7.781 | 0 | 0 |
| ENSG00000264575 |  | -1.968 | 0.358 | -5.505 | 0 | 0 |
| ENSG00000245888 | NSMCE1-DT | -1.968 | 0.589 | -3.341 | 0.0008 | 0.0035 |
| ENSG00000159167 | STC1 | -1.967 | 0.223 | -8.817 | 0 | 0 |
| ENSG00000178093 | TSSK6 | -1.964 | 0.458 | -4.292 | 0 | 0.0001 |
| ENSG00000009335 | UBE3C | -1.963 | 0.221 | -8.885 | 0 | 0 |
| ENSG00000276728 |  | -1.962 | 0.357 | -5.497 | 0 | 0 |
| ENSG00000204381 | LAYN | -1.962 | 0.312 | -6.285 | 0 | 0 |
| ENSG00000229043 | ZFAND2A-DT | -1.961 | 0.361 | -5.428 | 0 | 0 |
| ENSG00000267737 |  | -1.96 | 0.83 | -2.363 | 0.0182 | 0.0461 |
| ENSG00000221949 | LINC01465 | -1.957 | 0.276 | -7.083 | 0 | 0 |
| ENSG00000179029 | TMEM107 | -1.956 | 0.327 | -5.985 | 0 | 0 |
| ENSG00000139679 | LPAR6 | -1.955 | 0.298 | -6.57 | 0 | 0 |
| ENSG00000228955 |  | -1.955 | 0.432 | -4.525 | 0 | 0 |
| ENSG00000106077 | ABHD11 | -1.954 | 0.281 | -6.961 | 0 | 0 |
| ENSG00000260830 |  | -1.954 | 0.641 | -3.046 | 0.0023 | 0.0083 |
| ENSG00000264187 |  | -1.953 | 0.782 | -2.496 | 0.0125 | 0.034 |
| ENSG00000269652 |  | -1.952 | 0.549 | -3.554 | 0.0004 | 0.0018 |
| ENSG00000205593 | DENND6B | -1.95 | 0.315 | -6.184 | 0 | 0 |
| ENSG00000204569 | PPP1R10 | -1.95 | 0.208 | -9.376 | 0 | 0 |
| ENSG00000280247 |  | -1.95 | 0.758 | -2.57 | 0.0102 | 0.0286 |
| ENSG00000075618 | FSCN1 | -1.949 | 0.219 | -8.884 | 0 | 0 |
| ENSG00000226891 | LINC01359 | -1.949 | 0.629 | -3.099 | 0.0019 | 0.0071 |
| ENSG00000183161 | FANCF | -1.948 | 0.302 | -6.457 | 0 | 0 |
| ENSG00000118523 | CCN2 | -1.948 | 0.204 | -9.555 | 0 | 0 |
| ENSG00000231889 | TRAF3IP2-AS1 | -1.947 | 0.296 | -6.572 | 0 | 0 |
| ENSG00000146476 | ARMT1 | -1.947 | 0.306 | -6.371 | 0 | 0 |
| ENSG00000100253 | MIOX | -1.942 | 0.633 | -3.068 | 0.0022 | 0.0078 |
| ENSG00000151611 | MMAA | -1.941 | 0.302 | -6.435 | 0 | 0 |
| ENSG00000124523 | SIRT5 | -1.94 | 0.308 | -6.297 | 0 | 0 |
| ENSG00000154451 | GBP5 | -1.94 | 0.66 | -2.941 | 0.0033 | 0.0111 |
| ENSG00000235180 | LINC00601 | -1.94 | 0.667 | -2.91 | 0.0036 | 0.0121 |
| ENSG00000152292 | SH2D6 | -1.938 | 0.568 | -3.411 | 0.0006 | 0.0028 |
| ENSG00000181104 | F2R | -1.937 | 0.286 | -6.783 | 0 | 0 |
| ENSG00000174885 | NLRP6 | -1.935 | 0.776 | -2.492 | 0.0127 | 0.0343 |
| ENSG00000116685 | KIAA2013 | -1.934 | 0.219 | -8.843 | 0 | 0 |
| ENSG00000149798 | CDC42EP2 | -1.934 | 0.293 | -6.595 | 0 | 0 |
| ENSG00000253490 | LINC02099 | -1.933 | 0.397 | -4.874 | 0 | 0 |
| ENSG00000274220 |  | -1.933 | 0.476 | -4.065 | 0 | 0.0003 |
| ENSG00000102931 | ARL2BP | -1.933 | 0.589 | -3.283 | 0.001 | 0.0042 |
| ENSG00000168876 | ANKRD49 | -1.932 | 0.336 | -5.746 | 0 | 0 |
| ENSG00000179476 | C14orf28 | -1.931 | 0.342 | -5.651 | 0 | 0 |
| ENSG00000224149 |  | -1.931 | 0.49 | -3.94 | 0.0001 | 0.0005 |
| ENSG00000201558 | RNVU1-6 | -1.931 | 0.729 | -2.649 | 0.0081 | 0.0237 |
| ENSG00000188869 | TMC3 | -1.931 | 0.76 | -2.542 | 0.011 | 0.0306 |
| ENSG00000266554 | LINC01443 | -1.93 | 0.301 | -6.404 | 0 | 0 |
| ENSG00000279072 |  | -1.93 | 0.704 | -2.741 | 0.0061 | 0.0189 |
| ENSG00000212916 | MAP10 | -1.928 | 0.7 | -2.753 | 0.0059 | 0.0183 |
| ENSG00000263826 |  | -1.927 | 0.293 | -6.57 | 0 | 0 |
| ENSG00000226193 |  | -1.926 | 0.724 | -2.658 | 0.0079 | 0.0232 |
| ENSG00000251015 | SLC25A30-AS1 | -1.926 | 0.741 | -2.598 | 0.0094 | 0.0267 |
| ENSG00000131094 | C1QL1 | -1.926 | 0.756 | -2.548 | 0.0108 | 0.0302 |
| ENSG00000182196 | ARL6IP4 | -1.924 | 0.588 | -3.271 | 0.0011 | 0.0043 |
| ENSG00000259146 |  | -1.924 | 0.622 | -3.092 | 0.002 | 0.0073 |
| ENSG00000106366 | SERPINE1 | -1.922 | 0.281 | -6.834 | 0 | 0 |
| ENSG00000241370 | RPP21 | -1.922 | 0.784 | -2.452 | 0.0142 | 0.0377 |
| ENSG00000128311 | TST | -1.921 | 0.369 | -5.199 | 0 | 0 |
| ENSG00000176340 | COX8A | -1.921 | 0.204 | -9.435 | 0 | 0 |
| ENSG00000135116 | HRK | -1.921 | 0.444 | -4.329 | 0 | 0.0001 |
| ENSG00000248932 | LOC100507291 | -1.921 | 0.458 | -4.198 | 0 | 0.0002 |
| ENSG00000142871 | CCN1 | -1.92 | 0.241 | -7.958 | 0 | 0 |
| ENSG00000150054 | MPP7 | -1.92 | 0.609 | -3.154 | 0.0016 | 0.0061 |
| ENSG00000254739 |  | -1.92 | 0.643 | -2.987 | 0.0028 | 0.0098 |
| ENSG00000081041 | CXCL2 | -1.919 | 0.411 | -4.668 | 0 | 0 |
| ENSG00000248323 | LUCAT1 | -1.919 | 0.44 | -4.36 | 0 | 0.0001 |
| ENSG00000029153 | ARNTL2 | -1.918 | 0.234 | -8.195 | 0 | 0 |
| ENSG00000164053 | ATRIP | -1.918 | 0.394 | -4.869 | 0 | 0 |
| ENSG00000114315 | HES1 | -1.915 | 0.355 | -5.389 | 0 | 0 |
| ENSG00000241288 | LINC02614 | -1.915 | 0.674 | -2.842 | 0.0045 | 0.0145 |
| ENSG00000186897 | C1QL4 | -1.915 | 0.786 | -2.436 | 0.0149 | 0.0391 |
| ENSG00000186806 | VSIG10L | -1.914 | 0.381 | -5.026 | 0 | 0 |
| ENSG00000106003 | LFNG | -1.913 | 0.434 | -4.403 | 0 | 0.0001 |
| ENSG00000270426 |  | -1.913 | 0.489 | -3.908 | 0.0001 | 0.0005 |
| ENSG00000183535 | COL18A1-AS1 | -1.913 | 0.795 | -2.408 | 0.016 | 0.0416 |
| ENSG00000280062 |  | -1.913 | 0.805 | -2.376 | 0.0175 | 0.0448 |
| ENSG00000102003 | SYP | -1.912 | 0.774 | -2.471 | 0.0135 | 0.036 |
| ENSG00000142192 | APP | -1.911 | 0.207 | -9.253 | 0 | 0 |
| ENSG00000114784 | EIF1B | -1.909 | 0.256 | -7.456 | 0 | 0 |
| ENSG00000236778 | INTS6-AS1 | -1.909 | 0.453 | -4.215 | 0 | 0.0002 |
| ENSG00000136371 | MTHFS | -1.909 | 0.774 | -2.466 | 0.0137 | 0.0364 |
| ENSG00000238184 | CD81-AS1 | -1.909 | 0.813 | -2.347 | 0.0189 | 0.0477 |
| ENSG00000188542 | DUSP28 | -1.907 | 0.268 | -7.116 | 0 | 0 |
| ENSG00000112902 | SEMA5A | -1.907 | 0.229 | -8.317 | 0 | 0 |
| ENSG00000179922 | ZNF784 | -1.907 | 0.653 | -2.918 | 0.0035 | 0.0118 |
| ENSG00000215912 | TTC34 | -1.907 | 0.708 | -2.693 | 0.0071 | 0.0213 |
| ENSG00000250536 |  | -1.905 | 0.783 | -2.432 | 0.015 | 0.0394 |
| ENSG00000221826 | PSG3 | -1.903 | 0.357 | -5.322 | 0 | 0 |
| ENSG00000153904 | DDAH1 | -1.903 | 0.267 | -7.133 | 0 | 0 |
| ENSG00000185515 | BRCC3 | -1.903 | 0.35 | -5.442 | 0 | 0 |
| ENSG00000249087 | ZNF436-AS1 | -1.901 | 0.361 | -5.267 | 0 | 0 |
| ENSG00000164061 | BSN | -1.9 | 0.491 | -3.872 | 0.0001 | 0.0006 |
| ENSG00000239219 | LOC100128164 | -1.9 | 0.552 | -3.44 | 0.0006 | 0.0026 |
| ENSG00000099956 | SMARCB1 | -1.895 | 0.273 | -6.947 | 0 | 0 |
| ENSG00000235885 | LOC101927661 | -1.895 | 0.391 | -4.842 | 0 | 0 |
| ENSG00000138617 | PARP16 | -1.895 | 0.36 | -5.266 | 0 | 0 |
| ENSG00000230658 |  | -1.895 | 0.401 | -4.721 | 0 | 0 |
| ENSG00000172345 | STARD5 | -1.895 | 0.526 | -3.606 | 0.0003 | 0.0015 |
| ENSG00000125735 | TNFSF14 | -1.894 | 0.673 | -2.813 | 0.0049 | 0.0157 |
| ENSG00000008323 | PLEKHG6 | -1.892 | 0.531 | -3.566 | 0.0004 | 0.0017 |
| ENSG00000249096 |  | -1.891 | 0.558 | -3.387 | 0.0007 | 0.003 |
| ENSG00000259162 |  | -1.891 | 0.623 | -3.037 | 0.0024 | 0.0085 |
| ENSG00000125657 | TNFSF9 | -1.889 | 0.325 | -5.806 | 0 | 0 |
| ENSG00000188177 | ZC3H6 | -1.889 | 0.299 | -6.317 | 0 | 0 |
| ENSG00000227775 |  | -1.889 | 0.486 | -3.887 | 0.0001 | 0.0006 |
| ENSG00000089163 | SIRT4 | -1.889 | 0.587 | -3.217 | 0.0013 | 0.0051 |
| ENSG00000278876 |  | -1.887 | 0.699 | -2.7 | 0.0069 | 0.0209 |
| ENSG00000263280 |  | -1.887 | 0.745 | -2.534 | 0.0113 | 0.0312 |
| ENSG00000242265 | PEG10 | -1.886 | 0.257 | -7.331 | 0 | 0 |
| ENSG00000273175 |  | -1.886 | 0.781 | -2.414 | 0.0158 | 0.0411 |
| ENSG00000198771 | RCSD1 | -1.884 | 0.661 | -2.852 | 0.0043 | 0.0141 |
| ENSG00000257596 | SCAT2 | -1.882 | 0.564 | -3.337 | 0.0008 | 0.0035 |
| ENSG00000275329 |  | -1.882 | 0.655 | -2.872 | 0.0041 | 0.0134 |
| ENSG00000101189 | MRGBP | -1.88 | 0.257 | -7.312 | 0 | 0 |
| ENSG00000125843 | AP5S1 | -1.877 | 0.302 | -6.21 | 0 | 0 |
| ENSG00000113391 | FAM172A | -1.875 | 0.331 | -5.656 | 0 | 0 |
| ENSG00000272686 |  | -1.874 | 0.25 | -7.481 | 0 | 0 |
| ENSG00000153044 | CENPH | -1.873 | 0.324 | -5.787 | 0 | 0 |
| ENSG00000159596 | TMEM69 | -1.872 | 0.25 | -7.496 | 0 | 0 |
| ENSG00000100243 | CYB5R3 | -1.872 | 0.24 | -7.811 | 0 | 0 |
| ENSG00000167034 | NKX3-1 | -1.872 | 0.297 | -6.312 | 0 | 0 |
| ENSG00000175449 | RFESD | -1.872 | 0.461 | -4.062 | 0 | 0.0003 |
| ENSG00000269959 | SPACA6P-AS | -1.872 | 0.67 | -2.794 | 0.0052 | 0.0165 |
| ENSG00000220256 |  | -1.871 | 0.754 | -2.481 | 0.0131 | 0.0352 |
| ENSG00000235314 |  | -1.871 | 0.775 | -2.413 | 0.0158 | 0.0411 |
| ENSG00000251191 | LINC00589 | -1.87 | 0.616 | -3.033 | 0.0024 | 0.0086 |
| ENSG00000097021 | ACOT7 | -1.869 | 0.265 | -7.054 | 0 | 0 |
| ENSG00000263412 |  | -1.866 | 0.318 | -5.866 | 0 | 0 |
| ENSG00000185127 | C6orf120 | -1.865 | 0.216 | -8.626 | 0 | 0 |
| ENSG00000237886 |  | -1.865 | 0.603 | -3.095 | 0.002 | 0.0072 |
| ENSG00000147144 | CCDC120 | -1.865 | 0.629 | -2.966 | 0.003 | 0.0104 |
| ENSG00000174327 | SLC16A13 | -1.865 | 0.704 | -2.65 | 0.008 | 0.0236 |
| ENSG00000266053 | NDUFV2-AS1 | -1.864 | 0.427 | -4.365 | 0 | 0.0001 |
| ENSG00000156384 | SFR1 | -1.862 | 0.268 | -6.955 | 0 | 0 |
| ENSG00000214900 | LINC01588 | -1.861 | 0.265 | -7.024 | 0 | 0 |
| ENSG00000237338 | FTCD-AS1 | -1.861 | 0.523 | -3.561 | 0.0004 | 0.0017 |
| ENSG00000205424 |  | -1.861 | 0.561 | -3.315 | 0.0009 | 0.0038 |
| ENSG00000240065 | PSMB9 | -1.86 | 0.443 | -4.198 | 0 | 0.0002 |
| ENSG00000268100 |  | -1.86 | 0.66 | -2.817 | 0.0048 | 0.0155 |
| ENSG00000280594 | BTG3-AS1 | -1.859 | 0.353 | -5.274 | 0 | 0 |
| ENSG00000254530 |  | -1.859 | 0.739 | -2.516 | 0.0119 | 0.0325 |
| ENSG00000169220 | RGS14 | -1.858 | 0.564 | -3.294 | 0.001 | 0.004 |
| ENSG00000169715 | MT1E | -1.856 | 0.259 | -7.169 | 0 | 0 |
| ENSG00000247624 | CPEB2-DT | -1.856 | 0.439 | -4.228 | 0 | 0.0002 |
| ENSG00000258399 |  | -1.854 | 0.466 | -3.978 | 0.0001 | 0.0004 |
| ENSG00000130303 | BST2 | -1.854 | 0.764 | -2.427 | 0.0152 | 0.0399 |
| ENSG00000125434 | SLC25A35 | -1.853 | 0.429 | -4.318 | 0 | 0.0001 |
| ENSG00000269416 | LINC01224 | -1.853 | 0.775 | -2.392 | 0.0167 | 0.0431 |
| ENSG00000265737 |  | -1.85 | 0.635 | -2.912 | 0.0036 | 0.012 |
| ENSG00000134602 | STK26 | -1.849 | 0.587 | -3.149 | 0.0016 | 0.0062 |
| ENSG00000099251 | HSD17B7P2 | -1.849 | 0.648 | -2.854 | 0.0043 | 0.0141 |
| ENSG00000180694 | TMEM64 | -1.848 | 0.256 | -7.231 | 0 | 0 |
| ENSG00000279339 |  | -1.844 | 0.501 | -3.678 | 0.0002 | 0.0012 |
| ENSG00000180209 | MYLPF | -1.844 | 0.664 | -2.776 | 0.0055 | 0.0173 |
| ENSG00000274029 |  | -1.844 | 0.676 | -2.73 | 0.0063 | 0.0194 |
| ENSG00000102984 | ZNF821 | -1.842 | 0.44 | -4.191 | 0 | 0.0002 |
| ENSG00000129535 | NRL | -1.841 | 0.745 | -2.471 | 0.0135 | 0.036 |
| ENSG00000111231 | GPN3 | -1.839 | 0.358 | -5.131 | 0 | 0 |
| ENSG00000179965 | ZNF771 | -1.839 | 0.341 | -5.399 | 0 | 0 |
| ENSG00000160746 | ANO10 | -1.839 | 0.244 | -7.53 | 0 | 0 |
| ENSG00000108342 | CSF3 | -1.838 | 0.434 | -4.23 | 0 | 0.0002 |
| ENSG00000100522 | GNPNAT1 | -1.837 | 0.251 | -7.305 | 0 | 0 |
| ENSG00000108064 | TFAM | -1.837 | 0.247 | -7.448 | 0 | 0 |
| ENSG00000101310 | SEC23B | -1.837 | 0.263 | -6.972 | 0 | 0 |
| ENSG00000181649 | PHLDA2 | -1.835 | 0.253 | -7.263 | 0 | 0 |
| ENSG00000276255 | LINC02809 | -1.835 | 0.757 | -2.424 | 0.0153 | 0.0401 |
| ENSG00000178719 | GRINA | -1.832 | 0.231 | -7.935 | 0 | 0 |
| ENSG00000221916 | C19orf73 | -1.832 | 0.513 | -3.569 | 0.0004 | 0.0017 |
| ENSG00000144229 | THSD7B | -1.831 | 0.755 | -2.425 | 0.0153 | 0.0401 |
| ENSG00000213420 | GPC2 | -1.83 | 0.549 | -3.336 | 0.0009 | 0.0036 |
| ENSG00000228412 | LNC-LBCS | -1.83 | 0.567 | -3.226 | 0.0013 | 0.005 |
| ENSG00000272240 |  | -1.83 | 0.572 | -3.198 | 0.0014 | 0.0054 |
| ENSG00000136802 | LRRC8A | -1.829 | 0.292 | -6.271 | 0 | 0 |
| ENSG00000166359 | WDR88 | -1.829 | 0.535 | -3.42 | 0.0006 | 0.0027 |
| ENSG00000204060 | FOXO6 | -1.829 | 0.675 | -2.71 | 0.0067 | 0.0204 |
| ENSG00000243926 |  | -1.828 | 0.315 | -5.807 | 0 | 0 |
| ENSG00000227438 |  | -1.827 | 0.59 | -3.095 | 0.002 | 0.0072 |
| ENSG00000253878 |  | -1.825 | 0.319 | -5.718 | 0 | 0 |
| ENSG00000260714 |  | -1.824 | 0.537 | -3.396 | 0.0007 | 0.003 |
| ENSG00000260366 |  | -1.824 | 0.687 | -2.655 | 0.0079 | 0.0234 |
| ENSG00000249502 |  | -1.823 | 0.401 | -4.549 | 0 | 0 |
| ENSG00000218537 |  | -1.821 | 0.498 | -3.657 | 0.0003 | 0.0013 |
| ENSG00000232442 | MHENCR | -1.819 | 0.309 | -5.888 | 0 | 0 |
| ENSG00000198053 | SIRPA | -1.818 | 0.343 | -5.298 | 0 | 0 |
| ENSG00000233276 | GPX1 | -1.818 | 0.248 | -7.321 | 0 | 0 |
| ENSG00000215790 | SLC35E2A | -1.817 | 0.488 | -3.723 | 0.0002 | 0.001 |
| ENSG00000241211 |  | -1.816 | 0.438 | -4.15 | 0 | 0.0002 |
| ENSG00000119535 | CSF3R | -1.816 | 0.563 | -3.225 | 0.0013 | 0.005 |
| ENSG00000275481 |  | -1.815 | 0.699 | -2.594 | 0.0095 | 0.027 |
| ENSG00000196110 | ZNF699 | -1.813 | 0.244 | -7.441 | 0 | 0 |
| ENSG00000276791 |  | -1.813 | 0.276 | -6.578 | 0 | 0 |
| ENSG00000267685 |  | -1.812 | 0.688 | -2.632 | 0.0085 | 0.0246 |
| ENSG00000136999 | CCN3 | -1.811 | 0.216 | -8.381 | 0 | 0 |
| ENSG00000263727 |  | -1.809 | 0.705 | -2.568 | 0.0102 | 0.0287 |
| ENSG00000270750 |  | -1.806 | 0.631 | -2.862 | 0.0042 | 0.0138 |
| ENSG00000196420 | S100A5 | -1.806 | 0.671 | -2.692 | 0.0071 | 0.0214 |
| ENSG00000159352 | PSMD4 | -1.805 | 0.253 | -7.135 | 0 | 0 |
| ENSG00000130589 | HELZ2 | -1.804 | 0.234 | -7.719 | 0 | 0 |
| ENSG00000205559 | CHKB-DT | -1.804 | 0.5 | -3.608 | 0.0003 | 0.0015 |
| ENSG00000172935 | MRGPRF | -1.802 | 0.256 | -7.051 | 0 | 0 |
| ENSG00000105245 | NUMBL | -1.802 | 0.363 | -4.967 | 0 | 0 |
| ENSG00000127870 | RNF6 | -1.801 | 0.218 | -8.251 | 0 | 0 |
| ENSG00000267321 |  | -1.8 | 0.283 | -6.367 | 0 | 0 |
| ENSG00000206885 | SNORA75 | -1.8 | 0.703 | -2.561 | 0.0104 | 0.0292 |
| ENSG00000224152 |  | -1.8 | 0.712 | -2.527 | 0.0115 | 0.0316 |
| ENSG00000186352 | ANKRD37 | -1.799 | 0.346 | -5.201 | 0 | 0 |
| ENSG00000234577 |  | -1.799 | 0.528 | -3.408 | 0.0007 | 0.0028 |
| ENSG00000247735 |  | -1.799 | 0.556 | -3.237 | 0.0012 | 0.0048 |
| ENSG00000227946 |  | -1.798 | 0.49 | -3.668 | 0.0002 | 0.0012 |
| ENSG00000078401 | EDN1 | -1.797 | 0.232 | -7.754 | 0 | 0 |
| ENSG00000084463 | WBP11 | -1.796 | 0.27 | -6.661 | 0 | 0 |
| ENSG00000261189 |  | -1.796 | 0.309 | -5.815 | 0 | 0 |
| ENSG00000178764 | ZHX2 | -1.796 | 0.463 | -3.883 | 0.0001 | 0.0006 |
| ENSG00000147140 | NONO | -1.795 | 0.206 | -8.726 | 0 | 0 |
| ENSG00000241313 | WWTR1-AS1 | -1.792 | 0.651 | -2.751 | 0.0059 | 0.0184 |
| ENSG00000130943 | PKDREJ | -1.79 | 0.53 | -3.377 | 0.0007 | 0.0031 |
| ENSG00000205208 | C4orf46 | -1.789 | 0.232 | -7.708 | 0 | 0 |
| ENSG00000224743 | TEX26-AS1 | -1.789 | 0.462 | -3.874 | 0.0001 | 0.0006 |
| ENSG00000124593 |  | -1.789 | 0.618 | -2.894 | 0.0038 | 0.0126 |
| ENSG00000276248 |  | -1.789 | 0.656 | -2.725 | 0.0064 | 0.0196 |
| ENSG00000234917 |  | -1.788 | 0.481 | -3.714 | 0.0002 | 0.001 |
| ENSG00000047457 | CP | -1.787 | 0.379 | -4.711 | 0 | 0 |
| ENSG00000198569 | SLC34A3 | -1.787 | 0.55 | -3.247 | 0.0012 | 0.0047 |
| ENSG00000188739 | RBM34 | -1.787 | 0.654 | -2.732 | 0.0063 | 0.0193 |
| ENSG00000244509 | APOBEC3C | -1.786 | 0.252 | -7.086 | 0 | 0 |
| ENSG00000143878 | RHOB | -1.786 | 0.209 | -8.552 | 0 | 0 |
| ENSG00000164440 | TXLNB | -1.785 | 0.427 | -4.184 | 0 | 0.0002 |
| ENSG00000261242 |  | -1.785 | 0.553 | -3.225 | 0.0013 | 0.005 |
| ENSG00000107518 | ATRNL1 | -1.783 | 0.328 | -5.441 | 0 | 0 |
| ENSG00000270194 | LOC152048 | -1.782 | 0.236 | -7.546 | 0 | 0 |
| ENSG00000176896 | TCEANC | -1.782 | 0.403 | -4.419 | 0 | 0.0001 |
| ENSG00000087494 | PTHLH | -1.781 | 0.385 | -4.62 | 0 | 0 |
| ENSG00000282386 |  | -1.781 | 0.567 | -3.143 | 0.0017 | 0.0063 |
| ENSG00000200534 | SNORA33 | -1.781 | 0.569 | -3.129 | 0.0018 | 0.0065 |
| ENSG00000269570 |  | -1.78 | 0.698 | -2.551 | 0.0107 | 0.0299 |
| ENSG00000186564 | FOXD2 | -1.779 | 0.558 | -3.186 | 0.0014 | 0.0056 |
| ENSG00000250007 | LOC101928174 | -1.779 | 0.582 | -3.056 | 0.0022 | 0.008 |
| ENSG00000134440 | NARS1 | -1.778 | 0.214 | -8.307 | 0 | 0 |
| ENSG00000260273 |  | -1.778 | 0.436 | -4.076 | 0 | 0.0003 |
| ENSG00000257913 | DDN-AS1 | -1.776 | 0.455 | -3.903 | 0.0001 | 0.0005 |
| ENSG00000187229 |  | -1.776 | 0.68 | -2.613 | 0.009 | 0.0258 |
| ENSG00000246863 |  | -1.775 | 0.326 | -5.442 | 0 | 0 |
| ENSG00000166341 | DCHS1 | -1.774 | 0.317 | -5.599 | 0 | 0 |
| ENSG00000167645 | YIF1B | -1.773 | 0.235 | -7.532 | 0 | 0 |
| ENSG00000223776 | LGALS8-AS1 | -1.773 | 0.596 | -2.973 | 0.003 | 0.0102 |
| ENSG00000275966 |  | -1.773 | 0.714 | -2.483 | 0.013 | 0.035 |
| ENSG00000272525 |  | -1.772 | 0.39 | -4.546 | 0 | 0 |
| ENSG00000019549 | SNAI2 | -1.771 | 0.234 | -7.575 | 0 | 0 |
| ENSG00000163661 | PTX3 | -1.771 | 0.338 | -5.234 | 0 | 0 |
| ENSG00000269737 |  | -1.771 | 0.499 | -3.546 | 0.0004 | 0.0018 |
| ENSG00000166428 | PLD4 | -1.771 | 0.568 | -3.118 | 0.0018 | 0.0068 |
| ENSG00000278899 | LOC645967 | -1.77 | 0.528 | -3.35 | 0.0008 | 0.0034 |
| ENSG00000275709 |  | -1.77 | 0.671 | -2.637 | 0.0084 | 0.0243 |
| ENSG00000267394 |  | -1.77 | 0.74 | -2.393 | 0.0167 | 0.0431 |
| ENSG00000232048 | HNRNPCP9 | -1.77 | 0.757 | -2.336 | 0.0195 | 0.0489 |
| ENSG00000144152 | FBLN7 | -1.769 | 0.347 | -5.093 | 0 | 0 |
| ENSG00000165152 | PGAP4 | -1.769 | 0.315 | -5.625 | 0 | 0 |
| ENSG00000258274 | LOC101928731 | -1.769 | 0.699 | -2.53 | 0.0114 | 0.0314 |
| ENSG00000269399 |  | -1.768 | 0.388 | -4.559 | 0 | 0 |
| ENSG00000116741 | RGS2 | -1.767 | 0.267 | -6.627 | 0 | 0 |
| ENSG00000244968 | LIFR-AS1 | -1.767 | 0.537 | -3.292 | 0.001 | 0.0041 |
| ENSG00000246777 | LOC106699570 | -1.766 | 0.642 | -2.753 | 0.0059 | 0.0183 |
| ENSG00000204371 | EHMT2 | -1.765 | 0.241 | -7.308 | 0 | 0 |
| ENSG00000245146 |  | -1.765 | 0.385 | -4.589 | 0 | 0 |
| ENSG00000275894 |  | -1.765 | 0.392 | -4.498 | 0 | 0.0001 |
| ENSG00000197961 | ZNF121 | -1.763 | 0.206 | -8.554 | 0 | 0 |
| ENSG00000180592 | SKIDA1 | -1.763 | 0.706 | -2.499 | 0.0125 | 0.0338 |
| ENSG00000262454 |  | -1.762 | 0.503 | -3.499 | 0.0005 | 0.0021 |
| ENSG00000114631 | PODXL2 | -1.761 | 0.36 | -4.896 | 0 | 0 |
| ENSG00000171148 | TADA3 | -1.761 | 0.241 | -7.298 | 0 | 0 |
| ENSG00000188488 | SERPINA5 | -1.761 | 0.68 | -2.588 | 0.0096 | 0.0274 |
| ENSG00000111450 | STX2 | -1.76 | 0.31 | -5.68 | 0 | 0 |
| ENSG00000155876 | RRAGA | -1.759 | 0.224 | -7.866 | 0 | 0 |
| ENSG00000115423 | DNAH6 | -1.757 | 0.657 | -2.674 | 0.0075 | 0.0223 |
| ENSG00000184697 | CLDN6 | -1.755 | 0.496 | -3.538 | 0.0004 | 0.0019 |
| ENSG00000272141 |  | -1.755 | 0.559 | -3.139 | 0.0017 | 0.0064 |
| ENSG00000264920 | LOC102724532 | -1.754 | 0.448 | -3.91 | 0.0001 | 0.0005 |
| ENSG00000179111 | HES7 | -1.754 | 0.656 | -2.675 | 0.0075 | 0.0223 |
| ENSG00000230715 |  | -1.754 | 0.688 | -2.548 | 0.0108 | 0.0302 |
| ENSG00000227354 | RBM26-AS1 | -1.753 | 0.378 | -4.633 | 0 | 0 |
| ENSG00000059804 | SLC2A3 | -1.753 | 0.232 | -7.563 | 0 | 0 |
| ENSG00000169918 | OTUD7A | -1.752 | 0.659 | -2.657 | 0.0079 | 0.0233 |
| ENSG00000275494 |  | -1.752 | 0.67 | -2.614 | 0.009 | 0.0257 |
| ENSG00000101204 | CHRNA4 | -1.751 | 0.648 | -2.701 | 0.0069 | 0.0209 |
| ENSG00000178878 | APOLD1 | -1.749 | 0.259 | -6.749 | 0 | 0 |
| ENSG00000100399 | CHADL | -1.749 | 0.417 | -4.195 | 0 | 0.0002 |
| ENSG00000107669 | ATE1 | -1.747 | 0.314 | -5.555 | 0 | 0 |
| ENSG00000249673 |  | -1.747 | 0.273 | -6.395 | 0 | 0 |
| ENSG00000235560 | LOC107984875 | -1.747 | 0.69 | -2.533 | 0.0113 | 0.0312 |
| ENSG00000160298 | C21orf58 | -1.746 | 0.299 | -5.848 | 0 | 0 |
| ENSG00000173852 | DPY19L1 | -1.745 | 0.318 | -5.485 | 0 | 0 |
| ENSG00000159200 | RCAN1 | -1.744 | 0.297 | -5.871 | 0 | 0 |
| ENSG00000248968 |  | -1.744 | 0.697 | -2.504 | 0.0123 | 0.0334 |
| ENSG00000226200 |  | -1.743 | 0.37 | -4.705 | 0 | 0 |
| ENSG00000272667 |  | -1.743 | 0.405 | -4.306 | 0 | 0.0001 |
| ENSG00000185187 | SIGIRR | -1.741 | 0.409 | -4.262 | 0 | 0.0001 |
| ENSG00000196337 | CGB7 | -1.737 | 0.516 | -3.367 | 0.0008 | 0.0032 |
| ENSG00000101294 | HM13 | -1.736 | 0.265 | -6.549 | 0 | 0 |
| ENSG00000279227 |  | -1.736 | 0.292 | -5.944 | 0 | 0 |
| ENSG00000112041 | TULP1 | -1.736 | 0.529 | -3.281 | 0.001 | 0.0042 |
| ENSG00000254486 | LINC02547 | -1.734 | 0.4 | -4.338 | 0 | 0.0001 |
| ENSG00000103599 | IQCH | -1.732 | 0.545 | -3.18 | 0.0015 | 0.0057 |
| ENSG00000125148 | MT2A | -1.731 | 0.231 | -7.477 | 0 | 0 |
| ENSG00000279539 |  | -1.731 | 0.43 | -4.024 | 0.0001 | 0.0003 |
| ENSG00000228801 | LOC102724330 | -1.731 | 0.541 | -3.2 | 0.0014 | 0.0053 |
| ENSG00000227811 | INKA2-AS1 | -1.731 | 0.689 | -2.513 | 0.012 | 0.0326 |
| ENSG00000215417 | MIR17HG | -1.73 | 0.375 | -4.606 | 0 | 0 |
| ENSG00000157653 | C9orf43 | -1.73 | 0.449 | -3.856 | 0.0001 | 0.0006 |
| ENSG00000155897 | ADCY8 | -1.73 | 0.644 | -2.688 | 0.0072 | 0.0216 |
| ENSG00000269899 |  | -1.729 | 0.63 | -2.743 | 0.0061 | 0.0188 |
| ENSG00000145022 | TCTA | -1.728 | 0.241 | -7.157 | 0 | 0 |
| ENSG00000187634 | SAMD11 | -1.728 | 0.406 | -4.256 | 0 | 0.0001 |
| ENSG00000248257 |  | -1.727 | 0.296 | -5.846 | 0 | 0 |
| ENSG00000228300 | FAM174C | -1.726 | 0.222 | -7.786 | 0 | 0 |
| ENSG00000261008 | LINC01572 | -1.726 | 0.507 | -3.407 | 0.0007 | 0.0029 |
| ENSG00000187792 | ZNF70 | -1.725 | 0.397 | -4.346 | 0 | 0.0001 |
| ENSG00000270083 |  | -1.725 | 0.681 | -2.532 | 0.0113 | 0.0313 |
| ENSG00000214401 | KANSL1-AS1 | -1.724 | 0.455 | -3.793 | 0.0001 | 0.0008 |
| ENSG00000101608 | MYL12A | -1.723 | 0.245 | -7.04 | 0 | 0 |
| ENSG00000229388 | LINC01715 | -1.722 | 0.341 | -5.055 | 0 | 0 |
| ENSG00000187514 | PTMA | -1.721 | 0.208 | -8.28 | 0 | 0 |
| ENSG00000175305 | CCNE2 | -1.721 | 0.298 | -5.778 | 0 | 0 |
| ENSG00000120149 | MSX2 | -1.72 | 0.328 | -5.251 | 0 | 0 |
| ENSG00000235316 |  | -1.72 | 0.383 | -4.486 | 0 | 0.0001 |
| ENSG00000272247 |  | -1.72 | 0.402 | -4.281 | 0 | 0.0001 |
| ENSG00000125848 | FLRT3 | -1.718 | 0.233 | -7.361 | 0 | 0 |
| ENSG00000279673 |  | -1.718 | 0.733 | -2.343 | 0.0191 | 0.0481 |
| ENSG00000099385 | BCL7C | -1.717 | 0.24 | -7.151 | 0 | 0 |
| ENSG00000188612 | SUMO2 | -1.716 | 0.221 | -7.746 | 0 | 0 |
| ENSG00000257097 |  | -1.715 | 0.352 | -4.871 | 0 | 0 |
| ENSG00000233693 | PBX1-AS1 | -1.715 | 0.534 | -3.213 | 0.0013 | 0.0051 |
| ENSG00000275964 |  | -1.714 | 0.501 | -3.423 | 0.0006 | 0.0027 |
| ENSG00000258553 |  | -1.714 | 0.643 | -2.664 | 0.0077 | 0.0228 |
| ENSG00000268575 |  | -1.714 | 0.646 | -2.655 | 0.0079 | 0.0234 |
| ENSG00000158062 | UBXN11 | -1.712 | 0.277 | -6.181 | 0 | 0 |
| ENSG00000127774 | EMC6 | -1.711 | 0.543 | -3.15 | 0.0016 | 0.0062 |
| ENSG00000272186 |  | -1.711 | 0.695 | -2.463 | 0.0138 | 0.0367 |
| ENSG00000224505 |  | -1.711 | 0.722 | -2.371 | 0.0177 | 0.0453 |
| ENSG00000077713 | SLC25A43 | -1.71 | 0.293 | -5.835 | 0 | 0 |
| ENSG00000262185 | LINC02861 | -1.71 | 0.478 | -3.58 | 0.0003 | 0.0016 |
| ENSG00000213145 | CRIP1 | -1.706 | 0.62 | -2.752 | 0.0059 | 0.0184 |
| ENSG00000224936 |  | -1.706 | 0.622 | -2.74 | 0.0061 | 0.0189 |
| ENSG00000108439 | PNPO | -1.705 | 0.273 | -6.246 | 0 | 0 |
| ENSG00000258813 |  | -1.705 | 0.401 | -4.251 | 0 | 0.0001 |
| ENSG00000212195 |  | -1.705 | 0.552 | -3.088 | 0.002 | 0.0073 |
| ENSG00000274104 |  | -1.705 | 0.593 | -2.873 | 0.0041 | 0.0134 |
| ENSG00000186847 | KRT14 | -1.705 | 0.63 | -2.707 | 0.0068 | 0.0205 |
| ENSG00000118600 | RXYLT1 | -1.704 | 0.28 | -6.091 | 0 | 0 |
| ENSG00000188693 | CYP51A1-AS1 | -1.704 | 0.634 | -2.688 | 0.0072 | 0.0216 |
| ENSG00000174365 | SNHG11 | -1.703 | 0.252 | -6.766 | 0 | 0 |
| ENSG00000186615 | KTN1-AS1 | -1.701 | 0.488 | -3.485 | 0.0005 | 0.0022 |
| ENSG00000251417 |  | -1.701 | 0.728 | -2.335 | 0.0196 | 0.049 |
| ENSG00000118655 | DCLRE1B | -1.7 | 0.274 | -6.205 | 0 | 0 |
| ENSG00000101955 | SRPX | -1.7 | 0.248 | -6.846 | 0 | 0 |
| ENSG00000143971 | ETAA1 | -1.7 | 0.341 | -4.991 | 0 | 0 |
| ENSG00000105649 | RAB3A | -1.699 | 0.396 | -4.292 | 0 | 0.0001 |
| ENSG00000067829 | IDH3G | -1.698 | 0.254 | -6.684 | 0 | 0 |
| ENSG00000267325 |  | -1.694 | 0.59 | -2.87 | 0.0041 | 0.0135 |
| ENSG00000189227 | C15orf61 | -1.692 | 0.284 | -5.958 | 0 | 0 |
| ENSG00000227908 | FLJ31104 | -1.691 | 0.408 | -4.143 | 0 | 0.0002 |
| ENSG00000175229 | GAL3ST3 | -1.69 | 0.462 | -3.658 | 0.0003 | 0.0013 |
| ENSG00000204301 | NOTCH4 | -1.689 | 0.421 | -4.014 | 0.0001 | 0.0004 |
| ENSG00000273064 |  | -1.688 | 0.482 | -3.505 | 0.0005 | 0.0021 |
| ENSG00000185332 | TMEM105 | -1.684 | 0.685 | -2.457 | 0.014 | 0.0372 |
| ENSG00000128656 | CHN1 | -1.683 | 0.256 | -6.566 | 0 | 0 |
| ENSG00000267858 | MZF1-AS1 | -1.683 | 0.418 | -4.027 | 0.0001 | 0.0003 |
| ENSG00000258346 |  | -1.682 | 0.693 | -2.428 | 0.0152 | 0.0398 |
| ENSG00000257135 |  | -1.681 | 0.412 | -4.08 | 0 | 0.0003 |
| ENSG00000237210 | LOC100130121 | -1.681 | 0.721 | -2.333 | 0.0196 | 0.0492 |
| ENSG00000168077 | SCARA3 | -1.68 | 0.262 | -6.4 | 0 | 0 |
| ENSG00000207205 | RNVU1-15 | -1.679 | 0.532 | -3.156 | 0.0016 | 0.0061 |
| ENSG00000231402 | WASF5P | -1.679 | 0.565 | -2.969 | 0.003 | 0.0103 |
| ENSG00000223473 |  | -1.678 | 0.581 | -2.888 | 0.0039 | 0.0128 |
| ENSG00000170345 | FOS | -1.677 | 0.234 | -7.168 | 0 | 0 |
| ENSG00000130985 | UBA1 | -1.677 | 0.209 | -8.036 | 0 | 0 |
| ENSG00000237605 |  | -1.677 | 0.289 | -5.8 | 0 | 0 |
| ENSG00000265480 | KRT18P55 | -1.677 | 0.557 | -3.01 | 0.0026 | 0.0092 |
| ENSG00000169570 | DTWD2 | -1.677 | 0.708 | -2.368 | 0.0179 | 0.0455 |
| ENSG00000155760 | FZD7 | -1.676 | 0.217 | -7.723 | 0 | 0 |
| ENSG00000133105 | RXFP2 | -1.676 | 0.674 | -2.485 | 0.0129 | 0.0349 |
| ENSG00000153250 | RBMS1 | -1.675 | 0.25 | -6.709 | 0 | 0 |
| ENSG00000147324 | MFHAS1 | -1.674 | 0.277 | -6.055 | 0 | 0 |
| ENSG00000259788 |  | -1.674 | 0.614 | -2.725 | 0.0064 | 0.0197 |
| ENSG00000167600 | CYP2S1 | -1.672 | 0.434 | -3.85 | 0.0001 | 0.0007 |
| ENSG00000199347 | RNU5E-1 | -1.671 | 0.447 | -3.74 | 0.0002 | 0.001 |
| ENSG00000113108 | APBB3 | -1.67 | 0.305 | -5.477 | 0 | 0 |
| ENSG00000120925 | RNF170 | -1.669 | 0.3 | -5.568 | 0 | 0 |
| ENSG00000176731 | RBIS | -1.667 | 0.362 | -4.602 | 0 | 0 |
| ENSG00000126524 | SBDS | -1.666 | 0.245 | -6.789 | 0 | 0 |
| ENSG00000233922 | LINC01694 | -1.666 | 0.696 | -2.393 | 0.0167 | 0.043 |
| ENSG00000267100 | ILF3-DT | -1.665 | 0.22 | -7.577 | 0 | 0 |
| ENSG00000108839 | ALOX12 | -1.665 | 0.412 | -4.04 | 0.0001 | 0.0003 |
| ENSG00000100156 | SLC16A8 | -1.663 | 0.582 | -2.856 | 0.0043 | 0.014 |
| ENSG00000102901 | CENPT | -1.662 | 0.262 | -6.337 | 0 | 0 |
| ENSG00000250742 | LINC02381 | -1.662 | 0.628 | -2.648 | 0.0081 | 0.0237 |
| ENSG00000127586 | CHTF18 | -1.661 | 0.308 | -5.396 | 0 | 0 |
| ENSG00000176903 | PNMA1 | -1.661 | 0.23 | -7.209 | 0 | 0 |
| ENSG00000125249 | RAP2A | -1.661 | 0.275 | -6.043 | 0 | 0 |
| ENSG00000170385 | SLC30A1 | -1.661 | 0.224 | -7.413 | 0 | 0 |
| ENSG00000108641 | B9D1 | -1.66 | 0.379 | -4.384 | 0 | 0.0001 |
| ENSG00000272768 |  | -1.66 | 0.459 | -3.614 | 0.0003 | 0.0015 |
| ENSG00000275479 |  | -1.66 | 0.525 | -3.162 | 0.0016 | 0.006 |
| ENSG00000171533 | MAP6 | -1.66 | 0.611 | -2.715 | 0.0066 | 0.0202 |
| ENSG00000176293 | ZNF135 | -1.657 | 0.672 | -2.466 | 0.0137 | 0.0364 |
| ENSG00000257594 | GALNT4 | -1.657 | 0.686 | -2.416 | 0.0157 | 0.0409 |
| ENSG00000127824 | TUBA4A | -1.656 | 0.321 | -5.154 | 0 | 0 |
| ENSG00000182179 | UBA7 | -1.656 | 0.35 | -4.736 | 0 | 0 |
| ENSG00000275765 |  | -1.656 | 0.425 | -3.9 | 0.0001 | 0.0005 |
| ENSG00000142798 | HSPG2 | -1.654 | 0.268 | -6.165 | 0 | 0 |
| ENSG00000182223 | ZAR1 | -1.654 | 0.494 | -3.347 | 0.0008 | 0.0034 |
| ENSG00000107821 | KAZALD1 | -1.653 | 0.28 | -5.901 | 0 | 0 |
| ENSG00000283341 |  | -1.653 | 0.516 | -3.205 | 0.0013 | 0.0053 |
| ENSG00000235106 | BRD3OS | -1.652 | 0.294 | -5.621 | 0 | 0 |
| ENSG00000236986 |  | -1.651 | 0.66 | -2.5 | 0.0124 | 0.0337 |
| ENSG00000148219 | ASTN2 | -1.649 | 0.352 | -4.687 | 0 | 0 |
| ENSG00000101347 | SAMHD1 | -1.648 | 0.239 | -6.897 | 0 | 0 |
| ENSG00000160282 | FTCD | -1.648 | 0.319 | -5.164 | 0 | 0 |
| ENSG00000177337 |  | -1.648 | 0.298 | -5.539 | 0 | 0 |
| ENSG00000160886 | LY6K | -1.647 | 0.272 | -6.046 | 0 | 0 |
| ENSG00000126217 | MCF2L | -1.647 | 0.287 | -5.739 | 0 | 0 |
| ENSG00000116198 | CEP104 | -1.643 | 0.286 | -5.756 | 0 | 0 |
| ENSG00000227959 |  | -1.643 | 0.386 | -4.257 | 0 | 0.0001 |
| ENSG00000273080 |  | -1.642 | 0.639 | -2.571 | 0.0101 | 0.0285 |
| ENSG00000070540 | WIPI1 | -1.641 | 0.265 | -6.183 | 0 | 0 |
| ENSG00000196368 | NUDT11 | -1.639 | 0.391 | -4.19 | 0 | 0.0002 |
| ENSG00000248975 |  | -1.639 | 0.671 | -2.443 | 0.0146 | 0.0385 |
| ENSG00000111961 | SASH1 | -1.638 | 0.242 | -6.756 | 0 | 0 |
| ENSG00000233538 |  | -1.638 | 0.465 | -3.525 | 0.0004 | 0.002 |
| ENSG00000271576 |  | -1.637 | 0.32 | -5.115 | 0 | 0 |
| ENSG00000257556 |  | -1.636 | 0.585 | -2.796 | 0.0052 | 0.0164 |
| ENSG00000273176 |  | -1.636 | 0.599 | -2.732 | 0.0063 | 0.0193 |
| ENSG00000259826 | LOC112267983 | -1.635 | 0.331 | -4.936 | 0 | 0 |
| ENSG00000137331 | IER3 | -1.635 | 0.225 | -7.284 | 0 | 0 |
| ENSG00000204420 | MPIG6B | -1.635 | 0.582 | -2.81 | 0.005 | 0.0158 |
| ENSG00000234432 | LOC100129484 | -1.634 | 0.246 | -6.645 | 0 | 0 |
| ENSG00000162004 | CCDC78 | -1.634 | 0.412 | -3.971 | 0.0001 | 0.0004 |
| ENSG00000257950 | P2RX5-TAX1BP3 | -1.632 | 0.3 | -5.446 | 0 | 0 |
| ENSG00000213742 | ZNF337-AS1 | -1.632 | 0.461 | -3.541 | 0.0004 | 0.0019 |
| ENSG00000235848 |  | -1.631 | 0.463 | -3.525 | 0.0004 | 0.002 |
| ENSG00000272787 |  | -1.631 | 0.637 | -2.56 | 0.0105 | 0.0293 |
| ENSG00000237301 |  | -1.63 | 0.561 | -2.904 | 0.0037 | 0.0123 |
| ENSG00000103254 | ANTKMT | -1.629 | 0.301 | -5.408 | 0 | 0 |
| ENSG00000279452 |  | -1.629 | 0.351 | -4.645 | 0 | 0 |
| ENSG00000179743 | FLJ37453 | -1.627 | 0.285 | -5.705 | 0 | 0 |
| ENSG00000256092 |  | -1.626 | 0.299 | -5.433 | 0 | 0 |
| ENSG00000167617 | CDC42EP5 | -1.626 | 0.294 | -5.538 | 0 | 0 |
| ENSG00000244167 |  | -1.625 | 0.56 | -2.901 | 0.0037 | 0.0124 |
| ENSG00000243406 | MRPS31P5 | -1.625 | 0.643 | -2.528 | 0.0115 | 0.0316 |
| ENSG00000125046 | SSUH2 | -1.624 | 0.44 | -3.688 | 0.0002 | 0.0011 |
| ENSG00000184009 | ACTG1 | -1.623 | 0.256 | -6.338 | 0 | 0 |
| ENSG00000248690 |  | -1.623 | 0.367 | -4.419 | 0 | 0.0001 |
| ENSG00000187583 | PLEKHN1 | -1.623 | 0.373 | -4.35 | 0 | 0.0001 |
| ENSG00000207752 | MIR199A1 | -1.623 | 0.507 | -3.202 | 0.0014 | 0.0053 |
| ENSG00000233011 |  | -1.623 | 0.569 | -2.852 | 0.0043 | 0.0141 |
| ENSG00000183155 | RABIF | -1.621 | 0.279 | -5.813 | 0 | 0 |
| ENSG00000214960 | CRPPA | -1.621 | 0.422 | -3.84 | 0.0001 | 0.0007 |
| ENSG00000261079 |  | -1.621 | 0.587 | -2.763 | 0.0057 | 0.0179 |
| ENSG00000283696 |  | -1.621 | 0.64 | -2.532 | 0.0114 | 0.0313 |
| ENSG00000277382 |  | -1.619 | 0.537 | -3.014 | 0.0026 | 0.0091 |
| ENSG00000253250 | C8orf88 | -1.618 | 0.505 | -3.206 | 0.0013 | 0.0053 |
| ENSG00000101842 | VSIG1 | -1.618 | 0.511 | -3.163 | 0.0016 | 0.0059 |
| ENSG00000108381 | ASPA | -1.618 | 0.677 | -2.389 | 0.0169 | 0.0434 |
| ENSG00000238266 | LINC00707 | -1.616 | 0.42 | -3.846 | 0.0001 | 0.0007 |
| ENSG00000261824 | LINC00662 | -1.615 | 0.298 | -5.411 | 0 | 0 |
| ENSG00000273329 |  | -1.615 | 0.318 | -5.079 | 0 | 0 |
| ENSG00000008735 | MAPK8IP2 | -1.615 | 0.456 | -3.539 | 0.0004 | 0.0019 |
| ENSG00000135899 | SP110 | -1.613 | 0.678 | -2.381 | 0.0173 | 0.0443 |
| ENSG00000196912 | ANKRD36B | -1.611 | 0.575 | -2.8 | 0.0051 | 0.0162 |
| ENSG00000268858 | LOC112268269 | -1.609 | 0.299 | -5.382 | 0 | 0 |
| ENSG00000266524 | GDF10 | -1.608 | 0.647 | -2.483 | 0.013 | 0.035 |
| ENSG00000176912 | TYMSOS | -1.607 | 0.462 | -3.478 | 0.0005 | 0.0023 |
| ENSG00000186260 | MRTFB | -1.607 | 0.547 | -2.939 | 0.0033 | 0.0112 |
| ENSG00000280414 |  | -1.607 | 0.624 | -2.578 | 0.0099 | 0.0281 |
| ENSG00000250959 | GLUD1P3 | -1.606 | 0.494 | -3.252 | 0.0011 | 0.0046 |
| ENSG00000176692 | FOXC2 | -1.605 | 0.346 | -4.632 | 0 | 0 |
| ENSG00000138771 | SHROOM3 | -1.605 | 0.255 | -6.284 | 0 | 0 |
| ENSG00000180769 | WDFY3-AS2 | -1.605 | 0.546 | -2.938 | 0.0033 | 0.0112 |
| ENSG00000172059 | KLF11 | -1.604 | 0.286 | -5.609 | 0 | 0 |
| ENSG00000280890 |  | -1.604 | 0.39 | -4.113 | 0 | 0.0003 |
| ENSG00000227053 | MUC12-AS1 | -1.604 | 0.641 | -2.504 | 0.0123 | 0.0334 |
| ENSG00000187961 | KLHL17 | -1.602 | 0.287 | -5.575 | 0 | 0 |
| ENSG00000165516 | KLHDC2 | -1.601 | 0.309 | -5.187 | 0 | 0 |
| ENSG00000137959 | IFI44L | -1.601 | 0.432 | -3.71 | 0.0002 | 0.0011 |
| ENSG00000187066 | TMEM262 | -1.601 | 0.578 | -2.769 | 0.0056 | 0.0176 |
| ENSG00000183688 | RFLNB | -1.6 | 0.292 | -5.486 | 0 | 0 |
| ENSG00000182103 | FAM181B | -1.6 | 0.649 | -2.467 | 0.0136 | 0.0363 |
| ENSG00000184838 | PRR16 | -1.599 | 0.288 | -5.549 | 0 | 0 |
| ENSG00000236530 | KPNA2P1 | -1.599 | 0.664 | -2.409 | 0.016 | 0.0415 |
| ENSG00000147804 | SLC39A4 | -1.598 | 0.407 | -3.923 | 0.0001 | 0.0005 |
| ENSG00000204388 | HSPA1B | -1.597 | 0.239 | -6.673 | 0 | 0 |
| ENSG00000134970 | TMED7 | -1.597 | 0.246 | -6.507 | 0 | 0 |
| ENSG00000138100 | TRIM54 | -1.597 | 0.506 | -3.157 | 0.0016 | 0.006 |
| ENSG00000164342 | TLR3 | -1.596 | 0.495 | -3.226 | 0.0013 | 0.005 |
| ENSG00000187688 | TRPV2 | -1.595 | 0.349 | -4.573 | 0 | 0 |
| ENSG00000105696 | TMEM59L | -1.594 | 0.511 | -3.12 | 0.0018 | 0.0067 |
| ENSG00000197162 | ZNF785 | -1.593 | 0.324 | -4.92 | 0 | 0 |
| ENSG00000198721 | ECI2 | -1.592 | 0.281 | -5.662 | 0 | 0 |
| ENSG00000170310 | STX8 | -1.592 | 0.309 | -5.147 | 0 | 0 |
| ENSG00000270441 |  | -1.592 | 0.64 | -2.487 | 0.0129 | 0.0347 |
| ENSG00000117984 | CTSD | -1.591 | 0.262 | -6.08 | 0 | 0 |
| ENSG00000099625 | CBARP | -1.591 | 0.289 | -5.498 | 0 | 0 |
| ENSG00000280424 | LOC730668 | -1.59 | 0.66 | -2.409 | 0.016 | 0.0415 |
| ENSG00000257702 | LBX2-AS1 | -1.589 | 0.363 | -4.38 | 0 | 0.0001 |
| ENSG00000129810 | SGO1 | -1.589 | 0.357 | -4.455 | 0 | 0.0001 |
| ENSG00000273802 | H2BC8 | -1.587 | 0.224 | -7.076 | 0 | 0 |
| ENSG00000137834 | SMAD6 | -1.587 | 0.452 | -3.51 | 0.0004 | 0.0021 |
| ENSG00000203724 | C1orf53 | -1.585 | 0.42 | -3.771 | 0.0002 | 0.0009 |
| ENSG00000119862 | LGALSL | -1.584 | 0.263 | -6.013 | 0 | 0 |
| ENSG00000184402 | SS18L1 | -1.584 | 0.375 | -4.224 | 0 | 0.0002 |
| ENSG00000118420 | UBE3D | -1.584 | 0.475 | -3.331 | 0.0009 | 0.0036 |
| ENSG00000132359 | RAP1GAP2 | -1.583 | 0.298 | -5.309 | 0 | 0 |
| ENSG00000204389 | HSPA1A | -1.583 | 0.267 | -5.924 | 0 | 0 |
| ENSG00000279584 |  | -1.583 | 0.386 | -4.103 | 0 | 0.0003 |
| ENSG00000260777 |  | -1.583 | 0.523 | -3.025 | 0.0025 | 0.0088 |
| ENSG00000188365 |  | -1.583 | 0.63 | -2.514 | 0.0119 | 0.0326 |
| ENSG00000148331 | ASB6 | -1.582 | 0.249 | -6.345 | 0 | 0 |
| ENSG00000121900 | TMEM54 | -1.582 | 0.392 | -4.039 | 0.0001 | 0.0003 |
| ENSG00000179818 | PCBP1-AS1 | -1.581 | 0.239 | -6.605 | 0 | 0 |
| ENSG00000176533 | GNG7 | -1.581 | 0.432 | -3.661 | 0.0003 | 0.0013 |
| ENSG00000225313 |  | -1.581 | 0.444 | -3.564 | 0.0004 | 0.0017 |
| ENSG00000254469 | LOC100133315 | -1.581 | 0.55 | -2.877 | 0.004 | 0.0132 |
| ENSG00000254505 | CHMP4A | -1.581 | 0.582 | -2.719 | 0.0065 | 0.0199 |
| ENSG00000133477 | FAM83F | -1.581 | 0.636 | -2.486 | 0.0129 | 0.0348 |
| ENSG00000169252 | ADRB2 | -1.58 | 0.228 | -6.927 | 0 | 0 |
| ENSG00000276727 |  | -1.58 | 0.581 | -2.72 | 0.0065 | 0.0199 |
| ENSG00000186665 | C17orf58 | -1.578 | 0.352 | -4.488 | 0 | 0.0001 |
| ENSG00000102100 | SLC35A2 | -1.576 | 0.253 | -6.238 | 0 | 0 |
| ENSG00000187601 | MAGEH1 | -1.576 | 0.258 | -6.109 | 0 | 0 |
| ENSG00000271797 |  | -1.576 | 0.447 | -3.523 | 0.0004 | 0.002 |
| ENSG00000135124 | P2RX4 | -1.575 | 0.242 | -6.515 | 0 | 0 |
| ENSG00000155755 | TMEM237 | -1.575 | 0.258 | -6.1 | 0 | 0 |
| ENSG00000269800 |  | -1.574 | 0.308 | -5.102 | 0 | 0 |
| ENSG00000198455 | ZXDB | -1.574 | 0.245 | -6.414 | 0 | 0 |
| ENSG00000213977 | TAX1BP3 | -1.574 | 0.288 | -5.471 | 0 | 0 |
| ENSG00000034510 | TMSB10 | -1.573 | 0.215 | -7.331 | 0 | 0 |
| ENSG00000250325 |  | -1.573 | 0.364 | -4.326 | 0 | 0.0001 |
| ENSG00000207357 | RNU6-2 | -1.572 | 0.328 | -4.788 | 0 | 0 |
| ENSG00000155868 | MED7 | -1.572 | 0.339 | -4.632 | 0 | 0 |
| ENSG00000213654 | GPSM3 | -1.572 | 0.386 | -4.076 | 0 | 0.0003 |
| ENSG00000215068 | LOC153684 | -1.572 | 0.446 | -3.528 | 0.0004 | 0.0019 |
| ENSG00000273010 |  | -1.572 | 0.476 | -3.303 | 0.001 | 0.0039 |
| ENSG00000279529 |  | -1.571 | 0.341 | -4.603 | 0 | 0 |
| ENSG00000280604 |  | -1.571 | 0.393 | -3.995 | 0.0001 | 0.0004 |
| ENSG00000140481 | CCDC33 | -1.571 | 0.632 | -2.484 | 0.013 | 0.0349 |
| ENSG00000153815 | CMIP | -1.569 | 0.333 | -4.716 | 0 | 0 |
| ENSG00000268364 |  | -1.569 | 0.526 | -2.983 | 0.0029 | 0.0099 |
| ENSG00000153391 | INO80C | -1.568 | 0.444 | -3.527 | 0.0004 | 0.0019 |
| ENSG00000179528 | LBX2 | -1.568 | 0.453 | -3.463 | 0.0005 | 0.0024 |
| ENSG00000254554 |  | -1.567 | 0.437 | -3.584 | 0.0003 | 0.0016 |
| ENSG00000183454 | GRIN2A | -1.567 | 0.537 | -2.919 | 0.0035 | 0.0118 |
| ENSG00000166579 | NDEL1 | -1.565 | 0.284 | -5.519 | 0 | 0 |
| ENSG00000168066 | SF1 | -1.565 | 0.233 | -6.708 | 0 | 0 |
| ENSG00000257086 |  | -1.562 | 0.589 | -2.651 | 0.008 | 0.0236 |
| ENSG00000169213 | RAB3B | -1.561 | 0.251 | -6.228 | 0 | 0 |
| ENSG00000256591 |  | -1.561 | 0.428 | -3.644 | 0.0003 | 0.0013 |
| ENSG00000181638 | ZFP41 | -1.561 | 0.43 | -3.632 | 0.0003 | 0.0014 |
| ENSG00000140650 | PMM2 | -1.56 | 0.273 | -5.72 | 0 | 0 |
| ENSG00000103355 | PRSS33 | -1.557 | 0.577 | -2.699 | 0.007 | 0.021 |
| ENSG00000137486 | ARRB1 | -1.555 | 0.391 | -3.971 | 0.0001 | 0.0004 |
| ENSG00000176597 | B3GNT5 | -1.555 | 0.494 | -3.144 | 0.0017 | 0.0063 |
| ENSG00000169223 | LMAN2 | -1.554 | 0.25 | -6.225 | 0 | 0 |
| ENSG00000155254 | MARVELD1 | -1.554 | 0.215 | -7.239 | 0 | 0 |
| ENSG00000174292 | TNK1 | -1.554 | 0.557 | -2.792 | 0.0052 | 0.0166 |
| ENSG00000165655 | ZNF503 | -1.553 | 0.349 | -4.456 | 0 | 0.0001 |
| ENSG00000181481 | RNF135 | -1.552 | 0.267 | -5.808 | 0 | 0 |
| ENSG00000145979 | TBC1D7 | -1.551 | 0.269 | -5.768 | 0 | 0 |
| ENSG00000080824 | HSP90AA1 | -1.551 | 0.21 | -7.375 | 0 | 0 |
| ENSG00000169184 | MN1 | -1.549 | 0.25 | -6.195 | 0 | 0 |
| ENSG00000256269 | HMBS | -1.549 | 0.243 | -6.378 | 0 | 0 |
| ENSG00000280162 |  | -1.549 | 0.429 | -3.608 | 0.0003 | 0.0015 |
| ENSG00000258317 |  | -1.549 | 0.535 | -2.895 | 0.0038 | 0.0126 |
| ENSG00000183020 | AP2A2 | -1.548 | 0.24 | -6.461 | 0 | 0 |
| ENSG00000168286 | THAP11 | -1.548 | 0.242 | -6.409 | 0 | 0 |
| ENSG00000198576 | ARC | -1.547 | 0.33 | -4.689 | 0 | 0 |
| ENSG00000183340 | JRKL | -1.547 | 0.271 | -5.71 | 0 | 0 |
| ENSG00000133027 | PEMT | -1.544 | 0.295 | -5.233 | 0 | 0 |
| ENSG00000120802 | TMPO | -1.544 | 0.237 | -6.516 | 0 | 0 |
| ENSG00000099256 | PRTFDC1 | -1.543 | 0.292 | -5.293 | 0 | 0 |
| ENSG00000063660 | GPC1 | -1.542 | 0.245 | -6.287 | 0 | 0 |
| ENSG00000070404 | FSTL3 | -1.542 | 0.294 | -5.246 | 0 | 0 |
| ENSG00000073146 | MOV10L1 | -1.542 | 0.446 | -3.46 | 0.0005 | 0.0024 |
| ENSG00000274292 |  | -1.542 | 0.57 | -2.706 | 0.0068 | 0.0206 |
| ENSG00000115540 | MOB4 | -1.541 | 0.357 | -4.315 | 0 | 0.0001 |
| ENSG00000272476 |  | -1.54 | 0.478 | -3.225 | 0.0013 | 0.005 |
| ENSG00000001460 | STPG1 | -1.539 | 0.272 | -5.668 | 0 | 0 |
| ENSG00000135617 | PRADC1 | -1.539 | 0.284 | -5.417 | 0 | 0 |
| ENSG00000182257 |  | -1.539 | 0.653 | -2.358 | 0.0184 | 0.0466 |
| ENSG00000174206 | C12orf66 | -1.538 | 0.269 | -5.717 | 0 | 0 |
| ENSG00000213190 | MLLT11 | -1.538 | 0.305 | -5.039 | 0 | 0 |
| ENSG00000079462 | PAFAH1B3 | -1.538 | 0.249 | -6.182 | 0 | 0 |
| ENSG00000169908 | TM4SF1 | -1.538 | 0.372 | -4.138 | 0 | 0.0002 |
| ENSG00000200156 | RNU5B-1 | -1.538 | 0.512 | -3.006 | 0.0026 | 0.0092 |
| ENSG00000117906 | RCN2 | -1.535 | 0.23 | -6.678 | 0 | 0 |
| ENSG00000081377 | CDC14B | -1.535 | 0.273 | -5.624 | 0 | 0 |
| ENSG00000260062 |  | -1.535 | 0.491 | -3.126 | 0.0018 | 0.0066 |
| ENSG00000137203 | TFAP2A | -1.534 | 0.262 | -5.857 | 0 | 0 |
| ENSG00000077585 | GPR137B | -1.533 | 0.3 | -5.119 | 0 | 0 |
| ENSG00000260588 |  | -1.531 | 0.377 | -4.064 | 0 | 0.0003 |
| ENSG00000272631 |  | -1.531 | 0.41 | -3.734 | 0.0002 | 0.001 |
| ENSG00000168806 | LCMT2 | -1.53 | 0.29 | -5.273 | 0 | 0 |
| ENSG00000258694 |  | -1.53 | 0.584 | -2.618 | 0.0088 | 0.0254 |
| ENSG00000089775 | ZBTB25 | -1.527 | 0.327 | -4.669 | 0 | 0 |
| ENSG00000136235 | GPNMB | -1.526 | 0.21 | -7.264 | 0 | 0 |
| ENSG00000227105 | PARP1P1 | -1.526 | 0.403 | -3.784 | 0.0002 | 0.0008 |
| ENSG00000214087 | ARL16 | -1.524 | 0.256 | -5.962 | 0 | 0 |
| ENSG00000106348 | IMPDH1 | -1.521 | 0.233 | -6.529 | 0 | 0 |
| ENSG00000176871 | WSB2 | -1.52 | 0.221 | -6.891 | 0 | 0 |
| ENSG00000123106 | CCDC91 | -1.52 | 0.302 | -5.033 | 0 | 0 |
| ENSG00000267519 |  | -1.519 | 0.221 | -6.876 | 0 | 0 |
| ENSG00000123143 | PKN1 | -1.519 | 0.259 | -5.865 | 0 | 0 |
| ENSG00000170855 | TRIAP1 | -1.519 | 0.22 | -6.902 | 0 | 0 |
| ENSG00000188158 | NHS | -1.519 | 0.447 | -3.397 | 0.0007 | 0.0029 |
| ENSG00000161677 | JOSD2 | -1.518 | 0.29 | -5.237 | 0 | 0 |
| ENSG00000259721 | LOC100131315 | -1.517 | 0.251 | -6.044 | 0 | 0 |
| ENSG00000244045 | TMEM199 | -1.515 | 0.3 | -5.055 | 0 | 0 |
| ENSG00000172375 | C2CD2L | -1.515 | 0.335 | -4.524 | 0 | 0 |
| ENSG00000235781 |  | -1.514 | 0.552 | -2.742 | 0.0061 | 0.0188 |
| ENSG00000189369 | GSPT2 | -1.512 | 0.254 | -5.953 | 0 | 0 |
| ENSG00000224738 |  | -1.512 | 0.461 | -3.276 | 0.0011 | 0.0043 |
| ENSG00000266411 |  | -1.509 | 0.602 | -2.506 | 0.0122 | 0.0332 |
| ENSG00000130522 | JUND | -1.508 | 0.225 | -6.691 | 0 | 0 |
| ENSG00000169020 | ATP5ME | -1.508 | 0.265 | -5.685 | 0 | 0 |
| ENSG00000102054 | RBBP7 | -1.508 | 0.228 | -6.604 | 0 | 0 |
| ENSG00000243317 | STMP1 | -1.506 | 0.303 | -4.968 | 0 | 0 |
| ENSG00000134480 | CCNH | -1.506 | 0.291 | -5.183 | 0 | 0 |
| ENSG00000170340 | B3GNT2 | -1.506 | 0.35 | -4.297 | 0 | 0.0001 |
| ENSG00000131871 | SELENOS | -1.505 | 0.221 | -6.808 | 0 | 0 |
| ENSG00000245522 | LINC02709 | -1.505 | 0.385 | -3.908 | 0.0001 | 0.0005 |
| ENSG00000001626 | CFTR | -1.505 | 0.57 | -2.643 | 0.0082 | 0.024 |
| ENSG00000184497 | TMEM255B | -1.503 | 0.279 | -5.395 | 0 | 0 |
| ENSG00000167552 | TUBA1A | -1.503 | 0.362 | -4.146 | 0 | 0.0002 |
| ENSG00000230513 | THAP7-AS1 | -1.503 | 0.427 | -3.517 | 0.0004 | 0.002 |
| ENSG00000205592 | MUC19 | -1.503 | 0.469 | -3.201 | 0.0014 | 0.0053 |
| ENSG00000233654 | LOC105376748 | -1.503 | 0.556 | -2.702 | 0.0069 | 0.0208 |
| ENSG00000167280 | ENGASE | -1.499 | 0.357 | -4.194 | 0 | 0.0002 |
| ENSG00000186017 | ZNF566 | -1.499 | 0.359 | -4.173 | 0 | 0.0002 |
| ENSG00000197879 | MYO1C | -1.498 | 0.233 | -6.438 | 0 | 0 |
| ENSG00000260233 |  | -1.498 | 0.291 | -5.142 | 0 | 0 |
| ENSG00000184508 | HDDC3 | -1.498 | 0.467 | -3.212 | 0.0013 | 0.0052 |
| ENSG00000234882 | EIF3EP1 | -1.498 | 0.616 | -2.429 | 0.0151 | 0.0397 |
| ENSG00000263272 |  | -1.497 | 0.494 | -3.031 | 0.0024 | 0.0086 |
| ENSG00000225173 |  | -1.495 | 0.375 | -3.989 | 0.0001 | 0.0004 |
| ENSG00000188243 | COMMD6 | -1.494 | 0.281 | -5.323 | 0 | 0 |
| ENSG00000188242 | PP7080 | -1.494 | 0.299 | -4.996 | 0 | 0 |
| ENSG00000205084 | TMEM231 | -1.493 | 0.433 | -3.445 | 0.0006 | 0.0025 |
| ENSG00000280426 |  | -1.492 | 0.536 | -2.784 | 0.0054 | 0.0169 |
| ENSG00000107130 | NCS1 | -1.491 | 0.23 | -6.487 | 0 | 0 |
| ENSG00000261251 |  | -1.491 | 0.436 | -3.423 | 0.0006 | 0.0027 |
| ENSG00000177575 | CD163 | -1.489 | 0.604 | -2.463 | 0.0138 | 0.0367 |
| ENSG00000089693 | MLF2 | -1.487 | 0.249 | -5.974 | 0 | 0 |
| ENSG00000159267 | HLCS | -1.487 | 0.312 | -4.76 | 0 | 0 |
| ENSG00000273387 |  | -1.487 | 0.405 | -3.671 | 0.0002 | 0.0012 |
| ENSG00000169682 | SPNS1 | -1.487 | 0.522 | -2.849 | 0.0044 | 0.0142 |
| ENSG00000134107 | BHLHE40 | -1.486 | 0.228 | -6.527 | 0 | 0 |
| ENSG00000107140 | TESK1 | -1.486 | 0.289 | -5.138 | 0 | 0 |
| ENSG00000228594 | FNDC10 | -1.486 | 0.376 | -3.947 | 0.0001 | 0.0005 |
| ENSG00000129636 | ITFG1 | -1.485 | 0.287 | -5.174 | 0 | 0 |
| ENSG00000100302 | RASD2 | -1.485 | 0.447 | -3.323 | 0.0009 | 0.0037 |
| ENSG00000101353 | MROH8 | -1.485 | 0.48 | -3.093 | 0.002 | 0.0073 |
| ENSG00000134574 | DDB2 | -1.484 | 0.232 | -6.384 | 0 | 0 |
| ENSG00000275693 |  | -1.484 | 0.532 | -2.789 | 0.0053 | 0.0167 |
| ENSG00000075624 | ACTB | -1.483 | 0.248 | -5.98 | 0 | 0 |
| ENSG00000260343 | LINC01043 | -1.483 | 0.52 | -2.853 | 0.0043 | 0.0141 |
| ENSG00000009780 | FAM76A | -1.481 | 0.326 | -4.544 | 0 | 0 |
| ENSG00000109854 | HTATIP2 | -1.481 | 0.287 | -5.163 | 0 | 0 |
| ENSG00000176401 | EID2B | -1.481 | 0.341 | -4.339 | 0 | 0.0001 |
| ENSG00000259062 | ACTN1-AS1 | -1.481 | 0.471 | -3.143 | 0.0017 | 0.0063 |
| ENSG00000121552 | CSTA | -1.481 | 0.582 | -2.546 | 0.0109 | 0.0303 |
| ENSG00000188396 | TCTEX1D4 | -1.479 | 0.481 | -3.078 | 0.0021 | 0.0076 |
| ENSG00000005249 | PRKAR2B | -1.478 | 0.376 | -3.926 | 0.0001 | 0.0005 |
| ENSG00000164087 | POC1A | -1.477 | 0.289 | -5.108 | 0 | 0 |
| ENSG00000124216 | SNAI1 | -1.476 | 0.298 | -4.959 | 0 | 0 |
| ENSG00000236671 |  | -1.475 | 0.605 | -2.437 | 0.0148 | 0.039 |
| ENSG00000060642 | PIGV | -1.474 | 0.271 | -5.444 | 0 | 0 |
| ENSG00000177731 | FLII | -1.474 | 0.228 | -6.476 | 0 | 0 |
| ENSG00000177370 | TIMM22 | -1.474 | 0.224 | -6.583 | 0 | 0 |
| ENSG00000129515 | SNX6 | -1.473 | 0.237 | -6.228 | 0 | 0 |
| ENSG00000198788 | MUC2 | -1.473 | 0.456 | -3.233 | 0.0012 | 0.0049 |
| ENSG00000115163 | CENPA | -1.472 | 0.34 | -4.327 | 0 | 0.0001 |
| ENSG00000258920 | FOXN3-AS1 | -1.472 | 0.332 | -4.432 | 0 | 0.0001 |
| ENSG00000179761 | PIPOX | -1.471 | 0.536 | -2.744 | 0.0061 | 0.0187 |
| ENSG00000140905 | GCSH | -1.471 | 0.571 | -2.573 | 0.0101 | 0.0284 |
| ENSG00000213753 | CENPBD1P1 | -1.47 | 0.237 | -6.213 | 0 | 0 |
| ENSG00000254860 | TMEM9B-AS1 | -1.47 | 0.6 | -2.449 | 0.0143 | 0.0379 |
| ENSG00000069812 | HES2 | -1.469 | 0.6 | -2.448 | 0.0144 | 0.038 |
| ENSG00000156482 | RPL30 | -1.468 | 0.241 | -6.086 | 0 | 0 |
| ENSG00000196810 |  | -1.468 | 0.259 | -5.677 | 0 | 0 |
| ENSG00000188185 |  | -1.468 | 0.38 | -3.861 | 0.0001 | 0.0006 |
| ENSG00000180182 | MED14 | -1.467 | 0.305 | -4.815 | 0 | 0 |
| ENSG00000186480 | INSIG1 | -1.465 | 0.221 | -6.628 | 0 | 0 |
| ENSG00000169429 | CXCL8 | -1.465 | 0.323 | -4.535 | 0 | 0 |
| ENSG00000134901 | POGLUT2 | -1.464 | 0.29 | -5.057 | 0 | 0 |
| ENSG00000124257 | NEURL2 | -1.463 | 0.564 | -2.595 | 0.0095 | 0.0269 |
| ENSG00000197238 | H4C11 | -1.462 | 0.432 | -3.383 | 0.0007 | 0.0031 |
| ENSG00000104472 | CHRAC1 | -1.461 | 0.24 | -6.095 | 0 | 0 |
| ENSG00000068650 | ATP11A | -1.461 | 0.274 | -5.33 | 0 | 0 |
| ENSG00000079257 | LXN | -1.461 | 0.336 | -4.347 | 0 | 0.0001 |
| ENSG00000227512 |  | -1.461 | 0.583 | -2.504 | 0.0123 | 0.0334 |
| ENSG00000235499 |  | -1.461 | 0.622 | -2.348 | 0.0189 | 0.0476 |
| ENSG00000163558 | PRKCI | -1.46 | 0.247 | -5.901 | 0 | 0 |
| ENSG00000167699 | GLOD4 | -1.46 | 0.296 | -4.936 | 0 | 0 |
| ENSG00000162894 | FCMR | -1.46 | 0.567 | -2.573 | 0.0101 | 0.0284 |
| ENSG00000279691 |  | -1.46 | 0.614 | -2.376 | 0.0175 | 0.0448 |
| ENSG00000143753 | DEGS1 | -1.459 | 0.248 | -5.874 | 0 | 0 |
| ENSG00000261334 |  | -1.457 | 0.526 | -2.769 | 0.0056 | 0.0176 |
| ENSG00000214553 | LRRC37A11P | -1.457 | 0.566 | -2.577 | 0.01 | 0.0282 |
| ENSG00000135517 | MIP | -1.456 | 0.438 | -3.325 | 0.0009 | 0.0037 |
| ENSG00000237054 | PRMT5-AS1 | -1.455 | 0.442 | -3.292 | 0.001 | 0.0041 |
| ENSG00000275552 |  | -1.455 | 0.561 | -2.593 | 0.0095 | 0.027 |
| ENSG00000111801 | BTN3A3 | -1.455 | 0.601 | -2.424 | 0.0154 | 0.0402 |
| ENSG00000178927 | CYBC1 | -1.454 | 0.264 | -5.506 | 0 | 0 |
| ENSG00000271780 |  | -1.453 | 0.277 | -5.241 | 0 | 0 |
| ENSG00000169604 | ANTXR1 | -1.452 | 0.283 | -5.129 | 0 | 0 |
| ENSG00000172046 | USP19 | -1.452 | 0.271 | -5.354 | 0 | 0 |
| ENSG00000099365 | STX1B | -1.452 | 0.338 | -4.293 | 0 | 0.0001 |
| ENSG00000214892 | USP8P1 | -1.452 | 0.475 | -3.059 | 0.0022 | 0.008 |
| ENSG00000117519 | CNN3 | -1.451 | 0.228 | -6.351 | 0 | 0 |
| ENSG00000167565 | SERTAD3 | -1.45 | 0.275 | -5.276 | 0 | 0 |
| ENSG00000075651 | PLD1 | -1.45 | 0.36 | -4.024 | 0.0001 | 0.0003 |
| ENSG00000165731 | RET | -1.45 | 0.453 | -3.202 | 0.0014 | 0.0053 |
| ENSG00000178567 | EPM2AIP1 | -1.449 | 0.269 | -5.388 | 0 | 0 |
| ENSG00000279030 |  | -1.448 | 0.433 | -3.341 | 0.0008 | 0.0035 |
| ENSG00000183801 | OLFML1 | -1.447 | 0.47 | -3.081 | 0.0021 | 0.0075 |
| ENSG00000179604 | CDC42EP4 | -1.445 | 0.295 | -4.896 | 0 | 0 |
| ENSG00000119922 | IFIT2 | -1.444 | 0.215 | -6.72 | 0 | 0 |
| ENSG00000184786 | DYNLT2 | -1.442 | 0.249 | -5.797 | 0 | 0 |
| ENSG00000107819 | SFXN3 | -1.442 | 0.258 | -5.584 | 0 | 0 |
| ENSG00000196878 | LAMB3 | -1.442 | 0.377 | -3.823 | 0.0001 | 0.0007 |
| ENSG00000273264 |  | -1.441 | 0.531 | -2.712 | 0.0067 | 0.0203 |
| ENSG00000112234 | FBXL4 | -1.44 | 0.323 | -4.456 | 0 | 0.0001 |
| ENSG00000260278 |  | -1.439 | 0.319 | -4.509 | 0 | 0.0001 |
| ENSG00000231971 | CT69 | -1.438 | 0.4 | -3.59 | 0.0003 | 0.0016 |
| ENSG00000271851 |  | -1.438 | 0.618 | -2.329 | 0.0199 | 0.0497 |
| ENSG00000182197 | EXT1 | -1.437 | 0.211 | -6.808 | 0 | 0 |
| ENSG00000246731 | MGC16275 | -1.436 | 0.371 | -3.873 | 0.0001 | 0.0006 |
| ENSG00000261430 |  | -1.435 | 0.549 | -2.613 | 0.009 | 0.0258 |
| ENSG00000254671 |  | -1.435 | 0.568 | -2.529 | 0.0114 | 0.0315 |
| ENSG00000145817 | YIPF5 | -1.434 | 0.285 | -5.038 | 0 | 0 |
| ENSG00000140443 | IGF1R | -1.434 | 0.235 | -6.109 | 0 | 0 |
| ENSG00000013392 | RWDD2A | -1.434 | 0.336 | -4.264 | 0 | 0.0001 |
| ENSG00000278376 |  | -1.434 | 0.44 | -3.261 | 0.0011 | 0.0045 |
| ENSG00000131746 | TNS4 | -1.434 | 0.48 | -2.99 | 0.0028 | 0.0097 |
| ENSG00000169105 | CHST14 | -1.433 | 0.256 | -5.589 | 0 | 0 |
| ENSG00000230454 |  | -1.433 | 0.526 | -2.723 | 0.0065 | 0.0198 |
| ENSG00000172725 | CORO1B | -1.432 | 0.209 | -6.835 | 0 | 0 |
| ENSG00000146701 | MDH2 | -1.432 | 0.227 | -6.301 | 0 | 0 |
| ENSG00000107738 | VSIR | -1.431 | 0.334 | -4.287 | 0 | 0.0001 |
| ENSG00000255224 |  | -1.43 | 0.285 | -5.022 | 0 | 0 |
| ENSG00000197780 | TAF13 | -1.43 | 0.22 | -6.506 | 0 | 0 |
| ENSG00000240225 | ZNF542P | -1.43 | 0.271 | -5.271 | 0 | 0 |
| ENSG00000105426 | PTPRS | -1.43 | 0.335 | -4.263 | 0 | 0.0001 |
| ENSG00000100628 | ASB2 | -1.43 | 0.431 | -3.32 | 0.0009 | 0.0037 |
| ENSG00000275714 | H3C1 | -1.429 | 0.276 | -5.181 | 0 | 0 |
| ENSG00000259083 |  | -1.429 | 0.555 | -2.572 | 0.0101 | 0.0284 |
| ENSG00000113448 | PDE4D | -1.428 | 0.313 | -4.559 | 0 | 0 |
| ENSG00000174705 | SH3PXD2B | -1.428 | 0.287 | -4.976 | 0 | 0 |
| ENSG00000116833 | NR5A2 | -1.428 | 0.422 | -3.385 | 0.0007 | 0.0031 |
| ENSG00000106392 | C1GALT1 | -1.426 | 0.261 | -5.471 | 0 | 0 |
| ENSG00000162433 | AK4 | -1.426 | 0.435 | -3.278 | 0.001 | 0.0042 |
| ENSG00000071909 | MYO3B | -1.426 | 0.5 | -2.854 | 0.0043 | 0.0141 |
| ENSG00000037280 | FLT4 | -1.426 | 0.541 | -2.637 | 0.0084 | 0.0243 |
| ENSG00000136816 | TOR1B | -1.424 | 0.249 | -5.719 | 0 | 0 |
| ENSG00000107968 | MAP3K8 | -1.423 | 0.304 | -4.686 | 0 | 0 |
| ENSG00000101188 | NTSR1 | -1.423 | 0.336 | -4.232 | 0 | 0.0002 |
| ENSG00000142920 | AZIN2 | -1.423 | 0.449 | -3.171 | 0.0015 | 0.0058 |
| ENSG00000224116 | INHBA-AS1 | -1.423 | 0.5 | -2.849 | 0.0044 | 0.0142 |
| ENSG00000257815 | PRANCR | -1.422 | 0.312 | -4.564 | 0 | 0 |
| ENSG00000139641 | ESYT1 | -1.422 | 0.239 | -5.949 | 0 | 0 |
| ENSG00000138796 | HADH | -1.421 | 0.28 | -5.066 | 0 | 0 |
| ENSG00000173638 | SLC19A1 | -1.421 | 0.354 | -4.011 | 0.0001 | 0.0004 |
| ENSG00000231453 | LINC01305 | -1.421 | 0.521 | -2.731 | 0.0063 | 0.0194 |
| ENSG00000198642 | KLHL9 | -1.42 | 0.27 | -5.26 | 0 | 0 |
| ENSG00000181788 | SIAH2 | -1.42 | 0.263 | -5.402 | 0 | 0 |
| ENSG00000156968 | MPV17L | -1.419 | 0.585 | -2.426 | 0.0153 | 0.04 |
| ENSG00000196776 | CD47 | -1.416 | 0.342 | -4.145 | 0 | 0.0002 |
| ENSG00000196890 | H2BU1 | -1.415 | 0.36 | -3.936 | 0.0001 | 0.0005 |
| ENSG00000257298 |  | -1.415 | 0.368 | -3.849 | 0.0001 | 0.0007 |
| ENSG00000181588 | MEX3D | -1.414 | 0.257 | -5.508 | 0 | 0 |
| ENSG00000099866 | MADCAM1 | -1.414 | 0.449 | -3.148 | 0.0016 | 0.0062 |
| ENSG00000187193 | MT1X | -1.413 | 0.325 | -4.353 | 0 | 0.0001 |
| ENSG00000147799 | ARHGAP39 | -1.412 | 0.378 | -3.736 | 0.0002 | 0.001 |
| ENSG00000171792 | RHNO1 | -1.411 | 0.238 | -5.924 | 0 | 0 |
| ENSG00000128564 | VGF | -1.411 | 0.412 | -3.426 | 0.0006 | 0.0027 |
| ENSG00000140093 | SERPINA10 | -1.411 | 0.514 | -2.742 | 0.0061 | 0.0188 |
| ENSG00000197702 | PARVA | -1.41 | 0.29 | -4.87 | 0 | 0 |
| ENSG00000200312 |  | -1.41 | 0.398 | -3.544 | 0.0004 | 0.0018 |
| ENSG00000065357 | DGKA | -1.409 | 0.242 | -5.814 | 0 | 0 |
| ENSG00000186666 | BCDIN3D | -1.409 | 0.327 | -4.308 | 0 | 0.0001 |
| ENSG00000187840 | EIF4EBP1 | -1.408 | 0.278 | -5.063 | 0 | 0 |
| ENSG00000025708 | TYMP | -1.408 | 0.359 | -3.919 | 0.0001 | 0.0005 |
| ENSG00000222365 | SNORD12B | -1.408 | 0.447 | -3.149 | 0.0016 | 0.0062 |
| ENSG00000257863 |  | -1.408 | 0.494 | -2.851 | 0.0044 | 0.0142 |
| ENSG00000167930 | FAM234A | -1.406 | 0.273 | -5.149 | 0 | 0 |
| ENSG00000150459 | SAP18 | -1.406 | 0.204 | -6.893 | 0 | 0 |
| ENSG00000241973 | PI4KA | -1.406 | 0.249 | -5.655 | 0 | 0 |
| ENSG00000172818 | OVOL1 | -1.406 | 0.311 | -4.522 | 0 | 0 |
| ENSG00000260806 |  | -1.406 | 0.342 | -4.116 | 0 | 0.0002 |
| ENSG00000114737 | CISH | -1.406 | 0.532 | -2.643 | 0.0082 | 0.024 |
| ENSG00000018280 | SLC11A1 | -1.405 | 0.492 | -2.858 | 0.0043 | 0.0139 |
| ENSG00000240053 | LY6G5B | -1.405 | 0.575 | -2.445 | 0.0145 | 0.0383 |
| ENSG00000163041 | H3-3A | -1.404 | 0.334 | -4.204 | 0 | 0.0002 |
| ENSG00000160401 | CFAP157 | -1.404 | 0.454 | -3.09 | 0.002 | 0.0073 |
| ENSG00000273062 |  | -1.402 | 0.52 | -2.698 | 0.007 | 0.021 |
| ENSG00000145248 | SLC10A4 | -1.402 | 0.539 | -2.602 | 0.0093 | 0.0265 |
| ENSG00000235652 | FBXO30-DT | -1.401 | 0.519 | -2.699 | 0.007 | 0.021 |
| ENSG00000088986 | DYNLL1 | -1.4 | 0.218 | -6.432 | 0 | 0 |
| ENSG00000142002 | DPP9 | -1.399 | 0.221 | -6.333 | 0 | 0 |
| ENSG00000235374 | SSR4P1 | -1.399 | 0.4 | -3.501 | 0.0005 | 0.0021 |
| ENSG00000255568 | BRWD1-AS2 | -1.399 | 0.421 | -3.321 | 0.0009 | 0.0037 |
| ENSG00000177606 | JUN | -1.398 | 0.209 | -6.686 | 0 | 0 |
| ENSG00000138172 | CALHM2 | -1.397 | 0.285 | -4.908 | 0 | 0 |
| ENSG00000131153 | GINS2 | -1.397 | 0.279 | -5.012 | 0 | 0 |
| ENSG00000170852 | KBTBD2 | -1.397 | 0.283 | -4.947 | 0 | 0 |
| ENSG00000161381 | PLXDC1 | -1.397 | 0.425 | -3.287 | 0.001 | 0.0041 |
| ENSG00000173894 | CBX2 | -1.397 | 0.48 | -2.91 | 0.0036 | 0.0121 |
| ENSG00000243679 |  | -1.397 | 0.598 | -2.335 | 0.0195 | 0.049 |
| ENSG00000280152 |  | -1.396 | 0.39 | -3.58 | 0.0003 | 0.0016 |
| ENSG00000108639 | SYNGR2 | -1.395 | 0.251 | -5.566 | 0 | 0 |
| ENSG00000157111 | TMEM171 | -1.395 | 0.307 | -4.544 | 0 | 0 |
| ENSG00000100422 | CERK | -1.395 | 0.272 | -5.123 | 0 | 0 |
| ENSG00000125648 | SLC25A23 | -1.395 | 0.299 | -4.667 | 0 | 0 |
| ENSG00000253227 |  | -1.395 | 0.555 | -2.514 | 0.0119 | 0.0326 |
| ENSG00000184381 | PLA2G6 | -1.394 | 0.381 | -3.656 | 0.0003 | 0.0013 |
| ENSG00000113732 | ATP6V0E1 | -1.393 | 0.21 | -6.626 | 0 | 0 |
| ENSG00000163347 | CLDN1 | -1.393 | 0.457 | -3.051 | 0.0023 | 0.0082 |
| ENSG00000157601 | MX1 | -1.392 | 0.273 | -5.102 | 0 | 0 |
| ENSG00000127419 | TMEM175 | -1.392 | 0.308 | -4.526 | 0 | 0 |
| ENSG00000144677 | CTDSPL | -1.392 | 0.29 | -4.805 | 0 | 0 |
| ENSG00000266074 | BAHCC1 | -1.392 | 0.31 | -4.488 | 0 | 0.0001 |
| ENSG00000176463 | SLCO3A1 | -1.392 | 0.408 | -3.411 | 0.0006 | 0.0028 |
| ENSG00000167281 | RBFOX3 | -1.392 | 0.574 | -2.426 | 0.0153 | 0.04 |
| ENSG00000144021 | CIAO1 | -1.39 | 0.228 | -6.087 | 0 | 0 |
| ENSG00000168890 | TMEM150A | -1.39 | 0.322 | -4.318 | 0 | 0.0001 |
| ENSG00000185862 | EVI2B | -1.39 | 0.348 | -3.996 | 0.0001 | 0.0004 |
| ENSG00000243477 | NAA80 | -1.39 | 0.425 | -3.267 | 0.0011 | 0.0044 |
| ENSG00000054356 | PTPRN | -1.389 | 0.501 | -2.772 | 0.0056 | 0.0175 |
| ENSG00000179403 | VWA1 | -1.388 | 0.421 | -3.296 | 0.001 | 0.004 |
| ENSG00000253327 | RAD21-AS1 | -1.388 | 0.559 | -2.483 | 0.013 | 0.035 |
| ENSG00000027075 | PRKCH | -1.385 | 0.467 | -2.968 | 0.003 | 0.0103 |
| ENSG00000125912 | NCLN | -1.384 | 0.242 | -5.726 | 0 | 0 |
| ENSG00000123989 | CHPF | -1.384 | 0.222 | -6.222 | 0 | 0 |
| ENSG00000213514 |  | -1.384 | 0.486 | -2.849 | 0.0044 | 0.0142 |
| ENSG00000057252 | SOAT1 | -1.383 | 0.222 | -6.238 | 0 | 0 |
| ENSG00000245694 | CRNDE | -1.383 | 0.319 | -4.335 | 0 | 0.0001 |
| ENSG00000119523 | ALG2 | -1.382 | 0.206 | -6.696 | 0 | 0 |
| ENSG00000145354 | CISD2 | -1.382 | 0.267 | -5.174 | 0 | 0 |
| ENSG00000123096 | SSPN | -1.382 | 0.415 | -3.328 | 0.0009 | 0.0036 |
| ENSG00000187642 | PERM1 | -1.382 | 0.433 | -3.194 | 0.0014 | 0.0054 |
| ENSG00000173928 | SWSAP1 | -1.382 | 0.58 | -2.384 | 0.0171 | 0.0439 |
| ENSG00000167900 | TK1 | -1.38 | 0.221 | -6.236 | 0 | 0 |
| ENSG00000160606 | TLCD1 | -1.38 | 0.347 | -3.98 | 0.0001 | 0.0004 |
| ENSG00000168282 | MGAT2 | -1.379 | 0.333 | -4.143 | 0 | 0.0002 |
| ENSG00000168952 | STXBP6 | -1.379 | 0.438 | -3.145 | 0.0017 | 0.0062 |
| ENSG00000167470 | MIDN | -1.378 | 0.262 | -5.259 | 0 | 0 |
| ENSG00000277895 |  | -1.378 | 0.454 | -3.035 | 0.0024 | 0.0085 |
| ENSG00000273061 | CDC37L1-DT | -1.378 | 0.537 | -2.565 | 0.0103 | 0.0289 |
| ENSG00000089053 | ANAPC5 | -1.376 | 0.26 | -5.29 | 0 | 0 |
| ENSG00000247271 | ZBED5-AS1 | -1.376 | 0.353 | -3.9 | 0.0001 | 0.0005 |
| ENSG00000108784 | NAGLU | -1.375 | 0.258 | -5.333 | 0 | 0 |
| ENSG00000140931 | CMTM3 | -1.375 | 0.269 | -5.118 | 0 | 0 |
| ENSG00000198682 | PAPSS2 | -1.375 | 0.254 | -5.405 | 0 | 0 |
| ENSG00000241360 | PDXP | -1.375 | 0.471 | -2.92 | 0.0035 | 0.0118 |
| ENSG00000132541 | RIDA | -1.373 | 0.292 | -4.707 | 0 | 0 |
| ENSG00000143797 | MBOAT2 | -1.372 | 0.305 | -4.499 | 0 | 0.0001 |
| ENSG00000171132 | PRKCE | -1.371 | 0.319 | -4.304 | 0 | 0.0001 |
| ENSG00000164849 | GPR146 | -1.371 | 0.475 | -2.885 | 0.0039 | 0.0129 |
| ENSG00000203930 | LINC00632 | -1.371 | 0.533 | -2.572 | 0.0101 | 0.0284 |
| ENSG00000258947 | TUBB3 | -1.37 | 0.307 | -4.457 | 0 | 0.0001 |
| ENSG00000279631 |  | -1.37 | 0.41 | -3.338 | 0.0008 | 0.0035 |
| ENSG00000173531 | MST1 | -1.369 | 0.375 | -3.647 | 0.0003 | 0.0013 |
| ENSG00000115257 | PCSK4 | -1.369 | 0.468 | -2.926 | 0.0034 | 0.0116 |
| ENSG00000226696 | LENG8-AS1 | -1.369 | 0.51 | -2.685 | 0.0073 | 0.0217 |
| ENSG00000118508 | RAB32 | -1.368 | 0.245 | -5.574 | 0 | 0 |
| ENSG00000198517 | MAFK | -1.368 | 0.242 | -5.643 | 0 | 0 |
| ENSG00000100426 | ZBED4 | -1.368 | 0.335 | -4.086 | 0 | 0.0003 |
| ENSG00000137193 | PIM1 | -1.367 | 0.308 | -4.446 | 0 | 0.0001 |
| ENSG00000231256 | CFAP97D1 | -1.367 | 0.51 | -2.68 | 0.0074 | 0.022 |
| ENSG00000123146 | ADGRE5 | -1.366 | 0.263 | -5.186 | 0 | 0 |
| ENSG00000101421 | CHMP4B | -1.366 | 0.246 | -5.549 | 0 | 0 |
| ENSG00000187091 | PLCD1 | -1.366 | 0.273 | -4.998 | 0 | 0 |
| ENSG00000188825 | LINC00910 | -1.366 | 0.429 | -3.184 | 0.0015 | 0.0056 |
| ENSG00000105613 | MAST1 | -1.366 | 0.453 | -3.013 | 0.0026 | 0.0091 |
| ENSG00000177697 | CD151 | -1.365 | 0.215 | -6.351 | 0 | 0 |
| ENSG00000147586 | MRPS28 | -1.365 | 0.5 | -2.731 | 0.0063 | 0.0194 |
| ENSG00000115145 | STAM2 | -1.363 | 0.266 | -5.114 | 0 | 0 |
| ENSG00000277715 |  | -1.363 | 0.543 | -2.511 | 0.012 | 0.0328 |
| ENSG00000164733 | CTSB | -1.361 | 0.242 | -5.621 | 0 | 0 |
| ENSG00000104626 | ERI1 | -1.36 | 0.287 | -4.74 | 0 | 0 |
| ENSG00000198034 | RPS4X | -1.36 | 0.24 | -5.667 | 0 | 0 |
| ENSG00000161558 | TMEM143 | -1.359 | 0.464 | -2.928 | 0.0034 | 0.0115 |
| ENSG00000267288 | LOC105371795 | -1.359 | 0.543 | -2.5 | 0.0124 | 0.0337 |
| ENSG00000117595 | IRF6 | -1.359 | 0.57 | -2.385 | 0.0171 | 0.0438 |
| ENSG00000118526 | TCF21 | -1.358 | 0.56 | -2.423 | 0.0154 | 0.0403 |
| ENSG00000105650 | PDE4C | -1.357 | 0.295 | -4.604 | 0 | 0 |
| ENSG00000040275 | SPDL1 | -1.357 | 0.263 | -5.162 | 0 | 0 |
| ENSG00000145214 | DGKQ | -1.357 | 0.289 | -4.7 | 0 | 0 |
| ENSG00000164466 | SFXN1 | -1.355 | 0.3 | -4.52 | 0 | 0 |
| ENSG00000203722 | RAET1G | -1.355 | 0.508 | -2.667 | 0.0076 | 0.0227 |
| ENSG00000105355 | PLIN3 | -1.354 | 0.219 | -6.194 | 0 | 0 |
| ENSG00000167065 | DUSP18 | -1.354 | 0.506 | -2.673 | 0.0075 | 0.0223 |
| ENSG00000163017 | ACTG2 | -1.353 | 0.26 | -5.205 | 0 | 0 |
| ENSG00000165312 | OTUD1 | -1.352 | 0.313 | -4.317 | 0 | 0.0001 |
| ENSG00000215712 | TMEM242 | -1.352 | 0.308 | -4.398 | 0 | 0.0001 |
| ENSG00000279692 |  | -1.352 | 0.488 | -2.773 | 0.0056 | 0.0174 |
| ENSG00000183486 | MX2 | -1.35 | 0.279 | -4.839 | 0 | 0 |
| ENSG00000173456 | RNF26 | -1.35 | 0.244 | -5.528 | 0 | 0 |
| ENSG00000271811 |  | -1.35 | 0.459 | -2.942 | 0.0033 | 0.0111 |
| ENSG00000215375 | MYL5 | -1.349 | 0.299 | -4.508 | 0 | 0.0001 |
| ENSG00000162576 | MXRA8 | -1.348 | 0.245 | -5.506 | 0 | 0 |
| ENSG00000087302 | RTRAF | -1.348 | 0.266 | -5.075 | 0 | 0 |
| ENSG00000179218 | CALR | -1.348 | 0.192 | -7.019 | 0 | 0 |
| ENSG00000116670 | MAD2L2 | -1.347 | 0.241 | -5.592 | 0 | 0 |
| ENSG00000188352 | FOCAD | -1.347 | 0.249 | -5.419 | 0 | 0 |
| ENSG00000114738 | MAPKAPK3 | -1.347 | 0.241 | -5.587 | 0 | 0 |
| ENSG00000133216 | EPHB2 | -1.347 | 0.407 | -3.311 | 0.0009 | 0.0038 |
| ENSG00000089597 | GANAB | -1.346 | 0.271 | -4.971 | 0 | 0 |
| ENSG00000168078 | PBK | -1.346 | 0.345 | -3.898 | 0.0001 | 0.0006 |
| ENSG00000111885 | MAN1A1 | -1.346 | 0.355 | -3.796 | 0.0001 | 0.0008 |
| ENSG00000070756 | PABPC1 | -1.344 | 0.257 | -5.237 | 0 | 0 |
| ENSG00000147883 | CDKN2B | -1.344 | 0.255 | -5.272 | 0 | 0 |
| ENSG00000255121 |  | -1.344 | 0.498 | -2.698 | 0.007 | 0.021 |
| ENSG00000196284 | SUPT3H | -1.344 | 0.541 | -2.485 | 0.013 | 0.0349 |
| ENSG00000179454 | KLHL28 | -1.343 | 0.288 | -4.663 | 0 | 0 |
| ENSG00000106723 | SPIN1 | -1.343 | 0.24 | -5.591 | 0 | 0 |
| ENSG00000151729 | SLC25A4 | -1.343 | 0.312 | -4.303 | 0 | 0.0001 |
| ENSG00000254254 | LOC101929415 | -1.343 | 0.441 | -3.047 | 0.0023 | 0.0082 |
| ENSG00000281357 | ARRDC3-AS1 | -1.343 | 0.477 | -2.817 | 0.0049 | 0.0155 |
| ENSG00000149609 | C20orf144 | -1.343 | 0.563 | -2.385 | 0.0171 | 0.0439 |
| ENSG00000159593 | NAE1 | -1.342 | 0.245 | -5.472 | 0 | 0 |
| ENSG00000118515 | SGK1 | -1.342 | 0.203 | -6.608 | 0 | 0 |
| ENSG00000143324 | XPR1 | -1.342 | 0.261 | -5.148 | 0 | 0 |
| ENSG00000140464 | PML | -1.342 | 0.261 | -5.143 | 0 | 0 |
| ENSG00000246067 |  | -1.342 | 0.382 | -3.509 | 0.0005 | 0.0021 |
| ENSG00000275807 |  | -1.342 | 0.527 | -2.548 | 0.0108 | 0.0301 |
| ENSG00000150938 | CRIM1 | -1.341 | 0.194 | -6.927 | 0 | 0 |
| ENSG00000206535 | LNP1 | -1.34 | 0.364 | -3.682 | 0.0002 | 0.0012 |
| ENSG00000228393 |  | -1.339 | 0.269 | -4.974 | 0 | 0 |
| ENSG00000184988 | TMEM106A | -1.339 | 0.336 | -3.99 | 0.0001 | 0.0004 |
| ENSG00000138378 | STAT4 | -1.339 | 0.447 | -2.997 | 0.0027 | 0.0095 |
| ENSG00000102804 | TSC22D1 | -1.338 | 0.207 | -6.465 | 0 | 0 |
| ENSG00000272556 |  | -1.338 | 0.504 | -2.653 | 0.008 | 0.0235 |
| ENSG00000279331 | RBM12B-AS1 | -1.338 | 0.525 | -2.549 | 0.0108 | 0.03 |
| ENSG00000125945 | ZNF436 | -1.337 | 0.24 | -5.571 | 0 | 0 |
| ENSG00000260001 | TGFBR3L | -1.337 | 0.561 | -2.383 | 0.0172 | 0.0441 |
| ENSG00000120539 | MASTL | -1.336 | 0.265 | -5.038 | 0 | 0 |
| ENSG00000153214 | TMEM87B | -1.336 | 0.274 | -4.878 | 0 | 0 |
| ENSG00000137720 | C11orf1 | -1.336 | 0.265 | -5.046 | 0 | 0 |
| ENSG00000173338 | KCNK7 | -1.336 | 0.376 | -3.551 | 0.0004 | 0.0018 |
| ENSG00000223509 |  | -1.336 | 0.547 | -2.444 | 0.0145 | 0.0384 |
| ENSG00000164627 | KIF6 | -1.335 | 0.504 | -2.65 | 0.008 | 0.0236 |
| ENSG00000265112 | MIR3153 | -1.335 | 0.505 | -2.644 | 0.0082 | 0.0239 |
| ENSG00000169242 | EFNA1 | -1.333 | 0.569 | -2.342 | 0.0192 | 0.0482 |
| ENSG00000158406 | H4C8 | -1.332 | 0.218 | -6.099 | 0 | 0 |
| ENSG00000104613 | INTS10 | -1.332 | 0.253 | -5.267 | 0 | 0 |
| ENSG00000109911 | ELP4 | -1.332 | 0.445 | -2.991 | 0.0028 | 0.0097 |
| ENSG00000254639 |  | -1.332 | 0.509 | -2.617 | 0.0089 | 0.0256 |
| ENSG00000066117 | SMARCD1 | -1.331 | 0.243 | -5.473 | 0 | 0 |
| ENSG00000141985 | SH3GL1 | -1.331 | 0.256 | -5.196 | 0 | 0 |
| ENSG00000136699 | SMPD4 | -1.33 | 0.285 | -4.664 | 0 | 0 |
| ENSG00000163686 | ABHD6 | -1.329 | 0.396 | -3.355 | 0.0008 | 0.0033 |
| ENSG00000103043 | VAC14 | -1.328 | 0.265 | -5.02 | 0 | 0 |
| ENSG00000272933 |  | -1.328 | 0.33 | -4.026 | 0.0001 | 0.0003 |
| ENSG00000172663 | TMEM134 | -1.328 | 0.332 | -3.997 | 0.0001 | 0.0004 |
| ENSG00000182087 | TMEM259 | -1.327 | 0.219 | -6.058 | 0 | 0 |
| ENSG00000123472 | ATPAF1 | -1.326 | 0.252 | -5.268 | 0 | 0 |
| ENSG00000175048 | ZDHHC14 | -1.326 | 0.373 | -3.553 | 0.0004 | 0.0018 |
| ENSG00000266680 |  | -1.325 | 0.27 | -4.903 | 0 | 0 |
| ENSG00000167695 | TLCD3A | -1.325 | 0.209 | -6.337 | 0 | 0 |
| ENSG00000260000 |  | -1.325 | 0.391 | -3.391 | 0.0007 | 0.003 |
| ENSG00000257557 |  | -1.325 | 0.526 | -2.519 | 0.0118 | 0.0322 |
| ENSG00000151929 | BAG3 | -1.324 | 0.213 | -6.214 | 0 | 0 |
| ENSG00000197324 | LRP10 | -1.324 | 0.211 | -6.28 | 0 | 0 |
| ENSG00000175513 | TSGA10IP | -1.324 | 0.337 | -3.935 | 0.0001 | 0.0005 |
| ENSG00000157557 | ETS2 | -1.323 | 0.234 | -5.657 | 0 | 0 |
| ENSG00000175482 | POLD4 | -1.322 | 0.315 | -4.192 | 0 | 0.0002 |
| ENSG00000280063 |  | -1.322 | 0.544 | -2.43 | 0.0151 | 0.0396 |
| ENSG00000155090 | KLF10 | -1.321 | 0.29 | -4.562 | 0 | 0 |
| ENSG00000083457 | ITGAE | -1.321 | 0.251 | -5.256 | 0 | 0 |
| ENSG00000205336 | ADGRG1 | -1.321 | 0.355 | -3.719 | 0.0002 | 0.001 |
| ENSG00000177706 | FAM20C | -1.32 | 0.251 | -5.256 | 0 | 0 |
| ENSG00000109738 | GLRB | -1.32 | 0.338 | -3.909 | 0.0001 | 0.0005 |
| ENSG00000263884 |  | -1.32 | 0.417 | -3.165 | 0.0016 | 0.0059 |
| ENSG00000238279 |  | -1.32 | 0.425 | -3.106 | 0.0019 | 0.007 |
| ENSG00000189339 | SLC35E2B | -1.319 | 0.27 | -4.879 | 0 | 0 |
| ENSG00000272168 |  | -1.319 | 0.347 | -3.802 | 0.0001 | 0.0008 |
| ENSG00000266998 |  | -1.319 | 0.508 | -2.599 | 0.0093 | 0.0267 |
| ENSG00000272913 |  | -1.318 | 0.308 | -4.281 | 0 | 0.0001 |
| ENSG00000163376 | KBTBD8 | -1.318 | 0.293 | -4.504 | 0 | 0.0001 |
| ENSG00000260077 |  | -1.318 | 0.529 | -2.494 | 0.0126 | 0.0342 |
| ENSG00000132912 | DCTN4 | -1.317 | 0.262 | -5.025 | 0 | 0 |
| ENSG00000154839 | SKA1 | -1.316 | 0.277 | -4.743 | 0 | 0 |
| ENSG00000111863 | ADTRP | -1.316 | 0.362 | -3.639 | 0.0003 | 0.0013 |
| ENSG00000274248 |  | -1.316 | 0.558 | -2.36 | 0.0183 | 0.0463 |
| ENSG00000012963 | UBR7 | -1.315 | 0.263 | -5.003 | 0 | 0 |
| ENSG00000239569 |  | -1.315 | 0.254 | -5.185 | 0 | 0 |
| ENSG00000113658 | SMAD5 | -1.313 | 0.242 | -5.424 | 0 | 0 |
| ENSG00000134056 | MRPS36 | -1.313 | 0.279 | -4.702 | 0 | 0 |
| ENSG00000204316 | MRPL38 | -1.313 | 0.439 | -2.988 | 0.0028 | 0.0097 |
| ENSG00000171155 | C1GALT1C1 | -1.312 | 0.249 | -5.261 | 0 | 0 |
| ENSG00000212978 | LOC339803 | -1.311 | 0.285 | -4.596 | 0 | 0 |
| ENSG00000280721 |  | -1.311 | 0.432 | -3.032 | 0.0024 | 0.0086 |
| ENSG00000104205 | SGK3 | -1.311 | 0.488 | -2.687 | 0.0072 | 0.0216 |
| ENSG00000184185 | KCNJ12 | -1.311 | 0.494 | -2.651 | 0.008 | 0.0236 |
| ENSG00000233452 | STXBP5-AS1 | -1.31 | 0.475 | -2.758 | 0.0058 | 0.0181 |
| ENSG00000233559 |  | -1.309 | 0.256 | -5.106 | 0 | 0 |
| ENSG00000165475 | CRYL1 | -1.308 | 0.39 | -3.351 | 0.0008 | 0.0034 |
| ENSG00000166710 | B2M | -1.307 | 0.211 | -6.2 | 0 | 0 |
| ENSG00000029364 | SLC39A9 | -1.305 | 0.233 | -5.59 | 0 | 0 |
| ENSG00000108960 | MMD | -1.305 | 0.384 | -3.402 | 0.0007 | 0.0029 |
| ENSG00000269246 |  | -1.304 | 0.497 | -2.622 | 0.0087 | 0.0252 |
| ENSG00000106733 | NMRK1 | -1.303 | 0.341 | -3.821 | 0.0001 | 0.0007 |
| ENSG00000139160 | ETFBKMT | -1.303 | 0.472 | -2.762 | 0.0057 | 0.0179 |
| ENSG00000228327 |  | -1.303 | 0.553 | -2.354 | 0.0186 | 0.047 |
| ENSG00000152518 | ZFP36L2 | -1.302 | 0.248 | -5.25 | 0 | 0 |
| ENSG00000168904 | LRRC28 | -1.302 | 0.399 | -3.265 | 0.0011 | 0.0044 |
| ENSG00000261105 | LMO7-AS1 | -1.302 | 0.528 | -2.466 | 0.0137 | 0.0364 |
| ENSG00000140022 | STON2 | -1.301 | 0.338 | -3.854 | 0.0001 | 0.0006 |
| ENSG00000173621 | LRFN4 | -1.3 | 0.231 | -5.629 | 0 | 0 |
| ENSG00000177352 | CCDC71 | -1.3 | 0.315 | -4.133 | 0 | 0.0002 |
| ENSG00000148908 | RGS10 | -1.299 | 0.317 | -4.096 | 0 | 0.0003 |
| ENSG00000136731 | UGGT1 | -1.298 | 0.229 | -5.662 | 0 | 0 |
| ENSG00000143164 | DCAF6 | -1.296 | 0.29 | -4.472 | 0 | 0.0001 |
| ENSG00000068354 | TBC1D25 | -1.296 | 0.351 | -3.69 | 0.0002 | 0.0011 |
| ENSG00000166167 | BTRC | -1.295 | 0.352 | -3.681 | 0.0002 | 0.0012 |
| ENSG00000204103 | MAFB | -1.295 | 0.407 | -3.178 | 0.0015 | 0.0057 |
| ENSG00000250486 | FAM218A | -1.295 | 0.419 | -3.087 | 0.002 | 0.0074 |
| ENSG00000081189 | MEF2C | -1.295 | 0.474 | -2.735 | 0.0062 | 0.0192 |
| ENSG00000186827 | TNFRSF4 | -1.294 | 0.405 | -3.196 | 0.0014 | 0.0054 |
| ENSG00000177954 | RPS27 | -1.293 | 0.194 | -6.672 | 0 | 0 |
| ENSG00000179941 | BBS10 | -1.293 | 0.255 | -5.066 | 0 | 0 |
| ENSG00000157193 | LRP8 | -1.293 | 0.337 | -3.839 | 0.0001 | 0.0007 |
| ENSG00000069849 | ATP1B3 | -1.292 | 0.237 | -5.453 | 0 | 0 |
| ENSG00000125818 | PSMF1 | -1.292 | 0.246 | -5.252 | 0 | 0 |
| ENSG00000173950 | XXYLT1 | -1.292 | 0.341 | -3.793 | 0.0001 | 0.0008 |
| ENSG00000168811 | IL12A | -1.29 | 0.302 | -4.266 | 0 | 0.0001 |
| ENSG00000127528 | KLF2 | -1.289 | 0.402 | -3.204 | 0.0014 | 0.0053 |
| ENSG00000198270 | TMEM116 | -1.288 | 0.259 | -4.969 | 0 | 0 |
| ENSG00000120833 | SOCS2 | -1.288 | 0.24 | -5.367 | 0 | 0 |
| ENSG00000187239 | FNBP1 | -1.288 | 0.304 | -4.236 | 0 | 0.0002 |
| ENSG00000119865 | CNRIP1 | -1.287 | 0.249 | -5.169 | 0 | 0 |
| ENSG00000135318 | NT5E | -1.286 | 0.218 | -5.903 | 0 | 0 |
| ENSG00000241058 | NSUN6 | -1.286 | 0.273 | -4.708 | 0 | 0 |
| ENSG00000118804 | STBD1 | -1.286 | 0.356 | -3.607 | 0.0003 | 0.0015 |
| ENSG00000132394 | EEFSEC | -1.286 | 0.519 | -2.479 | 0.0132 | 0.0353 |
| ENSG00000079246 | XRCC5 | -1.285 | 0.229 | -5.62 | 0 | 0 |
| ENSG00000049540 | ELN | -1.284 | 0.348 | -3.691 | 0.0002 | 0.0011 |
| ENSG00000175390 | EIF3F | -1.283 | 0.211 | -6.092 | 0 | 0 |
| ENSG00000167601 | AXL | -1.283 | 0.206 | -6.213 | 0 | 0 |
| ENSG00000260035 |  | -1.282 | 0.419 | -3.064 | 0.0022 | 0.0079 |
| ENSG00000275441 |  | -1.28 | 0.338 | -3.783 | 0.0002 | 0.0008 |
| ENSG00000197061 | H4C3 | -1.279 | 0.218 | -5.863 | 0 | 0 |
| ENSG00000166086 | JAM3 | -1.278 | 0.233 | -5.49 | 0 | 0 |
| ENSG00000267342 |  | -1.278 | 0.524 | -2.437 | 0.0148 | 0.039 |
| ENSG00000175315 | CST6 | -1.278 | 0.542 | -2.358 | 0.0184 | 0.0466 |
| ENSG00000079156 | OSBPL6 | -1.278 | 0.545 | -2.345 | 0.019 | 0.0478 |
| ENSG00000130821 | SLC6A8 | -1.277 | 0.276 | -4.619 | 0 | 0 |
| ENSG00000174807 | CD248 | -1.277 | 0.272 | -4.695 | 0 | 0 |
| ENSG00000271020 |  | -1.277 | 0.462 | -2.761 | 0.0058 | 0.018 |
| ENSG00000166920 | C15orf48 | -1.276 | 0.301 | -4.24 | 0 | 0.0002 |
| ENSG00000141933 | TPGS1 | -1.275 | 0.384 | -3.323 | 0.0009 | 0.0037 |
| ENSG00000266522 |  | -1.275 | 0.475 | -2.683 | 0.0073 | 0.0218 |
| ENSG00000270607 |  | -1.274 | 0.294 | -4.33 | 0 | 0.0001 |
| ENSG00000092208 | GEMIN2 | -1.274 | 0.483 | -2.64 | 0.0083 | 0.0242 |
| ENSG00000111906 | HDDC2 | -1.273 | 0.263 | -4.835 | 0 | 0 |
| ENSG00000158859 | ADAMTS4 | -1.273 | 0.318 | -4.005 | 0.0001 | 0.0004 |
| ENSG00000170175 | CHRNB1 | -1.273 | 0.366 | -3.475 | 0.0005 | 0.0023 |
| ENSG00000268460 | LOC93429 | -1.273 | 0.489 | -2.602 | 0.0093 | 0.0265 |
| ENSG00000214013 | GANC | -1.272 | 0.252 | -5.058 | 0 | 0 |
| ENSG00000264772 |  | -1.272 | 0.446 | -2.851 | 0.0044 | 0.0142 |
| ENSG00000077312 | SNRPA | -1.269 | 0.241 | -5.254 | 0 | 0 |
| ENSG00000175701 | MTLN | -1.269 | 0.252 | -5.035 | 0 | 0 |
| ENSG00000072849 | DERL2 | -1.269 | 0.263 | -4.826 | 0 | 0 |
| ENSG00000171865 | RNASEH1 | -1.269 | 0.217 | -5.849 | 0 | 0 |
| ENSG00000146592 | CREB5 | -1.269 | 0.333 | -3.811 | 0.0001 | 0.0008 |
| ENSG00000266921 | LOC101928063 | -1.269 | 0.509 | -2.493 | 0.0127 | 0.0342 |
| ENSG00000170791 | CHCHD7 | -1.268 | 0.241 | -5.257 | 0 | 0 |
| ENSG00000221878 | PSG7 | -1.268 | 0.238 | -5.324 | 0 | 0 |
| ENSG00000119333 | DYNC2I2 | -1.267 | 0.223 | -5.692 | 0 | 0 |
| ENSG00000168040 | FADD | -1.267 | 0.407 | -3.113 | 0.0019 | 0.0068 |
| ENSG00000137573 | SULF1 | -1.266 | 0.264 | -4.786 | 0 | 0 |
| ENSG00000274963 | RN7SL600P | -1.266 | 0.476 | -2.658 | 0.0079 | 0.0232 |
| ENSG00000165675 | ENOX2 | -1.266 | 0.516 | -2.453 | 0.0142 | 0.0376 |
| ENSG00000008838 | MED24 | -1.265 | 0.305 | -4.148 | 0 | 0.0002 |
| ENSG00000258655 |  | -1.265 | 0.322 | -3.923 | 0.0001 | 0.0005 |
| ENSG00000256304 |  | -1.264 | 0.264 | -4.78 | 0 | 0 |
| ENSG00000136770 | DNAJC1 | -1.264 | 0.3 | -4.21 | 0 | 0.0002 |
| ENSG00000130244 | FAM98C | -1.264 | 0.332 | -3.801 | 0.0001 | 0.0008 |
| ENSG00000124875 | CXCL6 | -1.264 | 0.376 | -3.362 | 0.0008 | 0.0033 |
| ENSG00000148120 | AOPEP | -1.262 | 0.229 | -5.515 | 0 | 0 |
| ENSG00000223508 |  | -1.262 | 0.357 | -3.538 | 0.0004 | 0.0019 |
| ENSG00000139725 | RHOF | -1.262 | 0.442 | -2.855 | 0.0043 | 0.014 |
| ENSG00000277075 | H2AC8 | -1.261 | 0.234 | -5.4 | 0 | 0 |
| ENSG00000180340 | FZD2 | -1.261 | 0.234 | -5.399 | 0 | 0 |
| ENSG00000175221 | MED16 | -1.26 | 0.26 | -4.839 | 0 | 0 |
| ENSG00000140104 | CLBA1 | -1.26 | 0.295 | -4.268 | 0 | 0.0001 |
| ENSG00000263050 |  | -1.259 | 0.521 | -2.416 | 0.0157 | 0.0409 |
| ENSG00000122691 | TWIST1 | -1.258 | 0.25 | -5.033 | 0 | 0 |
| ENSG00000255112 | CHMP1B | -1.258 | 0.206 | -6.109 | 0 | 0 |
| ENSG00000237765 | FAM200B | -1.258 | 0.302 | -4.169 | 0 | 0.0002 |
| ENSG00000149084 | HSD17B12 | -1.257 | 0.272 | -4.622 | 0 | 0 |
| ENSG00000034053 | APBA2 | -1.257 | 0.373 | -3.373 | 0.0007 | 0.0032 |
| ENSG00000151065 | DCP1B | -1.256 | 0.294 | -4.275 | 0 | 0.0001 |
| ENSG00000100116 | GCAT | -1.256 | 0.317 | -3.957 | 0.0001 | 0.0004 |
| ENSG00000168243 | GNG4 | -1.256 | 0.529 | -2.373 | 0.0176 | 0.0451 |
| ENSG00000160310 | PRMT2 | -1.255 | 0.232 | -5.419 | 0 | 0 |
| ENSG00000111237 | VPS29 | -1.255 | 0.285 | -4.406 | 0 | 0.0001 |
| ENSG00000105393 | BABAM1 | -1.254 | 0.264 | -4.742 | 0 | 0 |
| ENSG00000164362 | TERT | -1.254 | 0.272 | -4.606 | 0 | 0 |
| ENSG00000159069 | FBXW5 | -1.254 | 0.253 | -4.962 | 0 | 0 |
| ENSG00000154079 | SDHAF4 | -1.254 | 0.35 | -3.582 | 0.0003 | 0.0016 |
| ENSG00000148450 | MSRB2 | -1.253 | 0.348 | -3.597 | 0.0003 | 0.0015 |
| ENSG00000166136 | NDUFB8 | -1.252 | 0.296 | -4.231 | 0 | 0.0002 |
| ENSG00000253305 | PCDHGB6 | -1.252 | 0.52 | -2.41 | 0.0159 | 0.0414 |
| ENSG00000271643 |  | -1.251 | 0.379 | -3.304 | 0.001 | 0.0039 |
| ENSG00000117643 | MAN1C1 | -1.249 | 0.359 | -3.481 | 0.0005 | 0.0023 |
| ENSG00000118363 | SPCS2 | -1.248 | 0.273 | -4.574 | 0 | 0 |
| ENSG00000144320 | LNPK | -1.248 | 0.259 | -4.819 | 0 | 0 |
| ENSG00000160194 | NDUFV3 | -1.248 | 0.253 | -4.927 | 0 | 0 |
| ENSG00000159216 | RUNX1 | -1.247 | 0.211 | -5.9 | 0 | 0 |
| ENSG00000253669 |  | -1.246 | 0.374 | -3.333 | 0.0009 | 0.0036 |
| ENSG00000139329 | LUM | -1.245 | 0.257 | -4.839 | 0 | 0 |
| ENSG00000120306 | CYSTM1 | -1.245 | 0.276 | -4.514 | 0 | 0.0001 |
| ENSG00000152413 | HOMER1 | -1.245 | 0.327 | -3.812 | 0.0001 | 0.0007 |
| ENSG00000130066 | SAT1 | -1.244 | 0.276 | -4.511 | 0 | 0.0001 |
| ENSG00000142188 | TMEM50B | -1.244 | 0.337 | -3.689 | 0.0002 | 0.0011 |
| ENSG00000158483 | FAM86C1P | -1.244 | 0.357 | -3.481 | 0.0005 | 0.0023 |
| ENSG00000161609 | KASH5 | -1.243 | 0.43 | -2.888 | 0.0039 | 0.0128 |
| ENSG00000100983 | GSS | -1.242 | 0.267 | -4.657 | 0 | 0 |
| ENSG00000196428 | TSC22D2 | -1.242 | 0.237 | -5.251 | 0 | 0 |
| ENSG00000238227 | TMEM250 | -1.241 | 0.219 | -5.657 | 0 | 0 |
| ENSG00000196547 | MAN2A2 | -1.241 | 0.274 | -4.531 | 0 | 0 |
| ENSG00000167513 | CDT1 | -1.241 | 0.285 | -4.355 | 0 | 0.0001 |
| ENSG00000184083 | FAM120C | -1.241 | 0.299 | -4.149 | 0 | 0.0002 |
| ENSG00000116649 | SRM | -1.24 | 0.245 | -5.069 | 0 | 0 |
| ENSG00000067064 | IDI1 | -1.24 | 0.25 | -4.971 | 0 | 0 |
| ENSG00000112078 | KCTD20 | -1.24 | 0.282 | -4.402 | 0 | 0.0001 |
| ENSG00000186056 | MATN1-AS1 | -1.24 | 0.389 | -3.191 | 0.0014 | 0.0055 |
| ENSG00000240342 | RPS2P5 | -1.239 | 0.258 | -4.799 | 0 | 0 |
| ENSG00000185974 | GRK1 | -1.239 | 0.415 | -2.985 | 0.0028 | 0.0098 |
| ENSG00000160446 | ZDHHC12 | -1.238 | 0.269 | -4.601 | 0 | 0 |
| ENSG00000186222 | BLOC1S4 | -1.237 | 0.235 | -5.262 | 0 | 0 |
| ENSG00000177917 | ARL6IP6 | -1.237 | 0.291 | -4.254 | 0 | 0.0001 |
| ENSG00000235319 |  | -1.237 | 0.467 | -2.649 | 0.0081 | 0.0237 |
| ENSG00000196437 | ZNF569 | -1.236 | 0.451 | -2.74 | 0.0061 | 0.0189 |
| ENSG00000212607 | SNORA3B | -1.236 | 0.484 | -2.553 | 0.0107 | 0.0298 |
| ENSG00000101856 | PGRMC1 | -1.235 | 0.218 | -5.667 | 0 | 0 |
| ENSG00000108846 | ABCC3 | -1.235 | 0.355 | -3.478 | 0.0005 | 0.0023 |
| ENSG00000163428 | LRRC58 | -1.233 | 0.237 | -5.208 | 0 | 0 |
| ENSG00000132002 | DNAJB1 | -1.232 | 0.216 | -5.698 | 0 | 0 |
| ENSG00000212694 | LINC01089 | -1.232 | 0.303 | -4.071 | 0 | 0.0003 |
| ENSG00000167740 | CYB5D2 | -1.231 | 0.268 | -4.593 | 0 | 0 |
| ENSG00000070476 | ZXDC | -1.231 | 0.304 | -4.047 | 0.0001 | 0.0003 |
| ENSG00000189136 | UBE2Q2P1 | -1.231 | 0.493 | -2.498 | 0.0125 | 0.0339 |
| ENSG00000231982 |  | -1.23 | 0.491 | -2.507 | 0.0122 | 0.0332 |
| ENSG00000201302 | SNORA65 | -1.229 | 0.442 | -2.779 | 0.0054 | 0.0171 |
| ENSG00000067992 | PDK3 | -1.229 | 0.528 | -2.329 | 0.0199 | 0.0497 |
| ENSG00000149657 | LSM14B | -1.228 | 0.254 | -4.836 | 0 | 0 |
| ENSG00000273391 |  | -1.228 | 0.38 | -3.228 | 0.0012 | 0.0049 |
| ENSG00000264247 | LINC00909 | -1.226 | 0.247 | -4.952 | 0 | 0 |
| ENSG00000163539 | CLASP2 | -1.226 | 0.251 | -4.879 | 0 | 0 |
| ENSG00000160124 | CCDC58 | -1.226 | 0.377 | -3.253 | 0.0011 | 0.0046 |
| ENSG00000143443 | C1orf56 | -1.226 | 0.385 | -3.183 | 0.0015 | 0.0056 |
| ENSG00000206633 | SNORA80B | -1.225 | 0.512 | -2.391 | 0.0168 | 0.0433 |
| ENSG00000133816 | MICAL2 | -1.224 | 0.232 | -5.278 | 0 | 0 |
| ENSG00000117616 | RSRP1 | -1.224 | 0.313 | -3.909 | 0.0001 | 0.0005 |
| ENSG00000236753 | MKLN1-AS | -1.223 | 0.361 | -3.391 | 0.0007 | 0.003 |
| ENSG00000172927 | MYEOV | -1.221 | 0.302 | -4.047 | 0.0001 | 0.0003 |
| ENSG00000024422 | EHD2 | -1.22 | 0.226 | -5.401 | 0 | 0 |
| ENSG00000089723 | OTUB2 | -1.219 | 0.368 | -3.311 | 0.0009 | 0.0038 |
| ENSG00000154096 | THY1 | -1.218 | 0.244 | -4.986 | 0 | 0 |
| ENSG00000162069 | BICDL2 | -1.217 | 0.267 | -4.551 | 0 | 0 |
| ENSG00000112576 | CCND3 | -1.217 | 0.244 | -4.981 | 0 | 0 |
| ENSG00000165637 | VDAC2 | -1.217 | 0.238 | -5.121 | 0 | 0 |
| ENSG00000230510 | PPP5D1 | -1.217 | 0.468 | -2.602 | 0.0093 | 0.0265 |
| ENSG00000143919 | CAMKMT | -1.217 | 0.509 | -2.39 | 0.0168 | 0.0433 |
| ENSG00000183570 | PCBP3 | -1.216 | 0.364 | -3.338 | 0.0008 | 0.0035 |
| ENSG00000100802 | C14orf93 | -1.216 | 0.47 | -2.585 | 0.0097 | 0.0276 |
| ENSG00000197497 | ZNF665 | -1.216 | 0.474 | -2.567 | 0.0103 | 0.0288 |
| ENSG00000161203 | AP2M1 | -1.215 | 0.23 | -5.29 | 0 | 0 |
| ENSG00000183048 | SLC25A10 | -1.215 | 0.365 | -3.325 | 0.0009 | 0.0037 |
| ENSG00000181029 | TRAPPC5 | -1.215 | 0.382 | -3.185 | 0.0014 | 0.0056 |
| ENSG00000171105 | INSR | -1.215 | 0.398 | -3.056 | 0.0022 | 0.008 |
| ENSG00000253958 | CLDN23 | -1.214 | 0.304 | -3.986 | 0.0001 | 0.0004 |
| ENSG00000145982 | FARS2 | -1.214 | 0.432 | -2.814 | 0.0049 | 0.0156 |
| ENSG00000213859 | KCTD11 | -1.213 | 0.234 | -5.185 | 0 | 0 |
| ENSG00000189337 | KAZN | -1.213 | 0.437 | -2.778 | 0.0055 | 0.0172 |
| ENSG00000100075 | SLC25A1 | -1.212 | 0.259 | -4.671 | 0 | 0 |
| ENSG00000160014 | CALM3 | -1.212 | 0.203 | -5.957 | 0 | 0 |
| ENSG00000168237 | GLYCTK | -1.212 | 0.379 | -3.201 | 0.0014 | 0.0053 |
| ENSG00000157184 | CPT2 | -1.211 | 0.317 | -3.822 | 0.0001 | 0.0007 |
| ENSG00000131738 | KRT33B | -1.211 | 0.413 | -2.935 | 0.0033 | 0.0113 |
| ENSG00000173960 | UBXN2A | -1.21 | 0.252 | -4.794 | 0 | 0 |
| ENSG00000105516 | DBP | -1.21 | 0.38 | -3.181 | 0.0015 | 0.0057 |
| ENSG00000262420 |  | -1.21 | 0.423 | -2.859 | 0.0043 | 0.0139 |
| ENSG00000255198 | SNHG9 | -1.209 | 0.238 | -5.087 | 0 | 0 |
| ENSG00000104442 | ARMC1 | -1.209 | 0.261 | -4.626 | 0 | 0 |
| ENSG00000172986 | GXYLT2 | -1.209 | 0.353 | -3.429 | 0.0006 | 0.0027 |
| ENSG00000128298 | BAIAP2L2 | -1.208 | 0.389 | -3.111 | 0.0019 | 0.0069 |
| ENSG00000230882 |  | -1.208 | 0.452 | -2.673 | 0.0075 | 0.0223 |
| ENSG00000127080 | IPPK | -1.207 | 0.346 | -3.494 | 0.0005 | 0.0022 |
| ENSG00000058799 | YIPF1 | -1.206 | 0.278 | -4.343 | 0 | 0.0001 |
| ENSG00000157823 | AP3S2 | -1.206 | 0.287 | -4.201 | 0 | 0.0002 |
| ENSG00000167302 | TEPSIN | -1.206 | 0.311 | -3.879 | 0.0001 | 0.0006 |
| ENSG00000183023 | SLC8A1 | -1.205 | 0.262 | -4.593 | 0 | 0 |
| ENSG00000120333 | MRPS14 | -1.205 | 0.267 | -4.519 | 0 | 0.0001 |
| ENSG00000177674 | AGTRAP | -1.205 | 0.318 | -3.785 | 0.0002 | 0.0008 |
| ENSG00000259818 |  | -1.205 | 0.344 | -3.506 | 0.0005 | 0.0021 |
| ENSG00000162595 | DIRAS3 | -1.205 | 0.471 | -2.556 | 0.0106 | 0.0296 |
| ENSG00000101220 | C20orf27 | -1.203 | 0.279 | -4.304 | 0 | 0.0001 |
| ENSG00000124217 | MOCS3 | -1.202 | 0.244 | -4.93 | 0 | 0 |
| ENSG00000205277 | MUC12 | -1.202 | 0.409 | -2.937 | 0.0033 | 0.0112 |
| ENSG00000164054 | SHISA5 | -1.201 | 0.209 | -5.738 | 0 | 0 |
| ENSG00000156381 | ANKRD9 | -1.201 | 0.245 | -4.908 | 0 | 0 |
| ENSG00000122641 | INHBA | -1.201 | 0.277 | -4.339 | 0 | 0.0001 |
| ENSG00000160703 | NLRX1 | -1.201 | 0.38 | -3.157 | 0.0016 | 0.006 |
| ENSG00000186166 | CENATAC | -1.2 | 0.24 | -4.99 | 0 | 0 |
| ENSG00000100221 | JOSD1 | -1.2 | 0.22 | -5.462 | 0 | 0 |
| ENSG00000150995 | ITPR1 | -1.2 | 0.43 | -2.79 | 0.0053 | 0.0167 |
| ENSG00000172057 | ORMDL3 | -1.199 | 0.28 | -4.279 | 0 | 0.0001 |
| ENSG00000091592 | NLRP1 | -1.199 | 0.275 | -4.355 | 0 | 0.0001 |
| ENSG00000205639 | MFSD2B | -1.199 | 0.391 | -3.069 | 0.0022 | 0.0078 |
| ENSG00000176994 | SMCR8 | -1.198 | 0.276 | -4.34 | 0 | 0.0001 |
| ENSG00000138279 | ANXA7 | -1.197 | 0.214 | -5.588 | 0 | 0 |
| ENSG00000261188 |  | -1.197 | 0.346 | -3.464 | 0.0005 | 0.0024 |
| ENSG00000135919 | SERPINE2 | -1.196 | 0.295 | -4.06 | 0 | 0.0003 |
| ENSG00000175274 | TP53I11 | -1.195 | 0.255 | -4.678 | 0 | 0 |
| ENSG00000228474 | OST4 | -1.195 | 0.237 | -5.049 | 0 | 0 |
| ENSG00000182636 | NDN | -1.194 | 0.306 | -3.907 | 0.0001 | 0.0005 |
| ENSG00000168917 | SLC35G2 | -1.193 | 0.253 | -4.707 | 0 | 0 |
| ENSG00000100906 | NFKBIA | -1.193 | 0.218 | -5.472 | 0 | 0 |
| ENSG00000183955 | KMT5A | -1.192 | 0.245 | -4.857 | 0 | 0 |
| ENSG00000169047 | IRS1 | -1.192 | 0.237 | -5.029 | 0 | 0 |
| ENSG00000279069 |  | -1.192 | 0.401 | -2.972 | 0.003 | 0.0102 |
| ENSG00000100934 | SEC23A | -1.189 | 0.253 | -4.707 | 0 | 0 |
| ENSG00000196576 | PLXNB2 | -1.188 | 0.206 | -5.777 | 0 | 0 |
| ENSG00000274897 |  | -1.188 | 0.467 | -2.545 | 0.0109 | 0.0304 |
| ENSG00000268670 |  | -1.188 | 0.483 | -2.46 | 0.0139 | 0.037 |
| ENSG00000156860 | FBRS | -1.187 | 0.246 | -4.824 | 0 | 0 |
| ENSG00000075218 | GTSE1 | -1.187 | 0.287 | -4.132 | 0 | 0.0002 |
| ENSG00000270820 |  | -1.187 | 0.377 | -3.152 | 0.0016 | 0.0061 |
| ENSG00000152147 | GEMIN6 | -1.186 | 0.231 | -5.131 | 0 | 0 |
| ENSG00000148926 | ADM | -1.185 | 0.212 | -5.574 | 0 | 0 |
| ENSG00000145050 | MANF | -1.185 | 0.241 | -4.918 | 0 | 0 |
| ENSG00000117122 | MFAP2 | -1.185 | 0.345 | -3.439 | 0.0006 | 0.0026 |
| ENSG00000103187 | COTL1 | -1.184 | 0.215 | -5.513 | 0 | 0 |
| ENSG00000167995 | BEST1 | -1.184 | 0.267 | -4.436 | 0 | 0.0001 |
| ENSG00000254682 |  | -1.184 | 0.406 | -2.917 | 0.0035 | 0.0119 |
| ENSG00000077380 | DYNC1I2 | -1.183 | 0.231 | -5.123 | 0 | 0 |
| ENSG00000123352 | SPATS2 | -1.182 | 0.293 | -4.035 | 0.0001 | 0.0003 |
| ENSG00000167173 | C15orf39 | -1.182 | 0.318 | -3.715 | 0.0002 | 0.001 |
| ENSG00000118194 | TNNT2 | -1.182 | 0.422 | -2.797 | 0.0052 | 0.0163 |
| ENSG00000215193 | PEX26 | -1.181 | 0.28 | -4.22 | 0 | 0.0002 |
| ENSG00000171448 | ZBTB26 | -1.181 | 0.406 | -2.91 | 0.0036 | 0.0121 |
| ENSG00000236049 | LINC01920 | -1.179 | 0.397 | -2.968 | 0.003 | 0.0103 |
| ENSG00000140406 | TLNRD1 | -1.178 | 0.227 | -5.198 | 0 | 0 |
| ENSG00000116132 | PRRX1 | -1.178 | 0.222 | -5.31 | 0 | 0 |
| ENSG00000212747 | RTL8B | -1.178 | 0.313 | -3.769 | 0.0002 | 0.0009 |
| ENSG00000178695 | KCTD12 | -1.177 | 0.256 | -4.606 | 0 | 0 |
| ENSG00000168575 | SLC20A2 | -1.177 | 0.263 | -4.468 | 0 | 0.0001 |
| ENSG00000108666 | C17orf75 | -1.177 | 0.317 | -3.711 | 0.0002 | 0.0011 |
| ENSG00000082996 | RNF13 | -1.174 | 0.265 | -4.425 | 0 | 0.0001 |
| ENSG00000168310 | IRF2 | -1.174 | 0.329 | -3.571 | 0.0004 | 0.0017 |
| ENSG00000149346 | SLX4IP | -1.174 | 0.42 | -2.798 | 0.0051 | 0.0163 |
| ENSG00000128602 | SMO | -1.174 | 0.429 | -2.738 | 0.0062 | 0.019 |
| ENSG00000204070 | SYS1 | -1.173 | 0.255 | -4.598 | 0 | 0 |
| ENSG00000127922 | SEM1 | -1.173 | 0.215 | -5.453 | 0 | 0 |
| ENSG00000133935 | ERG28 | -1.173 | 0.269 | -4.357 | 0 | 0.0001 |
| ENSG00000173933 | RBM4 | -1.173 | 0.263 | -4.461 | 0 | 0.0001 |
| ENSG00000226380 |  | -1.172 | 0.222 | -5.268 | 0 | 0 |
| ENSG00000228782 |  | -1.172 | 0.436 | -2.691 | 0.0071 | 0.0214 |
| ENSG00000105281 | SLC1A5 | -1.171 | 0.244 | -4.789 | 0 | 0 |
| ENSG00000102893 | PHKB | -1.171 | 0.256 | -4.578 | 0 | 0 |
| ENSG00000182552 | RWDD4 | -1.171 | 0.292 | -4.008 | 0.0001 | 0.0004 |
| ENSG00000142396 | ERVK3-1 | -1.171 | 0.448 | -2.613 | 0.009 | 0.0258 |
| ENSG00000164292 | RHOBTB3 | -1.17 | 0.247 | -4.746 | 0 | 0 |
| ENSG00000236581 | STARD13-AS | -1.169 | 0.285 | -4.102 | 0 | 0.0003 |
| ENSG00000171703 | TCEA2 | -1.169 | 0.306 | -3.824 | 0.0001 | 0.0007 |
| ENSG00000221963 | APOL6 | -1.169 | 0.338 | -3.46 | 0.0005 | 0.0024 |
| ENSG00000116667 | C1orf21 | -1.168 | 0.304 | -3.838 | 0.0001 | 0.0007 |
| ENSG00000169992 | NLGN2 | -1.168 | 0.31 | -3.765 | 0.0002 | 0.0009 |
| ENSG00000181915 | ADO | -1.167 | 0.242 | -4.82 | 0 | 0 |
| ENSG00000179456 | ZBTB18 | -1.167 | 0.341 | -3.427 | 0.0006 | 0.0027 |
| ENSG00000162415 | ZSWIM5 | -1.167 | 0.36 | -3.241 | 0.0012 | 0.0047 |
| ENSG00000197191 | CYSRT1 | -1.167 | 0.434 | -2.691 | 0.0071 | 0.0214 |
| ENSG00000178726 | THBD | -1.166 | 0.238 | -4.902 | 0 | 0 |
| ENSG00000221500 | SNORD100 | -1.166 | 0.476 | -2.451 | 0.0142 | 0.0377 |
| ENSG00000160602 | NEK8 | -1.164 | 0.337 | -3.451 | 0.0006 | 0.0025 |
| ENSG00000229689 |  | -1.164 | 0.365 | -3.184 | 0.0015 | 0.0056 |
| ENSG00000175087 | PDIK1L | -1.164 | 0.373 | -3.117 | 0.0018 | 0.0068 |
| ENSG00000111850 | SMIM8 | -1.164 | 0.464 | -2.51 | 0.0121 | 0.0329 |
| ENSG00000126267 | COX6B1 | -1.163 | 0.21 | -5.546 | 0 | 0 |
| ENSG00000155034 | FBXL18 | -1.163 | 0.296 | -3.931 | 0.0001 | 0.0005 |
| ENSG00000213347 | MXD3 | -1.163 | 0.301 | -3.869 | 0.0001 | 0.0006 |
| ENSG00000165716 | DIPK1B | -1.163 | 0.364 | -3.192 | 0.0014 | 0.0055 |
| ENSG00000182325 | FBXL6 | -1.162 | 0.262 | -4.435 | 0 | 0.0001 |
| ENSG00000172731 | LRRC20 | -1.162 | 0.459 | -2.533 | 0.0113 | 0.0312 |
| ENSG00000124574 | ABCC10 | -1.16 | 0.284 | -4.09 | 0 | 0.0003 |
| ENSG00000127580 | WDR24 | -1.16 | 0.293 | -3.966 | 0.0001 | 0.0004 |
| ENSG00000173918 | C1QTNF1 | -1.16 | 0.315 | -3.684 | 0.0002 | 0.0012 |
| ENSG00000162775 | RBM15 | -1.159 | 0.249 | -4.663 | 0 | 0 |
| ENSG00000105612 | DNASE2 | -1.159 | 0.271 | -4.283 | 0 | 0.0001 |
| ENSG00000110172 | CHORDC1 | -1.159 | 0.304 | -3.818 | 0.0001 | 0.0007 |
| ENSG00000156642 | NPTN | -1.157 | 0.262 | -4.411 | 0 | 0.0001 |
| ENSG00000173269 | MMRN2 | -1.157 | 0.359 | -3.22 | 0.0013 | 0.005 |
| ENSG00000114850 | SSR3 | -1.156 | 0.274 | -4.214 | 0 | 0.0002 |
| ENSG00000014164 | ZC3H3 | -1.156 | 0.386 | -2.991 | 0.0028 | 0.0097 |
| ENSG00000259370 | LOC105370854 | -1.156 | 0.48 | -2.409 | 0.016 | 0.0415 |
| ENSG00000231925 | TAPBP | -1.155 | 0.212 | -5.437 | 0 | 0 |
| ENSG00000138029 | HADHB | -1.155 | 0.216 | -5.353 | 0 | 0 |
| ENSG00000105255 | FSD1 | -1.155 | 0.484 | -2.384 | 0.0171 | 0.0439 |
| ENSG00000139211 | AMIGO2 | -1.154 | 0.266 | -4.342 | 0 | 0.0001 |
| ENSG00000137691 | CFAP300 | -1.154 | 0.418 | -2.76 | 0.0058 | 0.018 |
| ENSG00000144115 | THNSL2 | -1.154 | 0.418 | -2.76 | 0.0058 | 0.018 |
| ENSG00000174132 | FAM174A | -1.153 | 0.45 | -2.561 | 0.0104 | 0.0293 |
| ENSG00000263426 | RN7SL471P | -1.152 | 0.234 | -4.919 | 0 | 0 |
| ENSG00000151176 | PLBD2 | -1.152 | 0.243 | -4.748 | 0 | 0 |
| ENSG00000278993 |  | -1.152 | 0.459 | -2.51 | 0.0121 | 0.0329 |
| ENSG00000126861 | OMG | -1.151 | 0.442 | -2.603 | 0.0092 | 0.0264 |
| ENSG00000002822 | MAD1L1 | -1.149 | 0.274 | -4.195 | 0 | 0.0002 |
| ENSG00000124496 | TRERF1 | -1.148 | 0.368 | -3.124 | 0.0018 | 0.0066 |
| ENSG00000113269 | RNF130 | -1.147 | 0.24 | -4.769 | 0 | 0 |
| ENSG00000146373 | RNF217 | -1.147 | 0.342 | -3.355 | 0.0008 | 0.0034 |
| ENSG00000176087 | SLC35A4 | -1.146 | 0.223 | -5.138 | 0 | 0 |
| ENSG00000111817 | DSE | -1.146 | 0.285 | -4.021 | 0.0001 | 0.0004 |
| ENSG00000180447 | GAS1 | -1.146 | 0.327 | -3.508 | 0.0005 | 0.0021 |
| ENSG00000005059 | MCUB | -1.144 | 0.291 | -3.93 | 0.0001 | 0.0005 |
| ENSG00000100722 | ZC3H14 | -1.144 | 0.305 | -3.746 | 0.0002 | 0.0009 |
| ENSG00000156795 | NTAQ1 | -1.144 | 0.304 | -3.758 | 0.0002 | 0.0009 |
| ENSG00000168916 | ZNF608 | -1.144 | 0.356 | -3.211 | 0.0013 | 0.0052 |
| ENSG00000113389 | NPR3 | -1.143 | 0.308 | -3.715 | 0.0002 | 0.001 |
| ENSG00000275835 | TUBGCP5 | -1.143 | 0.309 | -3.701 | 0.0002 | 0.0011 |
| ENSG00000187837 | H1-2 | -1.142 | 0.212 | -5.397 | 0 | 0 |
| ENSG00000140391 | TSPAN3 | -1.142 | 0.26 | -4.394 | 0 | 0.0001 |
| ENSG00000182310 | SPACA6 | -1.142 | 0.39 | -2.927 | 0.0034 | 0.0115 |
| ENSG00000116771 | AGMAT | -1.142 | 0.457 | -2.498 | 0.0125 | 0.0338 |
| ENSG00000184956 | MUC6 | -1.141 | 0.346 | -3.299 | 0.001 | 0.004 |
| ENSG00000182158 | CREB3L2 | -1.14 | 0.227 | -5.018 | 0 | 0 |
| ENSG00000138061 | CYP1B1 | -1.14 | 0.26 | -4.384 | 0 | 0.0001 |
| ENSG00000141562 | NARF | -1.14 | 0.286 | -3.983 | 0.0001 | 0.0004 |
| ENSG00000162076 | FLYWCH2 | -1.14 | 0.309 | -3.695 | 0.0002 | 0.0011 |
| ENSG00000145781 | COMMD10 | -1.139 | 0.346 | -3.292 | 0.001 | 0.0041 |
| ENSG00000105738 | SIPA1L3 | -1.138 | 0.256 | -4.438 | 0 | 0.0001 |
| ENSG00000271737 |  | -1.138 | 0.475 | -2.394 | 0.0167 | 0.0429 |
| ENSG00000119242 | CCDC92 | -1.137 | 0.292 | -3.893 | 0.0001 | 0.0006 |
| ENSG00000160392 | C19orf47 | -1.137 | 0.321 | -3.54 | 0.0004 | 0.0019 |
| ENSG00000134461 | ANKRD16 | -1.137 | 0.396 | -2.873 | 0.0041 | 0.0134 |
| ENSG00000102898 | NUTF2 | -1.136 | 0.231 | -4.922 | 0 | 0 |
| ENSG00000139190 | VAMP1 | -1.136 | 0.362 | -3.137 | 0.0017 | 0.0064 |
| ENSG00000261971 |  | -1.136 | 0.459 | -2.477 | 0.0133 | 0.0356 |
| ENSG00000120129 | DUSP1 | -1.135 | 0.217 | -5.22 | 0 | 0 |
| ENSG00000223482 | NUTM2A-AS1 | -1.135 | 0.323 | -3.52 | 0.0004 | 0.002 |
| ENSG00000105327 | BBC3 | -1.134 | 0.219 | -5.179 | 0 | 0 |
| ENSG00000168002 | POLR2G | -1.133 | 0.227 | -4.987 | 0 | 0 |
| ENSG00000141564 | RPTOR | -1.133 | 0.245 | -4.633 | 0 | 0 |
| ENSG00000090316 | MAEA | -1.133 | 0.309 | -3.668 | 0.0002 | 0.0012 |
| ENSG00000204923 | FBXO48 | -1.133 | 0.408 | -2.779 | 0.0054 | 0.0171 |
| ENSG00000101182 | PSMA7 | -1.132 | 0.227 | -4.992 | 0 | 0 |
| ENSG00000263843 | LOC100287042 | -1.132 | 0.398 | -2.847 | 0.0044 | 0.0143 |
| ENSG00000131507 | NDFIP1 | -1.131 | 0.232 | -4.868 | 0 | 0 |
| ENSG00000103227 | LMF1 | -1.131 | 0.319 | -3.547 | 0.0004 | 0.0018 |
| ENSG00000272288 | ILRUN-AS1 | -1.131 | 0.361 | -3.133 | 0.0017 | 0.0065 |
| ENSG00000213337 | ANKRD39 | -1.131 | 0.419 | -2.702 | 0.0069 | 0.0208 |
| ENSG00000173327 | MAP3K11 | -1.13 | 0.261 | -4.334 | 0 | 0.0001 |
| ENSG00000185475 | TMEM179B | -1.13 | 0.275 | -4.113 | 0 | 0.0003 |
| ENSG00000124098 | FAM210B | -1.13 | 0.285 | -3.966 | 0.0001 | 0.0004 |
| ENSG00000157224 | CLDN12 | -1.129 | 0.213 | -5.288 | 0 | 0 |
| ENSG00000131408 | NR1H2 | -1.129 | 0.233 | -4.847 | 0 | 0 |
| ENSG00000118503 | TNFAIP3 | -1.128 | 0.201 | -5.613 | 0 | 0 |
| ENSG00000147364 | FBXO25 | -1.128 | 0.288 | -3.909 | 0.0001 | 0.0005 |
| ENSG00000172543 | CTSW | -1.128 | 0.296 | -3.816 | 0.0001 | 0.0007 |
| ENSG00000205485 | LOC100133091 | -1.128 | 0.308 | -3.666 | 0.0002 | 0.0012 |
| ENSG00000198752 | CDC42BPB | -1.127 | 0.239 | -4.709 | 0 | 0 |
| ENSG00000151725 | CENPU | -1.127 | 0.219 | -5.137 | 0 | 0 |
| ENSG00000173581 | CCDC106 | -1.127 | 0.315 | -3.575 | 0.0004 | 0.0017 |
| ENSG00000198205 | ZXDA | -1.127 | 0.345 | -3.265 | 0.0011 | 0.0044 |
| ENSG00000119685 | TTLL5 | -1.126 | 0.315 | -3.575 | 0.0003 | 0.0017 |
| ENSG00000235109 | ZSCAN31 | -1.125 | 0.418 | -2.689 | 0.0072 | 0.0215 |
| ENSG00000183287 | CCBE1 | -1.124 | 0.268 | -4.193 | 0 | 0.0002 |
| ENSG00000101457 | DNTTIP1 | -1.124 | 0.303 | -3.711 | 0.0002 | 0.0011 |
| ENSG00000214160 | ALG3 | -1.123 | 0.223 | -5.038 | 0 | 0 |
| ENSG00000254473 | LOC105376114 | -1.123 | 0.359 | -3.124 | 0.0018 | 0.0066 |
| ENSG00000233436 | BTBD18 | -1.123 | 0.37 | -3.035 | 0.0024 | 0.0085 |
| ENSG00000069869 | NEDD4 | -1.122 | 0.208 | -5.387 | 0 | 0 |
| ENSG00000130520 | LSM4 | -1.122 | 0.265 | -4.237 | 0 | 0.0002 |
| ENSG00000006606 | CCL26 | -1.12 | 0.255 | -4.398 | 0 | 0.0001 |
| ENSG00000163328 | GPR155 | -1.12 | 0.474 | -2.36 | 0.0183 | 0.0464 |
| ENSG00000108797 | CNTNAP1 | -1.119 | 0.272 | -4.112 | 0 | 0.0003 |
| ENSG00000132763 | MMACHC | -1.119 | 0.29 | -3.861 | 0.0001 | 0.0006 |
| ENSG00000179588 | ZFPM1 | -1.119 | 0.288 | -3.887 | 0.0001 | 0.0006 |
| ENSG00000185338 | SOCS1 | -1.119 | 0.364 | -3.075 | 0.0021 | 0.0076 |
| ENSG00000125352 | RNF113A | -1.118 | 0.233 | -4.792 | 0 | 0 |
| ENSG00000153234 | NR4A2 | -1.118 | 0.256 | -4.375 | 0 | 0.0001 |
| ENSG00000062582 | MRPS24 | -1.118 | 0.331 | -3.372 | 0.0007 | 0.0032 |
| ENSG00000133195 | SLC39A11 | -1.118 | 0.387 | -2.887 | 0.0039 | 0.0129 |
| ENSG00000143368 | SF3B4 | -1.117 | 0.201 | -5.562 | 0 | 0 |
| ENSG00000127666 | TICAM1 | -1.117 | 0.273 | -4.089 | 0 | 0.0003 |
| ENSG00000162496 | DHRS3 | -1.117 | 0.432 | -2.583 | 0.0098 | 0.0277 |
| ENSG00000141449 | GREB1L | -1.116 | 0.308 | -3.63 | 0.0003 | 0.0014 |
| ENSG00000133107 | TRPC4 | -1.116 | 0.34 | -3.282 | 0.001 | 0.0042 |
| ENSG00000273148 |  | -1.116 | 0.364 | -3.062 | 0.0022 | 0.0079 |
| ENSG00000181722 | ZBTB20 | -1.115 | 0.274 | -4.068 | 0 | 0.0003 |
| ENSG00000084774 | CAD | -1.115 | 0.274 | -4.069 | 0 | 0.0003 |
| ENSG00000187173 | LCE2A | -1.115 | 0.285 | -3.917 | 0.0001 | 0.0005 |
| ENSG00000166548 | TK2 | -1.115 | 0.294 | -3.794 | 0.0001 | 0.0008 |
| ENSG00000143621 | ILF2 | -1.114 | 0.195 | -5.702 | 0 | 0 |
| ENSG00000272533 | SNORA28 | -1.114 | 0.471 | -2.367 | 0.0179 | 0.0457 |
| ENSG00000275202 |  | -1.113 | 0.305 | -3.65 | 0.0003 | 0.0013 |
| ENSG00000224870 | MRPL20-AS1 | -1.112 | 0.342 | -3.251 | 0.0012 | 0.0046 |
| ENSG00000172757 | CFL1 | -1.111 | 0.252 | -4.414 | 0 | 0.0001 |
| ENSG00000114626 | ABTB1 | -1.111 | 0.383 | -2.903 | 0.0037 | 0.0123 |
| ENSG00000271646 |  | -1.111 | 0.419 | -2.653 | 0.008 | 0.0235 |
| ENSG00000138814 | PPP3CA | -1.11 | 0.273 | -4.067 | 0 | 0.0003 |
| ENSG00000153363 | LINC00467 | -1.11 | 0.383 | -2.897 | 0.0038 | 0.0125 |
| ENSG00000183935 | HTR7P1 | -1.109 | 0.233 | -4.754 | 0 | 0 |
| ENSG00000167964 | RAB26 | -1.108 | 0.325 | -3.405 | 0.0007 | 0.0029 |
| ENSG00000109501 | WFS1 | -1.107 | 0.287 | -3.86 | 0.0001 | 0.0006 |
| ENSG00000167157 | PRRX2 | -1.107 | 0.296 | -3.74 | 0.0002 | 0.001 |
| ENSG00000235453 | SMIM27 | -1.107 | 0.337 | -3.282 | 0.001 | 0.0042 |
| ENSG00000249915 | PDCD6 | -1.105 | 0.253 | -4.371 | 0 | 0.0001 |
| ENSG00000183779 | ZNF703 | -1.105 | 0.319 | -3.465 | 0.0005 | 0.0024 |
| ENSG00000051180 | RAD51 | -1.105 | 0.353 | -3.128 | 0.0018 | 0.0066 |
| ENSG00000170684 | ZNF296 | -1.105 | 0.391 | -2.823 | 0.0048 | 0.0153 |
| ENSG00000218336 | TENM3 | -1.105 | 0.394 | -2.808 | 0.005 | 0.0159 |
| ENSG00000091986 | CCDC80 | -1.104 | 0.233 | -4.736 | 0 | 0 |
| ENSG00000213390 | ARHGAP19 | -1.104 | 0.407 | -2.714 | 0.0066 | 0.0202 |
| ENSG00000064666 | CNN2 | -1.103 | 0.229 | -4.825 | 0 | 0 |
| ENSG00000249592 | LOC100129917 | -1.103 | 0.458 | -2.408 | 0.016 | 0.0416 |
| ENSG00000171867 | PRNP | -1.102 | 0.196 | -5.635 | 0 | 0 |
| ENSG00000188042 | ARL4C | -1.101 | 0.246 | -4.477 | 0 | 0.0001 |
| ENSG00000282057 |  | -1.101 | 0.318 | -3.468 | 0.0005 | 0.0024 |
| ENSG00000233937 | CTC-338M12.4 | -1.101 | 0.337 | -3.267 | 0.0011 | 0.0044 |
| ENSG00000278238 |  | -1.101 | 0.363 | -3.034 | 0.0024 | 0.0086 |
| ENSG00000073756 | PTGS2 | -1.1 | 0.251 | -4.375 | 0 | 0.0001 |
| ENSG00000186111 | PIP5K1C | -1.1 | 0.253 | -4.339 | 0 | 0.0001 |
| ENSG00000163964 | PIGX | -1.1 | 0.33 | -3.335 | 0.0009 | 0.0036 |
| ENSG00000142208 | AKT1 | -1.099 | 0.225 | -4.892 | 0 | 0 |
| ENSG00000163191 | S100A11 | -1.099 | 0.252 | -4.356 | 0 | 0.0001 |
| ENSG00000119138 | KLF9 | -1.098 | 0.309 | -3.553 | 0.0004 | 0.0018 |
| ENSG00000079931 | MOXD1 | -1.097 | 0.238 | -4.613 | 0 | 0 |
| ENSG00000013297 | CLDN11 | -1.097 | 0.252 | -4.359 | 0 | 0.0001 |
| ENSG00000109861 | CTSC | -1.097 | 0.274 | -4.009 | 0.0001 | 0.0004 |
| ENSG00000109680 | TBC1D19 | -1.097 | 0.318 | -3.444 | 0.0006 | 0.0025 |
| ENSG00000270084 |  | -1.097 | 0.386 | -2.841 | 0.0045 | 0.0146 |
| ENSG00000108344 | PSMD3 | -1.096 | 0.199 | -5.508 | 0 | 0 |
| ENSG00000215271 | HOMEZ | -1.094 | 0.439 | -2.489 | 0.0128 | 0.0346 |
| ENSG00000157617 | C2CD2 | -1.093 | 0.282 | -3.883 | 0.0001 | 0.0006 |
| ENSG00000185236 | RAB11B | -1.093 | 0.308 | -3.553 | 0.0004 | 0.0018 |
| ENSG00000085063 | CD59 | -1.092 | 0.271 | -4.03 | 0.0001 | 0.0003 |
| ENSG00000189184 | PCDH18 | -1.092 | 0.293 | -3.728 | 0.0002 | 0.001 |
| ENSG00000237017 |  | -1.092 | 0.428 | -2.551 | 0.0107 | 0.0299 |
| ENSG00000104853 | CLPTM1 | -1.091 | 0.221 | -4.945 | 0 | 0 |
| ENSG00000136997 | MYC | -1.091 | 0.251 | -4.345 | 0 | 0.0001 |
| ENSG00000148344 | PTGES | -1.091 | 0.324 | -3.368 | 0.0008 | 0.0032 |
| ENSG00000083937 | CHMP2B | -1.09 | 0.24 | -4.549 | 0 | 0 |
| ENSG00000114450 | GNB4 | -1.09 | 0.267 | -4.086 | 0 | 0.0003 |
| ENSG00000077549 | CAPZB | -1.09 | 0.277 | -3.93 | 0.0001 | 0.0005 |
| ENSG00000178127 | NDUFV2 | -1.09 | 0.34 | -3.202 | 0.0014 | 0.0053 |
| ENSG00000169372 | CRADD | -1.09 | 0.396 | -2.755 | 0.0059 | 0.0182 |
| ENSG00000105971 | CAV2 | -1.089 | 0.234 | -4.658 | 0 | 0 |
| ENSG00000262251 |  | -1.089 | 0.343 | -3.174 | 0.0015 | 0.0058 |
| ENSG00000172992 | DCAKD | -1.088 | 0.306 | -3.558 | 0.0004 | 0.0018 |
| ENSG00000063601 | MTMR1 | -1.086 | 0.316 | -3.442 | 0.0006 | 0.0026 |
| ENSG00000231721 | LINC-PINT | -1.085 | 0.241 | -4.508 | 0 | 0.0001 |
| ENSG00000167315 | ACAA2 | -1.085 | 0.262 | -4.147 | 0 | 0.0002 |
| ENSG00000102158 | MAGT1 | -1.085 | 0.289 | -3.75 | 0.0002 | 0.0009 |
| ENSG00000205189 | ZBTB10 | -1.085 | 0.31 | -3.497 | 0.0005 | 0.0021 |
| ENSG00000260267 |  | -1.085 | 0.368 | -2.949 | 0.0032 | 0.0109 |
| ENSG00000154217 | PITPNC1 | -1.083 | 0.3 | -3.611 | 0.0003 | 0.0015 |
| ENSG00000171827 | ZNF570 | -1.083 | 0.302 | -3.586 | 0.0003 | 0.0016 |
| ENSG00000137274 | BPHL | -1.083 | 0.349 | -3.105 | 0.0019 | 0.007 |
| ENSG00000180914 | OXTR | -1.082 | 0.287 | -3.765 | 0.0002 | 0.0009 |
| ENSG00000029639 | TFB1M | -1.082 | 0.345 | -3.139 | 0.0017 | 0.0064 |
| ENSG00000166845 | C18orf54 | -1.082 | 0.356 | -3.04 | 0.0024 | 0.0084 |
| ENSG00000113721 | PDGFRB | -1.081 | 0.235 | -4.602 | 0 | 0 |
| ENSG00000066044 | ELAVL1 | -1.081 | 0.244 | -4.423 | 0 | 0.0001 |
| ENSG00000101194 | SLC17A9 | -1.081 | 0.257 | -4.204 | 0 | 0.0002 |
| ENSG00000156110 | ADK | -1.081 | 0.282 | -3.834 | 0.0001 | 0.0007 |
| ENSG00000276170 | LOC101929494 | -1.081 | 0.352 | -3.071 | 0.0021 | 0.0077 |
| ENSG00000164096 | C4orf3 | -1.08 | 0.237 | -4.565 | 0 | 0 |
| ENSG00000183668 | PSG9 | -1.08 | 0.262 | -4.126 | 0 | 0.0002 |
| ENSG00000186832 | KRT16 | -1.08 | 0.451 | -2.395 | 0.0166 | 0.0428 |
| ENSG00000268001 | CARD8-AS1 | -1.079 | 0.378 | -2.857 | 0.0043 | 0.0139 |
| ENSG00000103152 | MPG | -1.078 | 0.272 | -3.966 | 0.0001 | 0.0004 |
| ENSG00000111445 | RFC5 | -1.078 | 0.311 | -3.463 | 0.0005 | 0.0024 |
| ENSG00000149476 | TKFC | -1.077 | 0.255 | -4.217 | 0 | 0.0002 |
| ENSG00000064932 | SBNO2 | -1.077 | 0.289 | -3.722 | 0.0002 | 0.001 |
| ENSG00000171174 | RBKS | -1.077 | 0.297 | -3.621 | 0.0003 | 0.0014 |
| ENSG00000198598 | MMP17 | -1.077 | 0.301 | -3.575 | 0.0004 | 0.0017 |
| ENSG00000047230 | CTPS2 | -1.077 | 0.39 | -2.761 | 0.0058 | 0.0179 |
| ENSG00000058063 | ATP11B | -1.076 | 0.243 | -4.435 | 0 | 0.0001 |
| ENSG00000136720 | HS6ST1 | -1.076 | 0.345 | -3.119 | 0.0018 | 0.0067 |
| ENSG00000117632 | STMN1 | -1.075 | 0.223 | -4.815 | 0 | 0 |
| ENSG00000147535 | PLPP5 | -1.075 | 0.255 | -4.216 | 0 | 0.0002 |
| ENSG00000107872 | FBXL15 | -1.075 | 0.359 | -2.991 | 0.0028 | 0.0097 |
| ENSG00000145284 | SCD5 | -1.075 | 0.426 | -2.524 | 0.0116 | 0.0318 |
| ENSG00000168476 | REEP4 | -1.074 | 0.267 | -4.024 | 0.0001 | 0.0003 |
| ENSG00000068912 | ERLEC1 | -1.073 | 0.249 | -4.31 | 0 | 0.0001 |
| ENSG00000102081 | FMR1 | -1.073 | 0.267 | -4.011 | 0.0001 | 0.0004 |
| ENSG00000136238 | RAC1 | -1.072 | 0.235 | -4.567 | 0 | 0 |
| ENSG00000167380 | ZNF226 | -1.072 | 0.249 | -4.313 | 0 | 0.0001 |
| ENSG00000092841 | MYL6 | -1.07 | 0.237 | -4.514 | 0 | 0.0001 |
| ENSG00000133316 | WDR74 | -1.07 | 0.318 | -3.365 | 0.0008 | 0.0033 |
| ENSG00000178015 | GPR150 | -1.07 | 0.406 | -2.632 | 0.0085 | 0.0246 |
| ENSG00000272512 |  | -1.07 | 0.444 | -2.408 | 0.0161 | 0.0416 |
| ENSG00000100726 | TELO2 | -1.069 | 0.241 | -4.441 | 0 | 0.0001 |
| ENSG00000105419 | MEIS3 | -1.069 | 0.283 | -3.771 | 0.0002 | 0.0009 |
| ENSG00000233237 | LINC00472 | -1.069 | 0.394 | -2.712 | 0.0067 | 0.0203 |
| ENSG00000114125 | RNF7 | -1.068 | 0.275 | -3.889 | 0.0001 | 0.0006 |
| ENSG00000205707 | ETFRF1 | -1.068 | 0.322 | -3.312 | 0.0009 | 0.0038 |
| ENSG00000125378 | BMP4 | -1.067 | 0.338 | -3.152 | 0.0016 | 0.0061 |
| ENSG00000213221 | DNLZ | -1.067 | 0.426 | -2.503 | 0.0123 | 0.0334 |
| ENSG00000257511 | LOC100420981 | -1.067 | 0.435 | -2.451 | 0.0142 | 0.0377 |
| ENSG00000225511 |  | -1.066 | 0.206 | -5.171 | 0 | 0 |
| ENSG00000132313 | MRPL35 | -1.066 | 0.29 | -3.678 | 0.0002 | 0.0012 |
| ENSG00000202538 | RNU4-2 | -1.066 | 0.3 | -3.55 | 0.0004 | 0.0018 |
| ENSG00000177156 | TALDO1 | -1.065 | 0.23 | -4.639 | 0 | 0 |
| ENSG00000278272 |  | -1.065 | 0.228 | -4.665 | 0 | 0 |
| ENSG00000163170 | BOLA3 | -1.065 | 0.25 | -4.255 | 0 | 0.0001 |
| ENSG00000232533 |  | -1.064 | 0.228 | -4.676 | 0 | 0 |
| ENSG00000118680 | MYL12B | -1.064 | 0.24 | -4.441 | 0 | 0.0001 |
| ENSG00000126458 | RRAS | -1.064 | 0.268 | -3.975 | 0.0001 | 0.0004 |
| ENSG00000155957 | TMBIM4 | -1.064 | 0.351 | -3.033 | 0.0024 | 0.0086 |
| ENSG00000059377 | TBXAS1 | -1.064 | 0.437 | -2.433 | 0.015 | 0.0393 |
| ENSG00000003402 | CFLAR | -1.063 | 0.243 | -4.382 | 0 | 0.0001 |
| ENSG00000254685 | FPGT | -1.063 | 0.362 | -2.935 | 0.0033 | 0.0113 |
| ENSG00000065057 | NTHL1 | -1.063 | 0.423 | -2.511 | 0.012 | 0.0328 |
| ENSG00000086062 | B4GALT1 | -1.062 | 0.302 | -3.52 | 0.0004 | 0.002 |
| ENSG00000165175 | MID1IP1 | -1.061 | 0.228 | -4.648 | 0 | 0 |
| ENSG00000279696 |  | -1.06 | 0.261 | -4.066 | 0 | 0.0003 |
| ENSG00000227372 | TP73-AS1 | -1.06 | 0.315 | -3.369 | 0.0008 | 0.0032 |
| ENSG00000109775 | UFSP2 | -1.06 | 0.321 | -3.306 | 0.0009 | 0.0039 |
| ENSG00000157429 | ZNF19 | -1.06 | 0.384 | -2.762 | 0.0057 | 0.0179 |
| ENSG00000241399 | CD302 | -1.06 | 0.447 | -2.373 | 0.0176 | 0.0451 |
| ENSG00000099904 | ZDHHC8 | -1.059 | 0.225 | -4.714 | 0 | 0 |
| ENSG00000177732 | SOX12 | -1.059 | 0.269 | -3.937 | 0.0001 | 0.0005 |
| ENSG00000167700 | MFSD3 | -1.059 | 0.287 | -3.688 | 0.0002 | 0.0011 |
| ENSG00000114698 | PLSCR4 | -1.059 | 0.307 | -3.448 | 0.0006 | 0.0025 |
| ENSG00000136819 | C9orf78 | -1.058 | 0.201 | -5.258 | 0 | 0 |
| ENSG00000116473 | RAP1A | -1.058 | 0.263 | -4.025 | 0.0001 | 0.0003 |
| ENSG00000099341 | PSMD8 | -1.058 | 0.268 | -3.943 | 0.0001 | 0.0005 |
| ENSG00000126858 | RHOT1 | -1.058 | 0.273 | -3.88 | 0.0001 | 0.0006 |
| ENSG00000152778 | IFIT5 | -1.058 | 0.275 | -3.853 | 0.0001 | 0.0006 |
| ENSG00000256268 | LINC02454 | -1.058 | 0.351 | -3.013 | 0.0026 | 0.0091 |
| ENSG00000172613 | RAD9A | -1.057 | 0.258 | -4.098 | 0 | 0.0003 |
| ENSG00000255248 | MIR100HG | -1.057 | 0.264 | -3.998 | 0.0001 | 0.0004 |
| ENSG00000123358 | NR4A1 | -1.057 | 0.284 | -3.728 | 0.0002 | 0.001 |
| ENSG00000070501 | POLB | -1.057 | 0.302 | -3.507 | 0.0005 | 0.0021 |
| ENSG00000188783 | PRELP | -1.057 | 0.331 | -3.198 | 0.0014 | 0.0054 |
| ENSG00000272263 |  | -1.057 | 0.42 | -2.515 | 0.0119 | 0.0325 |
| ENSG00000151500 | THYN1 | -1.055 | 0.266 | -3.963 | 0.0001 | 0.0004 |
| ENSG00000277639 | LOC105371267 | -1.055 | 0.291 | -3.621 | 0.0003 | 0.0014 |
| ENSG00000168763 | CNNM3 | -1.055 | 0.434 | -2.433 | 0.015 | 0.0394 |
| ENSG00000126457 | PRMT1 | -1.054 | 0.207 | -5.095 | 0 | 0 |
| ENSG00000196605 | ZNF846 | -1.054 | 0.314 | -3.356 | 0.0008 | 0.0033 |
| ENSG00000040933 | INPP4A | -1.053 | 0.274 | -3.844 | 0.0001 | 0.0007 |
| ENSG00000106460 | TMEM106B | -1.052 | 0.26 | -4.046 | 0.0001 | 0.0003 |
| ENSG00000144136 | SLC20A1 | -1.051 | 0.252 | -4.167 | 0 | 0.0002 |
| ENSG00000129009 | ISLR | -1.051 | 0.402 | -2.612 | 0.009 | 0.0259 |
| ENSG00000246705 | H2AJ | -1.05 | 0.239 | -4.391 | 0 | 0.0001 |
| ENSG00000265972 | TXNIP | -1.05 | 0.27 | -3.891 | 0.0001 | 0.0006 |
| ENSG00000032742 | IFT88 | -1.05 | 0.358 | -2.931 | 0.0034 | 0.0114 |
| ENSG00000124920 | MYRF | -1.05 | 0.361 | -2.905 | 0.0037 | 0.0122 |
| ENSG00000269486 |  | -1.05 | 0.449 | -2.338 | 0.0194 | 0.0487 |
| ENSG00000146826 | MAP11 | -1.049 | 0.274 | -3.821 | 0.0001 | 0.0007 |
| ENSG00000228436 | LOC105378663 | -1.049 | 0.401 | -2.614 | 0.0089 | 0.0257 |
| ENSG00000196663 | TECPR2 | -1.048 | 0.28 | -3.738 | 0.0002 | 0.001 |
| ENSG00000113504 | SLC12A7 | -1.047 | 0.326 | -3.214 | 0.0013 | 0.0051 |
| ENSG00000181513 | ACBD4 | -1.047 | 0.406 | -2.577 | 0.01 | 0.0281 |
| ENSG00000128791 | TWSG1 | -1.046 | 0.217 | -4.82 | 0 | 0 |
| ENSG00000148468 | FAM171A1 | -1.046 | 0.261 | -4.013 | 0.0001 | 0.0004 |
| ENSG00000110906 | KCTD10 | -1.045 | 0.23 | -4.539 | 0 | 0 |
| ENSG00000198081 | ZBTB14 | -1.045 | 0.389 | -2.687 | 0.0072 | 0.0216 |
| ENSG00000149633 | KIAA1755 | -1.045 | 0.44 | -2.377 | 0.0175 | 0.0447 |
| ENSG00000172061 | LRRC15 | -1.044 | 0.249 | -4.185 | 0 | 0.0002 |
| ENSG00000148180 | GSN | -1.044 | 0.255 | -4.09 | 0 | 0.0003 |
| ENSG00000011132 | APBA3 | -1.044 | 0.277 | -3.766 | 0.0002 | 0.0009 |
| ENSG00000112592 | TBP | -1.044 | 0.289 | -3.61 | 0.0003 | 0.0015 |
| ENSG00000173409 | ARV1 | -1.044 | 0.314 | -3.32 | 0.0009 | 0.0037 |
| ENSG00000137714 | FDX1 | -1.044 | 0.34 | -3.076 | 0.0021 | 0.0076 |
| ENSG00000161395 | PGAP3 | -1.044 | 0.357 | -2.922 | 0.0035 | 0.0117 |
| ENSG00000141971 | MVB12A | -1.043 | 0.262 | -3.986 | 0.0001 | 0.0004 |
| ENSG00000263823 |  | -1.043 | 0.391 | -2.665 | 0.0077 | 0.0228 |
| ENSG00000238045 |  | -1.043 | 0.408 | -2.555 | 0.0106 | 0.0296 |
| ENSG00000132661 | NXT1 | -1.042 | 0.216 | -4.816 | 0 | 0 |
| ENSG00000108518 | PFN1 | -1.042 | 0.234 | -4.452 | 0 | 0.0001 |
| ENSG00000095383 | TBC1D2 | -1.042 | 0.272 | -3.827 | 0.0001 | 0.0007 |
| ENSG00000162065 | TBC1D24 | -1.041 | 0.352 | -2.954 | 0.0031 | 0.0107 |
| ENSG00000228506 |  | -1.041 | 0.399 | -2.608 | 0.0091 | 0.0261 |
| ENSG00000178026 | LRRC75B | -1.041 | 0.42 | -2.476 | 0.0133 | 0.0356 |
| ENSG00000152558 | TMEM123 | -1.04 | 0.241 | -4.316 | 0 | 0.0001 |
| ENSG00000280383 |  | -1.04 | 0.272 | -3.825 | 0.0001 | 0.0007 |
| ENSG00000092098 | RNF31 | -1.04 | 0.303 | -3.427 | 0.0006 | 0.0027 |
| ENSG00000070731 | ST6GALNAC2 | -1.04 | 0.311 | -3.344 | 0.0008 | 0.0035 |
| ENSG00000113140 | SPARC | -1.039 | 0.244 | -4.268 | 0 | 0.0001 |
| ENSG00000187676 | B3GLCT | -1.039 | 0.296 | -3.509 | 0.0005 | 0.0021 |
| ENSG00000198142 | SOWAHC | -1.038 | 0.233 | -4.465 | 0 | 0.0001 |
| ENSG00000162585 | FAAP20 | -1.038 | 0.249 | -4.166 | 0 | 0.0002 |
| ENSG00000185000 | DGAT1 | -1.037 | 0.268 | -3.863 | 0.0001 | 0.0006 |
| ENSG00000174501 | ANKRD36C | -1.037 | 0.396 | -2.619 | 0.0088 | 0.0254 |
| ENSG00000086598 | TMED2 | -1.036 | 0.227 | -4.571 | 0 | 0 |
| ENSG00000134490 | TMEM241 | -1.036 | 0.36 | -2.878 | 0.004 | 0.0132 |
| ENSG00000185201 | IFITM2 | -1.035 | 0.272 | -3.8 | 0.0001 | 0.0008 |
| ENSG00000115137 | DNAJC27 | -1.035 | 0.374 | -2.767 | 0.0057 | 0.0177 |
| ENSG00000164754 | RAD21 | -1.034 | 0.232 | -4.463 | 0 | 0.0001 |
| ENSG00000276107 |  | -1.034 | 0.271 | -3.811 | 0.0001 | 0.0008 |
| ENSG00000120992 | LYPLA1 | -1.034 | 0.302 | -3.424 | 0.0006 | 0.0027 |
| ENSG00000275126 | H4C13 | -1.033 | 0.414 | -2.497 | 0.0125 | 0.0339 |
| ENSG00000196526 | AFAP1 | -1.032 | 0.234 | -4.4 | 0 | 0.0001 |
| ENSG00000205531 | NAP1L4 | -1.032 | 0.232 | -4.455 | 0 | 0.0001 |
| ENSG00000126062 | TMEM115 | -1.032 | 0.245 | -4.213 | 0 | 0.0002 |
| ENSG00000245571 | FAM111A-DT | -1.032 | 0.403 | -2.562 | 0.0104 | 0.0292 |
| ENSG00000106993 | CDC37L1 | -1.031 | 0.245 | -4.204 | 0 | 0.0002 |
| ENSG00000237190 | CDKN2AIPNL | -1.031 | 0.26 | -3.971 | 0.0001 | 0.0004 |
| ENSG00000249353 |  | -1.031 | 0.397 | -2.594 | 0.0095 | 0.027 |
| ENSG00000185551 | NR2F2 | -1.03 | 0.266 | -3.87 | 0.0001 | 0.0006 |
| ENSG00000170390 | DCLK2 | -1.03 | 0.326 | -3.162 | 0.0016 | 0.006 |
| ENSG00000153936 | HS2ST1 | -1.03 | 0.337 | -3.057 | 0.0022 | 0.008 |
| ENSG00000143641 | GALNT2 | -1.029 | 0.264 | -3.899 | 0.0001 | 0.0005 |
| ENSG00000120137 | PANK3 | -1.028 | 0.229 | -4.48 | 0 | 0.0001 |
| ENSG00000188603 | CLN3 | -1.028 | 0.401 | -2.563 | 0.0104 | 0.0291 |
| ENSG00000145715 | RASA1 | -1.027 | 0.282 | -3.647 | 0.0003 | 0.0013 |
| ENSG00000170919 |  | -1.027 | 0.323 | -3.181 | 0.0015 | 0.0057 |
| ENSG00000136381 | IREB2 | -1.026 | 0.25 | -4.105 | 0 | 0.0003 |
| ENSG00000117697 | NSL1 | -1.026 | 0.281 | -3.648 | 0.0003 | 0.0013 |
| ENSG00000008300 | CELSR3 | -1.025 | 0.244 | -4.196 | 0 | 0.0002 |
| ENSG00000100097 | LGALS1 | -1.025 | 0.241 | -4.247 | 0 | 0.0002 |
| ENSG00000167615 | LENG8 | -1.025 | 0.283 | -3.627 | 0.0003 | 0.0014 |
| ENSG00000107796 | ACTA2 | -1.023 | 0.249 | -4.116 | 0 | 0.0002 |
| ENSG00000003436 | TFPI | -1.023 | 0.285 | -3.594 | 0.0003 | 0.0016 |
| ENSG00000229619 | MBNL1-AS1 | -1.023 | 0.325 | -3.146 | 0.0017 | 0.0062 |
| ENSG00000144840 | RABL3 | -1.022 | 0.306 | -3.341 | 0.0008 | 0.0035 |
| ENSG00000174695 | TMEM167A | -1.021 | 0.264 | -3.875 | 0.0001 | 0.0006 |
| ENSG00000161533 | ACOX1 | -1.02 | 0.22 | -4.63 | 0 | 0 |
| ENSG00000278705 | H4C2 | -1.02 | 0.294 | -3.47 | 0.0005 | 0.0023 |
| ENSG00000130560 | UBAC1 | -1.019 | 0.268 | -3.802 | 0.0001 | 0.0008 |
| ENSG00000226803 |  | -1.019 | 0.288 | -3.544 | 0.0004 | 0.0018 |
| ENSG00000102934 | PLLP | -1.018 | 0.318 | -3.198 | 0.0014 | 0.0054 |
| ENSG00000078124 | ACER3 | -1.017 | 0.272 | -3.739 | 0.0002 | 0.001 |
| ENSG00000170458 | CD14 | -1.017 | 0.336 | -3.026 | 0.0025 | 0.0087 |
| ENSG00000162298 | SYVN1 | -1.016 | 0.276 | -3.685 | 0.0002 | 0.0012 |
| ENSG00000156017 | CARNMT1 | -1.016 | 0.283 | -3.594 | 0.0003 | 0.0016 |
| ENSG00000213846 |  | -1.016 | 0.288 | -3.531 | 0.0004 | 0.0019 |
| ENSG00000173926 | MARCHF3 | -1.016 | 0.3 | -3.393 | 0.0007 | 0.003 |
| ENSG00000261338 |  | -1.016 | 0.4 | -2.536 | 0.0112 | 0.031 |
| ENSG00000101084 | RAB5IF | -1.015 | 0.254 | -4 | 0.0001 | 0.0004 |
| ENSG00000132589 | FLOT2 | -1.015 | 0.282 | -3.593 | 0.0003 | 0.0016 |
| ENSG00000014138 | POLA2 | -1.015 | 0.294 | -3.448 | 0.0006 | 0.0025 |
| ENSG00000152795 | HNRNPDL | -1.014 | 0.2 | -5.076 | 0 | 0 |
| ENSG00000149679 | CABLES2 | -1.014 | 0.254 | -3.989 | 0.0001 | 0.0004 |
| ENSG00000117751 | PPP1R8 | -1.013 | 0.238 | -4.248 | 0 | 0.0001 |
| ENSG00000104695 | PPP2CB | -1.013 | 0.23 | -4.398 | 0 | 0.0001 |
| ENSG00000119650 | IFT43 | -1.013 | 0.348 | -2.906 | 0.0037 | 0.0122 |
| ENSG00000184867 | ARMCX2 | -1.012 | 0.241 | -4.202 | 0 | 0.0002 |
| ENSG00000149600 | COMMD7 | -1.012 | 0.262 | -3.87 | 0.0001 | 0.0006 |
| ENSG00000157014 | TATDN2 | -1.012 | 0.275 | -3.677 | 0.0002 | 0.0012 |
| ENSG00000184384 | MAML2 | -1.012 | 0.321 | -3.153 | 0.0016 | 0.0061 |
| ENSG00000175220 | ARHGAP1 | -1.011 | 0.28 | -3.608 | 0.0003 | 0.0015 |
| ENSG00000196569 | LAMA2 | -1.01 | 0.303 | -3.336 | 0.0009 | 0.0036 |
| ENSG00000236859 | NIFK-AS1 | -1.01 | 0.373 | -2.709 | 0.0067 | 0.0204 |
| ENSG00000140365 | COMMD4 | -1.009 | 0.226 | -4.468 | 0 | 0.0001 |
| ENSG00000100478 | AP4S1 | -1.009 | 0.309 | -3.269 | 0.0011 | 0.0043 |
| ENSG00000144802 | NFKBIZ | -1.008 | 0.219 | -4.606 | 0 | 0 |
| ENSG00000164649 | CDCA7L | -1.008 | 0.328 | -3.077 | 0.0021 | 0.0076 |
| ENSG00000169925 | BRD3 | -1.008 | 0.407 | -2.477 | 0.0132 | 0.0355 |
| ENSG00000125841 | NRSN2 | -1.007 | 0.269 | -3.748 | 0.0002 | 0.0009 |
| ENSG00000177042 | TMEM80 | -1.006 | 0.26 | -3.873 | 0.0001 | 0.0006 |
| ENSG00000152240 | HAUS1 | -1.006 | 0.268 | -3.751 | 0.0002 | 0.0009 |
| ENSG00000185112 | FAM43A | -1.006 | 0.316 | -3.178 | 0.0015 | 0.0057 |
| ENSG00000099817 | POLR2E | -1.005 | 0.228 | -4.402 | 0 | 0.0001 |
| ENSG00000173482 | PTPRM | -1.004 | 0.26 | -3.866 | 0.0001 | 0.0006 |
| ENSG00000147202 | DIAPH2 | -1.004 | 0.347 | -2.893 | 0.0038 | 0.0126 |
| ENSG00000198832 | SELENOM | -1.002 | 0.247 | -4.063 | 0 | 0.0003 |
| ENSG00000183671 | GPR1 | -1.002 | 0.269 | -3.724 | 0.0002 | 0.001 |
| ENSG00000176055 | MBLAC2 | -1.002 | 0.367 | -2.728 | 0.0064 | 0.0195 |
| ENSG00000065268 | WDR18 | -1.001 | 0.29 | -3.455 | 0.0006 | 0.0025 |
| ENSG00000205740 | LOC107984285 | -1.001 | 0.361 | -2.776 | 0.0055 | 0.0173 |
|  |  |  |  |  |  |  |
|  |  |  |  |  |  |  |
|  |  |  |  |  |  |  |
|  |  |  |  |  |  |  |
|  |  |  |  |  |  |  |
|  |  |  |  |  |  |  |
|  |  |  |  |  |  |  |
|  |  |  |  |  |  |  |
|  |  |  |  |  |  |  |
|  |  |  |  |  |  |  |
|  |  |  |  |  |  |  |
|  |  |  |  |  |  |  |
|  |  |  |  |  |  |  |
|  |  |  |  |  |  |  |
|  |  |  |  |  |  |  |
|  |  |  |  |  |  |  |
|  |  |  |  |  |  |  |
|  |  |  |  |  |  |  |
|  |  |  |  |  |  |  |
|  |  |  |  |  |  |  |
|  |  |  |  |  |  |  |
|  |  |  |  |  |  |  |
|  |  |  |  |  |  |  |
|  |  |  |  |  |  |  |
|  |  |  |  |  |  |  |
|  |  |  |  |  |  |  |
|  |  |  |  |  |  |  |
|  |  |  |  |  |  |  |
|  |  |  |  |  |  |  |
|  |  |  |  |  |  |  |
|  |  |  |  |  |  |  |
|  |  |  |  |  |  |  |
|  |  |  |  |  |  |  |
|  |  |  |  |  |  |  |
|  |  |  |  |  |  |  |
|  |  |  |  |  |  |  |
|  |  |  |  |  |  |  |
|  |  |  |  |  |  |  |
|  |  |  |  |  |  |  |
|  |  |  |  |  |  |  |
|  |  |  |  |  |  |  |
|  |  |  |  |  |  |  |
|  |  |  |  |  |  |  |
|  |  |  |  |  |  |  |
|  |  |  |  |  |  |  |
|  |  |  |  |  |  |  |
|  |  |  |  |  |  |  |
|  |  |  |  |  |  |  |
|  |  |  |  |  |  |  |
|  |  |  |  |  |  |  |
|  |  |  |  |  |  |  |
|  |  |  |  |  |  |  |
|  |  |  |  |  |  |  |
|  |  |  |  |  |  |  |
|  |  |  |  |  |  |  |
|  |  |  |  |  |  |  |
|  |  |  |  |  |  |  |
|  |  |  |  |  |  |  |
|  |  |  |  |  |  |  |
|  |  |  |  |  |  |  |
|  |  |  |  |  |  |  |
|  |  |  |  |  |  |  |
|  |  |  |  |  |  |  |
|  |  |  |  |  |  |  |
|  |  |  |  |  |  |  |
|  |  |  |  |  |  |  |
|  |  |  |  |  |  |  |
|  |  |  |  |  |  |  |
|  |  |  |  |  |  |  |
|  |  |  |  |  |  |  |
|  |  |  |  |  |  |  |
|  |  |  |  |  |  |  |
|  |  |  |  |  |  |  |
|  |  |  |  |  |  |  |
|  |  |  |  |  |  |  |
|  |  |  |  |  |  |  |
|  |  |  |  |  |  |  |
|  |  |  |  |  |  |  |
|  |  |  |  |  |  |  |
|  |  |  |  |  |  |  |
|  |  |  |  |  |  |  |
|  |  |  |  |  |  |  |
|  |  |  |  |  |  |  |
|  |  |  |  |  |  |  |
|  |  |  |  |  |  |  |
|  |  |  |  |  |  |  |
|  |  |  |  |  |  |  |
|  |  |  |  |  |  |  |
|  |  |  |  |  |  |  |
|  |  |  |  |  |  |  |
|  |  |  |  |  |  |  |
|  |  |  |  |  |  |  |
|  |  |  |  |  |  |  |
|  |  |  |  |  |  |  |
|  |  |  |  |  |  |  |
|  |  |  |  |  |  |  |
|  |  |  |  |  |  |  |
|  |  |  |  |  |  |  |
|  |  |  |  |  |  |  |
|  |  |  |  |  |  |  |
|  |  |  |  |  |  |  |
|  |  |  |  |  |  |  |
|  |  |  |  |  |  |  |
|  |  |  |  |  |  |  |
|  |  |  |  |  |  |  |
|  |  |  |  |  |  |  |
|  |  |  |  |  |  |  |
|  |  |  |  |  |  |  |
|  |  |  |  |  |  |  |
|  |  |  |  |  |  |  |
|  |  |  |  |  |  |  |
|  |  |  |  |  |  |  |
|  |  |  |  |  |  |  |
|  |  |  |  |  |  |  |
|  |  |  |  |  |  |  |
|  |  |  |  |  |  |  |
|  |  |  |  |  |  |  |
|  |  |  |  |  |  |  |
|  |  |  |  |  |  |  |
|  |  |  |  |  |  |  |
|  |  |  |  |  |  |  |
|  |  |  |  |  |  |  |
|  |  |  |  |  |  |  |
|  |  |  |  |  |  |  |
|  |  |  |  |  |  |  |
|  |  |  |  |  |  |  |
|  |  |  |  |  |  |  |
|  |  |  |  |  |  |  |
|  |  |  |  |  |  |  |
|  |  |  |  |  |  |  |
|  |  |  |  |  |  |  |
|  |  |  |  |  |  |  |
|  |  |  |  |  |  |  |
|  |  |  |  |  |  |  |
|  |  |  |  |  |  |  |
|  |  |  |  |  |  |  |
|  |  |  |  |  |  |  |
|  |  |  |  |  |  |  |
|  |  |  |  |  |  |  |
|  |  |  |  |  |  |  |
|  |  |  |  |  |  |  |
|  |  |  |  |  |  |  |
|  |  |  |  |  |  |  |
|  |  |  |  |  |  |  |
|  |  |  |  |  |  |  |
|  |  |  |  |  |  |  |
|  |  |  |  |  |  |  |
|  |  |  |  |  |  |  |
|  |  |  |  |  |  |  |
|  |  |  |  |  |  |  |
|  |  |  |  |  |  |  |
|  |  |  |  |  |  |  |
|  |  |  |  |  |  |  |
|  |  |  |  |  |  |  |
|  |  |  |  |  |  |  |
|  |  |  |  |  |  |  |
|  |  |  |  |  |  |  |
|  |  |  |  |  |  |  |
|  |  |  |  |  |  |  |
|  |  |  |  |  |  |  |
|  |  |  |  |  |  |  |
|  |  |  |  |  |  |  |
|  |  |  |  |  |  |  |
|  |  |  |  |  |  |  |
|  |  |  |  |  |  |  |
|  |  |  |  |  |  |  |
|  |  |  |  |  |  |  |
|  |  |  |  |  |  |  |
|  |  |  |  |  |  |  |
|  |  |  |  |  |  |  |
|  |  |  |  |  |  |  |
|  |  |  |  |  |  |  |
|  |  |  |  |  |  |  |
|  |  |  |  |  |  |  |
|  |  |  |  |  |  |  |
|  |  |  |  |  |  |  |
|  |  |  |  |  |  |  |
|  |  |  |  |  |  |  |
|  |  |  |  |  |  |  |
|  |  |  |  |  |  |  |
|  |  |  |  |  |  |  |
|  |  |  |  |  |  |  |
|  |  |  |  |  |  |  |
|  |  |  |  |  |  |  |
|  |  |  |  |  |  |  |
|  |  |  |  |  |  |  |
|  |  |  |  |  |  |  |
|  |  |  |  |  |  |  |
|  |  |  |  |  |  |  |
|  |  |  |  |  |  |  |
|  |  |  |  |  |  |  |
|  |  |  |  |  |  |  |
|  |  |  |  |  |  |  |
|  |  |  |  |  |  |  |
|  |  |  |  |  |  |  |
|  |  |  |  |  |  |  |
|  |  |  |  |  |  |  |
|  |  |  |  |  |  |  |
|  |  |  |  |  |  |  |
|  |  |  |  |  |  |  |
|  |  |  |  |  |  |  |
|  |  |  |  |  |  |  |
|  |  |  |  |  |  |  |
|  |  |  |  |  |  |  |
|  |  |  |  |  |  |  |
|  |  |  |  |  |  |  |
|  |  |  |  |  |  |  |
|  |  |  |  |  |  |  |
|  |  |  |  |  |  |  |
|  |  |  |  |  |  |  |
|  |  |  |  |  |  |  |
|  |  |  |  |  |  |  |
|  |  |  |  |  |  |  |
|  |  |  |  |  |  |  |
|  |  |  |  |  |  |  |
|  |  |  |  |  |  |  |
|  |  |  |  |  |  |  |
|  |  |  |  |  |  |  |
|  |  |  |  |  |  |  |
|  |  |  |  |  |  |  |
|  |  |  |  |  |  |  |
|  |  |  |  |  |  |  |
|  |  |  |  |  |  |  |
|  |  |  |  |  |  |  |
|  |  |  |  |  |  |  |
|  |  |  |  |  |  |  |
|  |  |  |  |  |  |  |
|  |  |  |  |  |  |  |
|  |  |  |  |  |  |  |
|  |  |  |  |  |  |  |
|  |  |  |  |  |  |  |
|  |  |  |  |  |  |  |
|  |  |  |  |  |  |  |
|  |  |  |  |  |  |  |
|  |  |  |  |  |  |  |
|  |  |  |  |  |  |  |
|  |  |  |  |  |  |  |
|  |  |  |  |  |  |  |
|  |  |  |  |  |  |  |
|  |  |  |  |  |  |  |
|  |  |  |  |  |  |  |
|  |  |  |  |  |  |  |
|  |  |  |  |  |  |  |
|  |  |  |  |  |  |  |
|  |  |  |  |  |  |  |
|  |  |  |  |  |  |  |
|  |  |  |  |  |  |  |
|  |  |  |  |  |  |  |
|  |  |  |  |  |  |  |
|  |  |  |  |  |  |  |
|  |  |  |  |  |  |  |
|  |  |  |  |  |  |  |
|  |  |  |  |  |  |  |
|  |  |  |  |  |  |  |
|  |  |  |  |  |  |  |
|  |  |  |  |  |  |  |
|  |  |  |  |  |  |  |
|  |  |  |  |  |  |  |
|  |  |  |  |  |  |  |
|  |  |  |  |  |  |  |
|  |  |  |  |  |  |  |
|  |  |  |  |  |  |  |
|  |  |  |  |  |  |  |
|  |  |  |  |  |  |  |
|  |  |  |  |  |  |  |
|  |  |  |  |  |  |  |
|  |  |  |  |  |  |  |
|  |  |  |  |  |  |  |
|  |  |  |  |  |  |  |
|  |  |  |  |  |  |  |
|  |  |  |  |  |  |  |
|  |  |  |  |  |  |  |
|  |  |  |  |  |  |  |
|  |  |  |  |  |  |  |
|  |  |  |  |  |  |  |
|  |  |  |  |  |  |  |
|  |  |  |  |  |  |  |
|  |  |  |  |  |  |  |
|  |  |  |  |  |  |  |
|  |  |  |  |  |  |  |
|  |  |  |  |  |  |  |
|  |  |  |  |  |  |  |
|  |  |  |  |  |  |  |
|  |  |  |  |  |  |  |
|  |  |  |  |  |  |  |
|  |  |  |  |  |  |  |
|  |  |  |  |  |  |  |
|  |  |  |  |  |  |  |
|  |  |  |  |  |  |  |
|  |  |  |  |  |  |  |
|  |  |  |  |  |  |  |
|  |  |  |  |  |  |  |
|  |  |  |  |  |  |  |
|  |  |  |  |  |  |  |
|  |  |  |  |  |  |  |
|  |  |  |  |  |  |  |
|  |  |  |  |  |  |  |
|  |  |  |  |  |  |  |
|  |  |  |  |  |  |  |
|  |  |  |  |  |  |  |
|  |  |  |  |  |  |  |
|  |  |  |  |  |  |  |
|  |  |  |  |  |  |  |
|  |  |  |  |  |  |  |
|  |  |  |  |  |  |  |
|  |  |  |  |  |  |  |
|  |  |  |  |  |  |  |
|  |  |  |  |  |  |  |
|  |  |  |  |  |  |  |
|  |  |  |  |  |  |  |
|  |  |  |  |  |  |  |
|  |  |  |  |  |  |  |
|  |  |  |  |  |  |  |
|  |  |  |  |  |  |  |
|  |  |  |  |  |  |  |
|  |  |  |  |  |  |  |
|  |  |  |  |  |  |  |
|  |  |  |  |  |  |  |
|  |  |  |  |  |  |  |
|  |  |  |  |  |  |  |
|  |  |  |  |  |  |  |
|  |  |  |  |  |  |  |
|  |  |  |  |  |  |  |
|  |  |  |  |  |  |  |
|  |  |  |  |  |  |  |
|  |  |  |  |  |  |  |
|  |  |  |  |  |  |  |
|  |  |  |  |  |  |  |
|  |  |  |  |  |  |  |
|  |  |  |  |  |  |  |
|  |  |  |  |  |  |  |
|  |  |  |  |  |  |  |
|  |  |  |  |  |  |  |
|  |  |  |  |  |  |  |
|  |  |  |  |  |  |  |
|  |  |  |  |  |  |  |
|  |  |  |  |  |  |  |
|  |  |  |  |  |  |  |
|  |  |  |  |  |  |  |
|  |  |  |  |  |  |  |
|  |  |  |  |  |  |  |
|  |  |  |  |  |  |  |
|  |  |  |  |  |  |  |
|  |  |  |  |  |  |  |
|  |  |  |  |  |  |  |
|  |  |  |  |  |  |  |
|  |  |  |  |  |  |  |
|  |  |  |  |  |  |  |
|  |  |  |  |  |  |  |
|  |  |  |  |  |  |  |
|  |  |  |  |  |  |  |
|  |  |  |  |  |  |  |
|  |  |  |  |  |  |  |
|  |  |  |  |  |  |  |
|  |  |  |  |  |  |  |
|  |  |  |  |  |  |  |
|  |  |  |  |  |  |  |
|  |  |  |  |  |  |  |
|  |  |  |  |  |  |  |
|  |  |  |  |  |  |  |
|  |  |  |  |  |  |  |
|  |  |  |  |  |  |  |
|  |  |  |  |  |  |  |
|  |  |  |  |  |  |  |
|  |  |  |  |  |  |  |
|  |  |  |  |  |  |  |
|  |  |  |  |  |  |  |
|  |  |  |  |  |  |  |
|  |  |  |  |  |  |  |
|  |  |  |  |  |  |  |
|  |  |  |  |  |  |  |
|  |  |  |  |  |  |  |
|  |  |  |  |  |  |  |
|  |  |  |  |  |  |  |
|  |  |  |  |  |  |  |
|  |  |  |  |  |  |  |
|  |  |  |  |  |  |  |
|  |  |  |  |  |  |  |
|  |  |  |  |  |  |  |
|  |  |  |  |  |  |  |
|  |  |  |  |  |  |  |
|  |  |  |  |  |  |  |
|  |  |  |  |  |  |  |
|  |  |  |  |  |  |  |
|  |  |  |  |  |  |  |
|  |  |  |  |  |  |  |
|  |  |  |  |  |  |  |
|  |  |  |  |  |  |  |
|  |  |  |  |  |  |  |
|  |  |  |  |  |  |  |
|  |  |  |  |  |  |  |
|  |  |  |  |  |  |  |
|  |  |  |  |  |  |  |
|  |  |  |  |  |  |  |
|  |  |  |  |  |  |  |
|  |  |  |  |  |  |  |
|  |  |  |  |  |  |  |
|  |  |  |  |  |  |  |
|  |  |  |  |  |  |  |
|  |  |  |  |  |  |  |
|  |  |  |  |  |  |  |
|  |  |  |  |  |  |  |
|  |  |  |  |  |  |  |
|  |  |  |  |  |  |  |
|  |  |  |  |  |  |  |
|  |  |  |  |  |  |  |
|  |  |  |  |  |  |  |
|  |  |  |  |  |  |  |
|  |  |  |  |  |  |  |
|  |  |  |  |  |  |  |
|  |  |  |  |  |  |  |
|  |  |  |  |  |  |  |
|  |  |  |  |  |  |  |
|  |  |  |  |  |  |  |
|  |  |  |  |  |  |  |
|  |  |  |  |  |  |  |
|  |  |  |  |  |  |  |
|  |  |  |  |  |  |  |
|  |  |  |  |  |  |  |
|  |  |  |  |  |  |  |
|  |  |  |  |  |  |  |
|  |  |  |  |  |  |  |
|  |  |  |  |  |  |  |
|  |  |  |  |  |  |  |
|  |  |  |  |  |  |  |
|  |  |  |  |  |  |  |
|  |  |  |  |  |  |  |
|  |  |  |  |  |  |  |
|  |  |  |  |  |  |  |
|  |  |  |  |  |  |  |
|  |  |  |  |  |  |  |
|  |  |  |  |  |  |  |
|  |  |  |  |  |  |  |
|  |  |  |  |  |  |  |
|  |  |  |  |  |  |  |
|  |  |  |  |  |  |  |
|  |  |  |  |  |  |  |
|  |  |  |  |  |  |  |
|  |  |  |  |  |  |  |
|  |  |  |  |  |  |  |
|  |  |  |  |  |  |  |
|  |  |  |  |  |  |  |
|  |  |  |  |  |  |  |
|  |  |  |  |  |  |  |
|  |  |  |  |  |  |  |
|  |  |  |  |  |  |  |
|  |  |  |  |  |  |  |
|  |  |  |  |  |  |  |
|  |  |  |  |  |  |  |
|  |  |  |  |  |  |  |
|  |  |  |  |  |  |  |
|  |  |  |  |  |  |  |
|  |  |  |  |  |  |  |
|  |  |  |  |  |  |  |
|  |  |  |  |  |  |  |
|  |  |  |  |  |  |  |
|  |  |  |  |  |  |  |
|  |  |  |  |  |  |  |
|  |  |  |  |  |  |  |
|  |  |  |  |  |  |  |
|  |  |  |  |  |  |  |
